# In vivo self-assembled siRNA as a modality for combination therapy of ulcerative colitis

**DOI:** 10.1038/s41467-022-33436-0

**Published:** 2022-09-28

**Authors:** Xinyan Zhou, Mengchao Yu, Luzhen Ma, Jinyu Fu, Jingwei Guo, Jieqiong Lei, Zheng Fu, Yong Fu, Qipeng Zhang, Chen-Yu Zhang, Xi Chen

**Affiliations:** 1 Nanjing Drum Tower Hospital Center of Molecular Diagnostic and Therapy, State Key Laboratory of Pharmaceutical Biotechnology, Jiangsu Engineering Research Center for MicroRNA Biology and Biotechnology, NJU Advanced Institute of Life Sciences (NAILS), School of Life Sciences, Nanjing University, 210023 Nanjing, Jiangsu China; 2 Central Laboratories, Department of Gastroenterology, Qingdao Municipal Hospital, Qingdao University, 266061 Qingdao, China; 3 Chemistry and Biomedicine Innovation Center (ChemBIC), Nanjing University, 210023 Nanjing, Jiangsu China; 4 Research Unit of Extracellular RNA, Chinese Academy of Medical Sciences, Jiangsu 210023 Nanjing, China; 5 Institute of Artificial Intelligence Biomedicine, Nanjing University, Jiangsu 210023 Nanjing, China; 6 Pingshan Translational Medicine Center, Shenzhen Bay Laboratory, 518055 Shenzhen, Guangdong China

**Keywords:** Drug delivery, Ulcerative colitis, Gene therapy

## Abstract

Given the complex nature of ulcerative colitis, combination therapy targeting multiple pathogenic genes and pathways of ulcerative colitis may be required. Unfortunately, current therapeutic strategies are usually based on independent chemical compounds or monoclonal antibodies, and the full potential of combination therapy has not yet been realized for the treatment of ulcerative colitis. Here, we develop a synthetic biology strategy that integrates the naturally existing circulating system of small extracellular vesicles with artificial genetic circuits to reprogram the liver of male mice to self-assemble multiple siRNAs into secretory small extracellular vesicles and facilitate in vivo delivery siRNAs through circulating small extracellular vesicles for the combination therapy of mouse models of ulcerative colitis. Particularly, repeated injection of the multi-targeted genetic circuit designed for simultaneous inhibition of TNF-α, B7-1 and integrin α4 rapidly relieves intestinal inflammation and exerts a synergistic therapeutic effect against ulcerative colitis through suppressing the pro-inflammatory cascade in colonic macrophages, inhibiting the costimulatory signal to T cells and blocking T cell homing to sites of inflammation. More importantly, we design an AAV-driven genetic circuit to induce substantial and lasting inhibition of TNF-α, B7-1 and integrin α4 through only a single injection. Overall, this study establishes a feasible combination therapeutic strategy for ulcerative colitis, which may offer an alternative to conventional biological therapies requiring two or more independent compounds or antibodies.

## Abstract (web summary)

Management of ulcerative colitis can require a combination of treatments targeting different pathways. Here the authors design a therapy for ulcerative colitis based on a multitargeted genetic circuit to simultaneously target TNF-α, B7-1 and integrin α4, and show the therapy is effective in male mice with induced or spontaneous genetic colitis.

## Introduction

Ulcerative colitis (UC) is a type of chronic and relapsing inflammatory bowel disease (IBD) that is characterised by uncontrolled intestinal inflammation and disruption of the colonic epithelial layer^1^. Over the past decade, biological therapy with antibodies targeting to the excessive immune responses has been intensively investigated as a therapeutic option for UC patients, especially in cases where the disease is refractory or intolerant to conventional agents^2^. Based on the functions and mechanisms of the targets that are involved in the immune dysregulation of UC, current biological therapy can be classified into several subtypes: (1) Inhibition of pro-inflammatory cytokines or augmentation of the effect of anti-inflammatory cytokines^3^. The most representative one is infliximab, a chimeric monoclonal antibody (mAb) to human tumour necrosis factor-α (TNF-α), which has become the first-line treatment for moderate-to-severe UC^4^. Infliximab promotes anti-inflammatory effects mainly by inhibiting TNF-α in colonic macrophages and suppressing a pro-inflammatory cascade, thereby relieving structural damage to the mucosa^5^. (2) Blockade of T cell homing to sites of inflammation. Upon activation, T cells express integrin α4β7 to recognise mucosal addressin cellular adhesion molecule-1 (MAdCAM-1), which is expressed at sites of inflamed vascular endothelium, thereby directing the migration of effector T cells to the intestinal lamina propria^6^. Natalizumab, an antibody directed against integrin α4, can inhibit T cell migration through the endothelial vascular barrier, therefore showing clinical efficacy for the management of UC^7^. (3) Blockade of T cell activation. Complete T cell activation requires two signals: the first is mediated by the interaction of the T cell receptors (CD4 or CD3) with the antigenic peptides presented by major histocompatibility complex (MHC) molecules on the surface of antigen presenting cells (APCs), and the second is the costimulatory signal, which is provided by the interaction between CD28 protein on the T cell surface and B7 protein (CTLA-4) on the membrane of APCs^8^. Abatacept (a CLTA-4/Fc-fusion protein containing a high-affinity binding site for B7) inhibits the costimulatory signal to T cells, resulting in T cell anergy^9^. Although biological therapy has yielded promising therapeutic outcomes in the preclinical and clinical treatment of UC, it still suffers from some inherent limitations of antibody drugs. The biggest problem is that antibody drugs have strong immunogenicity. A large portion of patients fail to respond to or gradually develop resistance to antibody therapy because of the generation of anti-antibodies^10^. Simultaneously, the high treatment costs and serious side effects remain a major obstacle. Therefore, there is still a considerable unmet medical need to develop a new therapeutic approach for UC^11^.

Because UC is a multifactorial and multistep process, merely blocking a single inflammatory cytokine or immunological target may not be adequate and certainly not optimal to maintain clinical remission and achieve long-term efficacy^12,13^. Recently, combination therapy has been proposed as a promising treatment for a variety of diseases. Theoretically, a combination of two or more drugs given as a single formulation should be more effective than treatment with single or sequentially administered drugs^14^. Unfortunately, it is still technically immature to use combination therapy in UC, especially for patients showing immunological heterogeneity and phenotypic variation. More importantly, traditional combination therapy based on the simultaneous administration of multiple drugs is costly and may involve complicated treatment regimens, undesired drug-drug interactions and an accumulative risk of side effects^13^. Due to the high specificity, potency and flexibility of small interfering RNAs (siRNAs) to control multiple pathogenic genes and pathways at a single dosage, RNA interference (RNAi)-based therapeutics offer an attractive strategy to cotarget multiple pathways involved in the pathogenesis of UC^15^. In principle, the design of a combinatory expression cassette that carries multiple siRNA-expressing units can co-transcribe these siRNAs in cells, thus providing a simple and efficient strategy for inhibiting multiple genes simultaneously. However, siRNAs are susceptible to degradation by ubiquitous ribonucleases (RNases) and, as they are anionic and hydrophilic, are unable to cross the cell membrane efficiently^16^. Thus, the success of RNAi therapy relies heavily on siRNA carriers and delivery approaches. Currently, the development of an appropriate delivery system for siRNAs remains a major bottleneck of RNAi therapy. Recently, some pilot studies on extracellular vesicles (EVs) have provided some inspiration for siRNA delivery^17^. EVs are a heterogeneous group of endogenous, nanosized membrane vesicles that mediate communication between cells by delivering and exchanging proteins, lipids, mRNAs and miRNAs. Since EVs are self-produced by endogenous cells and have the intrinsic ability to transport RNAs in vivo, they emerge as promising delivery carriers for siRNAs^18^. However, it is still very difficult to load sufficient siRNA cargo into EVs and harvest sufficient EVs to reach the scale required by clinical practice. Recently, we developed a synthetic biology strategy that reprogrammes the host liver as a tissue chassis to trigger the self-assembly of siRNAs into secretory small EVs (sEVs, <200 nm) and facilitate the in vivo delivery of siRNAs to desired tissues based on the intrinsic capability of the liver to express transgenes introduced by intravenous injection of genetic circuits (in the form of naked DNA plasmids)^19^. This technology borrows the body’s workshop and reconceptualises the assembly and delivery strategies of siRNA-encapsulating sEVs, thereby avoiding the complicated procedures, high cost and labour intensity associated with current techniques for EV engineering and delivery. Since self-assembled siRNAs hold the potential to simultaneously and synergistically target multiple genes, this technology may provide a promising tool to cotarget multiple genes and pathways in the aetiology of UC, thereby addressing the bottleneck problem in the treatment of UC. In this study, we designed a combinatory multi-targeted genetic circuit and evaluated its therapeutic value in acute and chronic UC models.

## Results

### Construction and characterisation of the genetic circuits targeting TNF-α

**Fig. 1:**
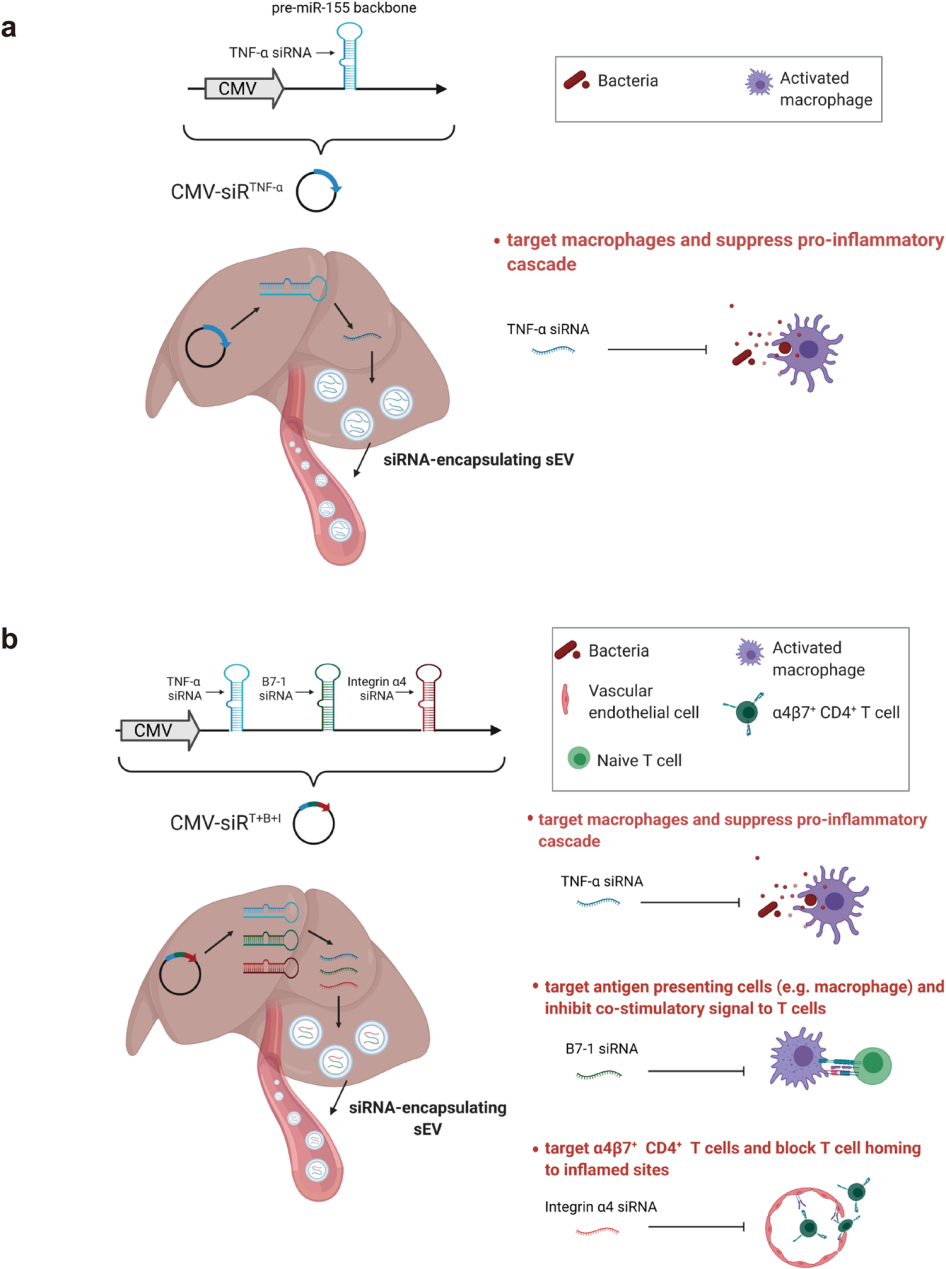
Schematic description of the architecture of the genetic circuits. **a** The CMV-siR^TNF-α^ circuit contains a CMV promoter part and a TNF-α siRNA-expressing part. When the CMV-siR^TNF-α^ circuit is taken up and processed by the liver after intravenous injection, the CMV promoter drives the transcription of TNF-α siRNA in cytoplasm, which leads to the loading of saturated TNF-α siRNA into small extracellular vesicles (sEVs) as cargo. Subsequently, TNF-α siRNA-encapsulating sEVs facilitate the systematic distribution of siRNAs to multiple tissues and cells, including colonic macrophages. Once arriving colonic macrophages, TNF-α siRNA has the potential to regulate immune balance and relieve intestinal inflammation by inhibiting the uncontrolled release of TNF-α from macrophages. **b** The CMV-siR^T+B+I^ circuit contains a CMV promoter part and a multiple siRNA-expressing part carrying three siRNA expression cassettes organised as a head-to-tail tandem array to simultaneously drive the transcription of TNF-α siRNA, B7-1 siRNA and integrin α4 siRNA. B7-1 siRNA is expected to target antigen-presenting cells (APCs) and inhibit the costimulatory signal to T cells. Integrin α4 siRNA is expected to target α4β7^+^ CD4^+^ T cells and block T cell homing to sites of inflammation. Created with BioRender.com.

TNF-α is a key pro-inflammatory cytokine produced primarily by activated macrophages and T lymphocytes, and it plays an integral role in the pathogenesis of UC^20^. TNF-α induces other pro-inflammatory cytokines (e.g., interleukin-1 (IL-1) and IL-6), enhances leucocyte migration by inducing the expression of adhesion molecules and inhibits apoptosis of inflammatory cells^21^. We therefore evaluated the therapeutic effects of the genetic circuit designed to specifically target TNF-α in colonic macrophages for the treatment of UC. We constructed a genetic circuit consisting of two functional modules: the promoter module drives the transcription of siRNA, which leads to the package of saturated cytoplasmic siRNA into sEVs, while the siRNA expression cassette module maximises the expression of the siRNA guide strand and minimises the expression of undesired passenger strand. Based on our previous study^19^, the cytomegalovirus (CMV) promoter was selected as the promoter module, and the pre-miR-155 backbone was selected as the optimal siRNA expression cassette to produce siRNA. By inserting the TNF-α siRNA sequence into the 5′ arm of the pre-miR-155 hairpin, a CMV-directed genetic circuit (in the form of a DNA plasmid) carrying a TNF-α siRNA expression cassette was constructed (hereafter, CMV-siR^TNF-α^) (Fig. 1a).

Next, we tested whether the CMV-siR^TNF-α^ circuit has the ability to synthesise functional TNF-α siRNA in vitro. ANA-1 cells were transfected with three CMV-siR^TNF-α^ circuits designed to target different sites of the coding sequence (CDS) of TNF-α and then treated with LPS to stimulate an inflammatory response. A CMV-directed genetic circuit encoding a scrambled RNA (hereafter, CMV-scrR) was transfected as a negative control. ANA-1 cells exhibited a remarkable increase in TNF-α mRNA expression with LPS stimulation; however, this effect was considerably reduced by transfection with the three CMV-siR^TNF-α^ circuits (Supplementary Fig. 1a). Likewise, while LPS dramatically stimulated the secretion of TNF-α into the cell culture medium, the three CMV-siR^TNF-α^ circuits significantly suppressed the amounts of secreted TNF-α (Supplementary Fig. 1b). The circuit with the greatest interference efficiency, CMV-siR^TNF-α^-1, was selected for further evaluation. In addition, HEK293T cells were used as the cell chassis for the in vitro assembly of TNF-α siRNA. When an increased amount of CMV-siR^TNF-α^-1 circuit was transfected into HEK293T cells, a dose-dependent increase in intracellular TNF-α siRNA was detected (Supplementary Fig. 1c), which was accompanied by a dose-dependent increase in TNF-α siRNA in secreted sEVs (Supplementary Fig. 1d).

### Evaluation of the self-assembly of TNF-α siRNA-encapsulating sEVs

**Fig. 2:**
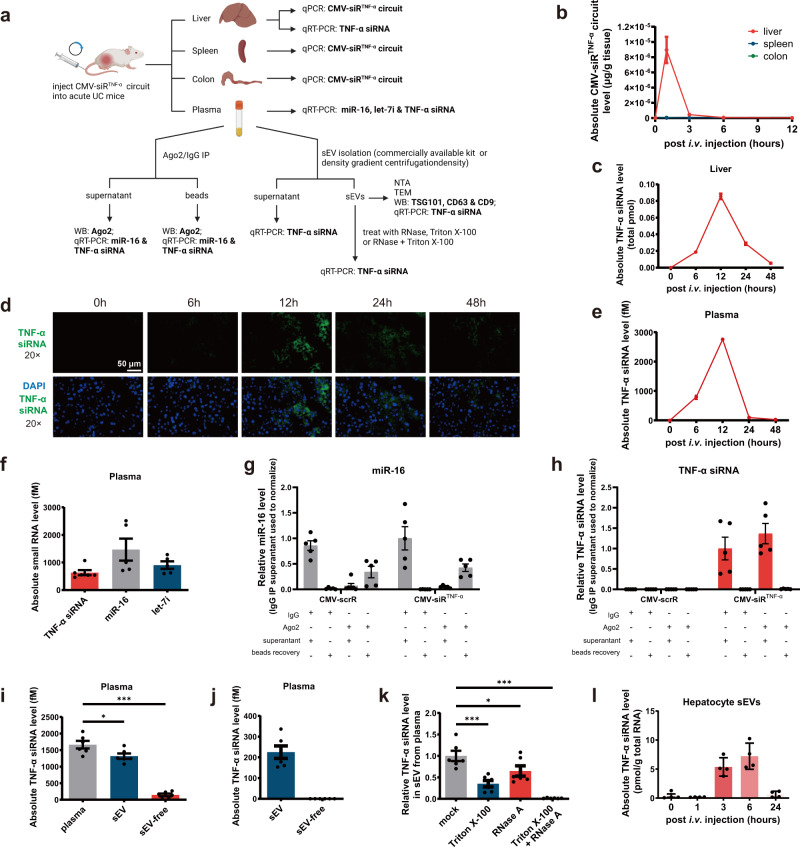
Evaluation of the self-assembly of TNF-α siRNA-encapsulating sEVs in vivo. **a** Schematic of the experimental design. Created with BioRender.com. **b** Kinetics of CMV-siR^TNF-α^ circuit (DNA plasmid) in mouse liver, colon or spleen following tail vein injection of the 5 mg/kg CMV-siR^TNF-α^ circuit (*n* = 3 in each group). **c** Kinetics of TNF-α siRNA in the mouse liver following tail vein injection of the 5 mg/kg CMV-siR^TNF-α^ circuit (*n* = 3 in each group). The total TNF-α siRNA level in the liver was calculated based on the total amount of RNA isolated from one gram of liver and the TNF-α siRNA content in one μg of total RNA. **d** In situ detection of TNF-α siRNA in liver sections of DSS mice at 0, 6, 12, 24 or 48 h after injection with the CMV-siR^TNF-α^ circuit. Positive in situ hybridisation signals are shown in green, and DAPI-stained nuclei are shown in blue. Scale bar: 50 μm. **e** Kinetics of TNF-α siRNA in mouse plasma following tail vein injection of the 5 mg/kg CMV-siR^TNF-α^ circuit (*n* = 3 in each group). **f** Quantitative RT-PCR analysis of the absolute expression levels of TNF-α siRNA, miR-16 and let-7i in mouse plasma at 12 h after injection (*n* = 5 in each group). **g**, **h** Bead-conjugated anti-Ago2 antibody was incubated with the plasma of CMV-scrR circuit- or CMV-siR^TNF-α^ circuit-injected mice under native conditions. The beads were separated by centrifuging, and RNA extracted from the supernatant was subjected to quantitative RT-PCR analysis to determine the amounts of miR-16 and TNF-α siRNA depleted by Ago2 immunoprecipitation. The beads were processed for RNA extraction followed by quantitative RT-PCR analysis to assess the recovery rates of miR-16 and TNF-α siRNA (*n* = 5 in each group). **i** Quantitative RT-PCR analysis of the absolute expression levels of TNF-α siRNA in plasma, sEVs and sEV-free plasma (*n* = 6 in each group). sEVs were purified by using a commercially available kit. **j** Quantitative RT-PCR analysis of the absolute expression levels of TNF-α siRNA in sEVs and sEV-free plasma (*n* = 6 in each group). sEVs were purified by using density gradient centrifugation. **k** Quantitative RT-PCR analysis of the survival percentage of TNF-α siRNA in sEVs treated with RNase A, Triton X-100 or RNase A plus Triton X-100 (*n* = 6 in each group). Untreated sEV sample served as a control. **l** Primary mouse hepatocytes were isolated from mice at different time points following intravenous injection of 5 mg/kg CMV-siR^TNF-α^ circuit. A quantitative RT-PCR assay was performed to assess TNF-α siRNA levels in sEVs derived from the culture medium of primary hepatocytes (*n* = 4 in each group). Values are presented as the mean ± SEM. Significance was determined using one-way ANOVA followed by Dunnett’s multiple comparison. *p < 0.05; ***p < 0.005.

We established several models to examine the self-assembly and secretion of TNF-α siRNA-encapsulating sEVs. An acute UC model was induced in male BALB/c mice by replacing their drinking water with a 2.5% DSS solution for 7 days; the CMV-scrR or CMV-siR^TNF-α^ circuit (5 mg/kg) was intravenously injected into DSS mice, and then the amounts of genetic circuits (plasmids) that were taken up by mouse liver and the amounts of siRNAs that were packaged into mouse plasma sEVs were specifically examined (Fig. 2a). First, we determined the amounts of the genetic circuits (plasmids) that were taken up by the liver and the resultant siRNAs that were generated in the liver. The CMV-siR^TNF-α^ circuit rapidly accumulated in the livers at 1 h then decreased to background levels at 6 h; no CMV-siR^TNF-α^ circuit was detected in other tissues of CMV-siR^TNF-α^-injected mice (Fig. 2b). Likewise, a time-dependent accumulation and clearance of TNF-α siRNA were observed in the livers of CMV-siR^TNF-α^-injected mice (peaking at 12 h and decreasing to a background level at 48 h) (Fig. 2c). Similarly, direct tracking of TNF-α siRNA in mouse liver by fluorescence in situ hybridisation (FISH) also revealed a time-dependent change in TNF-α siRNA levels in the liver (Fig. 2d and Supplementary Fig. 2a). These results are consistent with the findings of previous studies and support the idea that the liver can express transgenes introduced by intravenously injected genetic circuits (naked DNA plasmids)^22^.

Subsequently, we investigated whether the CMV-siR^TNF-α^ circuit could trigger efficient production and secretion of TNF-α siRNA into blood circulation. A time-dependent accumulation of TNF-α siRNA was observed in the plasma-derived from mice injected with the CMV-siR^TNF-α^ circuit (peaking at 12 h and decreasing to a background level at 48 h) (Fig. 2e). Meanwhile, TNF-α siRNA was present in mouse plasma in a similar concentration range as endogenous miR-16 and let-7i (Fig. 2f), which are two of the most abundant circulating miRNAs in the blood.

Previous studies have raised two possible mechanisms for the stability of circulating miRNAs despite the presence of ubiquitous RNases in the blood: (1) protection of miRNAs by the membrane structures of EVs^23–25^; and (2) stabilisation of miRNAs by their association with RNA-binding proteins, such as Argonaute-2 (Ago2)^26,27^. We investigated which mechanism provides a protected and controlled internal microenvironment for TNF-α siRNA, allowing it to travel in the blood without degradation by RNases: (1) to determine whether Ago2 conjunction may protect TNF-α siRNA from degradation by RNases in the plasma, the amounts of TNF-α siRNA remaining in the supernatant after Ago2 immunoprecipitation and the amounts recovered from the Ago2-immunoprecipitated beads were measured (Fig. 2a). The plasma of CMV-scrR circuit- or CMV-siR^TNF-α^ circuit-injected DSS mice was immunoprecipitated using control IgG or anti-Ago2 antibody, and the Ago2 and IgG immunoprecipitates were assayed for miR-16 and TNF-α siRNA to determine if the TNF-α siRNA is associated with Ago2 protein in blood circulation. Western blot analysis showed a specific Ago2 band at ∼97 kDa in the Ago2 immunoprecipitates, while no Ago2 was detected in IgG immunoprecipitates and in the supernatant immunoprecipitated with anti-Ago2 antibody (Supplementary Fig. 3), demonstrating that Ago2 was specifically precipitated from plasma. Quantitative RT-PCR analysis revealed that miR-16 was largely depleted from plasma by Ago2 immunoprecipitation compared with control IgG immunoprecipitation and that the depleted miR-16 could be efficiently recovered from the immunoprecipitated beads (Fig. 2g). In contrast, no TNF-α siRNA seemed to be immunoprecipitated from the plasma by anti-Ago2 antibody, and Ago2 immunoprecipitates were almost totally free of TNF-α siRNA (Fig. 2h). Thus, unlike the miR-16 that is predominantly associated with Ago2 protein in blood plasma, TNF-α siRNA may be protected by a mechanism other than conjunction with Ago2 protein. (2) Since miR-155 has been identified to be associated predominantly with sEV-rich fraction in the plasma^28–31^, we investigated whether TNF-α siRNA, generated through the pre-miR-155 backbone, might be released to the blood in an sEV-dependent manner (Fig. 2a). We characterised the physical properties of sEVs purified from the plasma of mice injected with the CMV-scrR or CMV-siR^TNF-α^ circuit. Nanoparticle tracking analysis (NTA) revealed that the plasma sEVs purified from circuit-injected mice using a commercially available kit had the typical size of nanoparticles (peaked at ~120 nm) and were present at a concentration of ~3.5 × 10^7^ particles/mL, similar to wild-type normal sEVs (Supplementary Fig. 4a). Transmission electron microscopy (TEM) showed that the purified sEVs exhibited a characteristic round vesicular morphology (Supplementary Fig. 4b). Enrichment of sEV markers (TSG101, CD63 and CD9) was detected only in purified sEVs but not in sEV-free plasma (Supplementary Fig. 4c). Density gradient centrifugation is the gold-standard method for achieving sEVs with the highest purity^32^. Therefore, we purified sEVs from the plasma of mice using density gradient centrifugation and characterised their particle size and morphology using NTA and TEM. The plasma sEVs purified using density gradient centrifugation exhibited typical sEV morphology and size (peaked at ~120 nm) and a similar concentration (~4 × 10^6^ particles/mL) in different groups (Supplementary Fig. 4d and 4e). However, the sEVs from density gradient centrifugation were typically lower than the yield from the commercially available kit. These results reveal that injection of genetic circuits does not affect the size, structure or number of sEVs generated in vivo. Notably, most TNF-α siRNA was detected in the plasma sEVs rather than the sEV-free plasma of CMV-siR^TNF-α^-injected mice, regardless of whether the plasma sEVs were purified using a commercially available kit or density gradient centrifugation (Fig. 2i, j). (3) To verify that TNF-α siRNA is indeed present inside the sEVs and protected by the lipid bilayer structure of sEVs, the sensitivity of TNF-α siRNA to RNase was determined in presence or absence of a non-ionic detergent (Triton X-100) (Fig. 2a). Plasma sEVs derived from CMV-siR^TNF-α^-injected mice were divided into four equal portions and were untreated or treated with RNase, Triton X-100 or RNase plus Triton X-100 for 30 min. The retained TNF-α siRNA in each sample was examined. TNF-α siRNA levels in the sEVs treated with RNase were little changed compared to untreated sEVs, and TNF-α siRNA levels in the sEVs treated with RNase and Triton X-100 were completely eliminated (Fig. 2k), which suggests that TNF-α siRNA was present within the sEVs and not simply released as a contaminant during the sEV purification process. Overall, these results suggested that TNF-α siRNA is present in mouse plasma in a form (in sEVs) that can be easily delivered to other cells, similar to those bioactive circulating miRNAs (e.g., miR-155).

To further confirm that hepatocytes were the original site for the self-assembly and secretion of TNF-α siRNA-encapsulating sEVs, we isolated primary mouse hepatocytes at different time points following the injection of the CMV-siR^TNF-α^ circuit into mice and assessed TNF-α siRNA levels in sEVs derived from the culture medium of primary hepatocytes. TNF-α siRNA levels were higher in the sEVs of primary hepatocytes isolated from CMV-siR^TNF-α^ circuit-injected mice at 6 h and were diminished in the sEVs of primary hepatocytes isolated from CMV-siR^TNF-α^ circuit-injected mice at 24 h (Fig. 2l). These results indicate that the secretion of TNF-α siRNA-encapsulating sEVs increased following the injection of CMV-siR^TNF-α^ circuit, reached a peak after 6 h then declined to background at 24 h in the ex vivo model.

### Evaluation of the activity of TNF-α siRNA-encapsulating sEVs in an ex vivo model

**Fig. 3:**
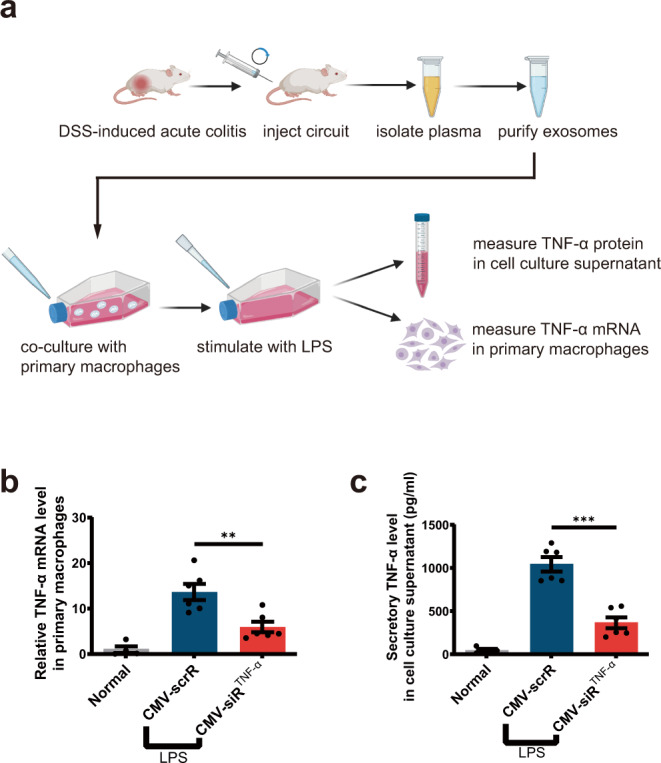
Evaluation of the activity of TNF-α siRNA-encapsulating sEVs in an ex vivo model. **a** Schematic of the experimental design. Acute UC was induced in male BALB/c mice by replacing their drinking water with a 2.5% DSS solution for 7 days. DSS mice were intravenously injected with 5 mg/kg CMV-scrR or CMV-siR^TNF-α^ circuit every day for a total of 7 times, and then the sEVs were purified from the plasma of each mouse and dissolved in 50 µL PBS. BCA method was employed to quantify total protein content in sEVs, and the isolated sEVs had a total protein concentration of ~0.8 μg/μL. Subsequently, the sEV solution (50 µL) was incubated with 1 × 10^5^ primary macrophages. After stimulating macrophages with 50 ng/mL LPS, the suppression of TNF-α expression by TNF-α siRNA were examined in this ex vivo model. **b** Quantitative RT-PCR analysis of the relative expression levels of TNF-α mRNA in primary macrophages (*n* = 6 in each group). Created with BioRender.com. **c** Determination of the levels of secretory TNF-α protein in cell culture supernatant by ELISA (*n* = 6 in each group). Values are presented as the mean ± SEM. Significance was determined using one-way ANOVA followed by Dunnett’s multiple comparison. **p < 0.01; ***p < 0.005.

We evaluated whether the formation of TNF-α siRNA-encapsulating plasma sEVs could reduce TNF-α expression in an ex vivo model (Fig. 3a). When macrophages were incubated with plasma sEVs derived from DSS mice injected with the CMV-siR^TNF-α^ circuit and then stimulated with LPS, a significant decrease in TNF-α mRNA expression was observed (Fig. 3b), which was accompanied by a reduced amount of TNF-α secreted into the cell culture medium (Fig. 3c). These results indicated that the CMV-siR^TNF-α^ circuit could trigger the self-assembly of TNF-α siRNA into plasma sEVs in vivo, which could be further internalised by recipient cells to block TNF-α expression.

### Tracking of the delivery of self-assembled TNF-α siRNA to immune cells in inflamed mucosa

**Fig. 4:**
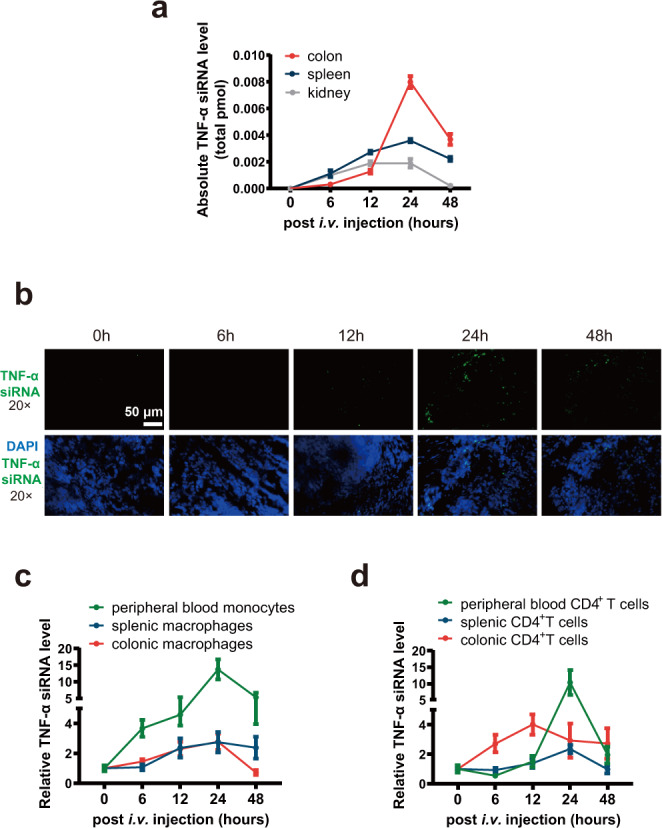
Tracking of the delivery of self-assembled TNF-α siRNA to immune cells in inflamed mucosa. **a** Kinetics of TNF-α siRNA in the mouse colon, spleen and kidney following tail vein injection of the 5 mg/kg CMV-siR^TNF-α^ circuit (*n* = 3 in each group). The total TNF-α siRNA levels in the colon, spleen and kidney were calculated based on the total amount of RNA isolated from one gram of each tissue and the TNF-α siRNA content in one μg of total RNA. **b** In situ detection of TNF-α siRNA in colon sections of DSS mice at 0, 6, 12, 24 or 48 h after injection with the CMV-siR^TNF-α^ circuit. Positive in situ hybridisation signals are shown in green, and DAPI-stained nuclei are shown in blue. Scale bar: 50 μm. **c** Kinetics of TNF-α siRNA in monocytes and macrophages derived from peripheral blood, spleen and colonic lamina propria of DSS mice following tail vein injection of the 5 mg/kg CMV-siR^TNF-α^ circuit (*n* = 3 in each group). **d** Kinetics of TNF-α siRNA in CD4^+^ T cells derived from peripheral blood, spleen and colonic lamina propria of DSS mice following tail vein injection of the 5 mg/kg CMV-siR^TNF-α^ circuit (*n* = 3 in each group).

We investigated whether sEV-enclosed TNF-α siRNA was successfully delivered to mononuclear phagocytes, particularly to colonic macrophages, via the circulating system of sEVs. The CMV-siR^TNF-α^ circuit was intravenously injected into DSS-induced acute UC mice, and then the biodistribution of TNF-α siRNA in various tissues was determined. A time-dependent accumulation of TNF-α siRNA was observed in the colon, spleen and kidney of CMV-siR^TNF-α^-injected mice (peaking at 12-24 h and decreasing significantly after 48 h) (Fig. 4a). Likewise, when the distribution of TNF-α siRNA to the colon was examined by FISH, hybridisation signals of TNF-α siRNA were detected, with a similar time trend, in the colon sections of CMV-siR^TNF-α^-injected mice (Fig. 4b and Supplementary Fig. 2b). Since TNF-α is produced primarily by activated macrophages and T lymphocytes^20^, we specifically isolated macrophages, monocytes (blood monocytes are essential for continually replenishing the resident macrophages in the gut wall) and CD4^+^ T cells from the colonic lamina propria, peripheral blood and spleen of CMV-siR^TNF-α^-treated mice to quantify the cellular uptake of TNF-α siRNA. A time-dependent increase in TNF-α siRNA levels was detected in these isolated immune cells (Fig. 4c, d). Thus, TNF-α siRNA-encapsulating sEVs possessed the capacity to access immune cells in inflamed mucosa, blood and spleen, resulting in efficient delivery of TNF-α siRNA to the sites of desire.

### In vivo therapeutic efficacy of self-assembled TNF-α siRNA in an acute UC model

**Fig. 5:**
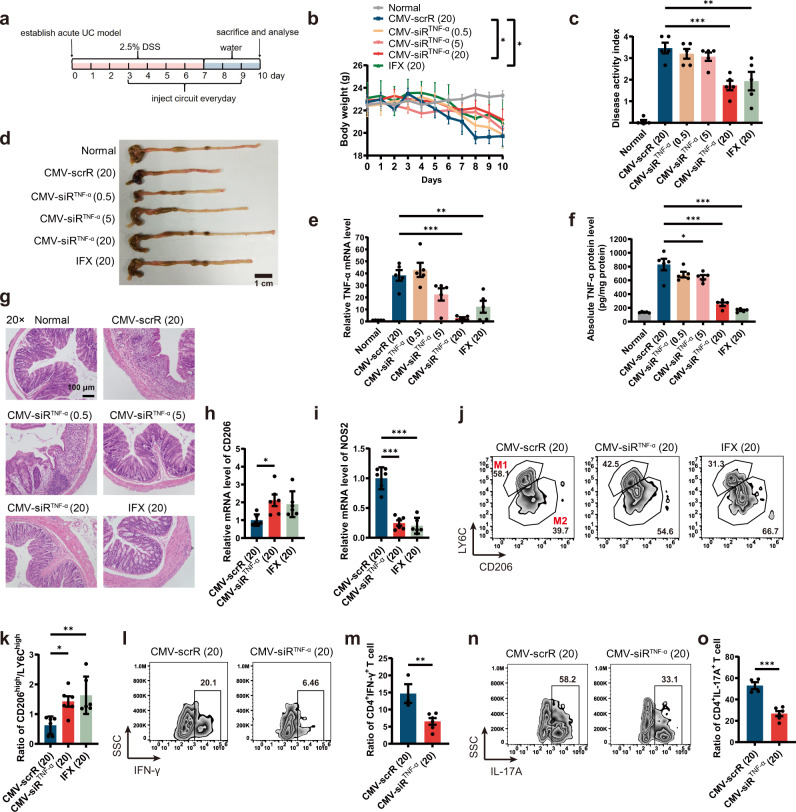
Intravenous injection of the CMV-siR^TNF-α^ circuit protects mice from DSS-induced acute UC. **a** Flow chart of the experimental design. **b** Body weight curves (*n* = 5 in each group). **c** Digital Acceleration Index (DAI) scores (*n* = 5 in each group). **d** Representative macroscopic features of colons. Scale bar: 1 cm. **e** Quantitative RT-PCR analysis of the relative expression levels of TNF-α mRNA in the colon (*n* = 5 in each group). **f** Determination of the absolute expression levels of TNF-α protein in the colon by ELISA (*n* = 5 in each group). **g** Representative images of H&E staining of colon sections. Scale bar: 100 μm. Each experiment was repeated independently three times, and representative results are shown. **h**, **i** Quantitative RT-PCR analysis of the relative expression levels of CD206 and NOS2 mRNA in the colon (*n* = 6 in each group). **j** Representative flow cytometric characterisation of colonic macrophages (CD11b^+^CD64^+^) subdivided into M1-like (Ly6C^high^CD206^low^) and M2-like (Ly6C^low^CD206^high^) macrophages. **k** Bar graph depicting the ratio between M2-like versus M1-like macrophages (*n* = 5 in each group). **l**, **n** Lamina propria mononuclear cells (LPMCs) were stimulated with PMA/Ionomycin mixture and BFA/Monensin mixture for 5 h, and the percentage of CD4^+^IFN-γ^+^ cells and CD4^+^IL-17A^+^ cells in CD4^+^ T cells was measured by flow cytometry. **m**, **o** Bar graph depicting the percentage of CD4^+^IFN-γ^+^ T cells (*n* = 3 in CMV-scrR group; *n* = 6 in CMV-siR^TNF-α^ group) and CD4^+^IL-17A^+^ T cells (*n* = 4 in CMV-scrR group; *n* = 6 in CMV-siR^TNF-α^ group). Values are presented as the mean ± SEM. Significance was determined using one-way ANOVA followed by Dunnett’s multiple comparison in (**c**, **e**, **f**, **h**, **i**, **k**, **m**, **o**) or using two-way ANOVA followed by Dunnett’s multiple comparison in panel b. *p < 0.05; **p < 0.01; ***p < 0.005.

Next, we evaluated the therapeutic effects of self-assembled TNF-α siRNA on mice suffering from acute UC. An acute UC model was induced in male BALB/c mice by replacing their drinking water with a 2.5% DSS solution for 7 days. Starting on day 3, DSS mice were intravenously injected with 20 mg/kg CMV-scrR or three dosages (0.5, 5 and 20 mg/kg) of the CMV-siR^TNF-α^ circuit or 20 mg/kg infliximab (IFX) once a day for seven times until the final analysis on day 10 (Fig. 5a). Untreated BALB/c mice were included as normal controls. CMV-scrR circuit-treated DSS mice experienced apparent body weight loss compared with normal control mice; however, treatment with a high dose of the CMV-siR^TNF-α^ circuit significantly alleviated body weight loss in DSS mice (Fig. 5b). The disease activity index (DAI), a composite score reflecting the severity of the disease characteristics, was dramatically elevated in CMV-scrR circuit-treated DSS mice, but this score was significantly alleviated by treatment with a high dose of the CMV-siR^TNF-α^ circuit (Fig. 5c). Furthermore, CMV-scrR circuit-treated DSS mice had distinctly shorter colon lengths than normal mice, but treatment with a high dose of the CMV-siR^TNF-α^ circuit caused a significant recovery of colon length (Fig. 5d and Supplementary Fig. 5a). In terms of the expression levels of TNF-α, while a remarkable increase in colonic TNF-α mRNA and protein levels was observed in the DSS mice treated with the CMV-scrR circuit, a dose-dependent reduction in TNF-α mRNA and protein levels was observed in the colon of DSS mice injected with the CMV-siR^TNF-α^ circuit (Fig. 5e, f). Immunofluorescence staining of TNF-α also confirmed a dose-dependent decline in TNF-α protein levels in the colonic lamina propria derived from CMV-siR^TNF-α^ circuit-treated mice (Supplementary Fig. 5b). Accordingly, while the production of pro-inflammatory cytokines (IL-6, IL-12p70 and IL-23) that are mechanistically associated with TNF-α was evoked in the colon of CMV-scrR circuit-treated DSS mice, all of these cytokines were markedly reduced by a high dose of the CMV-siR^TNF-α^ circuit (Supplementary Fig. 5c). Likewise, myeloperoxidase (MPO) activity, a direct indicator of the infiltration of neutrophils into colonic mucosa, was alleviated by a high dose of the CMV-siR^TNF-α^ circuit (Supplementary Fig. 5d). Finally, many cardinal histological signs of UC, including mucosal damage, ulceration, neutrophil infiltration, crypt abscesses and muscular layer thickness, were clearly manifested in the colonic sections derived from CMV-scrR circuit-treated mice, but these signs were significantly improved after treatment with the CMV-siR^TNF-α^ circuit, and the colonic sections derived from CMV-siR^TNF-α^ circuit-treated mice had relatively intact epithelia, well-defined crypt structures and relatively low levels of neutrophil infiltration (Fig. 5g and Supplementary Fig. 5e). Accordingly, the colonic histological score was higher in CMV-scrR circuit-treated mice, but this score was dose-dependently reduced by treatment with the CMV-siR^TNF-α^ circuit (Supplementary Fig. 5f). Overall, these results demonstrated that the self-assembled TNF-α siRNA induced by the CMV-siR^TNF-α^ circuit could alleviate the characteristics of DSS-induced inflammation and achieve mucosal healing. Conversely, infliximab also improved many critical signs of UC in DSS mice, but the therapeutic effect was somehow inferior to that of the CMV-siR^TNF-α^ circuit (Fig. 5b–g and Supplementary Fig. 5a–f). Similarly, in an acute colitis model established by trinitrobenzene sulfonic acid (TNBS), the characteristic symptoms of colitis were significantly alleviated by treatment with the CMV-siR^TNF-α^ circuit, to a similar, if not better, extent as treatment with infliximab (Supplementary Fig. 6).

At the cellular level, the functional alterations of macrophages in addition to reducing the production of TNF-α were evaluated. Macrophages are highly plastic cells that polarise into different phenotypes (pro-inflammatory M1 macrophages *vs*. immunosuppressive M2 macrophages) in response to surrounding stimuli^33^. Macrophages in the colon of UC are recruited and polarised locally into the M1 phenotype to drive further inflammation^34^. CMV-siR^TNF-α^ circuit-treated mice exhibited increased expression of CD206 (a marker of M2 macrophages) and reduced expression of NOS2 (a marker of M1 macrophages) in the colon compared to the control mice treated with the CMV-scrR circuit (Fig. 5h, i). The increased level of CD206 and the depressed level of NOS2 in the chronic UC model indicated a shift in intestinal macrophage balance towards the anti-inflammatory M2 phenotype. Macrophages were specifically isolated and characterised using flow cytometry to determine the expression of Ly6C and CD206, which differentiate between pro-inflammatory and immunosuppressive subsets of macrophages. A significantly higher rate of macrophages derived from the CMV-siR^TNF-α^ circuit-treated mice were identified as Ly6C^low^CD206^high^ macrophages compared to CMV-scrR circuit-treated control mice (Fig. 5j, k), which is consistent with an anti-inflammatory M2 phenotype. Infliximab also increased M2 macrophages and decreased M1 macrophages in the inflamed colon of the chronic UC model, and the therapeutic efficacy was equivalent to the CMV-siR^TNF-α^ circuit (Fig. 5h–k). Overall, these results demonstrate that in vivo self-assembled TNF-α siRNA actively regulates the differentiation and polarisation of macrophages in the inflamed colon and modulates the balance between M1 and M2 macrophages to prevent intestinal inflammation.

Because TNF-α is also produced from CD4^+^ T cells, we investigated whether CMV-siR^TNF-α^ treatment affected functional changes of CD4^+^ T cells in inflamed mucosa. IBD is classically associated with gut accumulation of pro-inflammatory T-helper 1 (Th1) and T-helper 17 (Th17) cells and an insufficient presence of regulatory T cells (Tregs) to suppress inflammation^35,36^. Analysis of the canonical Th1 cytokine IFN-γ and the Th17 cytokines IL-17A and IL-22 using ELISA revealed that treatment with the CMV-siR^TNF-α^ circuit significantly reduced the levels of IFN-γ, IL-17A and IL-22 in the colon of DSS mice; in contrast, the levels of IL-10 and transforming growth factor-β1 (TGF-β1), two main Treg cytokines that suppress the inflammatory response, were increased by treatment with the CMV-siR^TNF-α^ circuit (Supplementary Fig. 5g–k). We also analysed the phenotypes of CD4^+^ T cells using flow cytometry and found that CMV-siR^TNF-α^ circuit treatment resulted in a significantly reduced percentage of IFN-γ^+^CD4^+^ T cells and IL-17A^+^CD4^+^ T cells in the colonic lamina propria of DSS mice (Fig. 5l–o). These results indicate that in vivo self-assembled TNF-α siRNA restores the balance of Th1/Th17/Treg subsets and protects against intestinal inflammation in the UC model.

### In vivo therapeutic efficacy of self-assembled TNF-α siRNA in a chronic UC model

**Fig. 6:**
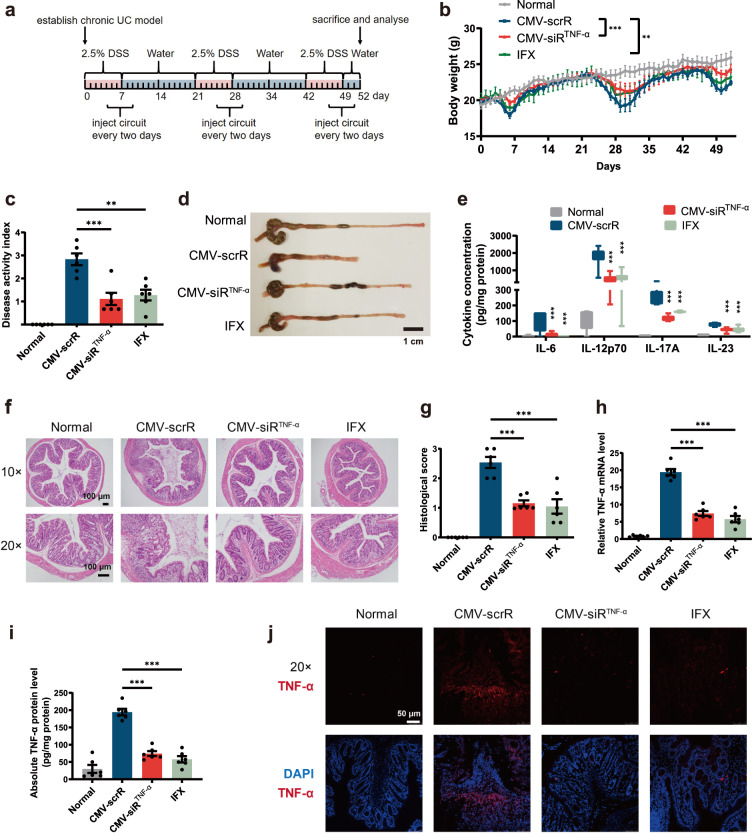
Intravenous injection of the CMV-siR^TNF-α^ circuit protects mice from DSS-induced chronic UC. **a** Flow chart of the experimental design. **b** Body weight curves (*n* = 6 in each group). **c** DAI scores (*n* = 6 in each group). **d** Representative macroscopic features of colons. Scale bar: 1 cm. **e** Determination of serum levels of IL-6, IL-12p70, IL-17A and IL-23 by ELISA (*n* = 6 in each group). **f** Representative images of H&E staining of colon sections. Scale bar: 100 μm. **g** Histological scores of colon sections (*n* = 6 in each group). **h** Quantitative RT-PCR analysis of the relative expression levels of TNF-α mRNA in the colon (*n* = 6 in each group). **i** Determination of the absolute expression levels of TNF-α protein in the colon by ELISA (*n* = 6 in each group). **j** Immunofluorescence staining of TNF-α (red) and DAPI (blue) in colon sections. Scale bar: 50 μm. Each immunofluorescence staining was repeated independently three times, and representative images are shown. Values are presented as the mean ± SEM. Significance was determined using one-way ANOVA followed by Dunnett’s multiple comparison in (**c**, **e**, **g**, **h**, **I**) or two-way ANOVA followed by Dunnett’s multiple comparison in (**b**). *p < 0.05; **p < 0.01; ***p < 0.005.

Because UC is a chronic condition characterised by recurrent episodes of intestinal inflammation, we established a DSS-induced chronic UC model. Chronic UC was induced in male BALB/c mice by rhythmically administering to mice 2.5% DSS for 1 week and water for 2 weeks for 3 cycles. Four days after each DSS drinking, mice were intravenously injected with 20 mg/kg CMV-scrR, 20 mg/kg CMV-siR^TNF-α^ or 20 mg/kg infliximab for a total of 3 times, once every 2 days (Fig. 6a). On day 52, the mice were euthanized, and symptoms and histology were evaluated. Untreated BALB/c mice were included as normal controls. Similar to the therapeutic efficacy acquired in acute model, CMV-siR^TNF-α^ circuit-treated mice experienced milder symptoms of UC, including lower body weight loss, a decreased DAI score, a longer colon length, reduced colonic pro-inflammatory cytokines, reduced MPO activity and an improved histological appearance of colonic sections, compared with the control mice treated with the CMV-scrR circuit (Fig. 6b–g and Supplementary Fig. 7a, b). At the molecular level, treatment with the CMV-siR^TNF-α^ circuit significantly reduced the expression levels of TNF-α mRNA and protein in the colon of the chronic UC model (Fig. 6h–j and Supplementary Fig. 7c). The CMV-siR^TNF-α^ circuit performed slightly better than infliximab in ameliorating the clinical and histopathological severity of UC in the chronic model (Fig. 6b–j and Supplementary Fig. 7). Furthermore, the potential side effects and tissue toxicity of the genetic circuits were evaluated. Repeated injection of the CMV-siR^TNF-α^ circuit posed negligible hepatic toxicity and renal toxicity and caused no overt tissue damage in the chronic UC model (Supplementary Fig. 8a). Likewise, representative serum biochemical indexes, including alanine aminotransferase (ALT), aspartate aminotransferase (AST), total bilirubin (TBIL), blood urea nitrogen (BUN), alkaline phosphatase (ALP) and creatinine (CREA), were unchanged after treatment with the CMV-siR^TNF-α^ circuit (Supplementary Fig. 8b–g). In addition, the anti-TNF-α efficacy of the CMV-siR^TNF-α^ circuit was validated in a TNBS-induced chronic colitis model (Supplementary Fig. 9). Overall, these results demonstrate the therapeutic potential of the self-assembled TNF-α siRNA to inhibit the progression of intestinal inflammation and to promote the recovery of colon tissue from UC.

### Design of a multi-targeted genetic circuit to simultaneously assemble multiple siRNAs for combination therapy of UC

Subsequently, we designed a combinatory multi-targeted genetic circuit to simultaneously block multiple causal genes and pathways relevant to the onset and persistence of UC. Based on literature mining^37–41^, we first set up a group of candidate target genes, including pro-inflammatory cytokines (IL-17A, IFN-γ and IL-6)^37–40^, adhesion molecules involved in T cell trafficking to the gut (integrin α4 and ICAM-1)^41,42^ and molecules essential for T cell activation (CD3 and B7-1)^8,9^. Second, we constructed a library of genetic circuits among which each genetic circuit carried only one siRNA expression cassette directed against one of the candidate target genes. After assessing the therapeutic efficacy of these genetic circuits individually, genetic circuits targeting B7-1, integrin α4 and ICAM-1 were shown to be more effective than circuits targeting IL-17A, IFN-γ, IL-6 and CD3 in ameliorating the manifestations of DSS-induced UC, as assessed by the DAI score and colon length (Supplementary Fig. 10a–c). Moreover, genetic circuits targeting B7-1 and integrin α4 were more efficient than circuits targeting IL-17A, IFN-γ, IL-6, CD3 and ICAM-1 in knocking down their target genes (Supplementary Fig. 10d–j). To view the effects as a whole, genetic circuits targeting B7-1 and integrin α4 were retained. Third, we constructed a multi-targeted genetic circuit carrying three siRNA expression cassettes, which were organised into a head-to-tail tandem array under the control of a CMV promoter to simultaneously silence TNF-α, B7-1 and integrin α4 (hereafter, CMV-siR^T+B+I^) (Fig. 1b). As a basis of comparison, the CMV-siR^TNF-α^, CMV-siR^B7-1^ and CMV-siR^Integrin α4^ circuits carrying a single siRNA expression cassette against their corresponding target genes were included as controls.

To validate that intravenous injection of the CMV-siR^T+B+I^ circuit indeed induced the co-assembly of three siRNAs into plasma sEVs and facilitated the delivery of three siRNAs to target cells, we assessed the in vivo distribution of the three siRNAs in a DSS-induced UC model. Regardless of injection with individual CMV-siR^TNF-α^ circuit or multi-targeted CMV-siR^T+B+I^ circuit, a similar amount of TNF-α siRNA was detected in the plasma and colon of DSS mice (Supplementary Fig. 11a, b). Direct tracking of TNF-α siRNA by FISH also revealed the clear presence of TNF-α siRNA in the colonic macrophages derived from the mice injected with the CMV-siR^TNF-α^ or CMV-siR^T+B+I^ circuit (Supplementary Fig. 11c). Simultaneously, a similar amount of B7-1 siRNA accumulated in the plasma, colon and colonic macrophages derived from the mice treated with individual CMV-siR^B7-1^ circuit or multi-targeted CMV-siR^T+B+I^ circuit (Supplementary Fig. 11d–f). Moreover, compared with the DSS mice treated with the CMV-scrR circuit, extensive accumulation of integrin α4 siRNA was detected in the plasma, colon and colonic CD4^+^ T cells of DSS mice treated with the CMV-siR^Integrin α4^ or CMV-siR^T+B+I^ circuit (Supplementary Fig. 11g–i).

**Fig. 7:**
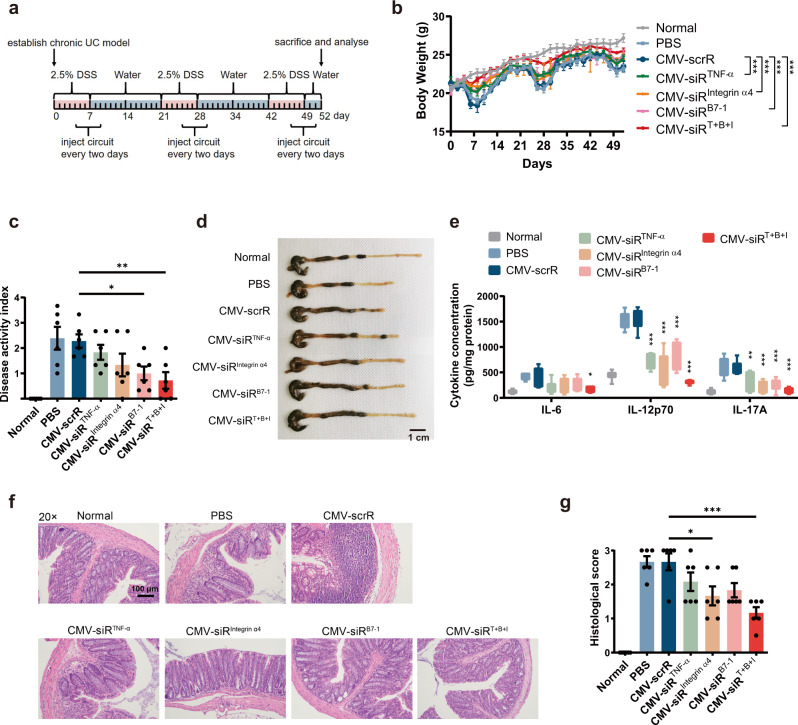
Intravenous injection of the multi-targeted CMV-siR^T+B+I^ circuit exerts a synergistic therapeutic effect against DSS-induced chronic UC. **a** Flow chart of the experimental design. **b** Body weight curves (*n* = 6 in each group). **c** DAI scores (*n* = 6 in each group). **d** Representative macroscopic features of colons. Scale bar: 1 cm. **e** Determination of serum levels of IL-6, IL-12p70 and IL-17A by ELISA (*n* = 6 in each group). **f** Representative images of H&E staining of colon sections. Scale bar: 100 μm. **g** Histological scores of colon sections (*n* = 6 in each group). Values are presented as the mean ± SEM. Significance was determined using one-way ANOVA followed by Dunnett’s multiple comparison in (**c**, **e,** **g**) or two-way ANOVA followed by Dunnett’s multiple comparison in (**b**). *p < 0.05; **p < 0.01; ***p < 0.005.

Next, the therapeutic effects of the multi-targeted CMV-siR^T+B+I^ circuit were evaluated in a DSS-induced chronic UC model to investigate whether an additive advantage was achieved. Chronic UC was induced in male BALB/c mice by rhythmically administering to mice 2.5% DSS for 1 week and water for 2 weeks and the cycle was repeated for 3 times. Four days after each DSS drinking, mice were intravenously injected with PBS or equal dose (10 mg/kg) of CMV-scrR, CMV-siR^TNF-α^, CMV-siR^Integrin α4^, CMV-siR^B7-1^ or CMV-siR^T+B+I^ circuit for a total of 3 times, once every 2 days (Fig. 7a). On day 52, the mice were euthanised, and symptoms and histology were evaluated. Untreated BALB/c mice were included as normal controls. While both multi- and single-targeted genetic circuits significantly alleviated body weight loss, promoted the recovery of colon length, reduced the DAI score and improved the histological appearance in the DSS-induced UC model, the CMV-siR^T+B+I^-treated group showed the least body weight loss, best colon length recovery, lowest DAI score and inflammatory cytokine levels, and minimal histological signs of UC among all treatment groups (Fig. 7b–g and Supplementary Fig. 12). In terms of body weight, colon length and inflammatory cytokine levels, treatment with the CMV-siR^T+B+I^ circuit even alleviated these pathophysiological parameters to a similar level in normal control mice (Fig. 7b–e and Supplementary Fig. 12a). In particular, colon tissues from the CMV-siR^T+B+I^-treated group exhibited almost the same tissue morphology as that observed in the normal control group, especially with respect to the integration of the colonic epithelial layer and the infiltration of inflammatory cells (Fig. 7f, g and Supplementary Fig. 12b). Moreover, hepatic and renal toxicity and abnormal alterations in serum biochemical indexes were not observed after injection with the multi-targeted CMV-siR^T+B+I^ circuit (Supplementary Fig. 8). Overall, combination therapy with the multi-targeted CMV-siR^T+B+I^ circuit yielded the highest therapeutic efficacy among all the tested groups, indicating that the multi-targeted genetic circuit could exert a synergistic therapeutic effect against DSS-induced UC.

**Fig. 8:**
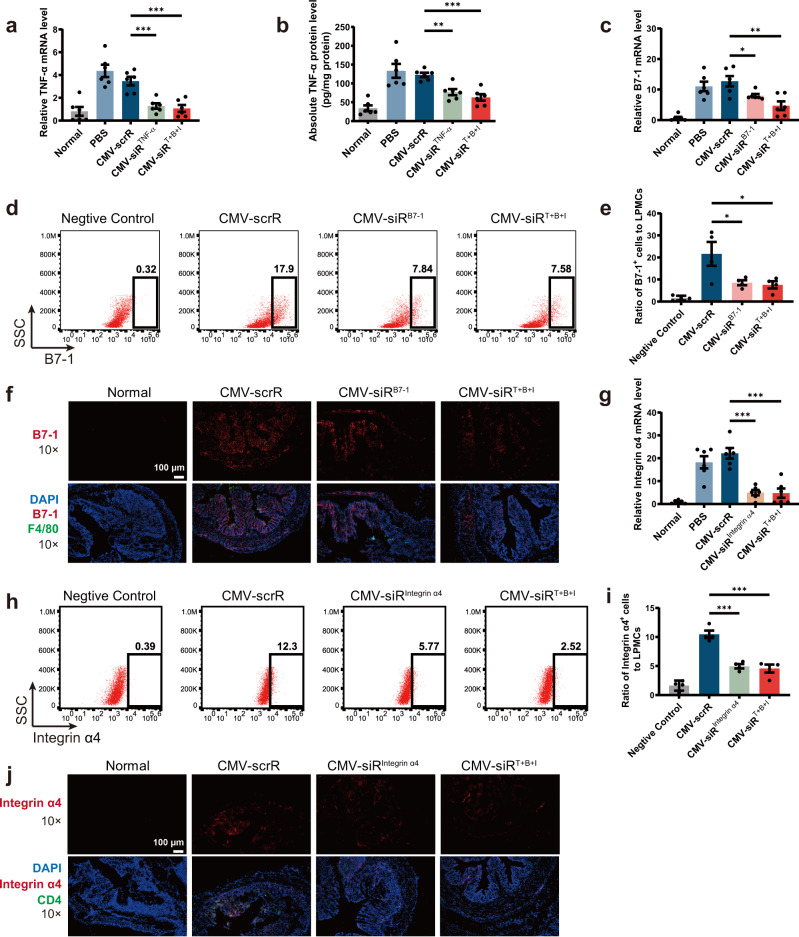
Intravenous injection of the multi-targeted CMV-siR^T+B+I^ circuit simultaneously inhibits TNF-α, B7-1 and integrin α4 in DSS-induced chronic UC. **a** Quantitative RT-PCR analysis of the relative expression levels of TNF-α mRNA in the colon (*n* = 6 in each group). **b** Determination of the absolute expression levels of TNF-α protein in the colon by ELISA (*n* = 6 in each group). **c** Quantitative RT-PCR analysis of the relative expression levels of B7-1 mRNA in the colon (*n* = 6 in each group). **d** Representative flow cytometric plots of B7-1 on the surface of colonic lamina propria mononuclear cells. IgG isotype-labelled cells was used as a negative control. **e** The population of B7-1^+^ cells in total colonic lamina propria mononuclear cells **(**LPMCs) (*n* = 4 in each group). **f** Immunofluorescence staining of B7-1 (red), F4/80 (green) and DAPI (blue) in colon sections. Double-positive (red and green) signals indicate B7-1^+^ macrophages. Scale bar: 100 μm. Each immunofluorescence staining was repeated independently three times, and representative images are shown. **g** Quantitative RT-PCR analysis of the relative expression levels of integrin α4 mRNA in the colon (*n* = 6 in each group). **h** Representative flow cytometric plots of integrin α4 on the surface of mononuclear cells derived from the colonic lamina propria. IgG isotype-labelled cells was used as a negative control. **i** The population of integrin α4^+^ lymphocytes in total colonic lamina propria lymphocytes (*n* = 4 in each group). **j** Immunofluorescence staining of integrin α4 (red), CD4 (green) and DAPI (blue) in colon sections. Double-positive (red and green) signals indicate integrin α4^+^ CD4^+^ cells. Scale bar: 100 μm. Values are presented as the mean ± SEM. Significance was determined using one-way ANOVA followed by Dunnett’s multiple comparison in (**a**–**c**, **e**, **g**, **i**). *p < 0.05; **p < 0.01; ***p < 0.005.

At the molecular level, the knockdown efficiency of the corresponding target genes was evaluated. First, a remarkable reduction in TNF-α mRNA and protein levels was observed in the colon of mice injected with the CMV-siR^TNF-α^ or CMV-siR^T+B+I^ circuit (Fig. 8a, b). To further characterise the colonic macrophages that internalised TNF-α siRNA, primary macrophages were specifically isolated from the colonic lamina propria and cultured in conditioned medium for 12 h. Immunofluorescence staining of TNF-α in primary macrophages revealed that TNF-α protein levels were significantly decreased by treatment with CMV-siR^TNF-α^ or CMV-siR^T+B+I^ circuit (Supplementary Fig. 13a). Accordingly, the number of colon-infiltrating macrophages, as measured by flow cytometric analysis of F4/80^+^ cells in colonic lamina propria macrophages, was significantly decreased by treatment with the CMV-siR^TNF-α^ or CMV-siR^T+B+I^ circuit (Supplementary Fig. 13b and 13c). Second, the levels of B7-1 mRNA were significantly reduced in the colon of mice injected with the CMV-siR^B7-1^ or CMV-siR^T+B+I^ circuit (Fig. 8c). Flow cytometry also revealed that the colonic lamina propria mononuclear cells, peripheral blood mononuclear cells and splenic mononuclear cells isolated from DSS mice treated with the CMV-siR^B7-1^ or CMV-siR^T+B+I^ circuit exhibited reduced amounts of B7-1 protein (Fig. 8d, e and Supplementary Fig. 14a–d). Immunofluorescence staining of B7-1 in primary cultured macrophages of DSS mice also confirmed an apparent reduction of B7-1 protein after treatment with the CMV-siR^B7-1^ or CMV-siR^T+B+I^ circuit (Supplementary Fig. 14e). Furthermore, double staining of B7-1 and F4/80 in colon sections of DSS mice revealed that the number of double-positive B7-1^+^ F4/80^+^ cells increased remarkably in CMV-scrR circuit-treated DSS mice compared with normal mice, but this increase was abrogated by treatment with the CMV-siR^B7-1^ or CMV-siR^T+B+I^ circuit, especially the latter (Fig. 8f and Supplementary Fig. 14f). These results suggest that B7-1-positive macrophages are increased at sites of intestinal inflammation, whereas intravenous injection of genetic circuits targeting B7-1 causes a synergistic decline in B7-1 protein and colon-infiltrating macrophages in inflamed mucosa. Since the B7-1 molecule on APCs provides costimulatory signals for T cell activation, we evaluated T cell activation using CD25 as a marker^43^. Flow cytometry revealed that after CMV-siR^B7-1^ or CMV-siR^T+B+I^ circuit treatment, the positive rate of CD25^+^ T cells was significantly reduced (Supplementary Fig. 14g, h). Third, a significant decrease in integrin α4 mRNA was observed in the colon of DSS mice injected with CMV-siR^Integrin α4^ or CMV-siR^T+B+I^ circuit (Fig. 8g), which was accompanied by a significant decline in integrin α4 protein levels in the membrane surface of lymphocytes derived from the colonic lamina propria, peripheral blood and spleen of these mice (Fig. 8h, i and Supplementary Fig. 15a–d). In the immunofluorescence staining assay, double-positive signals of integrin α4^+^ CD4^+^ cells were readily detected in the colon sections of CMV-scrR circuit-treated DSS mice; however, the double-positive signals were remarkably diminished by treatment with the CMV-siR^Integrin α4^ or CMV-siR^T+B+I^ circuit (Fig. 8j and Supplementary Fig. 15e). To investigate the interaction of integrin α4β7 with their ligands, a solid-phase adhesion assay was performed with plates coated with E-cadherin^41^. The amounts of adherent CD4^+^ T cells isolated from the peripheral blood of CMV-siR^Integrin α4^ circuit- or CMV-siR^T+B+I^ circuit-treated mice were significantly lower than those from CMV-scrR circuit-treated mice (Supplementary Fig. 15f, g), indicating that homing of lymphocytes to the inflamed gut mucosa was abolished by treatment with the CMV-siR^Integrin α4^ or CMV-siR^T+B+I^ circuit. Overall, these results clearly revealed that the self-assembled siRNAs induced by the multi-targeted genetic circuit were efficiently taken up by recipient cells (e.g., macrophages and CD4^+^ T cells), resulting in simultaneous suppression of corresponding target genes in a single session of treatment.

### In vivo therapeutic efficacy of self-assembled TNF-α siRNA in a spontaneous chronic colitis model

**Fig. 9:**
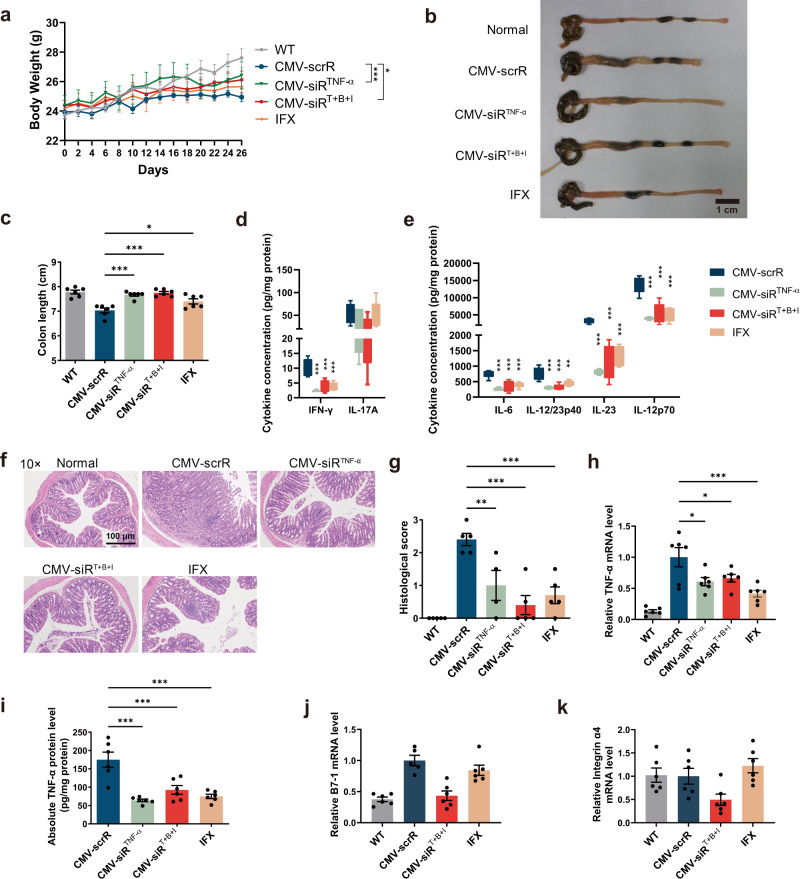
Intravenous injection of the CMV-siR^TNF-α^ or CMV-siR^T+B+I^ circuit ameliorates spontaneous UC in IL-10^−/−^ mice. **a** Body weight curves (*n* = 6 in each group). **b** Representative macroscopic features of colons. Scale bar: 1 cm. **c** Mean colon length (*n* = 6 in each group). **d**, **e** Determination of the levels of IFN-γ, IL-17A, IL-6, IL-12/23p40, IL-23 and IL-12p70 in the colon by ELISA (*n* = 6 in each group). **f** Representative images of H&E staining of colon sections. Scale bar: 200 μm. **g** Histological scores of colon sections (*n* = 5 in WT, CMV-scrR, CMV-siR^T+B+I^ and IFX groups; *n* = 4 in CMV-siR^TNF-α^ group). **h** Quantitative RT-PCR analysis of the relative expression levels of TNF-α mRNA in the colon (*n* = 6 in each group). **i** Determination of the absolute expression levels of TNF-α protein in the colon by ELISA (*n* = 6 in each group). **j**, **k** Quantitative RT-PCR analysis of the relative expression levels of B7-1 and integrin α4 mRNA in the colon (*n* = 6 in each group). Values are presented as the mean ± SEM. Significance was determined using one-way ANOVA followed by Dunnett’s multiple comparison in (**c**–**e**, **g**–**j**, **k**), or two-way ANOVA followed by Dunnett’s multiple comparison in (**a**). *p < 0.05; **p < 0.01; ***p < 0.005.

Interleukin-10 (IL-10) is an anti-inflammatory cytokine that plays a major role in gut homoeostasis. IL-10 knockout (IL-10^−/−^) mice develop spontaneous chronic enterocolitis that closely resembles the phenotypes of human IBD^44^. We administered a single-targeted CMV-siR^TNF-α^ circuit and a multi-targeted CMV-siR^T+B+I^ circuit to IL-10^−/−^ mice and evaluated the therapeutic effects of self-assembled siRNAs in this spontaneous colitis model. Eleven-week-old IL-10^−/−^ mice were intravenously injected with equal dose (10 mg/kg) of CMV-scrR, CMV-siR^TNF-α^ or CMV-siR^T+B+I^ circuit or with 10 mg/kg infliximab (IFX) every two days. After 14 injections, the mice were euthanized, and symptoms and histology were evaluated. Untreated C57BL/6 mice were included as normal controls. Compared to wild-type normal mice, CMV-scrR circuit-treated IL-10^−/−^ mice developed severe gut inflammation at approximately 8 weeks of age, which was characterised by substantial weight loss and a shorter colon length (Fig. 9a–c). In contrast, treatment with the CMV-siR^TNF-α^ and CMV-siR^T+B+I^ circuits significantly attenuated the loss of body weight and shortening of the colon in IL-10^−/−^ mice (Fig. 9a–c). Notably, the pro-inflammatory cytokines of the Th1 and Th17 classes, including IFN-γ, IL-17A, IL-6, IL-12/23p40, IL-23 and IL-12p70, were abundantly present in the colon of CMV-scrR circuit-treated IL-10^−/−^ mice, but these pro-inflammatory cytokines were significantly diminished in the colon of IL-10^−/−^ mice following treatment with CMV-siR^TNF-α^ and CMV-siR^T+B+I^ circuits (Fig. 9d, e). Histological examination detected obvious pathological signs of colitis in CMV-scrR circuit-treated IL-10^−/−^ mice, including epithelial hyperplasia, crypt abscesses, ulceration, bowel wall thickening and massive leucocytic infiltration, but the IL-10^−/−^ mice treated with CMV-siR^TNF-α^ and CMV-siR^T+B+I^ circuits exhibited much better consistency and morphology of colons without infiltrating inflammatory cells, and the histological scores of these mice were significantly lower than the CMV-scrR circuit-treated mice (Fig. 9f, g). Although infliximab protected IL-10^−/−^ mice from loss of body weight, shortening of colon lengths and production of excessive pro-inflammatory cytokines and mitigated the histological signs of colitis in colon sections, its therapeutic effect was inferior to the CMV-siR^T+B+I^ circuit (Fig. 9a–g). At the molecular level, treatment with the CMV-siR^TNF-α^ circuit, CMV-siR^T+B+I^ circuit and infliximab substantially reduced TNF-α levels in the colon tissues (Fig. 9h, i), but only the CMV-siR^T+B+I^ circuit knocked down B7-1 and integrin α4 levels in the colons of IL-10^−/−^ mice (Fig. 9j, k). Taken together, these data demonstrate that the in vivo self-assembled siRNAs limit excessive inflammation and promote intestinal recovery in a spontaneous chronic colitis model.

### Development of an AAV9-based strategy for long-term self-assembly and delivery of sEV-enclosed siRNAs for the treatment of UC

Next, we focused on optimising the delivery vehicles of genetic circuits to achieve a long-term therapeutic effect. Since adeno-associated virus (AAV) is clinically safe and capable of establishing long-term transgene expression, we sought to investigate whether AAV-based expression of a genetic circuit enabled long-term self-assembly of siRNAs in the liver and induced constant target gene silencing in vivo^45^. We inserted the whole sequence of CMV-siR^TNF-α^ or CMV-scrR circuit into an AAV serotype 9 (AAV9) vector (hereafter, AAV9-CMV-siR^TNF-α^ and AAV9-CMV-scrR) and evaluated the therapeutic effects in a chronic UC model. On week 0, BALB/c mice were intravenously injected with 100 μL AAV9-CMV-scrR (1.0 × 10^12^ V. G/mL) or 25, 50 or 100 μL AAV9-CMV-siR^TNF-α^ (1.0 × 10^12^ V. G/mL) (Supplementary Fig. 16a). Three weeks later, chronic UC was induced by rhythmically administering to mice 2.5% DSS for 1 week and water for 2 weeks and the cycle was repeated for 3 times. Symptoms and histology were evaluated at week 10. Untreated BALB/c mice were included as normal controls. Since the AAV9 vector could co-express TNF-α siRNA and a luciferase reporter, evaluation of luciferase activity might reflect TNF-α siRNA accumulation in vivo. AAV9-mediated luciferase expression was dose-dependently increased from week 1, reached a peak at week 3 and decreased to the background level at week 10 (Supplementary Fig. 16b and 16c). Compared with treatment with AAV9-CMV-scrR, treatment with a high dose of AAV9-CMV-siR^TNF-α^ caused a significant recovery of body weight and colon length, a significant decline in the DAI score and pro-inflammatory cytokine levels and an apparent alleviation of the histological appearance in DSS mice (Supplementary Fig. 16d–i). At the molecular level, treatment with a high dose of AAV9-CMV-siR^TNF-α^ resulted in a significant accumulation of TNF-α siRNA in plasma, and consequently, an apparent reduction in TNF-α mRNA and protein levels was observed in colon tissue (Supplementary Fig. 17). These results revealed that AAV9-mediated expression of a genetic circuit provided a substantial and lasting therapeutic effect following a single administration.

Subsequently, the multi-targeted CMV-siR^T+B+I^ circuit was inserted into the AAV9 vector (hereafter, AAV9-CMV-siR^T+B+I^) to induce long-term combination therapy in a chronic UC model. On week 0, BALB/c mice were intravenously injected with 100 μL AAV9-CMV-scrR (1.0 × 10^12^ V. G/mL) or 25, 50 or 100 μL AAV9-CMV-siR^T+B+I^ (1.0 × 10^12^ V. G/mL) (Supplementary Fig. 18a). Three weeks later, chronic UC was induced by rhythmically administering to mice 2.5% DSS for 1 week and water for 2 weeks and the cycle was repeated for 3 times. Symptoms and histology were evaluated at week 10. Untreated BALB/c mice were included as normal controls. AAV9-CMV-siR^T+B+I^-treated mice recovered body weight quite fast after each DSS administration, especially those in the high-dose group (Supplementary Fig. 18b). Likewise, AAV9-CMV-siR^T+B+I^-treated mice experienced a significant recovery in colon length, had a dramatic decrease in the DAI score and pro-inflammatory cytokine levels and displayed a striking improvement in histological signs compared with mice treated with AAV9-CMV-scrR (Supplementary Fig. 18c–h). Remarkably, while colon tissues from the AAV9-CMV-scrR-treated mice exhibited epithelial disruption, goblet cell depletion and significant infiltration of inflammatory cells into the mucosa, colons from the AAV9-CMV-siR^T+B+I^-treated mice showed a relatively normal histology, with no clear signs of inflammation or disruption of tissue morphology (Supplementary Fig. 18g). At the molecular level, treatment with AAV9-CMV-siR^T+B+I^ resulted in a dose-dependent increase in TNF-α siRNA, B7-1 siRNA and integrin α4 siRNA in the liver, plasma and colon of DSS mice (Supplementary Fig. 19a–i), which was accompanied by a dose-dependent reduction in TNF-α, B7-1 and integrin α4 in colon tissues (Supplementary Fig. 19j–l).

### Development of optimised AAV8-TBG-mediated liver-specific expression and assembly of siRNAs for the treatment of UC

Although we optimised the delivery vehicles of the genetic circuit and developed an AAV9-based strategy for permanent self-assembly of sEV-enclosed siRNAs for the treatment of UC, the therapeutic effects observed in UC models may not be liver dependent because AAV9 has tropism for a wide range of tissues, and the CMV promoter is a strong constitutive promoter without tissue specificity. AAV8 has strong liver tropism^46,47^, and the thyroxine-binding globulin (TBG) promoter is a hepatocyte-specific promoter^48,49^. Therefore, the expression cassette designed for tandem expression of TNF-α siRNA, B7-1 siRNA and integrin α4 siRNA was inserted downstream of the TBG promoter, and the entire circuit was further incorporated into an AAV8 vector (hereafter, AAV8-TBG-siR^T+B+I^). Such an AAV8-TBG-driven genetic circuit ensures that the corresponding siRNAs are transcribed only in hepatocytes while avoiding undesired siRNA expression in extrahepatic cells.

**Fig. 10:**
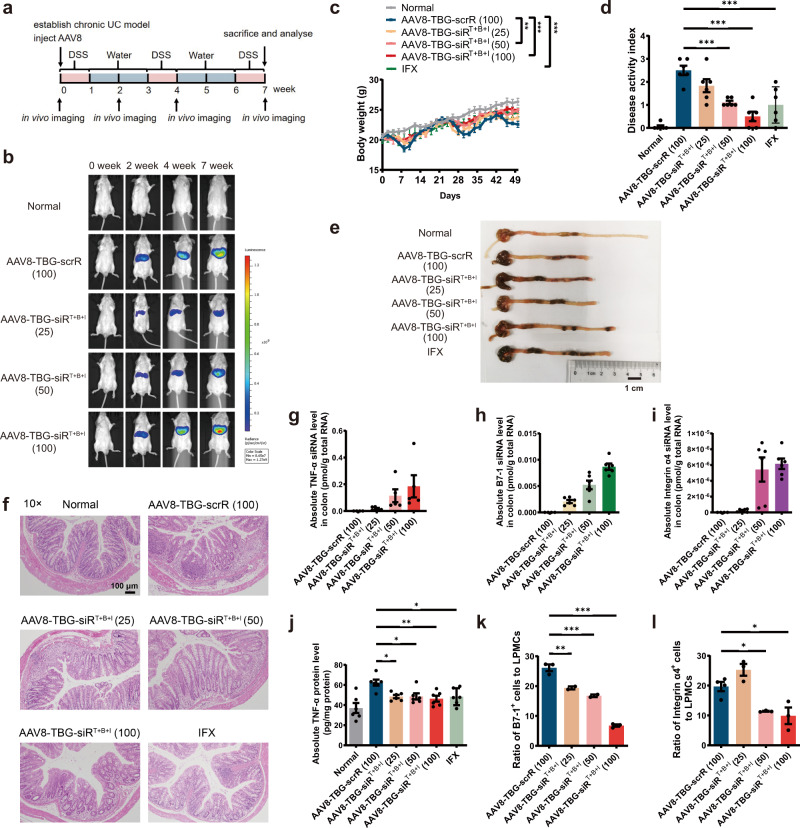
Intravenous injection of the AAV8-TBG-siR^T+B+I^ induces long-term combination therapy in the DSS-induced chronic UC model. **a** Flow chart of the experimental design. **b** Evaluation of AAV-mediated luciferase expression to reflect co-expressed siRNA accumulation in vivo (*n* = 6 in each group). **c** Body weight curves (*n* = 6 in each group). **d** DAI scores (*n* = 6 in each group). **e** Representative macroscopic features of colons. Scale bar: 1 cm. **f** Representative images of H&E staining of colon sections. Scale bar: 100 μm. Each H&E staining was repeated independently three times, and representative images are shown. **g**–**i** Quantitative RT-PCR analysis of the absolute expression levels of TNF-α siRNA, B7-1 siRNA and integrin α4 siRNA in the colon (*n* = 5–6 in each group). **j** Determination of the absolute expression levels of TNF-α protein in the colon by ELISA (*n* = 6 in each group). **k** The population of B7-1^+^ cells in total colonic lamina propria mononuclear cells (*n* = 2–3 in each group). **l** The population of integrin α4^+^ lymphocytes in total colonic lamina propria lymphocytes (*n* = 3–4 in each group). Values are presented as the mean ± SEM. Significance was determined using one-way ANOVA followed by Dunnett’s multiple comparison in (**d**, **j**, **k**, **l**) or using two-way ANOVA followed by Dunnett’s multiple comparison in (**c**). *p < 0.05; **p < 0.01; ***p < 0.005.

We evaluated the therapeutic effects of AAV8-TBG-siR^T+B+I^ in a chronic UC model (Fig. 10a). On week 0, chronic UC was induced by rhythmically administering to BALB/c mice 2.5% DSS for 1 week and water for 2 weeks, and the cycle was repeated for 3 times. At the same time, mice were intravenously injected with 100 μL AAV8-TBG-scrR (3 × 10^12^ V. G/mL) or 25, 50 or 100 μL AAV8-TBG-siR^T+B+I^ (3 × 10^12^ V. G/mL), and the symptoms and histology were evaluated at week 7. For the control group receiving infliximab, 4 days after each DSS drinking, mice were intravenously injected with 10 mg/kg infliximab for a total of 3 times, once every 2 days. Untreated BALB/c mice were included as normal controls. AAV8-TBG-mediated luciferase expression was dose-dependently increased from weeks 2 to 7 and remained stable over 7 weeks, and the luciferase signal was primarily restricted to the liver with negligible signals in extrahepatic tissues (Fig. 10b and Supplementary Fig. 20a, b). Control mice receiving AAV8-TBG-scrR showed typical characteristics of colitis in the chronic UC model, such as sustained body weight loss, increased DAI score, shortened colon length, enhanced inflammatory cytokine levels and apparent histological features in colonic sections. However, the mice receiving a high dose of AAV8-TBG-siR^T+B+I^ rapidly recovered the lost body weight and colon length, experienced a low DAI score, showed a significant decline in inflammatory cytokines and exhibited an improved colonic histological appearance (Fig. 10c–f and Supplementary Fig. 20c–f). At the molecular level, treatment with AAV8-TBG-siR^T+B+I^ resulted in a dose-dependent production of TNF-α siRNA, B7-1 siRNA and integrin α4 siRNA in the liver (Supplementary Fig. 21a–c), which were accompanied by a dose-dependent accumulation of TNF-α siRNA, B7-1 siRNA and integrin α4 siRNA in the colon (Fig. 10g–i). Consequently, dose-dependent reductions in colonic TNF-α, B7-1 and integrin α4 mRNA levels were detected after AAV8-TBG-siR^T+B+I^ treatment (Supplementary Fig. 21d–f). ELISA confirmed a dose-dependent decline in TNF-α protein levels in the colonic lamina propria derived from AAV8-TBG-siR^T+B+I^-treated mice (Fig. 10j), and flow cytometry confirmed a dose-dependent loss of B7-1 and integrin α4 proteins on the membrane surface of mononuclear cells derived from the colonic lamina propria, peripheral blood and spleen of AAV8-TBG-siR^T+B+I^-treated mice (Fig. 10k, l and Supplementary Figs. 22, 23). Infliximab also exhibited significant therapeutic activity in the chronic UC model, but AAV8-TBG-siR^T+B+I^ ameliorated the manifestations of chronic colitis and restored the expression of TNF-α, B7-1 and integrin α4 to a better extent than infliximab (Fig. 10c–l and Supplementary Figs. 20c–f, 21, 22, 23).

Because AAV8 induced long-term transgene expression in the liver, we assessed whether AAV8-TBG-mediated production, assembly and delivery of siRNAs produced toxic effects in certain tissues, especially the liver. The mice treated with different doses of AAV8-TBG-siR^T+B+I^, like AAV8-TBG-scrR-treated mice and untreated normal mice, did not exhibit apparent toxicity in the liver and other tissues. A panel of 6 serum biochemical parameters remained within the normal range for each group, except ALT (a sensitive marker of hepatocyte necrosis or damage) exhibited a gradual increasing trend after treatment with increasing amounts of AAV8-TBG-siR^T+B+I^, but without statistical significance (Supplementary Fig. 24a–f). Histological analyses also detected no pathological changes or signs of inflammation in the liver and kidney sections derived from AAV8-TBG-siR^T+B+I^-treated mice (Supplementary Fig. 24g). Overall, these results suggest AAV8-TBG-mediated liver expression and assembly of siRNAs as a potential solution to induce long-term combination therapy for chronic UC, with an acceptable safety profile.

## Discussion

Despite recent advancements in our knowledge of the pathogenic mechanisms of UC, a considerable unmet medical need for the treatment of UC remains. Overactive immune responses are the most promising therapeutic targets, and biological therapy using monoclonal antibodies (e.g., infliximab) has been specifically developed to block immunological targets and alleviate immune responses^50,51^. However, the high treatment costs, serious side effects and development of drug resistance associated with the development of autoimmunity to antibodies remain serious problems for biological therapy^51,52^. Therefore, it is urgent to develop an alternative strategy with high therapeutic efficiency and few side effects. Because siRNA is solely dependent on the mRNA sequence and inhibits immunological targets with strong specificity, RNAi therapy has the intrinsic ability to overcome the shortcomings of biological therapy^53,54^. Unfortunately, the lack of safe and effective carriers for the delivery of siRNA therapeutics remains a major barrier to its broad clinical application, especially for extrahepatic delivery situations. An ideal siRNA carrier must overcome a series of biological hurdles throughout the course of delivery: it should have no stimulatory effects on immune systems, protect siRNA from degradation by RNases in the internal environment, have proper permeability of the cell membrane, possess a suitable binding strength with siRNA and enable escape from lysosomes before the release of siRNAs into the cytoplasm of target cells. Kedmi et al. recently developed a self-assembled modular platform based on a membrane-anchored lipoprotein that was incorporated into siRNA-loaded lipid nanoparticles that interacted with the antibody crystallisable fragment (Fc) domain^55^. The therapeutic potential of the platform was demonstrated in an IBD model by targeting colon macrophages to reduce inflammatory symptoms. This study demonstrated the superiority of the self-assembled modular platform for the delivery of siRNAs to targeted cell populations in vivo. Notably, it established a promising tool that may be simply translated into the clinic because the self-assembly strategy is quite simple and avoids the massive development and production requirements and the high batch-to-batch variability of current technologies. We used a similar concept of self-assembly based on the nature of sEVs because sEVs and their cargoes are self-assembled by endogenous cells. Based on the intrinsic ability of the small RNA processing machinery of the host liver to self-assemble siRNA into sEVs and the endogenous circulating system of sEVs to transport siRNAs, we designed genetic circuits to engineer mouse liver to self-assemble siRNAs into secretory sEVs and deliver siRNA-encapsulating sEVs via the blood circulation to the sites of intestinal inflammation, which resulted in significant target gene reduction and symptom alleviation in acute and chronic UC models. Notably, genetic circuits showed an overall advantage over infliximab in alleviating the symptoms and signs of UC in different UC models (Supplementary Table 1). Because sEVs harbour excellent cellular compatibility and less immunogenicity than other delivery vehicles, no cytotoxicity, immunogenicity or side effects were observed after treatment with genetic circuits.

Combination therapy is emerging as an attractive therapeutic option for UC^56^. The basic concept behind this strategy is quite straightforward: simultaneous engagement of two or more different targets by a single drug formulation may have a synergistic therapeutic effect^57^. However, monoclonal antibodies are usually used alone rather than in combination with other monoclonal antibodies, considering both the possibility of an increased risk of immunogenicity and side effects. Conversely, because the design of siRNAs is quite simple and flexible, RNAi-based combination therapy conducted by the codelivery of multiple siRNAs enables the simultaneous blocking of two or more different target genes or pathways in a single treatment^19^. In this study, we developed an upgraded and innovative RNAi strategy through the design of a multi-targeted CMV-siR^T+B+I^ circuit carrying a siRNA expression cassette organised as a head-to-tail tandem array under the control of a single CMV promoter. This multi-targeted circuit reprogrammes the host liver to trigger the self-assembly of TNF-α siRNA, B7-1 siRNA and integrin α4 siRNA into secretory sEVs and facilitate the in vivo delivery of these siRNAs to colonic macrophages and CD4^+^ T cells. The improvements of the multiple-target strategy are reflected by two findings. First, in mouse models of UC, the multi-targeted CMV-siR^T+B+I^ circuit exerted strong inhibitory activities on each of the corresponding target genes and exhibited a synergistic and cooperative therapeutic effect compared with the single-targeted CMV-siR^TNF-α^, CMV-siR^B7-1^ or CMV-siR^Integrin α4^ circuit. Second, the multi-targeted CMV-siR^T+B+I^ circuit could simply decrease the dosages of plasmid and AAV carrying the genetic circuit when compared with the coinjection of three kinds of single-targeted circuits, thereby contributing to a reduction in the cytotoxicity, immunogenicity and side effects caused by the plasmid and AAV themselves. Overall, the multi-targeted genetic circuit offers an easy-to-use, effective and safe combination therapeutic RNAi strategy, which is superior to conventional biological therapies where two or more independent compounds or antibodies are needed.

Considering the short in vivo half-life of the genetic circuits formed as naked DNA plasmids, repeated injection of genetic circuits is inevitable for the long-term treatment of chronic UC. The problems associated with repeated injection should be dealt with properly^58^. To develop a strategy for permanent self-assembly of sEV-enclosed siRNAs for the treatment of chronic UC, we selected AAV as the carrier of genetic circuits because AAV is capable of establishing long-term transgene expression with minimal immunogenicity, toxicity and side effects^16^. A growing number of human clinical trials have used AAVs, achieving a good safety profile and significant clinical benefit in many diseases^45^. The advantage of AAV-driven genetic circuits is that they can carry multiple distinct siRNA expression cassettes targeting different causal genes of chronic UC to improve therapeutic outcomes via a single administration. We showed that AAV9-CMV- and AAV8-TBG-mediated production and assembly of multiple siRNAs caused substantial and lasting inhibition of the corresponding target genes in chronic UC models. The therapeutic benefit was comparable between a single injection of the AAV-driven genetic circuits and repeated injections of the plasmid-based genetic circuits. Although the AAV9 vector and CMV promoter exhibit high efficiency in a broad range of tissues, the AAV8 vector and TBG promoter have a strong liver tropism, which validates that the liver is the original site for the uptake of genetic circuits and self-assembly and secretion of siRNA-encapsulating sEVs. We confirmed that peripheral infusion of an AAV8-TBG-driven genetic circuit constantly generated self-assembled siRNAs in a safe, non-toxic and biocompatible manner. However, the expression levels of TNF-α siRNA, B7-1 siRNA and integrin α4 siRNA were decreased in the liver when the CMV promoter was replaced with a liver-specific TBG promoter in the genetic circuits, regardless of whether the genetic circuits were placed in an AAV or plasmid. This difference may be due to the lower efficiency of the TBG promoter in transcribing siRNAs than the CMV promoter, which is consistent with the results from previous studies^59^. Therefore, although the AAV8-TBG-driven genetic circuit yielded promising therapeutic outcomes in DSS-induced chronic UC models, there is much room for improvement. Nevertheless, our results provide broad insight into how a liver-tropic, AAV-based RNAi therapeutic strategy alleviated disease progression and promoted immune balance recovery in chronic UC, which led to mucosal restoration in disease sites and reduced the likelihood of adverse side effects. Although more work is needed to verify the therapeutic effects and ensure safety, AAV-driven genetic circuits hold strong promise for becoming an option for the treatment of complex chronic diseases, such as UC.

However, the exact mechanism underlying the self-assembly of siRNAs into sEVs is not known. Whether the loading and packaging of specific siRNAs into sEVs occur in an active (selective) or passive (random) manner remains controversial. Recent studies on secretory miRNAs may provide some inspiration for this question^60^. There is mounting evidence that cells selectively package certain miRNAs into EVs for active secretion^61–63^. However, the mechanism by which miRNAs are sorted into EVs or retained in cells remains elusive. EVs are a heterogeneous group of endogenous nanosized membrane vesicles that are generally categorised into two subtypes: exosomes (30–100 nm in diameter) that are derived from the luminal membrane of multivesicular bodies (MVBs) and released via exocytosis and microvesicles (100–1000 nm in diameter) that are shed directly from the plasma membrane^64^. Because of the heterogeneous nature of EVs, the molecular mechanisms responsible for the sorting and packaging of siRNAs into EVs must be quite diverse depending on the biogenesis routes, cargo compositions and functional specialisation of different EV subtypes. To simplify this complex diversity, we only used exosomes and exosome-enclosed siRNAs as examples. Exosome generation includes the following steps: (1) the cytoplasmic membrane invaginates to form an early endosome; (2) the payload sprouts inward to form intraluminal vesicles contained within the endosome and creates a structure classically described as MVB; and (3) intraluminal vesicles are released to the extracellular space as exosomes upon fusion of the MVB with the plasma membrane^65,66^. During the exosome formation process, materials from the cell cytoplasm, including proteins and miRNAs, are specifically sorted and encapsulated into intraluminal vesicles, which are the precursors of exosomes^65^. In our strategy, TNF-α/B7-1/integrin α4 siRNA was embedded in a pre-miR-155 backbone, which borrowed the endogenous miRNA processing machinery of the host liver to produce siRNAs and self-assemble siRNAs into secretory sEVs^19^. Therefore, the primary means of siRNA loading into exosomes may be equivalent to miRNAs. However, recent studies identified some specific short motifs in mature miRNAs that control miRNA sorting into exosomes^60^. Although these findings emphasise the importance of the miRNA sequence for material cargo sorting, the siRNA sequences designed for the silencing of TNF-α, B7-1 and integrin α4 did not harbour these motifs, which excludes the possibility of sequence-mediated selective sorting of siRNAs into exosomes. However, we observed a dose-dependent increase in intracellular TNF-α siRNA levels when an increased amount of CMV-siR^TNF-α^ circuit was transfected into HEK293T cells, which was accompanied by a dose-dependent increase in TNF-α siRNA in secreted sEVs. These results suggest another possibility in which the CMV promoter drives the transcription and accumulation of siRNAs in the cytoplasm of hepatocytes and leads to the loading and packaging of saturated cytoplasmic siRNAs into sEVs as cargo. Taken together, the molecular mechanisms underlying the selective or passive assembly of siRNAs into sEVs have not been elucidated. Because the promoter, siRNA sequence and pre-miRNA structure may join together to decide the sorting route of siRNAs to sEVs in the genetic circuit design, there is much room for optimising the biodistribution of siRNAs to guarantee the preferential sorting of siRNAs into sEVs rather than retaining them within cells.

Biological therapies, particularly anti-TNF-α agents (e.g., infliximab) and anti-integrin agents (e.g., natalizumab), have substantially extended the therapeutic armamentarium of IBD in the last decade. Combined immunosuppression using biological therapies and immunomodulators has become standard in the medical management of moderate-to-severe IBD because of clearly demonstrated efficacy^67^. However, the combination of immunosuppressive medications is also a risk factor for opportunistic infections. For example, infliximab-treated patients are susceptible to infections with opportunistic pathogens, including *Mycobacterium tuberculosis*, *Histoplasma capsulatum*, *Coccidioides immitis*, *Pneumocystis* and *Cytomegalovirus*^68^. Positive prevention, regular monitoring and timely control of opportunistic infections are the current focuses for improving the prognosis of IBD patients^69^. Therefore, a combination therapy towards multiple immunological targets may function as a “double-edged sword”. Although combination therapy may suppress inflammatory responses more rapidly and effectively, it may increase the risk of opportunistic infections. Therefore, an alternative strategy that enhances the immunomodulatory effects and minimises the undesired side effects should be developed. The present study raised the possibility that simultaneous inhibition of TNF-α, B7-1 and integrin α4 with multiple self-assembled siRNAs, via a distinct mechanism other than biological therapies and immunomodulators, rapidly relieved intestinal inflammation and exerted a synergistic therapeutic effect against UC. We did not observe abnormal phenotypes, apparent tissue damage or severe side effects in the liver, spleen, lung or kidney after the injection of genetic circuits, although self-assembled siRNAs were also delivered to the kidney, spleen and peripheral blood monocytes and CD4^+^ T cells apart from the colon. These results suggest that in vivo self-assembled siRNA is a promising combination therapeutic strategy for UC with an acceptable safety profile. However, the animal models were maintained under specific pathogen-free conditions in this study, and there is a certain risk that the multi-targeted self-assembled siRNAs may induce excessive immunosuppression and render the animals/patients highly susceptible to opportunistic infections in the real world. Therefore, the concerns associated with the combined immunosuppression mediated by self-assembled siRNAs should be addressed properly. First, the safety, dosing and durability of self-assembled siRNAs must be adequately fine-tuned. Second, the biodistribution of siRNAs in various tissues and immune cells must be measured more accurately, and whether the physiological functions and status of these tissues and immune cells are impaired after long-term therapy should be evaluated more systematically. Third, to confer targeting capability on sEVs, the guiding peptides that modify the membrane-anchored proteins of sEVs should be specifically designed and integrated into the genetic circuits to endow the modified sEVs with the ability to target immune cells with a high affinity and facilitate the preferential delivery of siRNAs into immune cells to minimise undesired off-target effects. Fourth, the therapeutic potential of self-assembled siRNAs has only been tested in mouse models and only in male mice. To clarify the generalisability of self-assembled siRNAs, their efficacy and safety should be further verified in other animal models and in female mice.

Taken together, this study induced controllable and predictable self-assembly and delivery of multiple siRNAs in mouse models of UC and allowed simultaneous control of multiple immunological targets in a purpose-driven mode. This technology has important theoretical significance and translational value because it may provide a feasible and promising solution for combination therapy in UC.

## Methods

### Animals

Six-week-old male BALB/c mice were purchased from the Model Animal Research Center of Nanjing University. Eleven-week-old male IL-10^-/-^ mice and wild-type C57BL/6 mice were kindly gifted by Prof. Zhen Huang (BioMed Laboratory, Nanjing University, China). The animals were housed under specific pathogen-free conditions and maintained in a temperature-controlled room with a 12 h light/dark cycle. The animals were provided ad libitum access to water and standard laboratory chow. The well-being of the animals was monitored daily. All animal experiments were performed in accordance with the Guide for the Care and Use of Laboratory Animals published by the National Institutes of Health (NIH Publication 85-23 (revised 1996)) and met the approval of the Animal Ethical and Welfare Committee of Nanjing University (approval number: IACUC-2011006). Euthanasia was performed by carbon dioxide inhalation.

### Design and construction of the genetic circuits

The CMV-siR^TNF-α^ circuit was generated by inserting a TNF-α siRNA sequence (5′-CCATTTGGGAACTTCTCATCC-3′) into a 166-bp pre-miR-155 backbone with structurally conserved nucleotide changes to maintain pairing (GGATCCTGGAGGCTTGCTGAAGGCTGTATGCTGAATTCGCCATTTGGGAACTTCTCATCCGTTTTGGCCACTGACTGACGGATGAGATTCCCAAATGGCAACCGGTCAGGACACAAGGCCTGTTACTAGCACTCACATGGAACAAATGGCCCAGATCTGGCCGCACTCGAG). For the CMV-siR^T+B+I^ circuit designed to co-express TNF-α siRNA, B7-1 siRNA and integrin α4 siRNA, two tandem pre-miR-155 backbones carrying the B7-1 siRNA sequence (5′- AAGAGAAACTAGTAAGAGTCT-3′) and the integrin α4 siRNA sequence (5′- ATCACATGATGCCCAAGGTGG -3′) were cloned downstream of the TNF-α siRNA element. A circuit designed to express a scrambled RNA was used as the negative control. The plasmids used to express genetic circuits were synthesised and constructed by Realgene Biotech Company (Nanjing, China). Maps and scaffolds of the plasmids are shown in Supplementary Fig. 25. The plasmids were transformed into *E. coli* DH5α competent cells (Tsingke, TSC01, Beijing, China), cultured with LB medium (with 50 µg/mL spectinomycin) for 14 h in a 37 °C shaking incubator and extracted and purified with an EndoFree Maxi Plasmid Kit V2 (Tiangen, DP120, Beijing, China) according to the manufacturer’s instructions. The purified plasmids were sequenced to ensure that the sequences of the inserted genetic circuits were correct. Sequences of the siRNAs designed for silencing of TNF-α, IL-17A, IFN-γ, IL-6, integrin α4, ICAM-1, CD3 and B7-1 are shown in Supplementary Table 2. Sequences of the siRNA expression cassettes are shown in Supplementary Fig. 26.

### Cell culture

The mouse macrophage cell line ANA-1 (CL-0023) were purchased from Procell Life Science&Technology Co.,Ltd (Wuhan, China). Human embryonic kidney cell line HEK293T (GNHu 43) were purchased from the Shanghai Institute of Cell Biology, Chinese Academy of Sciences (Shanghai, China). ANA-1 cells were cultured in RPMI-1640 (Gibco, 11875093, MA, USA). HEK293T cells were cultured in high-glucose (4.5 g/L) DMEM (Gibco, 10564011, MA, USA). All media were supplemented with 10% foetal bovine serum (FBS, Gibco, 10099141 C, MA, USA), penicillin and streptomycin in a 5% CO_2_, water-saturated atmosphere. All cell lines were identified by STR (Short Tandem Repeat) profiling by the source. Cells were expanded after being received and subsequently stored in liquid nitrogen. The storage vials were thawed for experiments and used within 2 months. Regular mycoplasma test showed both cell lines were mycoplasma negative.

### DSS-induced UC model and TNBS-induced colitis model

Acute UC was induced by feeding mice 2.5% dextran sodium sulphate solution (DSS, Millipore, Billerica, MA, USA) dissolved in physiological saline^70^. Seven days later, the mice were allowed to recover with normal drinking water for 3 days. From day 3 of modelling, the mice were intravenously injected with 20 mg/kg CMV-scrR or three dosages (0.5, 5 and 20 mg/kg) of CMV-siR^TNF-α^ circuit or 20 mg/kg infliximab (IFX, Meilunbio, Dalian, China) once a day for seven times until they were euthanised on day 10. Some of the tissue sample was fixed in 3.7% paraformaldehyde overnight at 4 °C, dehydrated in ethanol and embedded in paraffin. The sections were stained with haematoxylin and eosin (H&E) for observation of general histological changes. The remaining tissues were frozen in liquid nitrogen for molecular biological analyses.

For the DSS-induced chronic UC model, BALB/c mice were allowed to rhythmically drink 2.5% DSS for 1 week and normal water for 2 weeks, and the cycle was repeated 3 times^71^. For the single-targeted genetic circuits, four days after each DSS drinking, the mice were intravenously injected with PBS or 20 mg/kg CMV-scrR, 20 mg/kg CMV-siR^TNF-α^ or 20 mg/kg infliximab for a total of 3 times, once every 2 days. For the multi-targeted genetic circuits, four days after each DSS drinking, the mice were intravenously injected with PBS or equal dose (10 mg/kg) of CMV-scrR, CMV-siR^TNF-α^, CMV-siR^Integrin α4^, CMV-siR^B7-1^ or CMV-siR^T+B+I^ circuit for a total of 3 times, once every 2 days. One day after the final injection on day 52, the mice were euthanised and analysed.

A TNBS-induced acute colitis model was established by intracolonic administration of 2.5% TNBS solution^70^. Briefly, BALB/c mice were fasted for 48 h with free access to drinking water to remove as much stool as possible and then anaesthetised with pentobarbital sodium. A polyethylene catheter (2 mm in outer diameter) was inserted rectally until the splenic flexure (5-8 cm from the anus). Mice were rectally administered 2.0-2.5 mg of TNBS (Sigma-Aldrich, St Louis, MO, USA) dissolved in 50% ethanol through the catheter and then held in a headfirst position for at least 10 min. One day later, mice were intravenously injected with 20 mg/kg CMV-scrR or three dosages (0.5, 5 and 20 mg/kg) of CMV-siR^TNF-α^ or 20 mg/kg infliximab (IFX) once a day. After 7 injections, the mice were euthanized and analysed.

For the TNBS-induced chronic colitis model, mice were intracolonically administered 2.5% TNBS for 4 times, once a week^71^. After each TNBS administration, mice were intravenously injected with 20 mg/kg CMV-scrR, 20 mg/kg CMV-siR^TNF^ or 20 mg/kg infliximab for a total of 4 times over the first 4 days. Body weights were monitored daily, and symptoms and histology were evaluated on day 26.

In this study, we used a non-invasive normal tail vein injection procedure (100 μL solution was injected in 5 s for each mouse) rather than an invasive hydrodynamic injection procedure (1.6–1.8 mL solution was rapidly injected in 5 s for each mouse) for injection of genetic circuits (in the form of DNA plasmids).

### AAV treatments

For construction of the AAV-driven genetic circuits, the complete sequences of the CMV-siR^TNF-α^ and CMV-siR^T+B+I^ backbones co-expressing a luciferase reporter were inserted into the AAV9 vector, and the complete sequences of the TBG-siR^T+B+I^ backbone co-expressing a luciferase reporter were inserted into the AAV8 vector by HANBIO (Wuhan, China).

For the AAV9-based strategy, BALB/c mice were intravenously injected with 25, 50 or 100 μL AAV9-CMV-siR^TNF-α^ or AAV9-CMV-siR^T+B+I^ (10^12 ^V. G/mL) co-expressing a luciferase reporter. Luciferase activity was monitored at weeks 1, 3 and 10 using non-invasive in vivo bioluminescence imaging. At week 3, the mice were administered DSS to establish a chronic UC model, and the therapeutic effects were evaluated at week 10. For the AAV8-based strategy, BALB/c mice were intravenously injected with 100 μL AAV8-TBG-scrR (3 × 10^12^ V. G/mL) or 25, 50 or 100 μL AAV8-TBG-siR^T+B+I^ (3 × 10^12^ V. G/mL). Meanwhile, chronic UC was induced by rhythmically administering 2.5% DSS for 1 week and water for 2 weeks. Untreated BALB/c mice were included as normal controls. Luciferase activity was monitored at weeks 0, 2, 4 and 7 using non-invasive in vivo bioluminescence imaging. Body weights were monitored every two days, and symptoms and histology were evaluated at week 7. To confirm the tissue specificity of AAV8-TBG-siR^T+B+I^, the mice were sacrificed 14 days after AAV8-TBG-siR^T+B+I^ administration. AAV-mediated luciferase expression was evaluated to reflect co-expressed siRNA accumulation in various tissues of mice.

### Establishment of a spontaneous chronic colitis model in IL-10^−/−^ mice

Eleven-week-old IL-10^−/−^ mice were intravenously injected with equal dose (10 mg/kg) of CMV-scrR, CMV-siR^TNF-α^ or CMV-siR^T+B+I^ circuit or 10 mg/kg infliximab every two days. After 14 injections, the mice were euthanised. Body weights were monitored every two days, and symptoms and histology were evaluated on day 28. Untreated C57BL/6 mice were included as normal controls.

### sEV isolation

Venous blood samples of mice (~1 mL) were collected and placed in a plasma separator tube. The plasma was separated by centrifugation at room temperature for 10 min at 800 × *g*. To remove cell debris, the plasma was centrifuged at room temperature for 15 min at 10,000 × *g*. Then, a Total Exosome Isolation (from plasma) kit (4484450, Invitrogen, CA, USA) was used for sEV isolation from the plasma according to the manufacturer’s instructions.

Iodixanol density gradient fractionation was performed according to previous studies^32^. Briefly, the differential centrifugation was repeated four times to obtain the crude EVs (at 500 × *g* for 5 min, 3000 × *g* for 25 min 10,000 × *g* for 1 h and 120,000 × *g* for 1.5 h at 4 °C). To obtain highly pure vesicles, density gradient centrifugation was performed. Iodixanol density media (36%, 30%, 24% and 18%) (Sigma-Aldrich, D1556, MO, USA) were prepared in ice-cold PBS immediately. Crude pellets of EV were mixed with iodixanol density media for a final 36% iodixanol solution. The suspension was added to the bottom of a centrifugation tube and solutions of descending concentrations of iodixanol density media were carefully layered on top yielding the complete gradient. The centrifugation tubes were subjected to ultracentrifugation at 120,000 × *g* for 12 h at 4 °C. A 6 mL solution collected from the top of the gradient was transferred to new ultracentrifugation tubes, diluted 12-fold in PBS and subjected to ultracentrifugation at 120,000 × *g* for 4 h at 4 °C. For NTA and TEM analyses, fractions were diluted in particle-free PBS. The resulting pellets were lysed in cell lysis buffer for 30 min on ice for RNA extraction.

### Electron microscopy

sEVs were suspended in 1 × PBS and fixed in 2% paraformaldehyde. The fixed sample was absorbed by a copper mesh coated with formaldehyde in a dry environment for 20 min. The sample was fixed in 1% glutaraldehyde for 5 min. After rinsing in distilled water, the sample was dyed with uranyl oxalate for 5 min and then with uranyl acetate for 10 min on ice. Excess liquid was removed from the mesh with filter paper, and the mesh was stored at room temperature until imaging. Imaging was performed using KYKY-EM3200 microscope (KYKY Technology Company, Beijing, China).

### Nanoparticle tracking analysis

Separated sEVs were analysed using a NanosightLM10 system equipped with a blue laser (405 nm). The nanoparticle is illuminated with a laser, and its Brownian motion is captured for 60 s. At least 3 videos were collected from each individual sample to provide representative concentration measurements. The size distribution curves obtained from the NTA system were averaged within each sample in the video repetition and then averaged between samples to provide a representative size distribution. These profiles were then normalised to the total nanoparticle concentration or final cell count.

### RNA immunoprecipitation

Mice were intravenously injected with equal dose (5 mg/kg) of CMV-scrR or CMV-siR^TNF-α^ circuit every two days. After 7 injections, the mice were euthanized, and mouse plasma was isolated. Then plasma (200 µL) was incubated with RNase inhibitor (20 U/mL) and 5 μg anti-Ago2 antibody (Cell Signaling Technology, 2897, Danvers, MA, USA) or IgG for 12 h at 4 °C on a rocker. Subsequently, 40 µL Protein A/G PLUS-Agarose beads (Santa Cruz, sc-2003, CA, USA) were added to each tube and then rocked with the mixture for 12 h on rocker arms at 4 °C. After centrifugation at 4 °C at 1000 × *g* for 5 min, the supernatant was collected for Western blot analysis and quantitative RT-PCR. The immune precipitate was washed with 1 mL PBS (RNase-free) for 3 times and then centrifuged at 800 × *g* for 3 min at 4 °C. After elution, the beads were collected and divided into two parts: one was lysed in 100 µL lysis buffer for Western blot analysis, while the other was lysed with TRIzol reagent to isolate RNA.

### Western blot analysis

After treatment with Cell Extraction Buffer (FNN0011, Invitrogen, CA, USA), 30 μg total protein of sEVs was used for electrophoresis and transferred to PVDF membranes. The membranes were blocked with skim milk before antibody incubation at room temperature for 1 h. The antibodies used in the experiments were as follows: CD63 antibody (Proteintech, 25682-1-AP, Wuhan, China), CD9 antibody (Proteintech, 20597-1-AP, Wuhan, China), TSG101 antibody (Proteintech, 28283-1-AP, Wuhan, China). The primary antibody (1:1000 dilution) was incubated at 4 °C overnight, and the secondary antibody was incubated at 37 °C for 1 h before detection. The blot was visualised with the enhanced chemiluminescence (ECL) method. The original Western blot images were shown in Supplementary Fig. 27.

### Extraction and culture of primary cells

Peritoneal macrophages were collected from DSS-induced UC mice via lavage from the peritoneal cavity using 5 mL ice-cold PBS^72^. Peritoneal macrophages were cultured at 37 °C under 5% CO_2_ in RPMI-1640 supplemented with 10% foetal bovine serum (FBS, Gibco, MA, USA).

Mononuclear cells were isolated from the peripheral blood and spleen using a mouse peripheral blood mononuclear cell separation kit (TBD Science, LDS1090, Tianjin, China) and a mouse splenic mononuclear extraction kit (TBD Science, LDS1090PK, Tianjin, China), respectively. Lamina propria mononuclear cells were isolated using a method described previously^73^. Briefly, the colon was removed from the sacrificed mice, cut into 0.5-cm pieces and washed thoroughly with cold PBS to remove all debris and blood. After incubating with 2 mM dithiothreitol and 1 mM EDTA in PBS at 37 °C for 2 × 20 min under gentle shaking to remove intestinal epithelial cells, the tissues were digested in 10 mL 2% FBS-RPMI-Collagenase A (1 mg/mL, Roche, Mannheim, Germany) at 37 °C for 30 min. Lamina propria cells were collected and further purified via density gradient centrifugation with 40% and 70% Percoll–RPMI solution. Lamina propria mononuclear cells were collected from the interphase.

Monocytes were isolated from peripheral blood mononuclear cells using a peripheral blood monocyte extraction kit (TBD Science, TBD2011 M, Tianjin, China). Splenic macrophages and colonic macrophages were isolated using anti-F4/80 MicroBeads UltraPure (Miltenyi Biotec, 130-110-443, Bergisch Gladbach, Germany) from spleen mononuclear cells and lamina propria mononuclear cells.

Peripheral blood lymphocytes and spleen lymphocytes were enriched using a mouse peripheral blood lymphocyte separation kit (TBD Science, LTS1092, Tianjin, China) and a mouse splenic lymphocyte extraction kit (TBD Science, LTS1092PK, Tianjin, China). CD4^+^ T cells were obtained from peripheral blood lymphocytes, spleen lymphocytes and lamina propria mononuclear cells using a CD4^+^ T-cell Isolation kit (Miltenyi Biotec, 130-104-454, Bergisch Gladbach, Germany).

Primary hepatocytes were isolated according to previous studies^74^. The mice were anaesthetised using sodium pentobarbital before exposing the hepatic portal veins, which were washed to remove residual blood and perfused with collagenase (A004186-0001, Sangon Biotech, Shanghai, China) for approximately 10 min. The livers were immediately moved to a sterile 10-cm cell culture dish for mincing before the hepatocytes were dispersed, and the hepatocytes were filtered through a 70-mm membrane to remove tissue debris. After washing twice with cold DMEM and centrifuging at 100 × *g* for 4 min at 4 °C, the isolated hepatocytes were seeded at a density of 1 × 10^7^ cells per dish in 6-cm dishes in DMEM with 10% FBS. All primary cell extraction steps were performed under aseptic conditions, and the viability of the primary cells was determined using trypan blue staining and cell counting.

### Evaluation of in vivo accumulation of original circuits

The plasmid DNA was extracted from the liver, spleen and colon (~100 mg) using a plasmid extraction kit (TIANGEN Biotech, DP104-02, Beijing, China). The synthetic CMV-siR^TNF-α^ circuit (in the form of DNA plasmid) was serially diluted over several orders of magnitude, from 10^-1 ^µg/µL to 10^-11 ^µg/µL, and was assessed using quantitative PCR. The resulting C_T_ values were plotted against the amount of input CMV-siR^TNF-α^ circuit to generate a standard curve. The C_T_ values of the CMV-siR^TNF-α^ circuit in the liver, spleen and colon were measured. The absolute amounts of CMV-siR^TNF-α^ circuit at different time points were calculated from the standard curve.

### Quantitative RT-PCR assay

TRIzol Reagent (Invitrogen, CA, USA) was used to extract total RNA from cultured cells or mouse tissues according to the manufacturer’s instructions. siRNAs were quantified using TaqMan miRNA probes (Applied Biosystems, CA, USA) according to the manufacturer’s instructions. Briefly, 1 µg of total RNA was reverse-transcribed into cDNA using AMV reverse transcriptase (TaKaRa, Dalian, China) and a stem-loop RT primer (Applied Biosystems, CA, USA). The following reaction conditions were used: 16 °C for 30 min, 42 °C for 30 min, and 85 °C for 5 min. Real-time PCR was performed using a TaqMan™ MicroRNA Assay (Applied Biosystems, 4440887, CA, USA) and a LightCycler 96 System (Roche, Mannheim, Germany). The reactions were incubated in a 96-well optical plate at 95 °C for 10 min followed by 40 cycles at 95 °C for 15 s and 60 °C for 1 min.

For quantification of the absolute levels of TNF-α siRNA, synthetic single-stranded TNF-α siRNA was serially diluted and assessed using quantitative RT-PCR to generate a standard curve, and a no-template control was assessed simultaneously to determine the specificity of the primer set. By referring to the standard curve, the concentration of TNF-α siRNA in various tissues was calculated and presented as the absolute amounts of TNF-α siRNA in 1 g of total RNA isolated from each mouse tissue (pmol/g total RNA). For quantification of the relative amounts of siRNA, the siRNA levels in the cells and tissues were normalised to U6 snRNA, while in plasma and sEVs, the siRNA levels were normalised to endogenous miR-16.

For quantification of mRNAs, 1 μg of total RNA was reverse transcribed into cDNA using oligo(dT). Then, specific forward primers and reverse primers were used to amplify TNF-α mRNA, integrin α4 mRNA, B7-1 mRNA and GAPDH mRNA. Real-time PCR was incubated in a 96-well optical plate at 95 °C for 5 min, followed by 40 cycles of 95 °C for 30 s, 60 °C for 30 s and 72 °C for 1 min.

All reactions were run in triplicate. After the reactions were complete, the cycle threshold (C_T_) values were determined using fixed threshold settings. All sequences of the primers can be found in Supplementary Table 3.

### Determination of the distribution of siRNA in sEV and sEV-free fractions of mouse plasma

Plasma was collected from each mouse and centrifuged at 2000 × *g* for 20 min and then at 10,000 × *g* for 20 min to remove cells and debris. Approximately 200 μL of plasma was obtained from each mouse and was equally divided into two samples, one (~100 μL) for direct isolation of total RNA and quantification of siRNA levels, and the other for purification of sEVs. Plasma sEVs were isolated using the Total Exosome Isolation (from plasma) kit (Invitrogen) according to manufacturer’s instructions. In detail, equal volume (~100 μL) of PBS was added to the plasma followed by 0.2 total volume (~40 μL) of the Exosome Precipitation Reagent. After 10 min’ incubation of the sample at room temperature, sEVs and supernatant can be separated by centrifugation at 10,000 × *g* for 5 min at room temperature. TRIzol reagent was added to the sEV pellet for isolation of total RNA. For sEV-free supernatant (~220 μL), 800 μL TRIzol reagent was added to extract total RNA. Because sEV fraction and sEV-free plasma fraction are from the same plasma sample, the levels of siRNA in total plasma, sEVs and sEV-free plasma are comparable through a simple conversion process.

### In situ hybridisation of siRNA

In situ hybridisations were performed using 15-μm cryosections from formaldehyde-fixed mouse tissues^75^. Sections were prehybridised in hybridisation solution (50% formamide, 5 × SSC, 0.5 mg/mL yeast tRNA, 1 × Denhardt’s solution) at 25 °C below the predicted Tm value of the LNA probe for 30 min. Probes (3 pmol) (miRCURY LNA miRNA detection probe; Qiagen, Hilden, Germany) were DIG-labelled and hybridised to the sections for 10 h at the same temperature as in the prehybridisation. The slides were washed in 0.1 × SSC 3 times at 60 °C once for 5 min in 2 × SSC at room temperature with agitation. After blocking, anti-digoxigenin (Roche, 11207741910, Mannheim, Germany) was added to the section slides. After washing again, the slides were mounted in Prolong Gold containing DAPI (ProLong™ Gold Antifade Mountant with DAPI; Invitrogen, CA, USA).

### DAI

The DAI used to evaluate intestinal inflammation was based on a previously published grading system^71^. The scores ranged from 0 to 4 based on the following parameters: weight loss (0, <1%; 1, 1–5%; 2, 6–10%; 3, 11–18%; and 4, >18%), stool consistency (0, normal; 1, soft but still formed; 2, soft; 3, very soft and wet; and 4, watery diarrhoea) and blood (0 and 1, negative hemoccult; 2, positive hemoccult; 3, blood traces in stool visible; and 4, gross rectal bleeding) (Nanjing Jiancheng Bioengineering Institute, Urine faecal occult blood test kit, C027-1-1, Nanjing, China). The combined scores were then averaged to obtain the final DAI score.

### Cytokine and MPO analysis

For cytokine determination and MPO activity measurement in the colons, protein extracts were prepared by homogenising colonic segments (50 mg of tissue per mL) in 50 mmol/L Tris-HCl (pH 7.4)/0.5 mmol/L DTT/10 mg/mL with a Protease Inhibitor Cocktail (Sigma, P8340-1ML, Darmstadt, Germany). Samples were centrifuged at 1500 × *g* for 20 min and stored at −80 °C until analysis. The concentrations of TNF-α, IL-6, IL-12p70, IL-17A and IL-23 in colons were evaluated using ELISA kits (MultiSciences, 70-EK282/3-96, 70-EK206/3-96, 70-EK212/3-96, 70-EK217/2-96, 70-EK223-96, Hangzhou, China)^76^. MPO activity was determined using a kit from Jiancheng Biotech (Nanjing, China).

### Immunofluorescence staining and H&E staining

Cryosections were fixed with 4% paraformaldehyde and blocked with 5% BSA. They were then incubated with TNF-α (Abcam, ab183218, Cambridge, UK), B7-1 (Abcam, ab254579, Cambridge, UK), integrin α4 (Cell Signalling Technology, 8440S, Danvers, MA, USA), F4/80 (Abcam, ab60343, Cambridge, UK) or CD4 (Proteintech, CL488-65141) antibodies and then washed and incubated with goat anti-rabbit IgG (H + L) and CoraLite594-conjugated Goat Anti-Rabbit IgG(H + L) (Proteintech, SA00013-4, Wuhan, China). Nuclei were counterstained with DAPI (ProLong™ Gold Antifade Mountant with DAPI; Invitrogen, CA, USA), and samples were analysed by fluorescence confocal microscopy (LSM SP8, Leica).

For histopathological examination, the obtained colonic samples were fixed in Bouis buffer, embedded in paraffin, sectioned and stained with haematoxylin and eosin (H&E). Inflammation of DSS-induced UC was scored in a blinded manner from 0 to 3 as follows: tissue damage (0, no signs of inflammation; 1 isolated focal epithelial damage; 2 mucosal erosions and ulcerations; 3 extensive damage deep into the bowel wall) and lamina propria inflammatory cell infiltration (0, infrequent; 1, increased, some neutrophils; 2, submucosal presence of inflammatory cell clusters; 3, transmural cell infiltrations). The combined scores were then averaged to obtain the final histopathological scores^71^. Inflammation of TNBS-induced colitis was also scored in a blinded manner from 0 to 4 as follows: 0, no signs of inflammation; 1, low leucocyte infiltration; 2, moderate leucocyte infiltration; 3, high leucocyte infiltration, moderate fibrosis, high vascular density, thickening of the colon wall, moderate goblet cell loss, and focal loss of crypts; and 4, transmural infiltration, massive loss of goblet cells, extensive fibrosis, and diffuse loss of crypts^71^.

### Flow cytometry

For analysis of colonic macrophage polarisation, colonic tissue was processed as described previously. The antibodies used were obtained from Biolegend (San Diego, California) and included anti-CD11b-PerCP (clone M1/70), αCD64-PE (clone X54-5/7.1), αCD206-AF488 (clone C068C2) and anti-Ly6C-APC (clone HK1.4). Colonic macrophages were defined as CD11b^+^CD64^+^. The gating strategy was shown in Supplementary Fig. 28a.

A Mouse Th1 Staining Kit (MultiSciences, 70-KTH201-100, Hangzhou, China) and a Mouse Th17 Staining Kit (MultiSciences, 70-KTH217-25, Hangzhou, China) were used to determine the percentage of Th1 or Th17 cells in colonic lamina propria mononuclear cells. According to the manufacturer’s instructions, lamina propria mononuclear cells were stimulated with 1 μL PMA/ionomycin mixture (250 ×) and 1 μL BFA/monensin mixture (250×) for 5 h. Lamina propria mononuclear cells were surface-stained with anti-mouse CD4-PerCP-Cy5.5 and anti-mouse IFN-γ-PE for Th1 staining or anti-mouse CD4-PerCP-Cy5.5 and anti-mouse IL-17A-PE for Th17 staining. Gating strategy to obtain CD4^+^ T cells was shown in Supplementary Fig. 28b. Cells were analysed using flow cytometry on a FACScan flow cytometer and FlowJo 10 software (Oregon, USA).

To assess therapeutic effect of CMV-siR^T+B+I^, cells were stained with fluorochrome-conjugated antibodies against F4/80 (11-4801-81, Invitrogen, CA, USA), B7-1 (ab93507, Abcam, Cambridge, UK), integrin α4 (ab202969, Abcam, Cambridge, UK) and CD25 (ab86908, Abcam, Cambridge, UK). The gating strategy to obtain F4/80^+^ macrophages was shown in Supplementary Fig. 28c. Isotype-matched antibodies of irrelevant specificity were used to determine the level of nonspecific staining and frequency of cells stained with test antibodies.

### E-Cadherin adhesion assay

Wells were coated with E-cadherin (5 μg/mL, R*&*D Systems, MN, USA) in 150 mM NaCl with 20 mM HEPES at 37 °C overnight, followed by blocking with 5% BSA at 37 °C for 2 h. A total of 50000 purified CD4^+^ cells in adhesion buffer were added for 90 min at 37 °C. After washing, adherent cells were counterstained with DAPI. Finally, the slides were analysed by fluorescence and confocal microscopy, and adherent cells in at least three high-power fields per condition were counted^41^.

### Analyses of serum biochemical indicators and tissue damage

Chronic UC was induced in male BALB/c mice by rhythmically administering to mice 2.5% DSS for 1 week and water for 2 weeks and the cycle was repeated for 3 times. Four days after each DSS drinking, mice were intravenously injected with PBS, 20 mg/kg CMV-scrR, CMV-siR^TNF-α^ or CMV-siR^T+B+I^ circuit for a total of 3 times, once every 2 days. Twelve hours after the last injection, the mice were euthanised to collect peripheral blood and tissues. Liver and kidney specimens were fixed in 4% PFA for H&E staining to evaluate tissue damage. Fresh peripheral blood was subjected to separation of serum and measurement of representative serum biochemical indicators, including alanine aminotransferase (ALT), aspartate aminotransferase (AST), total bilirubin (TBIL), blood urea nitrogen (BUN), alkaline phosphatase (ALP) and creatinine (CREA).

### Statistics and reproducibility

Details on the statistics used can be found in the figure legends. All statistical analyses were performed using commercially available software (GraphPad Prism 9) (GraphPad Prism Inc., San Diego, CA, USA). Statistical significance was determined by two-tailed unpaired Student’s *t* test. Differences among groups were compared by one-way ANOVA, and multiple comparisons were conducted by Dunnett’s test. All box and whisker plots denote median + quartiles (box) and range (whiskers). *N* represents the number of samples used in the experiments. Data are means with error bars showing the SEM. Significance was assumed at *p < 0.05; **p < 0.01; ***p < 0.005. All micrographs and blots were representative of three independent experiments.

### Reporting summary

Further information on research design is available in the Nature Research Reporting Summary linked to this article.

## Supplementary information

Supplementary Information

Peer Review File

Reporting Summary

## Data Availability

All relevant data generated for this study are included in the article/Supplementary Material/Source Data File. Other data/materials that support the findings of this study are readily available from the corresponding author upon reasonable request. Source data are provided with this paper.

## Source data

Source Data
