# Supplementary material for: In vivo self-assembled siRNA as a modality for combination therapy of ulcerative colitis
Source: Nat Commun. 2022 Sep 28;13:5700. doi: 10.1038/s41467-022-33436-0 (PMC9519883; doi:10.1038/s41467-022-33436-0)
Supplement: Supplementary file 1 — Supplementary Information [file 41467_2022_33436_MOESM1_ESM.pdf]

Supplementary information for

In vivo self-assembled siRNA as a modality for combination therapy of ulcerative colitis

(Xinyan Zhou *et al.*)

This file includes:

Supplementary Figure 1 to Supplementary Figure 28

Supplementary Table 1 to Supplementary Table 3

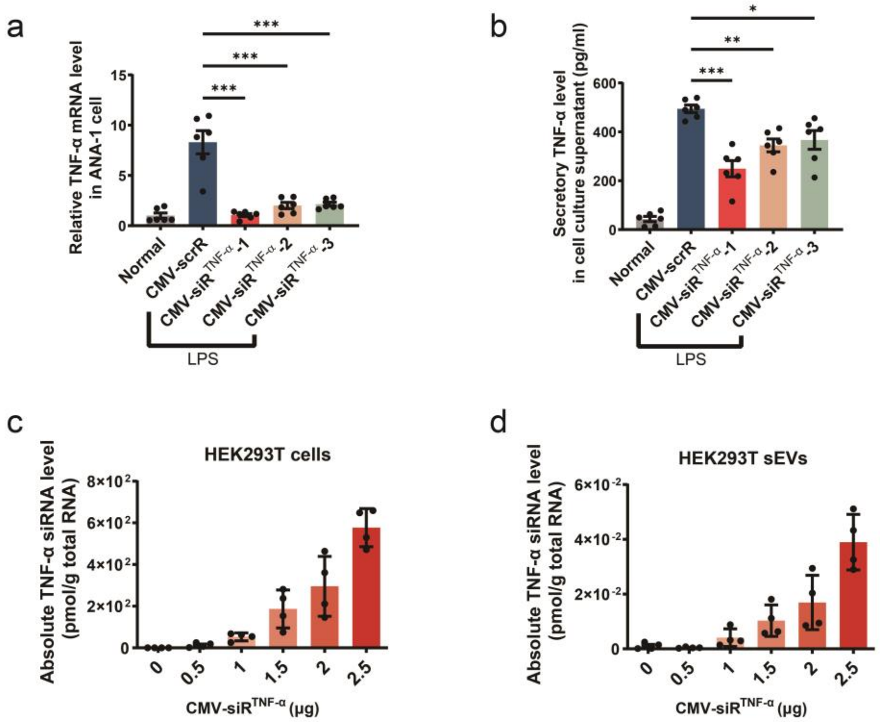

Supplementary Figure 1. Characterisation of the genetic circuits *in vitro*. (a) ANA-1 cells were transfected with CMV-scrR or three CMV-siR$^{TNF-\alpha}$ circuits. At 24 hours posttransfection, ANA-1 cells were treated with LPS to stimulate an inflammatory response, and quantitative RT-PCR analysis was performed to measure TNF-α mRNA levels at 48 hours posttransfection (n = 6 in each group). Untreated cells were used as normal controls. **(b)** Determination of the levels of secretory TNF-α protein in cell culture supernatant by ELISA (n = 6 in each group). **(c)** Quantitative RT-PCR analysis of TNF-α siRNA levels in HEK293T cells transfected with increasing dose of CMV-siR$^{TNF-\alpha}$ circuit (n = 4 in each group). **(d)** Quantitative RT-PCR analysis of TNF-α siRNA levels in the sEVs derived from the culture medium of HEK293T cells transfected with increasing dose of CMV-siR$^{TNF-\alpha}$ circuit (n = 4 in each group). Values are presented as the mean ± SEM. Significance was determined using one-way ANOVA followed by Dunnett's multiple comparison. * $p < 0.05$; ** $p < 0.01$; *** $p < 0.005$.

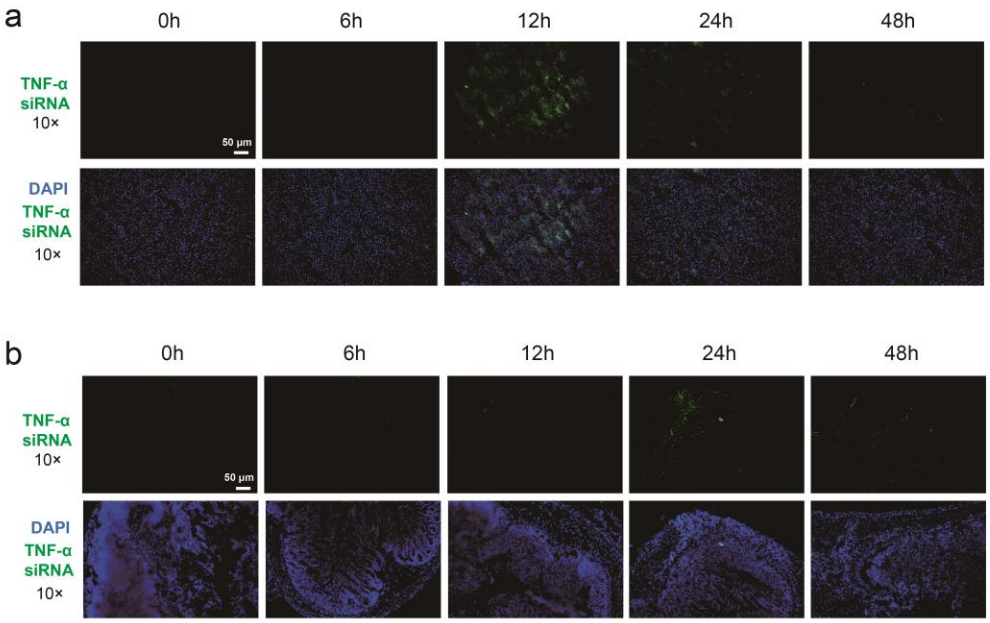

Supplementary Figure 2. Tracking and visualisation of TNF-α siRNA *in vivo*. (a and b) *In situ* detection of TNF-α siRNA in liver (a) and colon (b) sections of DSS mice at 0, 6, 12, 24 or 48 hours after injection with the CMV-siR$^{TNF\text{-}\alpha}$ circuit. Positive *in situ* hybridisation signals are shown in green, and DAPI-stained nuclei are shown in blue. Scale bar: 50 μm. Each *in situ* hybridisation was repeated independently three times, and representative images are shown.

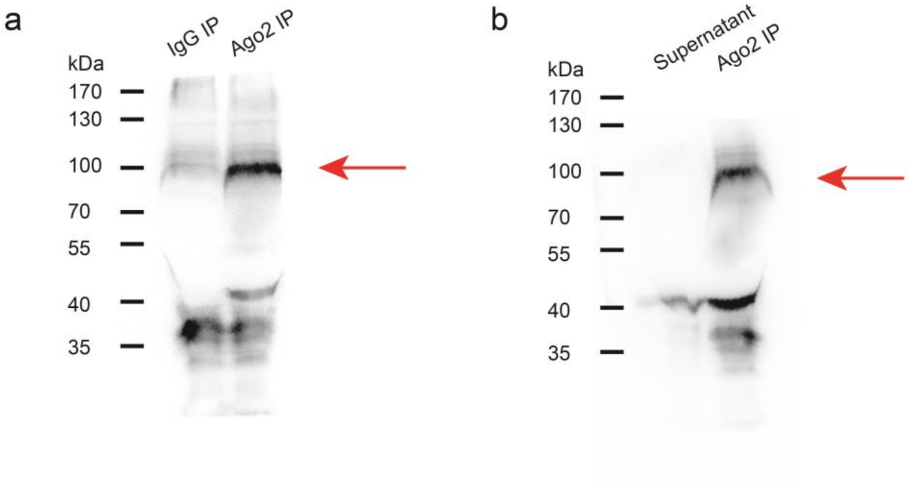

Supplementary Figure 3. Western blots probed with an anti-Ago2 antibody to assess the Ago2 associated with the captured beads following Ago2 immunoprecipitation. Bead-conjugated anti-Ago2 antibody was incubated with the plasma of CMV-scrR circuit- or CMV-siR$^{TNF-\alpha}$ circuit-injected mice under native conditions. The beads were separated by centrifuging and processed for protein extraction and subsequent Western blotting using a rabbit anti-Ago2 antibody. Total protein extracted from the supernatant was also subjected to Western blot analysis to determine the amounts of Ago2. The IgG antibody was used as a negative control. **(a)** Red arrow indicating an Ago2 band at ~97 kDa in the Ago2 immunoprecipitates but not in the IgG immunoprecipitates. **(b)** Red arrow indicating an Ago2 band at ~97 kDa in the Ago2 immunoprecipitates but not in the supernatant. Each immunoprecipitation was repeated independently three times, and representative images are shown.

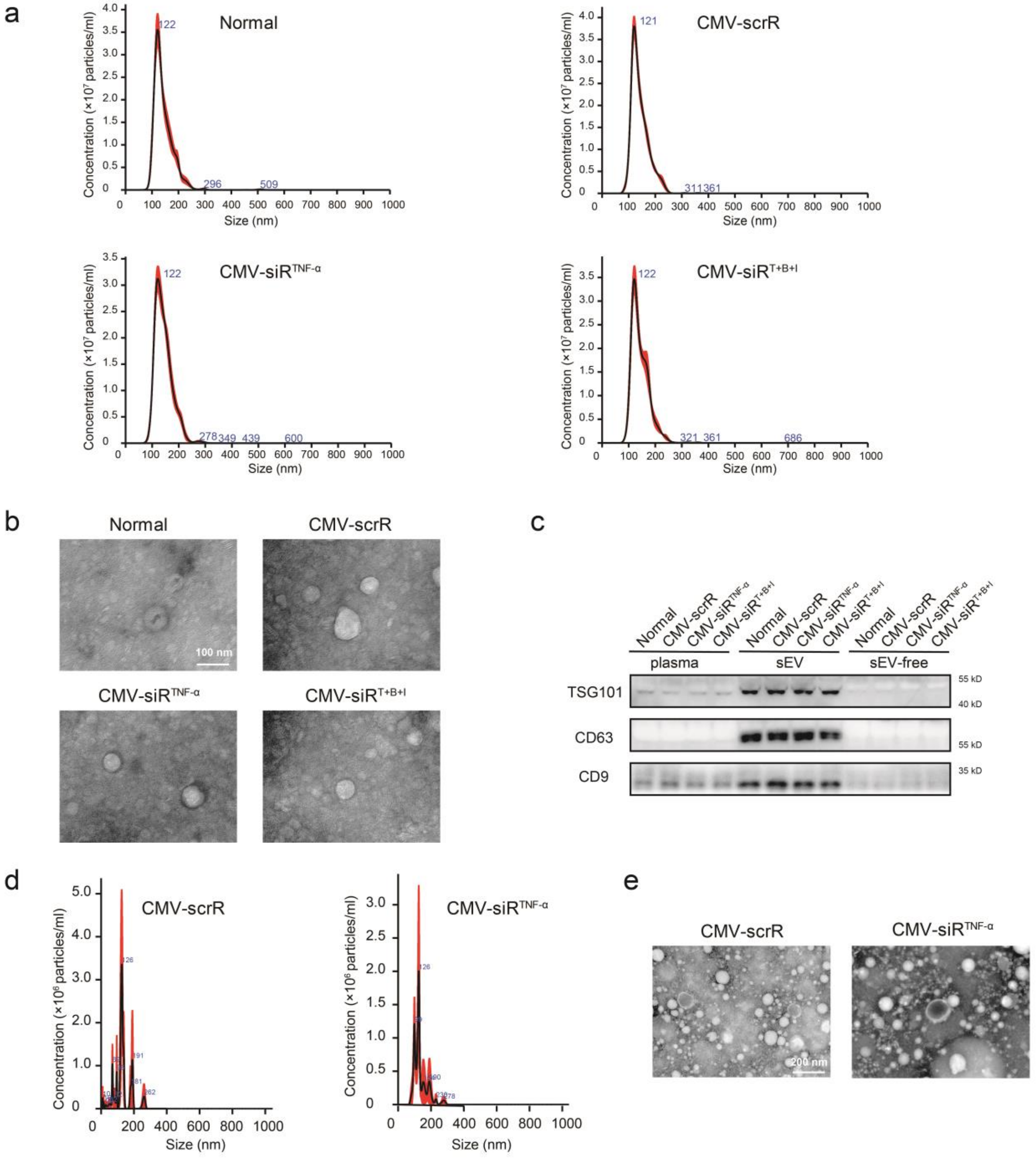

Supplementary Figure 4. Characterisation of the properties of siRNA-encapsulating sEVs. BALB/c mice were intravenously injected with 5 mg/kg CMV-scrR, CMV-siR$^{TNF-α}$ or CMV-siR^{T+B+I} circuit every day for a total of 7 times, and sEVs were then purified by using commercially available kit (a-c) or density gradient centrifugation (d and e) from mouse plasma and characterised using NTA and TEM. The enrichment of sEV markers was analysed by Western blotting. sEVs derived from untreated BALB/c mice were included as normal controls. **(a)** Size distribution and

concentration of purified sEVs determined by NTA. **(b)** Representative TEM images of sEVs. Scale bar: 100 nm. **(c)** Western blot analysis of specific sEV markers (TSG101, CD63 and CD9) in whole plasma, purified sEVs and sEV-free plasma. An equal amount of total protein was loaded in each lane. **(d)** Size distribution and concentration of purified sEVs determined by NTA. **(e)** Representative TEM images of sEVs. Scale bar: 100 nm. Each experiment was repeated independently three times, and representative results are shown.

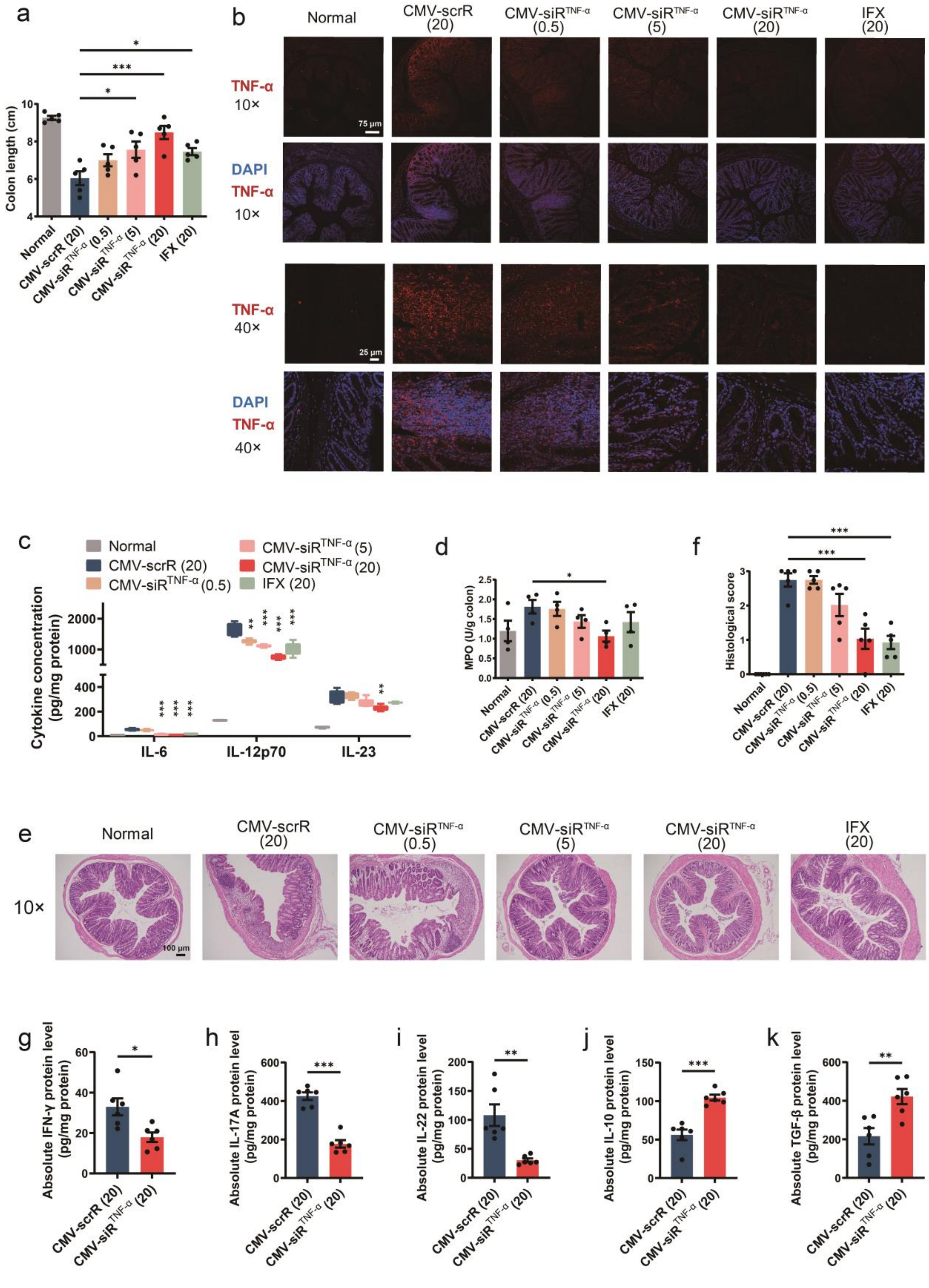
a
Colon length (cm)
Normal
CMV-scrR (20)
CMV-siR TNF-α (0.5)
CMV-siR TNF-α (5)
CMV-siR TNF-α (20)
IFX (20)
b
Normal
CMV-scrR (20)
CMV-siRTNF-α (0.5)
CMV-siRTNF-α (5)
CMV-siRTNF-α (20)
IFX (20)
TNF-α 10×
DAPI TNF-α 10×
TNF-α 40×
DAPI TNF-α 40×
75 μm
25 μm
c
Normal
CMV-scrR (20)
CMV-siRTNF-α (0.5)
CMV-siRTNF-α (5)
CMV-siRTNF-α (20)
IFX (20)
Cytokine concentration (pg/mg protein)
IL-6
IL-12p70
IL-23
d
MPO (U/g colon)
f
Histological score
e
Normal
CMV-scrR (20)
CMV-siRTNF-α (0.5)
CMV-siRTNF-α (5)
CMV-siRTNF-α (20)
IFX (20)
10×
100 μm
g
Absolute IFN-γ protein level (pg/mg protein)
h
Absolute IL-17A protein level (pg/mg protein)
i
Absolute IL-22 protein level (pg/mg protein)
j
Absolute IL-10 protein level (pg/mg protein)
k
Absolute TGF-β protein level (pg/mg protein)
CMV-scrR (20)
CMV-siR TNF-α (20)

Supplementary Figure 5. Intravenous injection of the CMV-siR$^{TNF\text{-}\alpha}$ circuit protects mice from DSS-induced acute UC. **(a)** Mean colon length (n = 5 in each group). **(b)** Immunofluorescence staining of TNF-α (red) and DAPI (blue) in colon sections. Scale bar: 75 μm (10 ×) or 25 μm (40 ×). **(c)** Determination of serum levels of IL-6, IL-12p70 and IL-23 by ELISA (n = 4 in normal group; n = 5 in CMV-scrR (20), CMV-siR$^{TNF\text{-}\alpha}$ (0.5), CMV-siR$^{TNF\text{-}\alpha}$ (5), CMV-siR$^{TNF\text{-}\alpha}$ (20) and IFX (20) groups). **(d)** Colonic MPO activity (n = 4 in each group). **(e)** Representative images of H&E staining of colon sections. Scale bar: 100 μm. **(f)** Histological scores of colon sections (n = 5 in each group). **(g–k)** Determination of the levels of IFN-γ, IL-17A, IL-22, IL-10 and TGF-β1 in the colon by ELISA (n = 6 in each group). Values are presented as the mean ± SEM. Significance was determined using one-way ANOVA followed by Dunnett's multiple comparison. * $p < 0.05$; ** $p < 0.01$; *** $p < 0.005$.

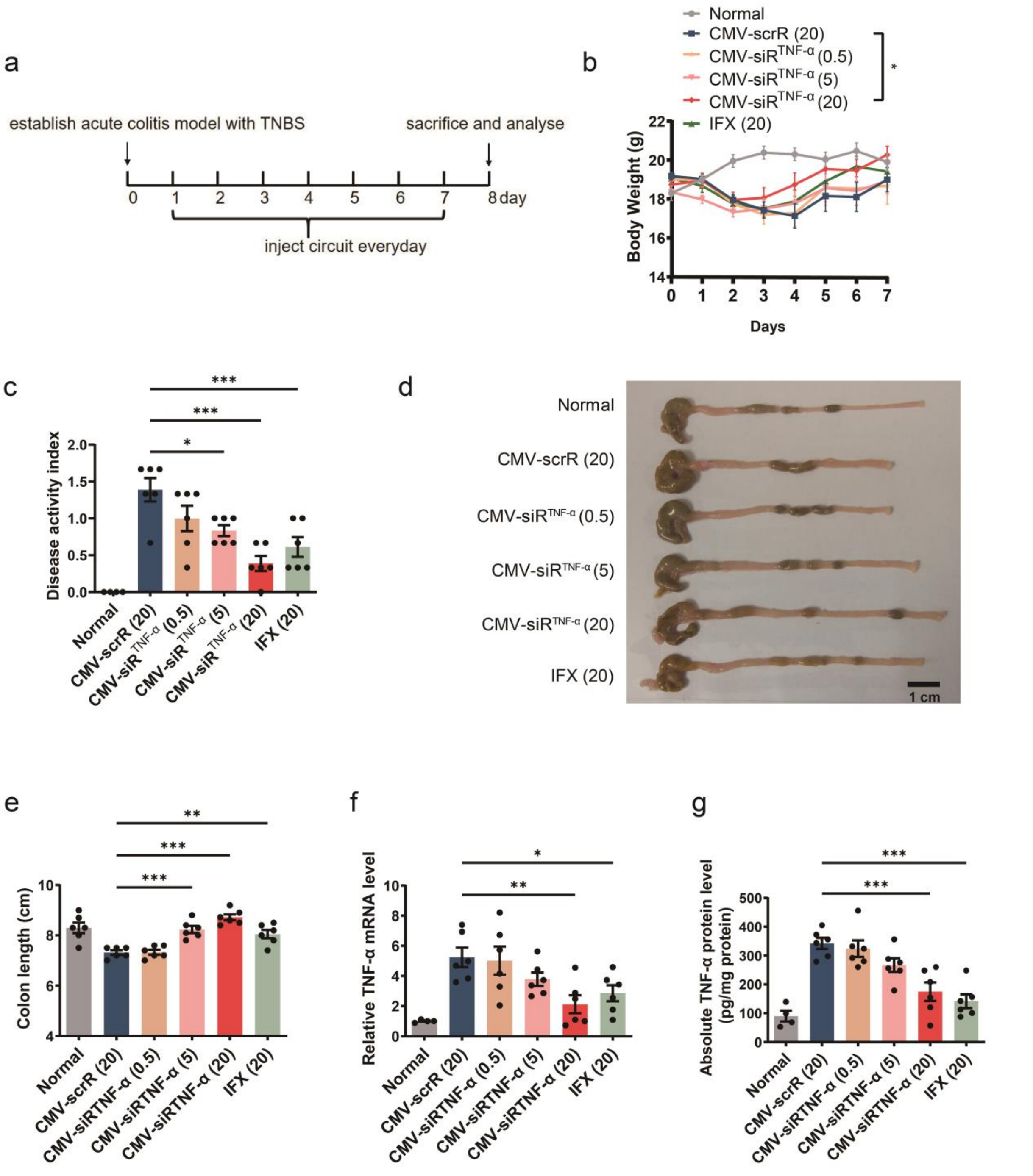

Supplementary Figure 6. Intravenous injection of the CMV-siR$^{TNF\text{-}\alpha}$ circuit protects mice from a TNBS-induced acute colitis model. (a) Flow chart of the experimental design. Acute colitis was induced by intracolonic administration of 2.5% TNBS into BALB/c mice. One day later, mice were intravenously injected with 20 mg/kg CMV-scrR or three dosages (0.5, 5 and 20 mg/kg)

of CMV-siR$^{TNF\text{-}\alpha}$ circuit or 20 mg/kg infliximab (IFX) once a day. After 7 injections, the mice were euthanised. Body weights were monitored daily, and symptoms and histology were evaluated on day 8. Untreated BALB/c mice were included as normal controls. **(b)** Body weight curves (n = 6 in each group). **(c)** DAI scores (n = 6 in each group). **(d)** Representative macroscopic features of colons. Scale bar: 1 cm. **(e)** Mean colon length (n = 6 in each group). **(f)** Quantitative RT-PCR analysis of the relative expression levels of TNF-α mRNA in the colon (n = 6 in each group). **(g)** Determination of the absolute expression levels of TNF-α protein in the colon by ELISA (n = 6 in each group). Values are presented as the mean ± SEM. Significance was determined using one-way ANOVA followed by Dunnett's multiple comparison in panels c, e, f and g or two-way ANOVA followed by Dunnett's multiple comparison in panel b. * $p < 0.05$; ** $p < 0.01$; *** $p < 0.005$.

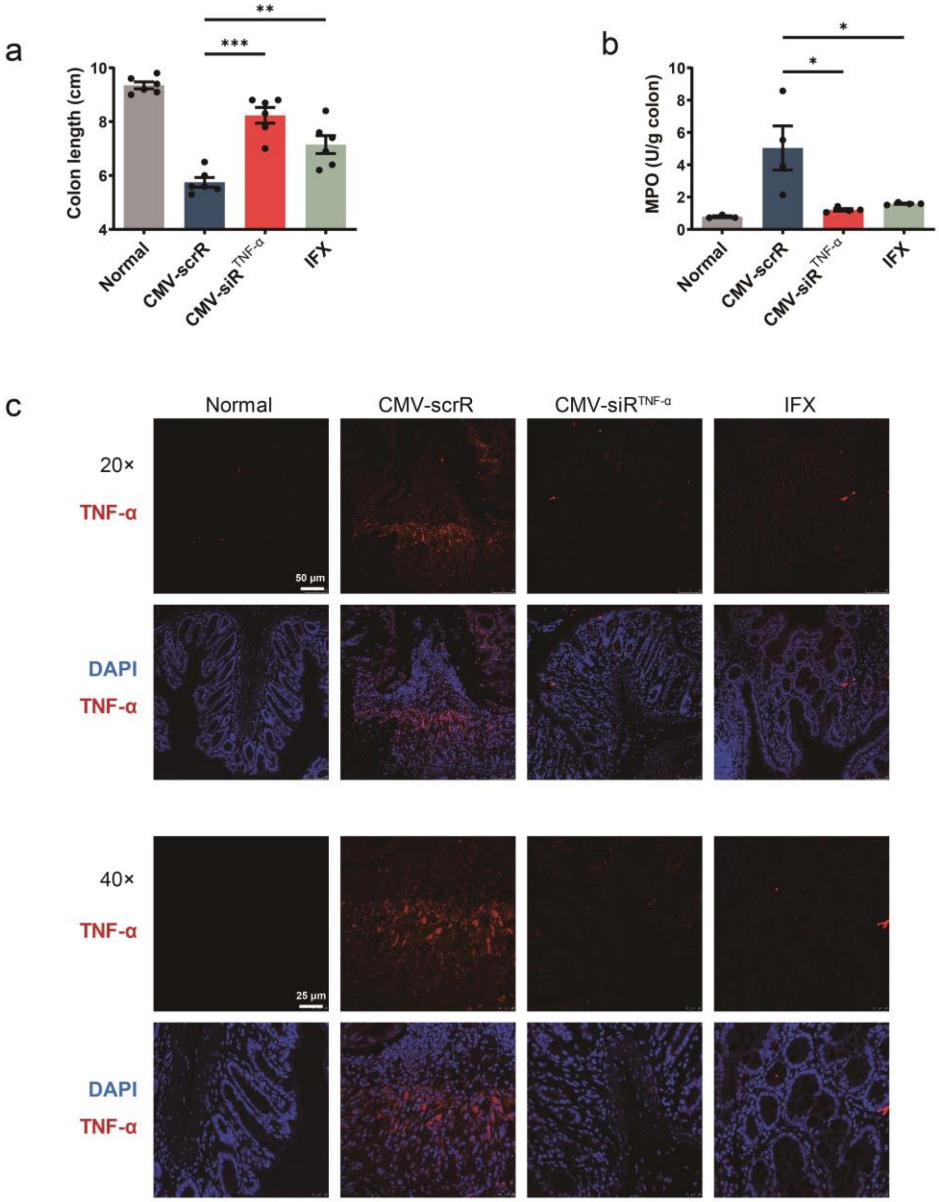

Supplementary Figure 7. Intravenous injection of the CMV-siR$^{TNF\text{-}\alpha}$ circuit protects mice from DSS-induced chronic UC. (a) Mean colon length (n = 6 in each group). **(b)** Colonic MPO activity (n = 4 in each group). **(c)** Immunofluorescence staining of TNF-α (red) and DAPI (blue) in colon sections. Scale bar: 50 μm (20 ×) or 25 μm (40 ×). Values are presented as the mean ± SEM. Significance was determined using one-way ANOVA followed by Dunnett's multiple comparison. * $p < 0.05$; ** $p < 0.01$; *** $p < 0.005$.

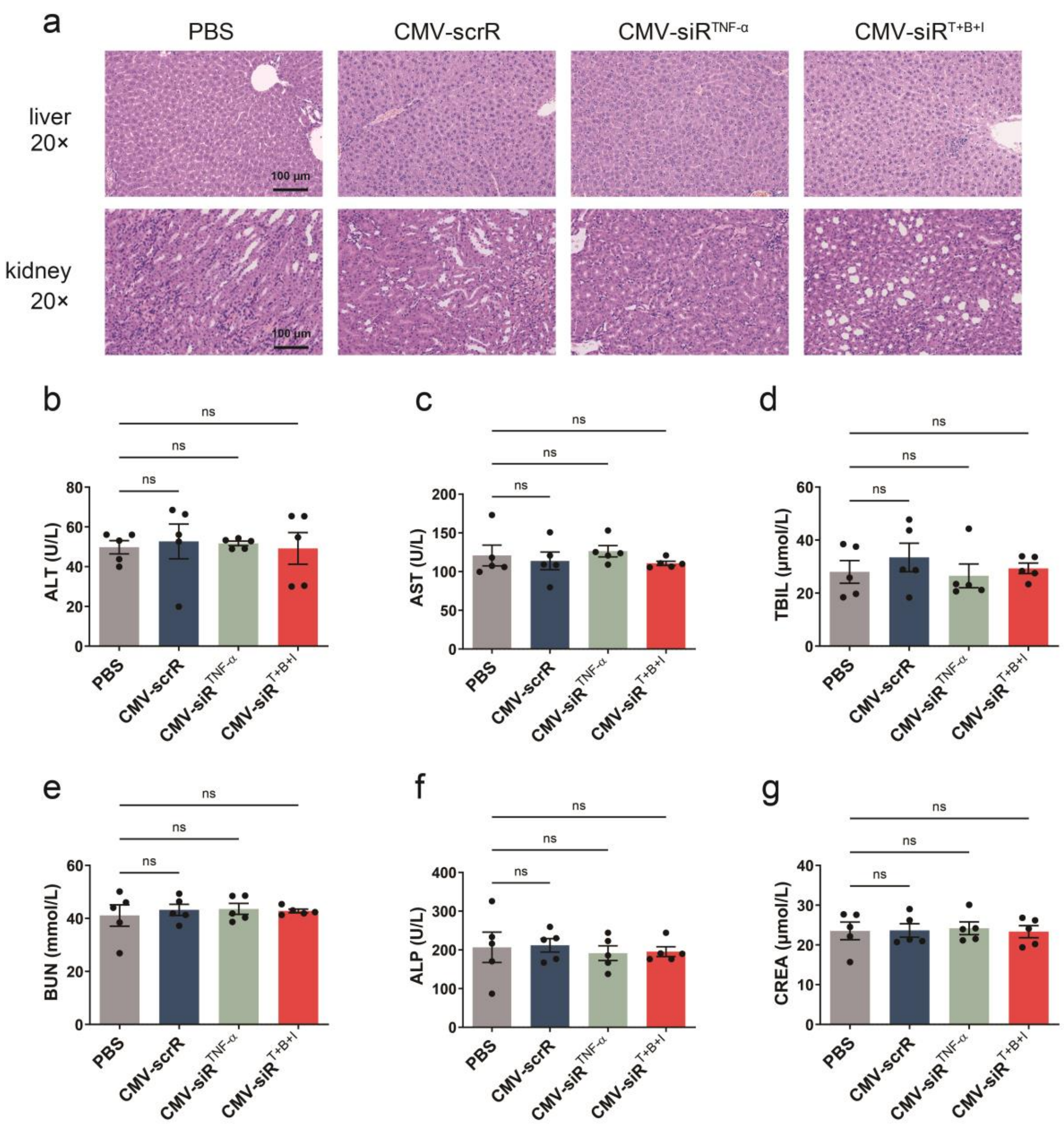

Supplementary Figure 8. Evaluation of the toxic effects and tissue damage in mice after intravenous injection of the genetic circuits. Chronic UC was induced in male BALB/c mice by rhythmically administering to mice 2.5% DSS for 1 week and water for 2 weeks and the cycle was repeated for 3 times. Four days after each DSS drinking, mice were intravenously injected with PBS or with equal dose (20 mg/kg) of CMV-scrR, CMV-siR$^{TNF-\alpha}$ or CMV-siR^{T+B+I} circuit for a total of 3 times, once every 2 days. Twelve hours after the last injection, mice were sacrificed, and blood and tissue samples were collected and analysed for serum biochemical indicators and tissue damage. **(a)** Histological examination of the liver and kidney. Scale bar: 100 μm. **(b-g)**

Measurement of representative serum biochemical indicators, including alanine aminotransferase (ALT), aspartate aminotransferase (AST), total bilirubin (TBIL), blood urea nitrogen (BUN), alkaline phosphatase (ALP) and creatinine (CREA), in the serum (n = 5 in each group). Values are presented as the mean ± SEM. Significance was determined using one-way ANOVA followed by Dunnett's multiple comparison. NS, not significant.

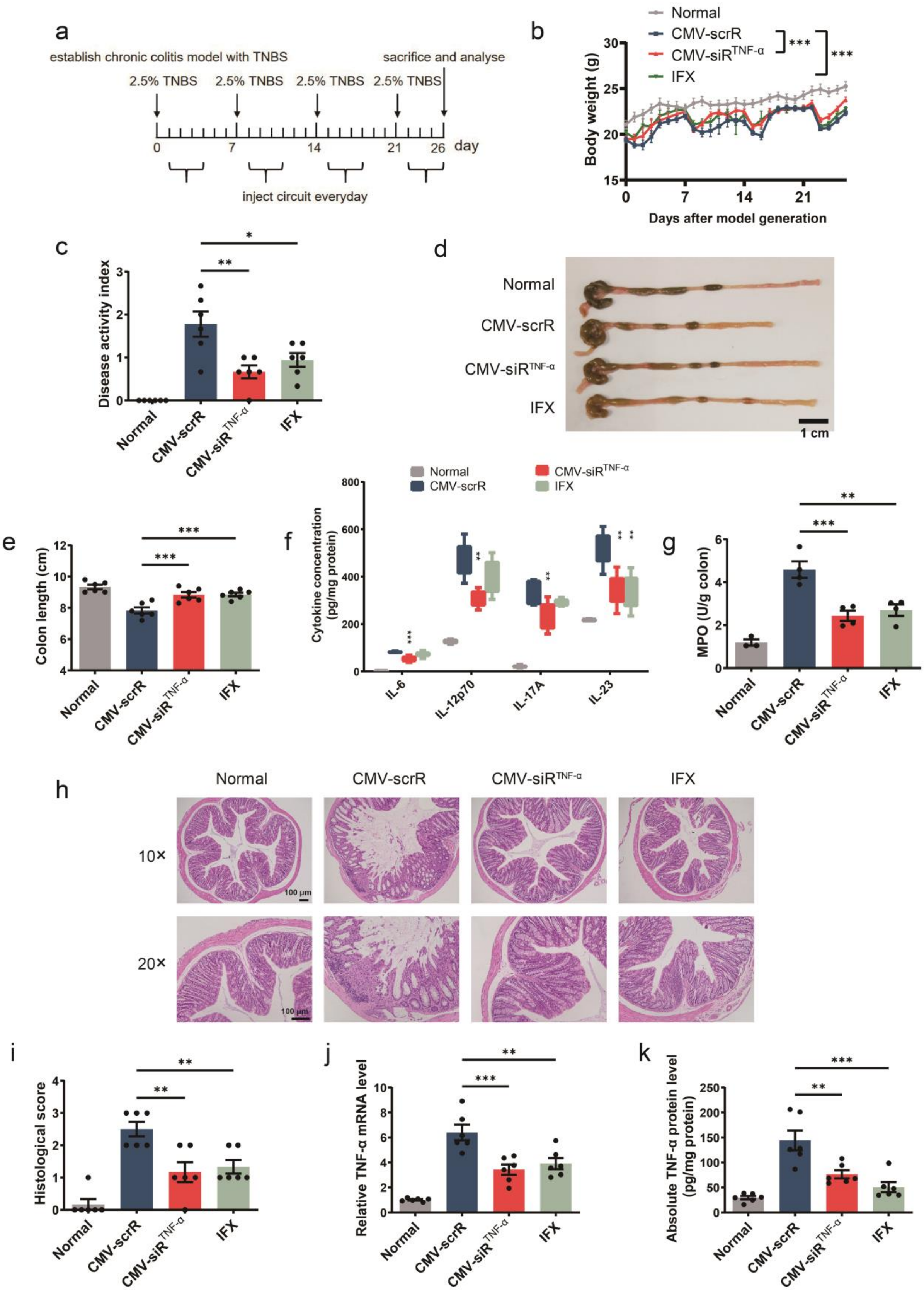
a
establish chronic colitis model with TNBS
sacrifice and analyse
2.5% TNBS
0
7
14
21
26
day
inject circuit everyday
b
Normal
CMV-scrR
CMV-siR$^{TNF-\alpha}$
IFX
Body weight (g)
Days after model generation
c
Disease activity index
d
Normal
CMV-scrR
CMV-siR$^{TNF-\alpha}$
IFX
1 cm
e
Colon length (cm)
f
Cytokine concentration (pg/mg protein)
IL-6
IL-12p70
IL-17A
IL-23
g
MPO (U/g colon)
h
10×
20×
100 μm
i
Histological score
j
Relative TNF-α mRNA level
k
Absolute TNF-α protein level (pg/mg protein)

Supplementary Figure 9. Intravenous injection of the CMV-siR$^{TNF-\alpha}$ circuit protects mice in the TNBS-induced chronic colitis model. **(a)** Flow chart of the experimental design. Chronic colitis was induced by rhythmic administration (once a week) of 2.5% TNBS into BALB/c mice for 4 times. After each TNBS administration, mice were intravenously injected with 20 mg/kg CMV-scrR, 20 mg/kg CMV-siR^{TNF} circuit or 20 mg/kg infliximab for a total of 4 times over the first 4 days. Body weights were monitored daily, and symptoms and histology were evaluated on day 26. Untreated BALB/c mice were used as normal controls. **(b)** Body weight curves (n = 6 in each group). **(c)** DAI scores (n = 6 in each group). **(d)** Representative macroscopic features of colons. Scale bar: 1 cm. **(e)** Mean colon length (n = 6 in each group). **(f)** Determination of serum levels of IL-6, IL-12p70, IL-17A and IL-23 by ELISA (n = 6 in each group). **(g)** Colonic MPO activity (n = 4 in each group). **(h)** Representative images of H&E staining of colon sections. Scale bar: 100 μm. **(i)** Histological scores of colon sections (n = 6 in each group). **(j)** Quantitative RT-PCR analysis of the relative expression levels of TNF-α mRNA in the colon (n = 6 in each group). **(k)** Determination of the absolute expression levels of TNF-α protein in the colon by ELISA (n = 6 in each group). Values are presented as the mean ± SEM. Significance was determined using one-way ANOVA followed by Dunnett's multiple comparison in panels c, e, f, g, i, j and k or two-way ANOVA followed by Dunnett's multiple comparison in panel b. * $p < 0.05$; ** $p < 0.01$; *** $p < 0.005$.

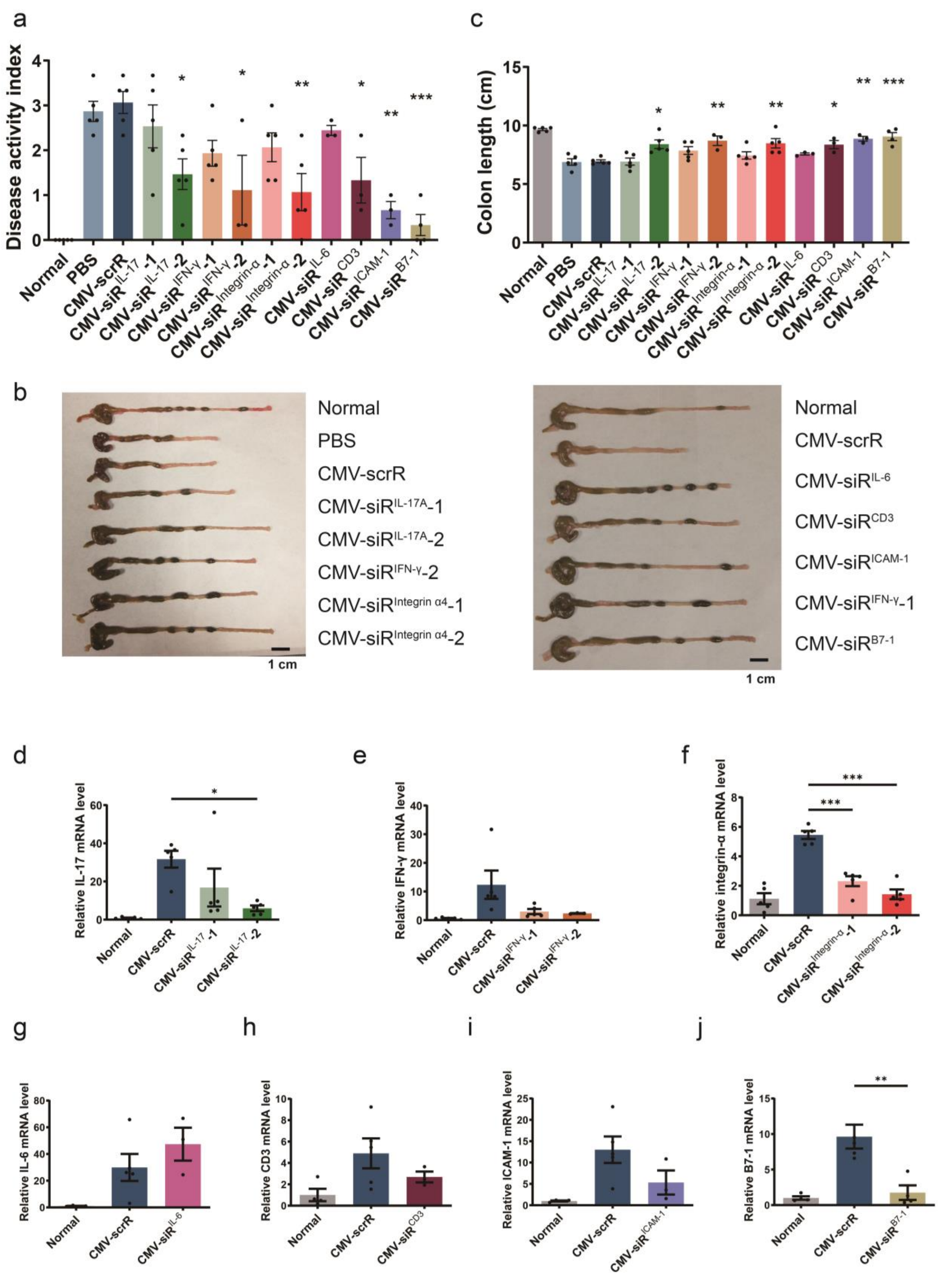
a
Disease activity index
Normal
PBS
CMV-scrR
CMV-siR IL-17 -1
CMV-siR IL-17 -2
CMV-siR IFN-γ -1
CMV-siR IFN-γ -2
CMV-siR Integrin-α -1
CMV-siR Integrin-α -2
CMV-siR IL-6
CMV-siR CD3
CMV-siR ICAM-1
CMV-siR B7-1
c
Colon length (cm)
b
Normal
PBS
CMV-scrR
CMV-siR IL-17A -1
CMV-siR IL-17A -2
CMV-siR IFN-γ -2
CMV-siR Integrin α4 -1
CMV-siR Integrin α4 -2
1 cm
Normal
CMV-scrR
CMV-siR IL-6
CMV-siR CD3
CMV-siR ICAM-1
CMV-siR IFN-γ -1
CMV-siR B7-1
1 cm
d
Relative IL-17 mRNA level
e
Relative IFN-γ mRNA level
f
Relative integrin-α mRNA level
g
Relative IL-6 mRNA level
h
Relative CD3 mRNA level
i
Relative ICAM-1 mRNA level
j
Relative B7-1 mRNA level

Supplementary Figure 10. Assessing the therapeutic efficacy of candidate siRNA expression cassettes. Acute UC was induced in male BALB/c mice by replacing their drinking water with a 2.5% DSS solution for 7 days. From day 3 of modelling, the mice were intravenously injected once a day with PBS, CMV-scrR, two types of genetic circuits targeting IL-17A (CMV-siR$^{\text{IL-17A}}$-1 and CMV-siR$^{\text{IL-17A}}$-2), two types of genetic circuits targeting INF-γ (CMV-siR$^{\text{INF-}\gamma}$-1 and CMV-siR$^{\text{INF-}\gamma}$-2), a genetic circuit targeting IL-6 (CMV-siR$^{\text{IL-6}}$), two types of genetic circuits targeting integrin α4 (CMV-siR$^{\text{Integrin }\alpha 4}$-1 and CMV-siR$^{\text{Integrin }\alpha 4}$-2), a genetic circuit targeting ICAM-1 (CMV-siR$^{\text{ICAM-1}}$), a genetic circuit targeting CD3 (CMV-siR$^{\text{CD3}}$) or a genetic circuit targeting B7-1 (CMV-siR$^{\text{B7-1}}$). Each genetic circuit was injected at a dosage of 20 mg/kg. After 7 injections on day 10, the mice were euthanised and analysed. Untreated BALB/c mice were included as normal controls. **(a)** DAI scores (n = 3 in each group). **(b)** Representative macroscopic features of colons. Scale bar: 1 cm. **(c)** Mean colon length (n = 3 in each group). **(d)** Quantitative RT-PCR analysis of the relative expression levels of IL-17A mRNA in the colon after treatment with CMV-siR$^{\text{IL-17A}}$-1 and CMV-siR$^{\text{IL-17A}}$-2 (n = 3 in each group). **(e)** Quantitative RT-PCR analysis of the relative expression levels of IFN-γ mRNA in the colon after treatment with CMV-siR$^{\text{INF-}\gamma}$-1 and CMV-siR$^{\text{INF-}\gamma}$-2 (n = 3 in each group). **(f)** Quantitative RT-PCR analysis of the relative expression levels of integrin α4 mRNA in the colon after treatment with CMV-siR$^{\text{Integrin }\alpha 4}$-1 and CMV-siR$^{\text{Integrin }\alpha 4}$-2 (n = 3 in each group). **(g)** Quantitative RT-PCR analysis of the relative expression levels of IL-6 mRNA in the colon after treatment with CMV-siR$^{\text{IL-6}}$ (n = 3 in each group). **(h)** Quantitative RT-PCR analysis of the relative expression levels of CD3 mRNA in the colon after treatment with CMV-siR$^{\text{CD3}}$ (n = 3 in each group). **(i)** Quantitative RT-PCR analysis of the relative expression levels of ICAM-1 mRNA in the colon after treatment with CMV-siR$^{\text{ICAM-1}}$ (n = 3 in each group). **(j)** Quantitative RT-PCR analysis of the relative expression levels of B7-1 mRNA in the colon after treatment with CMV-siR$^{\text{B7-1}}$ (n = 3 in each group). Values are presented as the mean ± SEM. Significance was determined using one-way ANOVA followed by Dunnett's multiple comparison. In panels a and b, all groups were compared with CMV-scrR. * $p < 0.05$; ** $p < 0.01$; *** $p < 0.005$.

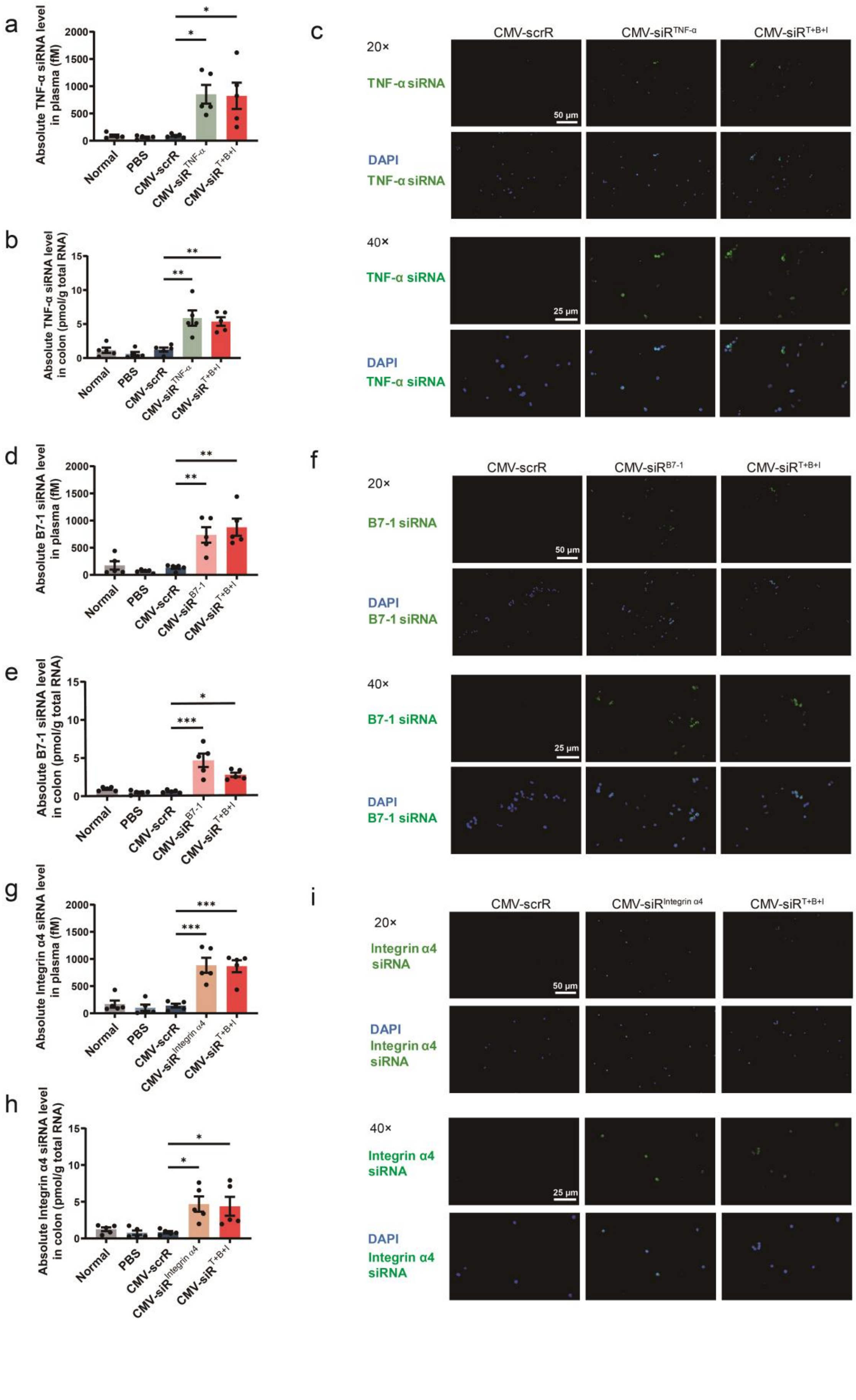

a
Absolute TNF-α siRNA level in plasma (fM)
Normal
PBS
CMV-scrR
CMV-siR TNF-α
CMV-siR T+B+I
b
Absolute TNF-α siRNA level in colon (pmol/g total RNA)
c
CMV-scrR
CMV-siR TNF-α
CMV-siR T+B+I
20×
40×
TNF-α siRNA
DAPI
50 μm
25 μm
d
Absolute B7-1 siRNA level in plasma (fM)
CMV-siR B7-1
e
Absolute B7-1 siRNA level in colon (pmol/g total RNA)
f
B7-1 siRNA
g
Absolute Integrin α4 siRNA level in plasma (fM)
CMV-siR Integrin α4
h
Absolute Integrin α4 siRNA level in colon (pmol/g total RNA)
i
Integrin α4 siRNA

Supplementary Figure 11. Tracking and visualisation of the delivery of self-assembled siRNAs into desired tissues and cells in the DSS-induced acute UC model. Chronic UC was induced in male BALB/c mice by rhythmically administering to mice 2.5% DSS for 1 week and water for 2 weeks and the cycle was repeated for 3 times. Four days after each DSS drinking, mice were intravenously injected with PBS or with equal dose (10 mg/kg) of CMV-scrR, CMV-siR$^{TNF\text{-}\alpha}$, CMV-siR$^{Integrin\ \alpha4}$, CMV-siR$^{B7\text{-}1}$ or CMV-siR^{T+B+I} circuit for a total of 3 times, once every 2 days. Two days after the final injection on day 52, the mice were euthanised, and the presence of siRNAs in various tissues and cells was evaluated. **(a and b)** Quantitative RT-PCR analysis of the absolute expression levels of TNF-α siRNA in the plasma and colon (n = 5 in each group). **(c)** *In situ* detection of TNF-α siRNA in colonic macrophages extracted from DSS mice. Positive *in situ* hybridisation signals are shown in green, and DAPI-stained nuclei are shown in blue. Scale bar: 50 μm (20 ×) or 25 μm (40 ×). **(d and e)** Quantitative RT-PCR analysis of the absolute expression levels of B7-1 siRNA in the plasma and colon (n = 5 in each group). **(f)** *In situ* detection of B7-1 siRNA in colonic macrophages extracted from DSS mice. Positive *in situ* hybridisation signals are shown in green, and DAPI-stained nuclei are shown in blue. Scale bar: 50 μm (20 ×) or 25 μm (40 ×). **(g and h)** Quantitative RT-PCR analysis of the absolute expression levels of integrin α4 siRNA in the plasma and colon (n = 5 in each group). **(i)** *In situ* detection of integrin α4 siRNA in peripheral blood $CD4^+$ T cells extracted from DSS mice. Positive *in situ* hybridisation signals are shown in green, and DAPI-stained nuclei are shown in blue. Scale bar: 50 μm (20 ×) or 25 μm (40 ×). Values are presented as the mean ± SEM. Significance was determined using one-way ANOVA followed by Dunnett's multiple comparison. * $p < 0.05$; ** $p < 0.01$; *** $p < 0.005$.

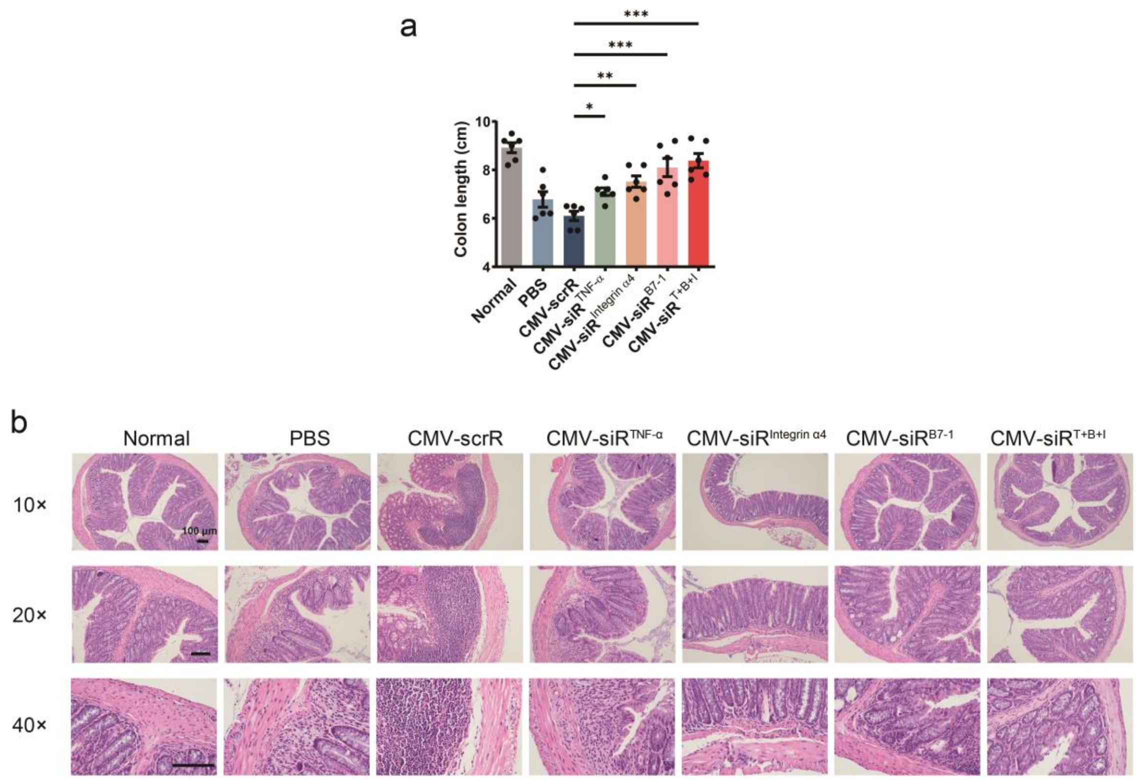

Supplementary Figure 12. Intravenous injection of the multi-targeted CMV-siR^{T+B+I} circuit exerts a synergistic therapeutic effect against DSS-induced chronic UC. Chronic UC was induced in male BALB/c mice by rhythmically administering to mice 2.5% DSS for 1 week and water for 2 weeks and the cycle was repeated for 3 times. Four days after each DSS drinking, mice were intravenously injected with PBS or with equal dose (10 mg/kg) of CMV-scrR, CMV-siR$^{TNF-\alpha}$, CMV-siR$^{Integrin\ \alpha4}$, CMV-siR^{B7-1} or CMV-siR^{T+B+I} circuit for a total of 3 times, once every 2 days. Two days after the final injection on day 52, the mice were euthanised and analysed. Untreated BALB/c mice were included as normal controls. **(a)** Mean colon length (n = 6 in each group). **(b)** Representative images of H&E staining of colon sections. Scale bar: 100 μm. Values are presented as the mean ± SEM. Significance was determined using one-way ANOVA followed by Dunnett's multiple comparison. * $p < 0.05$; ** $p < 0.01$; *** $p < 0.005$.

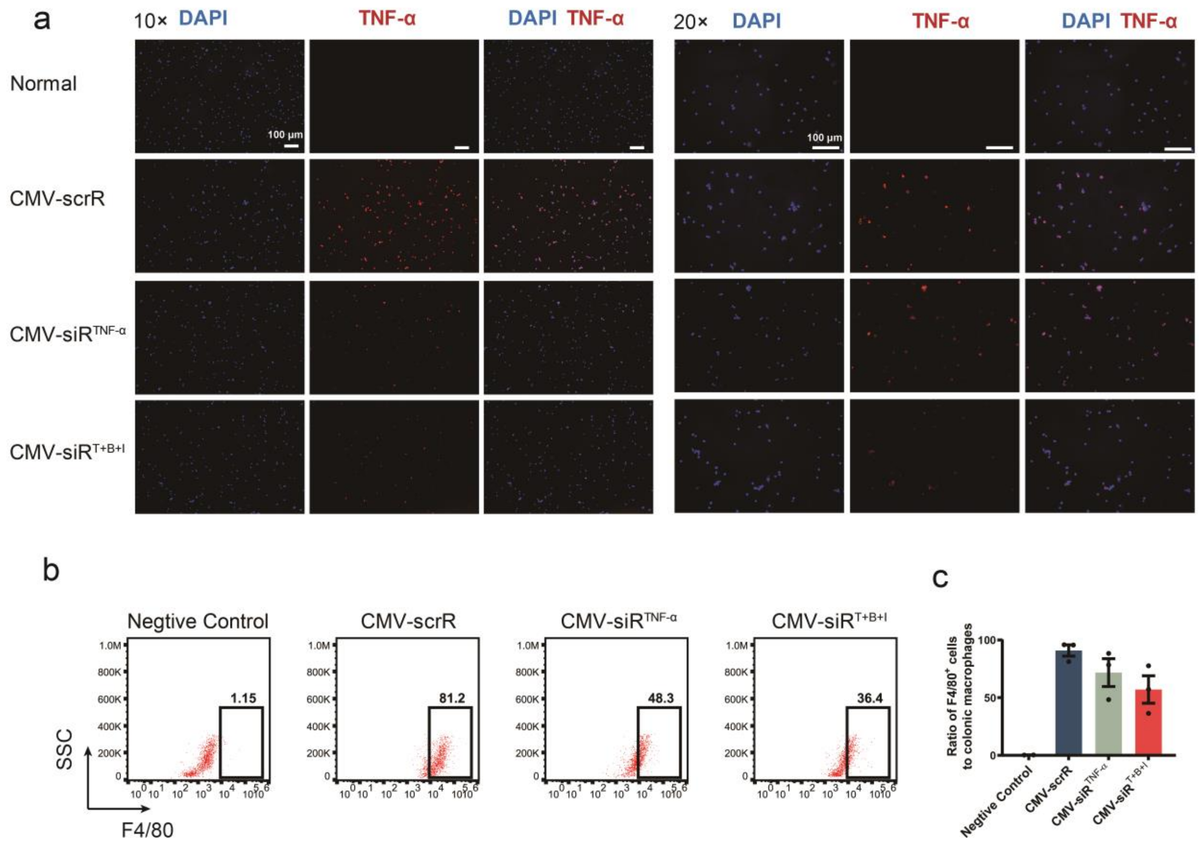

Supplementary Figure 13. Intravenous injection of the multi-targeted CMV-siR^{T+B+I} circuit inhibits TNF-α in macrophages. (a) Immunofluorescence staining of TNF-α (red) and DAPI (blue) in primary macrophages isolated from colonic lamina propria and cultured in conditioned medium for 12 hours. Scale bar: 100 µm. **(b)** Representative flow cytometric plots of F4/80$^+$ on colonic lamina propria macrophages. IgG isotype-labelled cells was used as a negative control. **(c)** The population of F4/80$^+$ cells in all colonic lamina propria macrophages (n = 3 in each group). Values are presented as the mean ± SEM. Significance was determined using one-way ANOVA followed by Dunnett’s multiple comparison.

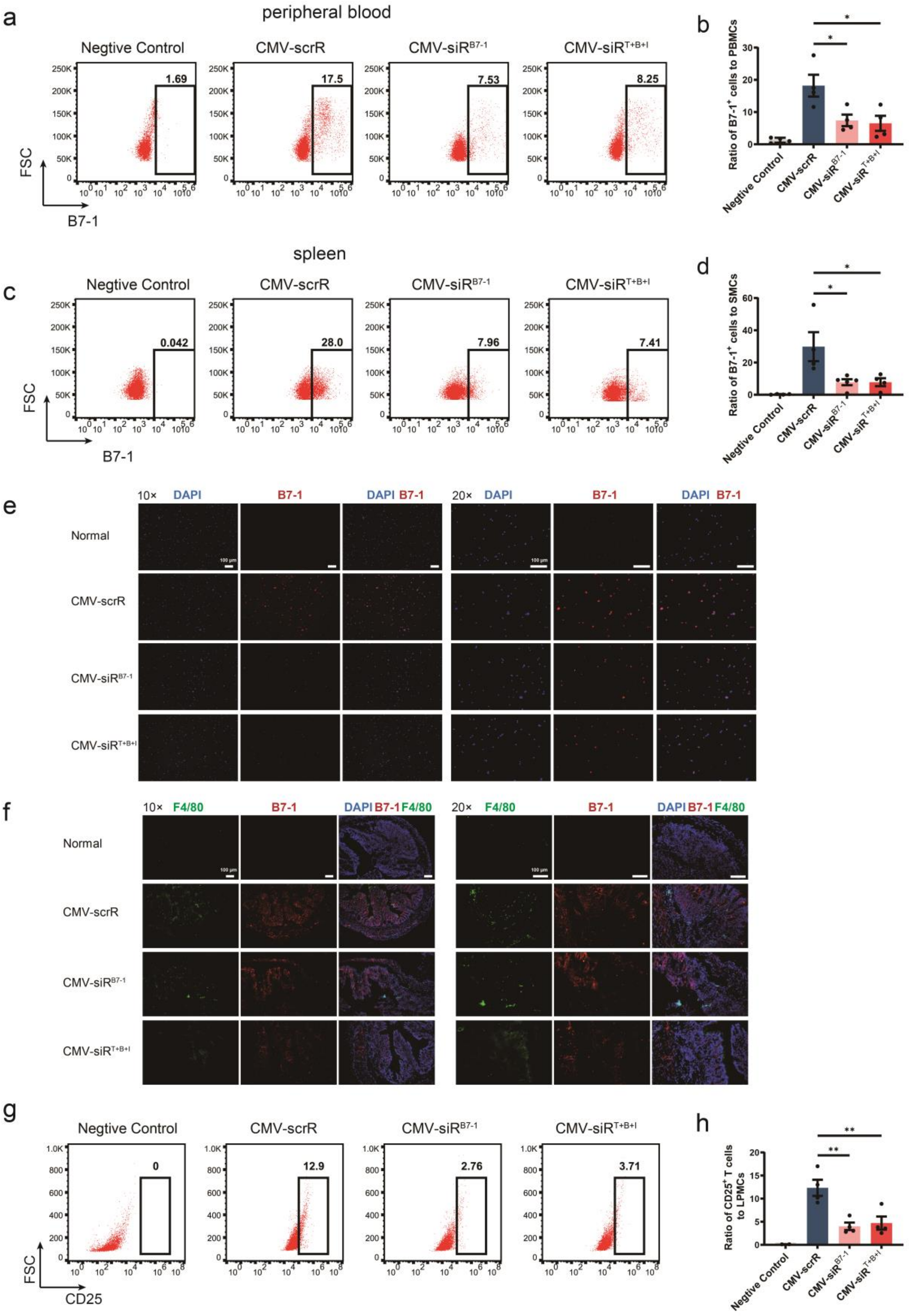
a
peripheral blood
Negtive Control
CMV-scrR
CMV-siR^B7-1
CMV-siR^T+B+I
1.69
17.5
7.53
8.25
FSC
B7-1
b
Ratio of B7-1+ cells to PBMCs
Negtive Control
CMV-scrR
CMV-siR^B7-1
CMV-siR^T+B+I
spleen
c
Negtive Control
CMV-scrR
CMV-siR^B7-1
CMV-siR^T+B+I
0.042
28.0
7.96
7.41
FSC
B7-1
d
Ratio of B7-1+ cells to SMCs
e
10×
DAPI
B7-1
DAPI B7-1
20×
Normal
CMV-scrR
CMV-siR^B7-1
CMV-siR^T+B+I
f
10×
F4/80
B7-1
DAPI B7-1 F4/80
20×
Normal
CMV-scrR
CMV-siR^B7-1
CMV-siR^T+B+I
g
Negtive Control
CMV-scrR
CMV-siR^B7-1
CMV-siR^T+B+I
0
12.9
2.76
3.71
FSC
CD25
h
Ratio of CD25+ T cells to LPMCs
Negtive Control
CMV-scrR
CMV-siR^B7-1
CMV-siR^T+B+I

Supplementary Figure 14. Intravenous injection of the multi-targeted CMV-siR^{T+B+I} circuit inhibits B7-1 in macrophages. (a) Representative flow cytometric plots of B7-1 on the surface of peripheral blood mononuclear cells. IgG isotype-labelled cells was used as a negative control. **(b)** The population of B7-1^{+} cells in peripheral blood mononuclear cells (PBMCs) ($n = 4$ in each group). **(c)** Representative flow cytometric plots of B7-1 on the surface of splenic mononuclear cells. IgG isotype-labelled cells was used as a negative control. **(d)** The population of B7-1^{+} cells in splenic mononuclear cells (SMCs) ($n = 4$ in each group). **(e)** Immunofluorescence staining of B7-1 (red) and DAPI (blue) in primary macrophages isolated from colonic lamina propria and cultured in conditioned medium for 12 hours. Scale bar: 100 μm. **(f)** Immunofluorescence staining of B7-1 (red), F4/80 (green) and DAPI (blue) in colon sections. Double-positive (red and green) signals indicate B7-1^{+} macrophages. Scale bar: 100 μm. **(g)** Representative flow cytometric plots of CD25 on colonic lamina propria mononuclear cells. IgG isotype-labelled cells was used as a negative control. **(h)** The population of CD25^{+} T cells in total colonic lamina propria mononuclear cells ($n = 4$ in each group). Values are presented as the mean ± SEM. Significance was determined using one-way ANOVA followed by Dunnett's multiple comparison. * $p < 0.05$; ** $p < 0.01$.

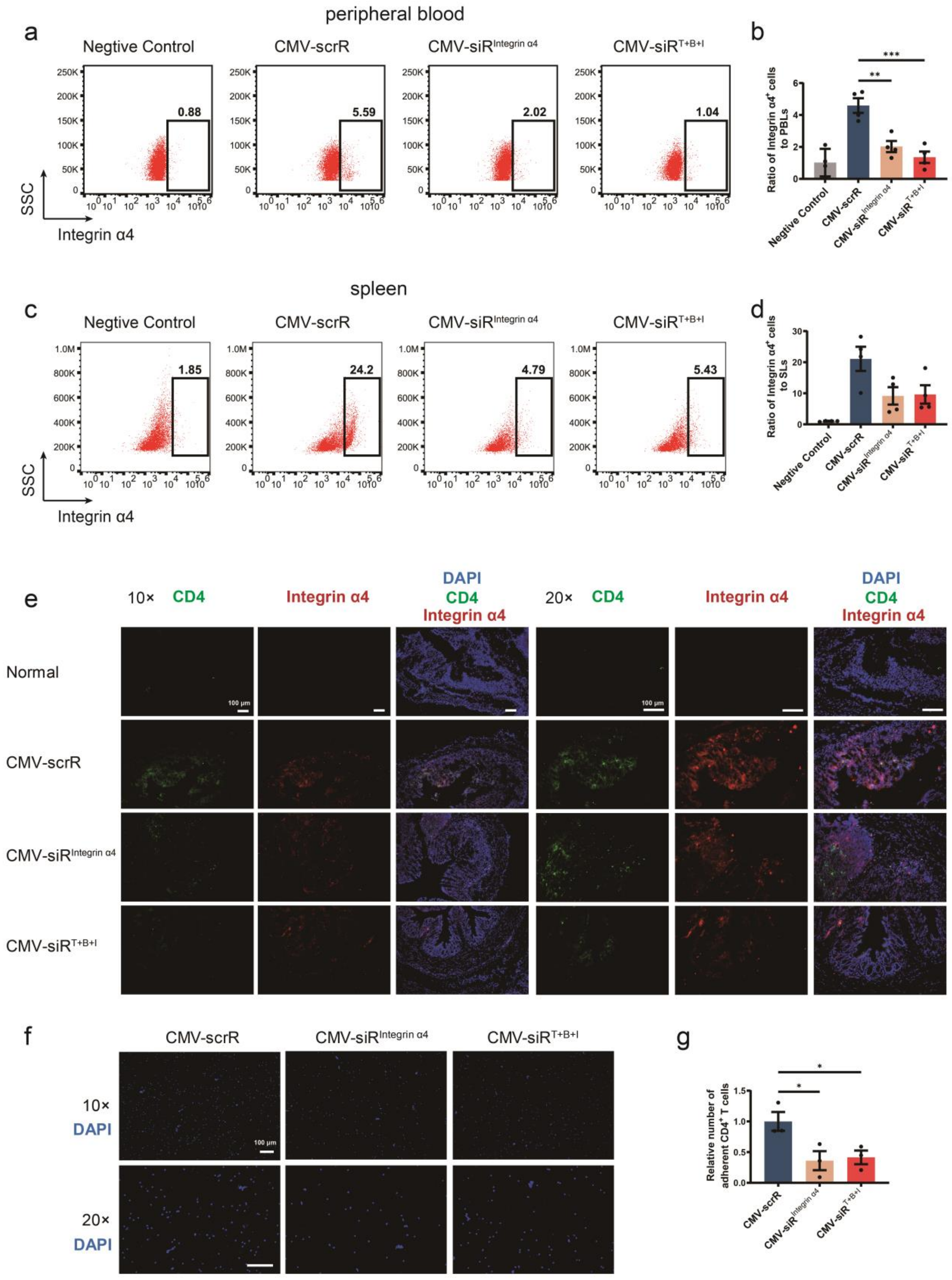

peripheral blood
a
Negtive Control
CMV-scrR
CMV-siRIntegrin α4
CMV-siRT+B+I
0.88
5.59
2.02
1.04
SSC
Integrin α4
b
Ratio of Integrin α4+ cells to PBLs
spleen
c
1.85
24.2
4.79
5.43
d
Ratio of Integrin α4+ cells to SLs
e
10×
20×
CD4
Integrin α4
DAPI
Normal
CMV-scrR
CMV-siRIntegrin α4
CMV-siRT+B+I
100 μm
f
10× DAPI
20× DAPI
g
Relative number of adherent CD4+ T cells

Supplementary Figure 15. Intravenous injection of the multi-targeted CMV-siR^{T+B+I} circuit inhibits integrin α4 in CD4^{+} T cells. (a) Representative flow cytometric plots of integrin α4 on the surface of peripheral blood lymphocytes. IgG isotype-labelled cells was used as a negative control. **(b)** The population of integrin α4^{+} cells in peripheral blood lymphocytes (PBLs) (n = 4 in each group). **(c)** Representative flow cytometric plots of integrin α4 on the surface of splenic lymphocytes. IgG isotype-labelled cells was used as a negative control. **(d)** The population of integrin α4^{+} cells in splenic lymphocytes (SLs) (n = 4 in each group). **(e)** Immunofluorescence staining of integrin α4 (red), CD4 (green) and DAPI (blue) in colon sections. Double-positive (red and green) signals indicate integrin α4^{+} CD4^{+} cells. Scale bar: 100 µm. **(f)** Representative images of adhesion assays showing the adhesion of peripheral blood integrin α4^{+} CD4^{+} T cells to plates coated with E-cadherin. Scale bars: 100 µm. **(g)** Quantitative analysis of the amounts of adherent CD4^{+} T cells (n = 3 in each group). Values are presented as the mean ± SEM. Significance was determined using one-way ANOVA followed by Dunnett's multiple comparison. * $p < 0.05$; ** $p < 0.01$; *** $p < 0.005$.

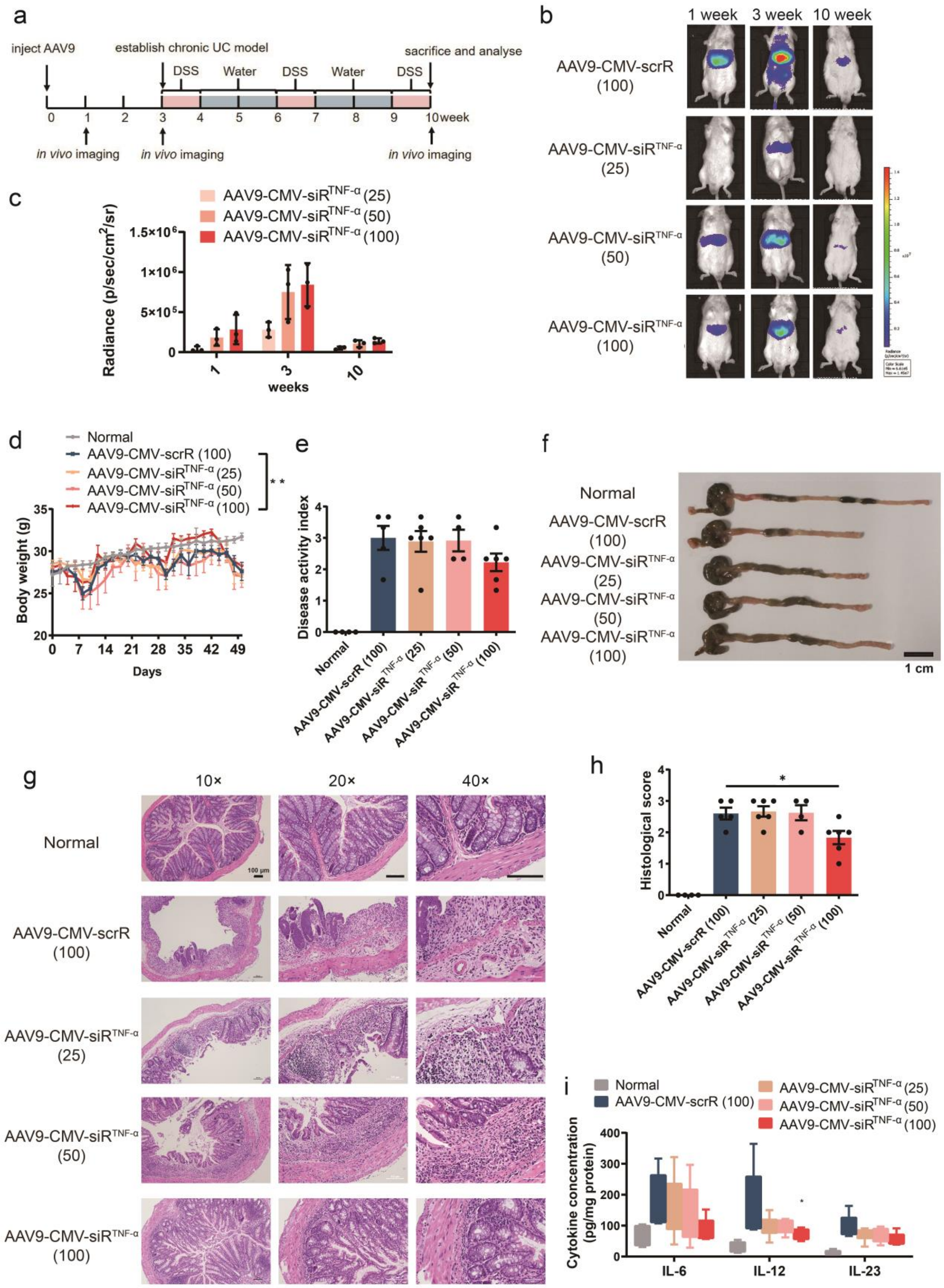

a
inject AAV9
establish chronic UC model
sacrifice and analyse
DSS
Water
DSS
Water
DSS
0 1 2 3 4 5 6 7 8 9 10week
in vivo imaging
in vivo imaging
in vivo imaging
b
1 week 3 week 10 week
AAV9-CMV-scrR (100)
AAV9-CMV-siR TNF-α (25)
AAV9-CMV-siR TNF-α (50)
AAV9-CMV-siR TNF-α (100)
c
AAV9-CMV-siR TNF-α (25)
AAV9-CMV-siR TNF-α (50)
AAV9-CMV-siR TNF-α (100)
Radiance (p/sec/cm2/sr)
weeks
d
Normal
AAV9-CMV-scrR (100)
AAV9-CMV-siR TNF-α (25)
AAV9-CMV-siR TNF-α (50)
AAV9-CMV-siR TNF-α (100)
Body weight (g)
Days
e
Disease activity index
f
Normal
AAV9-CMV-scrR (100)
AAV9-CMV-siR TNF-α (25)
AAV9-CMV-siR TNF-α (50)
AAV9-CMV-siR TNF-α (100)
1 cm
g
10× 20× 40×
Normal
AAV9-CMV-scrR (100)
AAV9-CMV-siR TNF-α (25)
AAV9-CMV-siR TNF-α (50)
AAV9-CMV-siR TNF-α (100)
h
Histological score
i
Normal
AAV9-CMV-scrR (100)
AAV9-CMV-siR TNF-α (25)
AAV9-CMV-siR TNF-α (50)
AAV9-CMV-siR TNF-α (100)
Cytokine concentration (pg/mg protein)
IL-6 IL-12 IL-23

Supplementary Figure 16. Intravenous injection of the AAV9-CMV-siR$^{TNF\text{-}\alpha}$ induces a long-term therapeutic effect in the DSS-induced chronic UC model. (a) Flow chart of the experimental design. **(b and c)** Evaluation of AAV-mediated luciferase expression to reflect co-expressed TNF-α siRNA accumulation *in vivo* (n = 3 in each group). **(d)** Body weight curves (Normal, n = 4; AAV9-CMV-scrR (100), n = 5; AAV9-CMV-siR$^{TNF\text{-}\alpha}$ (25), n = 6; AAV9-CMV-siR$^{TNF\text{-}\alpha}$ (50), n = 4; AAV9-CMV-siR$^{TNF\text{-}\alpha}$ (100), n = 6). **(e)** DAI scores (Normal, n = 4; AAV9-CMV-scrR (100), n = 5; AAV9-CMV-siR$^{TNF\text{-}\alpha}$ (25), n = 6; AAV9-CMV-siR$^{TNF\text{-}\alpha}$ (50), n = 4; AAV9-CMV-siR$^{TNF\text{-}\alpha}$ (100), n = 6). **(f)** Representative macroscopic features of colons. Scale bar: 1 cm. **(g)** Representative images of H&E staining of colon sections. Scale bar: 100 μm. **(h)** Histological scores of colon sections (Normal, n = 4; AAV9-CMV-scrR (100), n = 5; AAV9-CMV-siR$^{TNF\text{-}\alpha}$ (25), n = 6; AAV9-CMV-siR$^{TNF\text{-}\alpha}$ (50), n = 4; AAV9-CMV-siR$^{TNF\text{-}\alpha}$ (100), n = 6). **(i)** Determination of serum levels of IL-6, IL-12p70 and IL-23 by ELISA (Normal, n = 4; AAV9-CMV-scrR (100), n = 5; AAV9-CMV-siR$^{TNF\text{-}\alpha}$ (25), n = 6; AAV9-CMV-siR$^{TNF\text{-}\alpha}$ (50), n = 4; AAV9-CMV-siR$^{TNF\text{-}\alpha}$ (100), n = 6). Values are presented as the mean ± SEM. Significance was determined using one-way ANOVA followed by Dunnett's multiple comparison in panels e, h and i or two-way ANOVA followed by Dunnett's multiple comparison in panel d. * $p < 0.05$; ** $p < 0.01$.

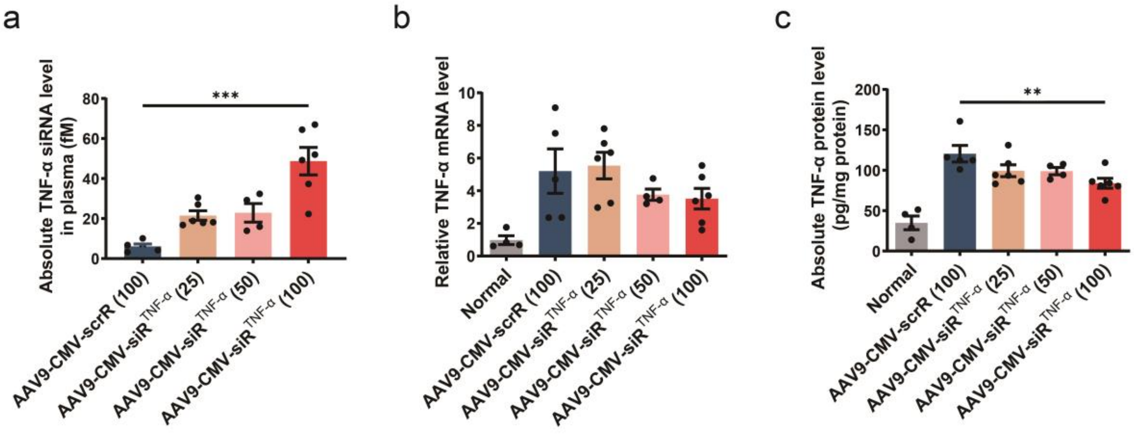

Supplementary Figure 17. Intravenous injection of the AAV9-CMV-siR$^{TNF-\alpha}$ results in TNF-α siRNA accumulation in the plasma and TNF-α downregulation in the colon. (a) Quantitative RT-PCR analysis of the absolute expression levels of TNF-α siRNA in plasma (Normal, n = 4; AAV9-CMV-scrR (100), n = 5; AAV9-CMV-siR$^{TNF-\alpha}$ (25), n = 6; AAV9-CMV-siR$^{TNF-\alpha}$ (50), n = 4; AAV9-CMV-siR$^{TNF-\alpha}$ (100), n = 6). **(b)** Quantitative RT-PCR analysis of the relative expression levels of TNF-α mRNA in the colon (Normal, n = 4; AAV9-CMV-scrR (100), n = 5; AAV9-CMV-siR$^{TNF-\alpha}$ (25), n = 6; AAV9-CMV-siR$^{TNF-\alpha}$ (50), n = 4; AAV9-CMV-siR$^{TNF-\alpha}$ (100), n = 6). **(c)** Determination of the absolute expression levels of TNF-α protein in the colon by ELISA (Normal, n = 4; AAV9-CMV-scrR (100), n = 5; AAV9-CMV-siR$^{TNF-\alpha}$ (25), n = 6; AAV9-CMV-siR$^{TNF-\alpha}$ (50), n = 4; AAV9-CMV-siR$^{TNF-\alpha}$ (100), n = 6). Values are presented as the mean ± SEM. Significance was determined using one-way ANOVA followed by Dunnett's multiple comparison. ** $p < 0.01$, *** $p < 0.005$.

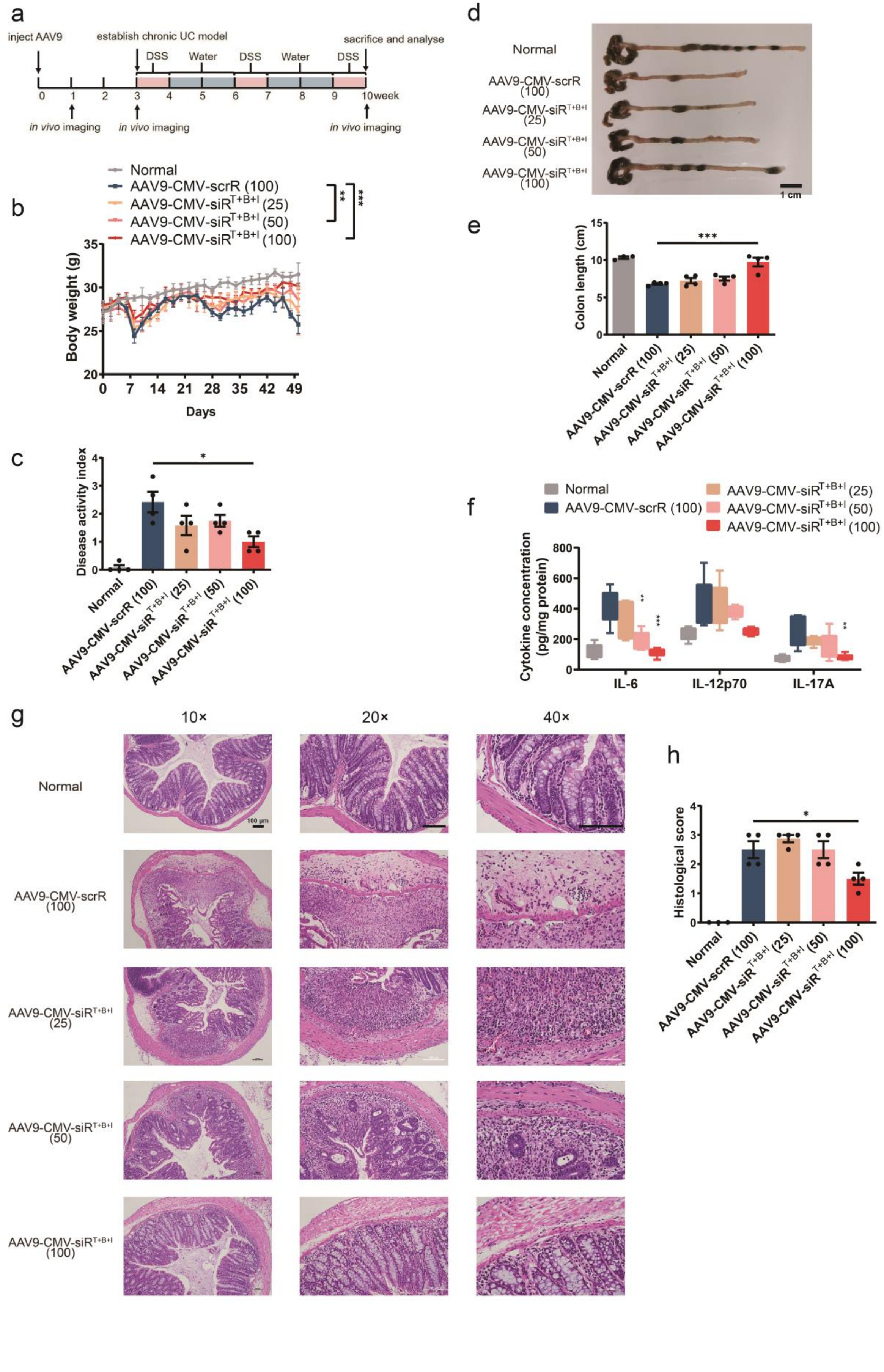
a
inject AAV9
establish chronic UC model
sacrifice and analyse
DSS
Water
DSS
Water
DSS
0 1 2 3 4 5 6 7 8 9 10week
in vivo imaging
in vivo imaging
in vivo imaging
b
Normal
AAV9-CMV-scrR (100)
AAV9-CMV-siR^T+B+I (25)
AAV9-CMV-siR^T+B+I (50)
AAV9-CMV-siR^T+B+I (100)
**
***
Body weight (g)
Days
c
Disease activity index
*
d
Normal
AAV9-CMV-scrR (100)
AAV9-CMV-siR^T+B+I (25)
AAV9-CMV-siR^T+B+I (50)
AAV9-CMV-siR^T+B+I (100)
1 cm
e
Colon length (cm)
***
f
Cytokine concentration (pg/mg protein)
IL-6
IL-12p70
IL-17A
g
10×
20×
40×
100 μm
h
Histological score
*

Supplementary Figure 18. Intravenous injection of the AAV9-CMV-siR^{T+B+I} induces long-term combination therapy in the DSS-induced chronic UC model. (a) Flow chart of the experimental design. **(b)** Body weight curves (n = 4 in each group). **(c)** DAI scores (n = 4 in each group). **(d)** Representative macroscopic features of colons. Scale bar: 1 cm. **(e)** Mean colon length (n = 4 in each group). **(f)** Determination of serum levels of IL-6, IL-12p70 and IL-17A by ELISA (n = 4 in each group). **(g)** Representative images of H&E staining of colon sections. Scale bar: 100 μm. **(h)** Histological scores of colon sections (n = 4 in each group). Values are presented as the mean ± SEM. Significance was determined using one-way ANOVA followed by Dunnett's multiple comparison in panels c, e, f and h or two-way ANOVA followed by Dunnett's multiple comparison in panel b. * $p < 0.05$; ** $p < 0.01$; *** $p < 0.005$.

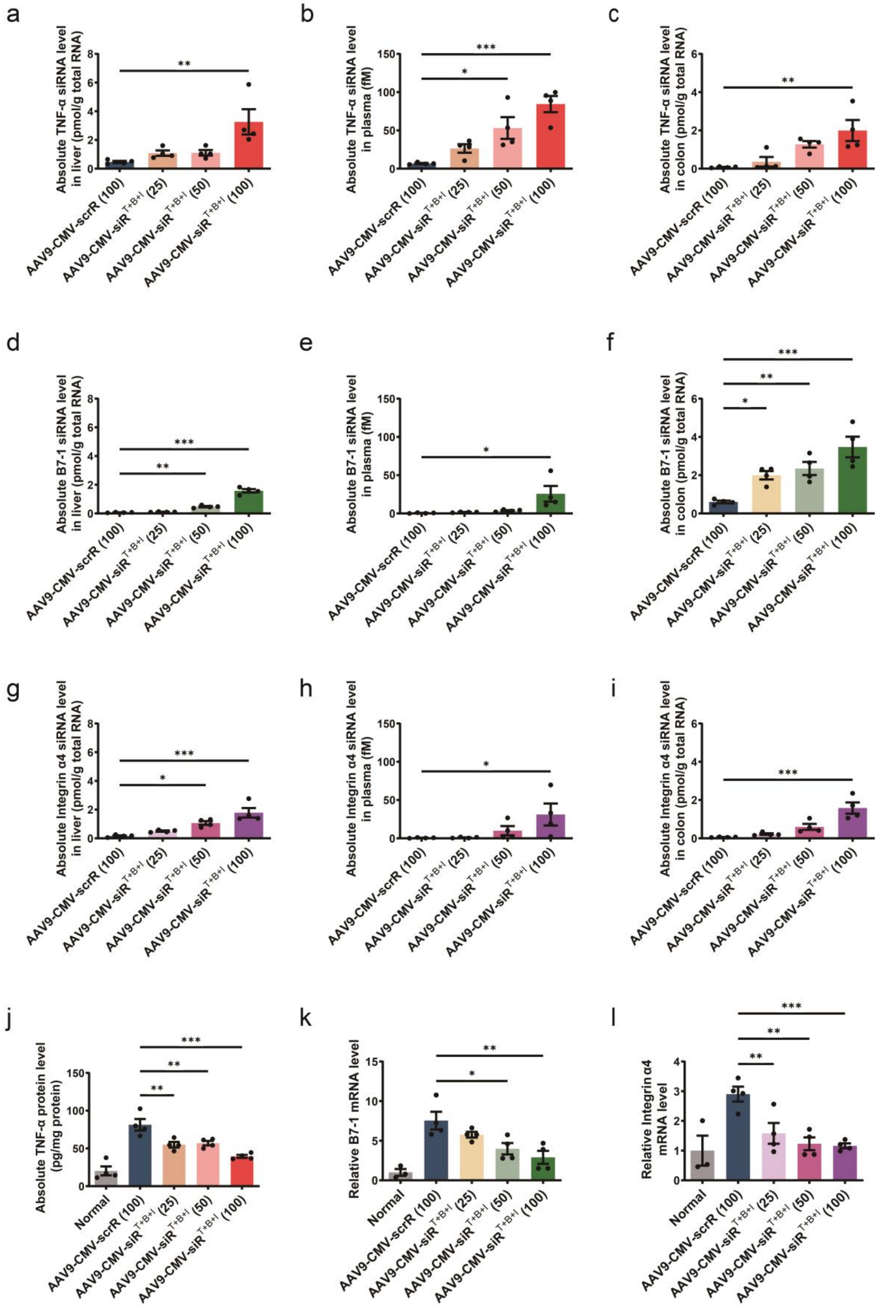
a
b
c
d
e
f
g
h
i
j
k
l
Absolute TNF-α siRNA level in liver (pmol/g total RNA)
Absolute TNF-α siRNA level in plasma (fM)
Absolute TNF-α siRNA level in colon (pmol/g total RNA)
Absolute B7-1 siRNA level in liver (pmol/g total RNA)
Absolute B7-1 siRNA level in plasma (fM)
Absolute B7-1 siRNA level in colon (pmol/g total RNA)
Absolute Integrin α4 siRNA level in liver (pmol/g total RNA)
Absolute Integrin α4 siRNA level in plasma (fM)
Absolute Integrin α4 siRNA level in colon (pmol/g total RNA)
Absolute TNF-α protein level (pg/mg protein)
Relative B7-1 mRNA level
Relative Integrin α4 mRNA level
Normal
AAV9-CMV-scrR (100)
AAV9-CMV-siR^T+B+I (25)
AAV9-CMV-siR^T+B+I (50)
AAV9-CMV-siR^T+B+I (100)

Supplementary Figure 19. Intravenous injection of the AAV9-CMV-siR^{T+B+I} results in accumulation of TNF-α siRNA, B7-1 siRNA and integrin α4 siRNA *in vivo* and silencing of target genes in the colon. (a-c) Quantitative RT-PCR analysis of the absolute expression levels of TNF-α siRNA in the liver, plasma and colon (n = 4 in each group). **(d-f)** Quantitative RT-PCR analysis of the absolute expression levels of B7-1 siRNA in the liver, plasma and colon (n = 4 in each group). **(g-i)** Quantitative RT-PCR analysis of the absolute expression levels of integrin α4 siRNA in the liver, plasma and colon (n = 4 in each group). **(j)** Determination of the absolute expression levels of TNF-α protein in the colon by ELISA (n = 4 in each group). **(k and l)** Quantitative RT-PCR analysis of the relative expression levels of B7-1 mRNA and integrin α4 mRNA in the colon (n = 4 in each group). Values are presented as the mean ± SEM. Significance was determined using one-way ANOVA followed by Dunnett's multiple comparison. * $p < 0.05$; ** $p < 0.01$; *** $p < 0.005$.

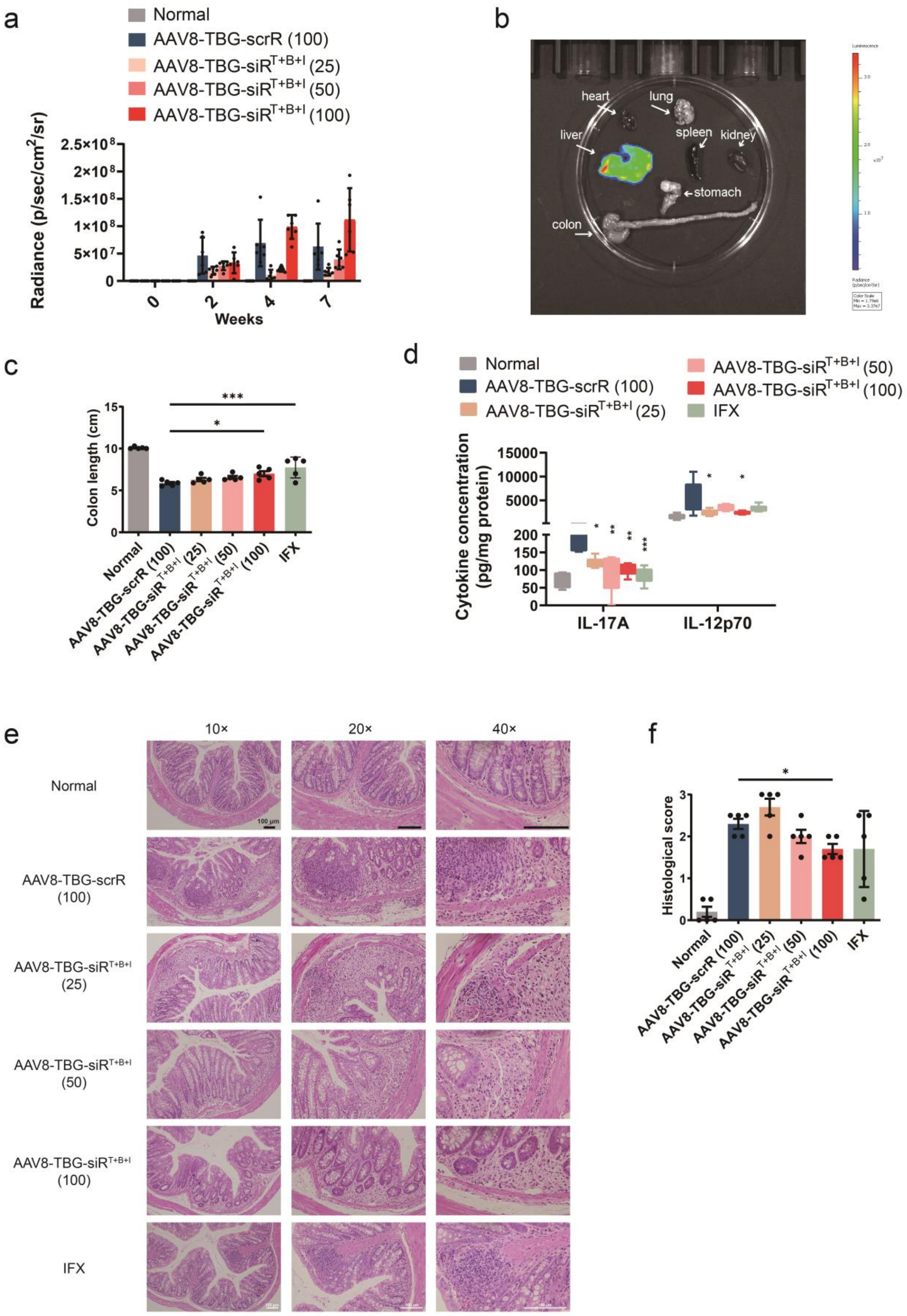
a
Normal
AAV8-TBG-scrR (100)
AAV8-TBG-siRT+B+I (25)
AAV8-TBG-siRT+B+I (50)
AAV8-TBG-siRT+B+I (100)
Radiance (p/sec/cm2/sr)
Weeks
b
heart
lung
liver
spleen
kidney
stomach
colon
c
Colon length (cm)
Normal
AAV8-TBG-scrR (100)
AAV8-TBG-siRT+B+I (25)
AAV8-TBG-siRT+B+I (50)
AAV8-TBG-siRT+B+I (100)
IFX
d
IFX
Cytokine concentration (pg/mg protein)
IL-17A
IL-12p70
e
10×
20×
40×
Normal
AAV8-TBG-scrR (100)
AAV8-TBG-siRT+B+I (25)
AAV8-TBG-siRT+B+I (50)
AAV8-TBG-siRT+B+I (100)
IFX
100 μm
f
Histological score

Supplementary Figure 20. Intravenous injection of the AAV8-TBG-siR^{T+B+I} induces long-term combination therapy in the DSS-induced chronic UC model. (a) Evaluation of AAV-mediated luciferase expression to reflect co-expressed siRNA accumulation *in vivo* (n = 6 in each group). **(b)** Evaluation of AAV-mediated luciferase expression in various tissues of mice. **(c)** Mean colon length (n = 5 in each group). **(d)** Determination of serum levels of IL-17A and IL-12p70 by ELISA (n = 5 in each group). **(e)** Representative images of H&E staining of colon sections. Scale bar: 100 μm. **(f)** Histological scores of colon sections (n = 5 in each group). Values are presented as the mean ± SEM. Significance was determined using one-way ANOVA followed by Dunnett's multiple comparison. * $p < 0.05$; ** $p < 0.01$; *** $p < 0.005$.

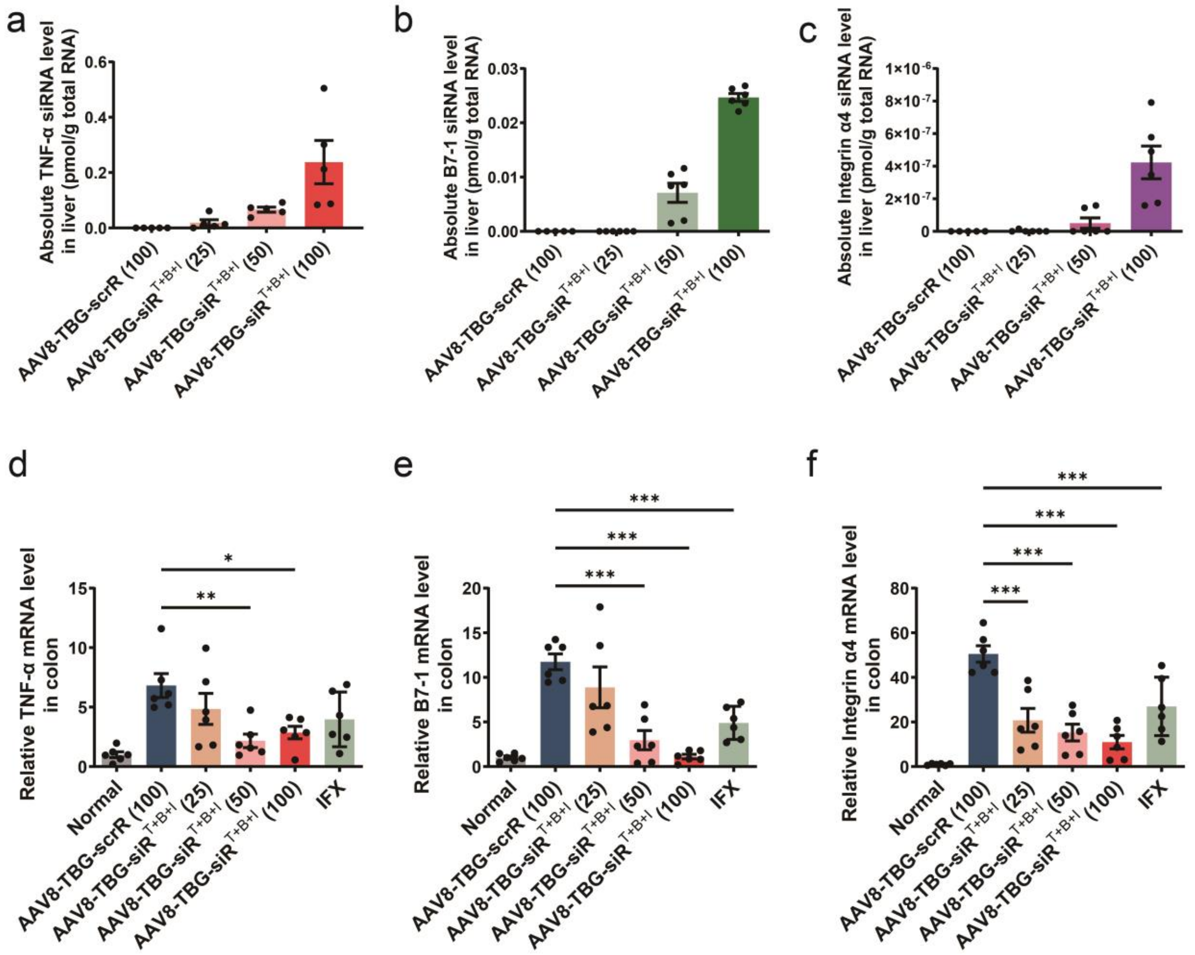

Supplementary Figure 21. Intravenous injection of the AAV8-TBG-siR^{T+B+I} results in accumulation of TNF-α siRNA, B7-1 siRNA and integrin α4 siRNA *in vivo* and silencing of target genes in the colon. (a-c) Quantitative RT-PCR analysis of the absolute expression levels of TNF-α siRNA, B7-1 siRNA and integrin α4 siRNA in the liver (n = 6 in each group). **(d-f)** Quantitative RT-PCR analysis of the absolute expression levels of TNF-α mRNA, B7-1 mRNA and integrin α4 mRNA in the colon (n = 6 in normal, AAV8-TBG-scrR (100), AAV8-TBG-siR^{T+B+I} (25), AAV8-TBG-siR^{T+B+I} (50), and AAV8-TBG-siR^{T+B+I} (100) groups; n = 5 in IFX group). Values are presented as the mean ± SEM. Significance was determined using one-way ANOVA followed by Dunnett's multiple comparison. * $p < 0.05$; ** $p < 0.01$; *** $p < 0.005$.

349 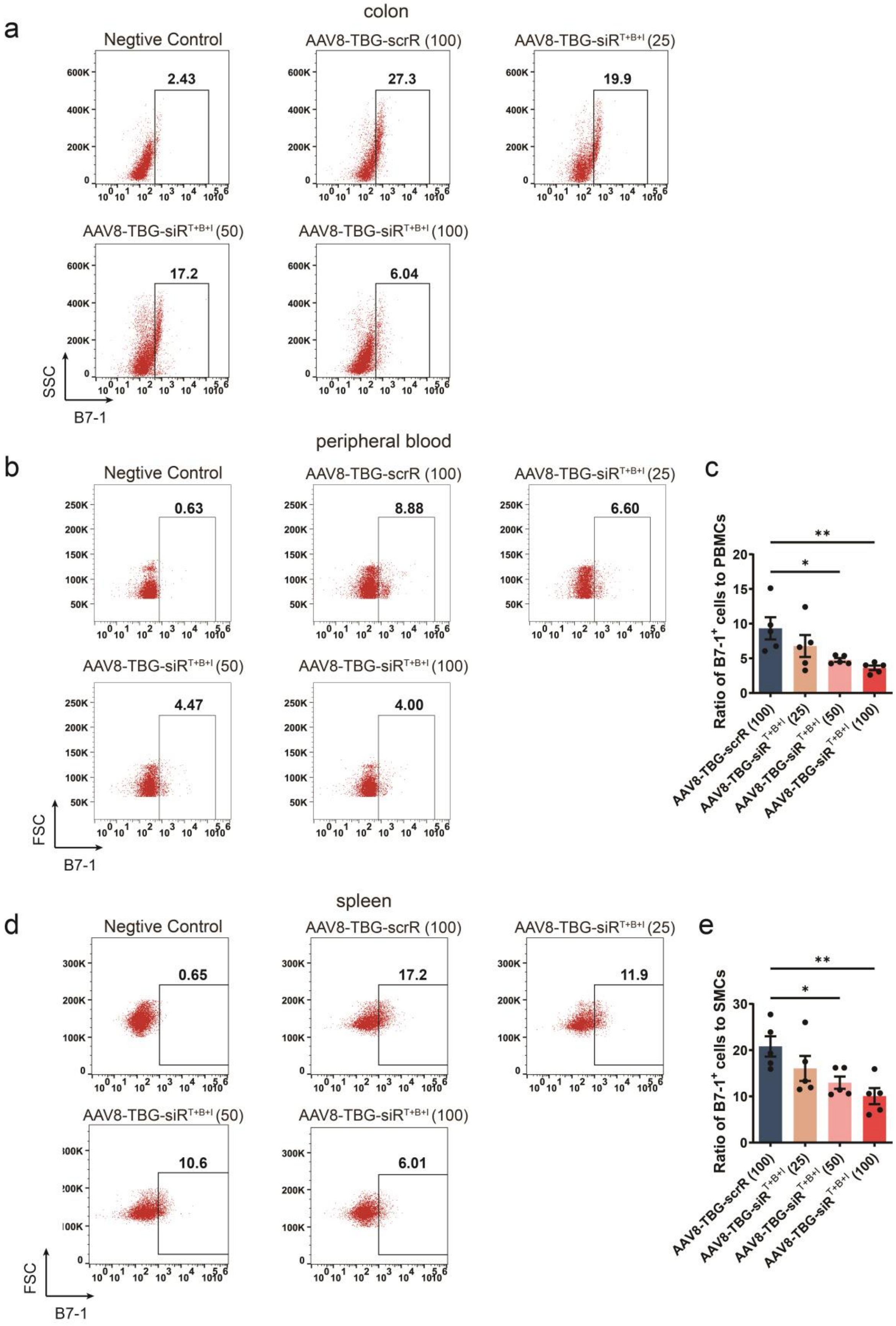
colon
a
Negtive Control
AAV8-TBG-scrR (100)
AAV8-TBG-siR T+B+I (25)
AAV8-TBG-siR T+B+I (50)
AAV8-TBG-siR T+B+I (100)
2.43
27.3
19.9
17.2
6.04
SSC
B7-1
peripheral blood
b
0.63
8.88
6.60
4.47
4.00
FSC
B7-1
c
Ratio of B7-1+ cells to PBMCs
spleen
d
0.65
17.2
11.9
10.6
6.01
FSC
B7-1
e
Ratio of B7-1+ cells to SMCs

Supplementary Figure 22. Intravenous injection of the AAV8-TBG-siR^{T+B+I} results in loss of B7-1 protein on the membrane surface of mononuclear cells. (a) Representative flow cytometric plots of B7-1 on the surface of colonic lamina propria mononuclear cells. IgG isotype-labelled cells was used as a negative control. **(b)** Representative flow cytometric plots of B7-1 on the surface of peripheral blood mononuclear cells. IgG isotype-labelled cells was used as a negative control. **(c)** The population of B7-1$^+$ cells in peripheral blood mononuclear cells (n = 5 in each group). **(d)** Representative flow cytometric plots of B7-1 on the surface of splenic mononuclear cells. IgG isotype-labelled cells was used as a negative control. **(e)** The population of B7-1$^+$ cells in splenic mononuclear cells (n = 5 in each group). Values are presented as the mean ± SEM. Significance was determined using one-way ANOVA followed by Dunnett's multiple comparison. * $p < 0.05$; ** $p < 0.01$.

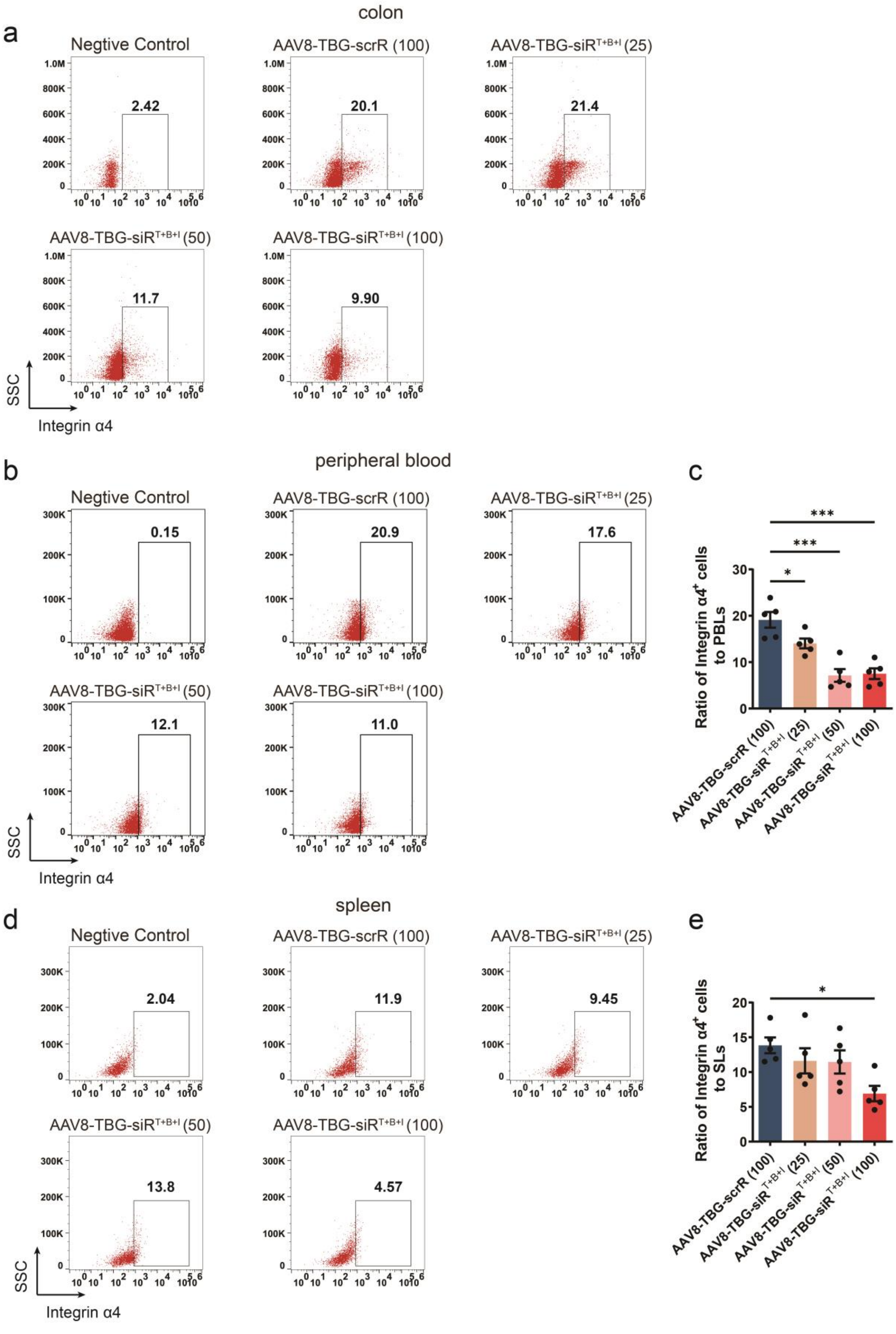

colon
a
Negtive Control
AAV8-TBG-scrR (100)
AAV8-TBG-siR T+B+I (25)
AAV8-TBG-siR T+B+I (50)
AAV8-TBG-siR T+B+I (100)
2.42
20.1
21.4
11.7
9.90
SSC
Integrin α4
peripheral blood
b
0.15
20.9
17.6
12.1
11.0
c
Ratio of Integrin α4+ cells to PBLs
***
*
spleen
d
2.04
11.9
9.45
13.8
4.57
e
Ratio of Integrin α4+ cells to SLs

Supplementary Figure 23. Intravenous injection of the AAV8-TBG-siR^{T+B+I} results in loss of integrin α4 protein on the membrane surface of mononuclear cells. (a) Representative flow cytometric plots of integrin α4 on the surface of mononuclear cells derived from the colonic lamina propria. IgG isotype-labelled cells was used as a negative control. **(b)** Representative flow cytometric plots of integrin α4 on the surface of peripheral blood lymphocytes. IgG isotype-labelled cells was used as a negative control. **(c)** The population of integrin α4^{+} cells in peripheral blood lymphocytes (n = 5 in each group). **(d)** Representative flow cytometric plots of integrin α4 on the surface of splenic lymphocytes. IgG isotype-labelled cells was used as a negative control. **(e)** The population of integrin α4^{+} cells in splenic lymphocytes (n = 5 in each group). Values are presented as the mean ± SEM. Significance was determined using one-way ANOVA followed by Dunnett's multiple comparison. * $p < 0.05$; *** $p < 0.005$.

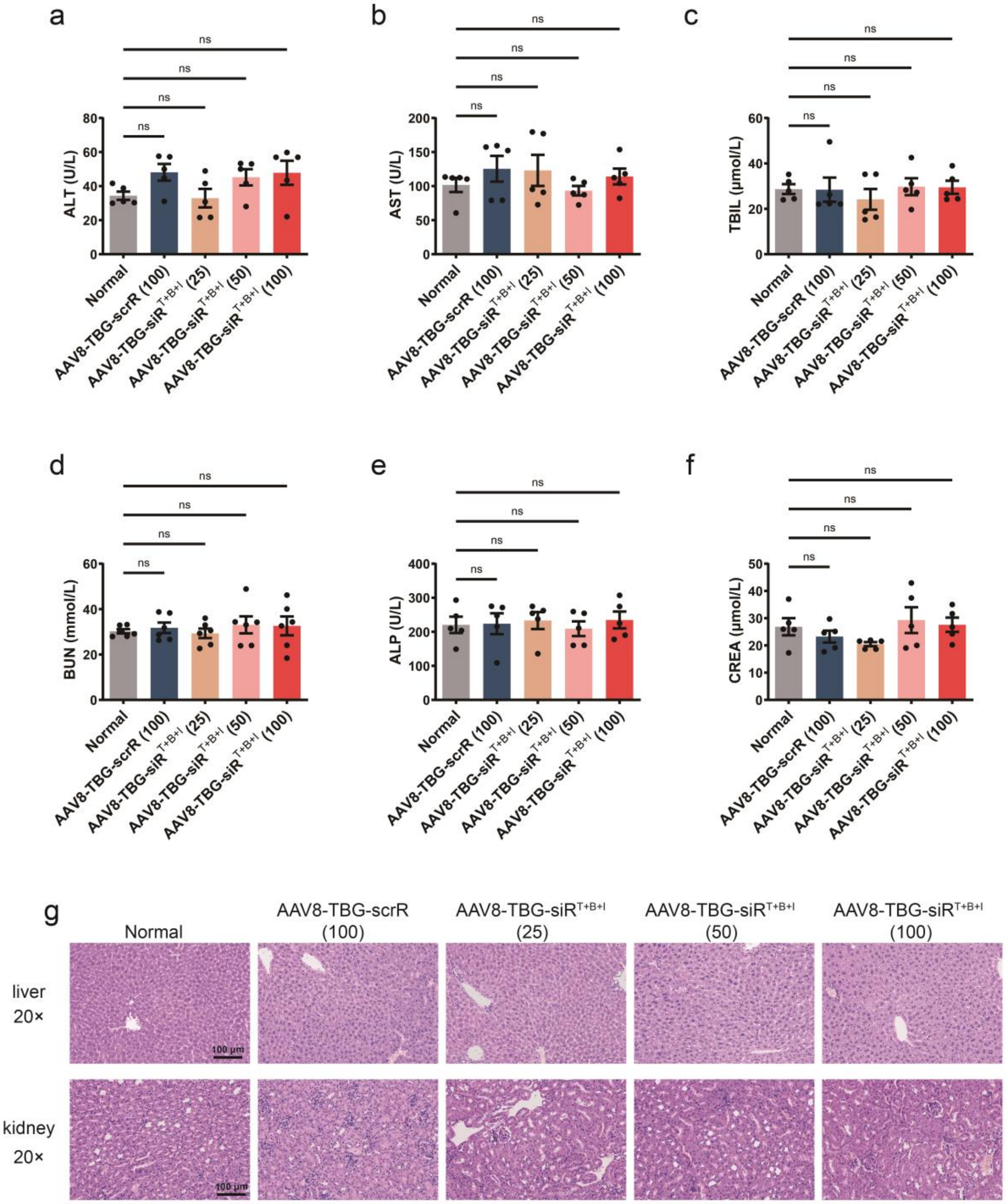

a
ALT (U/L)
b
AST (U/L)
c
TBIL (μmol/L)
d
BUN (mmol/L)
e
ALP (U/L)
f
CREA (μmol/L)
ns
Normal
AAV8-TBG-scrR (100)
AAV8-TBG-siR T+B+I (25)
AAV8-TBG-siR T+B+I (50)
AAV8-TBG-siR T+B+I (100)
g
liver 20×
kidney 20×
100 μm

Supplementary Figure 24. Evaluation of the toxic effects and tissue damage in mice after intravenous injection of AAV8-TBG-siR^{T+B+I}. On week 0, BALB/c mice were intravenously injected with 100 μL AAV8-TBG-scrR (3.0 × 10^{12} V. G/mL) or 25, 50 or 100 μL AAV8-TBG-siR^{T+B+I} (3.0 × 10^{12} V. G/mL). At the same time, chronic UC was induced by rhythmically administering to mice 2.5% DSS for 1 week and water for 2 weeks and the cycle was repeated for 3 times. Untreated BALB/c mice were included as normal controls. After the treatment, mice were sacrificed, and blood and tissue samples were collected and analysed for serum biochemical indicators and tissue damage. **(a-f)** Measurement of representative serum biochemical indicators, including alanine aminotransferase (ALT), aspartate aminotransferase (AST), total bilirubin (TBIL), blood urea nitrogen (BUN), alkaline phosphatase (ALP) and creatinine (CREA), in the serum (n = 5 in each group). **(g)** Histological examination of the liver and kidney. Scale bar: 100 μm. Values are presented as the mean ± SEM. Significance was determined using one-way ANOVA followed by Dunnett's multiple comparison. NS, not significant.

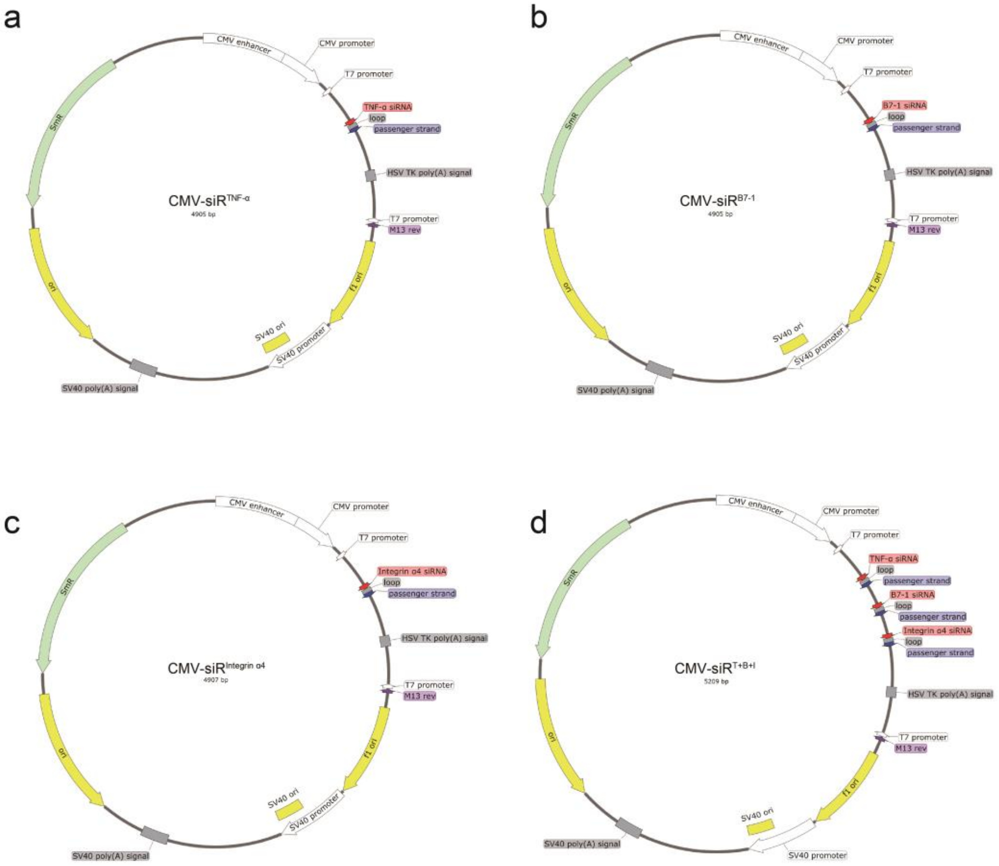

Supplementary Figure 25. Maps of the plasmids used to express genetic circuits. (a) CMV-siR$^{\text{TNF-}\alpha}$ circuit. **(b)** CMV-siR$^{\text{B7-1}}$ circuit. **(c)** CMV-siR$^{\text{Integrin }\alpha 4}$ circuit. **(d)** CMV-siR$^{\text{T+B+I}}$ circuit.

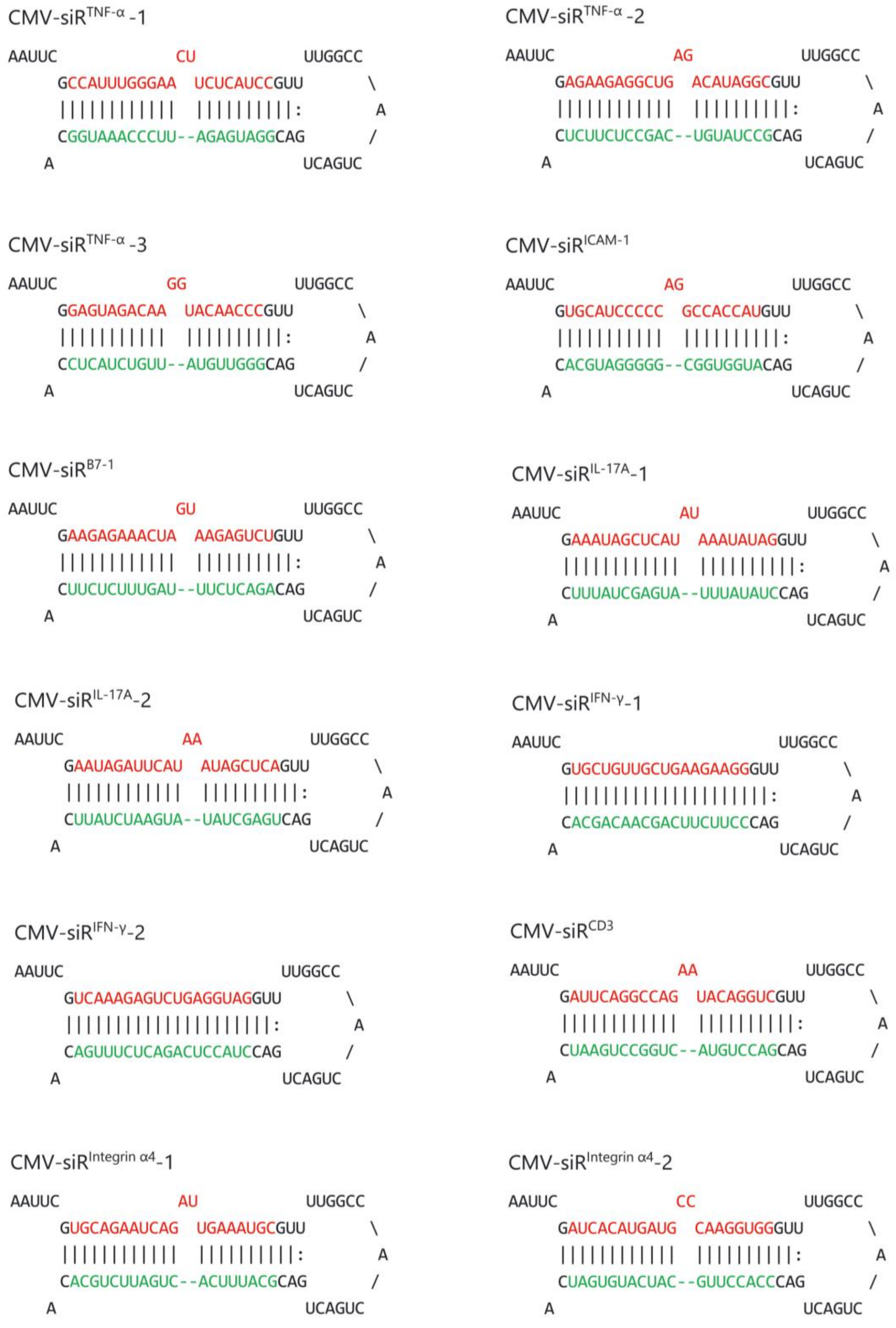

Supplementary Figure 26. Sequences of siRNA expression cassettes. Sequences of the siRNA expression cassettes designed for silencing of TNF-α, IL-17A, IFN-γ, IL-6, integrin α4, ICAM-1, CD3 and B7-1 are shown. Guide strands are marked in red, and passenger strands are marked in green.

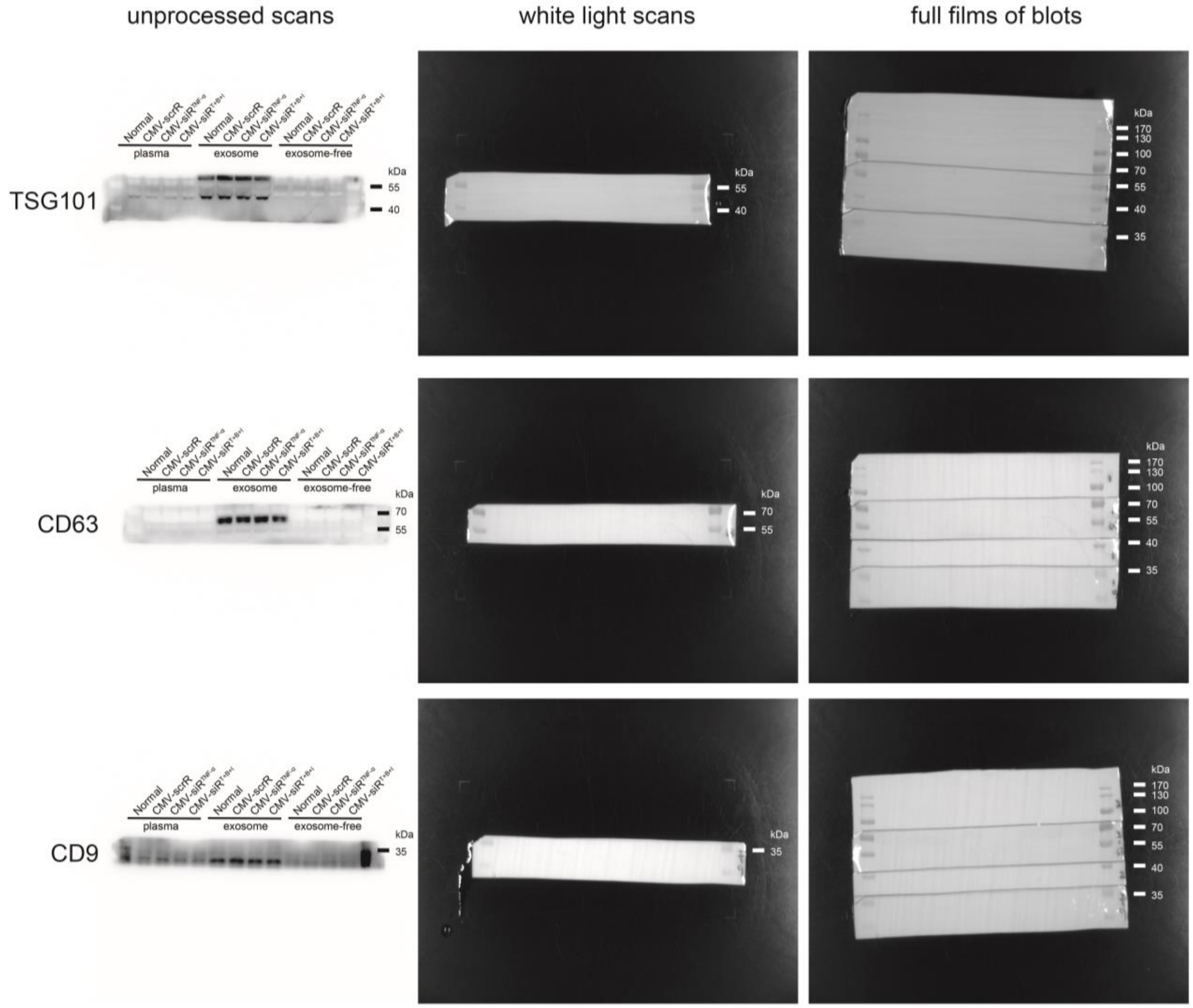

Supplementary Figure 27. Original Western blot images for Supplementary Figure 4c. Western blot analysis was performed to determine specific sEV markers (TSG101, CD63 and CD9) in whole plasma, purified sEVs and sEV-free plasma. An equal amount of total protein was loaded in each lane. Whole uncropped images of the original western blots and the raw, unprocessed scans are shown.

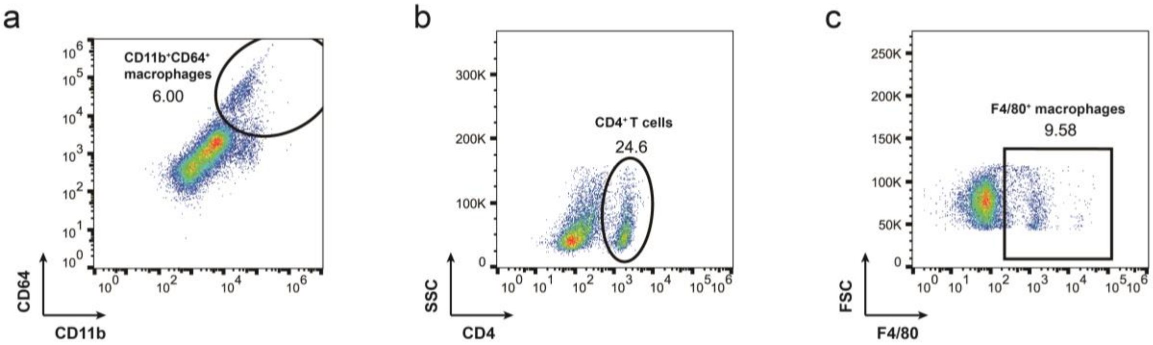

Supplementary Figure 28. Gating strategy. (a) Gating strategy to obtain $CD11b^+CD64^+$ macrophages. Strategy applies to data generated in Figure 5j. **(b)** Gating strategy to obtain $CD4^+$ T cells. Strategy applies to data generated in Figure 5l, 5n and 8h and Supplementary Figure 14g, 15a, 15c, 23a, 23b and 23d. **(c)** Gating strategy to obtain $F4/80^+$ macrophages. Strategy applies to data generated in Figure 8d and Supplementary Figure 13c, 14a, 14c, 22a, 22b and 22d.

Supplementary Tables

Supplementary Table 1. Comparison of the therapeutic effects between genetic circuits and infliximab (IFX).

Models	Groups	Body weight	Colon length	DAI	Expression of pro-inflammatory cytokines	Pathological evaluation
DSS-induced acute UC model	CMV-siR$^{TNF\text{-}\alpha}$ *vs.* IFX	≈	>	>	>	<
TNBS-induced acute colitis model	CMV-siR$^{TNF\text{-}\alpha}$ *vs.* IFX	>	>	>	\	\
DSS-induced chronic UC model	CMV-siR$^{TNF\text{-}\alpha}$ *vs.* IFX	>	>	>	>	>
TNBS-induced chronic colitis model	CMV-siR$^{TNF\text{-}\alpha}$ *vs.* IFX	>	≈	>	>	>
IL-10$^{-/-}$ mice with spontaneous chronic colitis	CMV-siR$^{TNF\text{-}\alpha}$ *vs.* IFX	≈	>	\	>	<
IL-10$^{-/-}$ mice with spontaneous chronic colitis	CMV-siR^{T+B+I} *vs.* IFX	≈	>	\	>	>
DSS-induced chronic UC model	AAV8-TBG-siR^{T+B+I} *vs.* IFX	≈	>	>	≈	≈

>: Plasmid-based or AAV-driven genetic circuits perform better than IFX.

<: Plasmid-based or AAV-driven genetic circuits perform worse than IFX.

≈: Plasmid-based or AAV-driven genetic circuits perform same as IFX.

\: not available.

Supplementary Table 2. Sequences of the siRNAs designed for silencing of TNF-α, IL-17A, INF-γ, IL-6, integrin α4, ICAM-1, CD3 and B7-1.

Name	siRNA sequences (5’ - 3’)
CMV-siR$^{\text{TNF-}\alpha}$-1	CCATTTGGGAACTTCTCATCC
CMV-siR$^{\text{TNF-}\alpha}$-2	AGAAGAGGCTGAGACATAGGC
CMV-siR$^{\text{TNF-}\alpha}$-3	GAGTAGACAAGGTACAACCC
CMV-siR$^{\text{ICAM-1}}$	TGCATCCCCCAGGCCACCAT
CMV-siR$^{\text{B7-1}}$	AAGAGAAACTAGTAAGAGTCT
CMV-siR$^{\text{IL-17A}}$-1	AAATAGCTCATATAAATATAG
CMV-siR$^{\text{IL-17A}}$-2	AATAGATTCATAAATAGCTCA
CMV-siR$^{\text{IFN-}\gamma}$-1	TGCTGTTGCTGAAGAAGG
CMV-siR$^{\text{IFN-}\gamma}$-2	TCAAAGAGTCTGAGGTAG
CMV-siR$^{\text{IL-6}}$	AGAGCAGAATGAGCTACAGAC
CMV-siR$^{\text{CD3}}$	ATTCAGGCCAGAATACAGGTC
CMV-siR$^{\text{Integrin }\alpha 4}$-1	TGCAGAATCAGATTGAAATGC
CMV-siR$^{\text{Integrin }\alpha 4}$-2	ATCACATGATGCCCAAGGTGG

Supplementary Table 3. Primer sequences.

Name	Primer sequences (5’ - 3’)
TNF-α (forward)	CAGGCGGTGCCTATGTCTC
TNF-α (reverse)	CGATCACCCCGAAGTTCAGTAG
B7-1 (forward)	TCCTGGGCCTGGTCCTTTCA
B7-1 (reverse)	GGGAAACCCCCGGAAGCAAA
Integrin α4 (forward)	GCCAACCGTCGCATCCTGTG
Integrin α4 (reverse)	TCGGTCTGCACCTCGCTTCC
ICAM-1 (forward)	CTGGCAGCAAGTAGGCAAGGAC
ICAM-1 (reverse)	TGGCTGGCGGCTCAGTATCTC
IFN-γ (forward)	CAGGCCATCAGCAACAACATAAGC
IFN-γ (reverse)	AGCTGGTGGACCACTCGGATG
IL-6 (forward)	AGGAGTGGCTAAGGACCAAGACC
IL-6 (reverse)	CTGACCACAGTGAGGAATGTCCAC
CD3 (forward)	GTCTGCGTCTGGTGCCTTCTTC
CD3 (reverse)	CGGCATCGTCCTGGCAAGTG
IL-17A (forward)	TGATGCTGTTGCTGCTGCTGAG
IL-17A (reverse)	TGGAACGGTTGAGGTAGTCTGAGG
GAPDH (forward)	GATATTGTTGCCATCAATGAC
GAPDH (reverse)	TTGATTTTGGAGGGATCTCG
