# Supplementary material for: In vivo self-assembled siRNA as a modality for combination therapy of ulcerative colitis
Source: Nat Commun. 2022 Sep 28;13:5700. doi: 10.1038/s41467-022-33436-0 (PMC9519883; doi:10.1038/s41467-022-33436-0)
Supplement: Supplementary file 3 — Reporting Summary [file 41467_2022_33436_MOESM3_ESM.pdf]

nature portfolio

Corresponding author(s): Xi Chen, Chenyu Zhang and Qipeng Zhang

Last updated by author(s): Aug 20, 2022

Reporting Summary

Nature Portfolio wishes to improve the reproducibility of the work that we publish. This form provides structure for consistency and transparency in reporting. For further information on Nature Portfolio policies, see our Editorial Policies and the Editorial Policy Checklist.

Statistics

For all statistical analyses, confirm that the following items are present in the figure legend, table legend, main text, or Methods section.

n/a	Confirmed	
☐	☒	The exact sample size (*n*) for each experimental group/condition, given as a discrete number and unit of measurement
☐	☒	A statement on whether measurements were taken from distinct samples or whether the same sample was measured repeatedly
☐	☒	The statistical test(s) used AND whether they are one- or two-sided<br>*Only common tests should be described solely by name; describe more complex techniques in the Methods section.*
☒	☐	A description of all covariates tested
☐	☒	A description of any assumptions or corrections, such as tests of normality and adjustment for multiple comparisons
☐	☒	A full description of the statistical parameters including central tendency (e.g. means) or other basic estimates (e.g. regression coefficient) AND variation (e.g. standard deviation) or associated estimates of uncertainty (e.g. confidence intervals)
☐	☒	For null hypothesis testing, the test statistic (e.g. *F*, *t*, *r*) with confidence intervals, effect sizes, degrees of freedom and *P* value noted<br>*Give P values as exact values whenever suitable.*
☒	☐	For Bayesian analysis, information on the choice of priors and Markov chain Monte Carlo settings
☒	☐	For hierarchical and complex designs, identification of the appropriate level for tests and full reporting of outcomes
☒	☐	Estimates of effect sizes (e.g. Cohen's *d*, Pearson's *r*), indicating how they were calculated

Our web collection on statistics for biologists contains articles on many of the points above.

Software and code

Policy information about availability of computer code

Data collection	The images of H&E staining were captured using a microscope (Nikon, Japan). The protein level was visualized using the Tanon 5200 Multi detection system (Tanon, Shanghai, China). The images of western blot data were quantified using ImageJ 1.51j8 software (NIH, Bethesda, MD). The Immunofluorescence were taken with inverted laser scanning confocal microscope TCS SP8 (Leica).
Data analysis	Details on the statistics used can be found in the figure legends. All statistical analyses were performed using commercially available software (GraphPad Prism 9). Data were first checked for a normal distribution, differences among groups were compared by one-way ANOVA, and multiple comparisons were conducted by Dunnett's test. N represents the number of samples used in the experiments. Data are means with error bars showing the SEM. Significance was assumed at *p < 0.05; **p < 0.01; ***p < 0.005. Cells were analysed by flow cytometry on a FACScan flow cytometer(Attune NxT,Thermo Fisher Scientific,America) using FlowJo 10 software.

For manuscripts utilizing custom algorithms or software that are central to the research but not yet described in published literature, software must be made available to editors and reviewers. We strongly encourage code deposition in a community repository (e.g. GitHub). See the Nature Portfolio guidelines for submitting code & software for further information.

Data

Policy information about availability of data

All manuscripts must include a data availability statement. This statement should provide the following information, where applicable:

- Accession codes, unique identifiers, or web links for publicly available datasets
- A description of any restrictions on data availability
- For clinical datasets or third party data, please ensure that the statement adheres to our policy

All relevant data generated for this study are included in the article/Supplementary Material/Source Data File. Other data/materials that support the findings of this study are readily available from the corresponding author upon reasonable request. Source data are provided with this paper.

Field-specific reporting

Please select the one below that is the best fit for your research. If you are not sure, read the appropriate sections before making your selection.

☒ Life sciences ☐ Behavioural & social sciences ☐ Ecological, evolutionary & environmental sciences

For a reference copy of the document with all sections, see nature.com/documents/nr-reporting-summary-flat.pdf

Life sciences study design

All studies must disclose on these points even when the disclosure is negative.

Sample size	The sample sizes chosen are consistent with previously published works in the field (Wirtz S, Popp V, Kindermann M, Gerlach K, Weigmann B, Fichtner-Feigl S, Neurath MF. Chemically induced mouse models of acute and chronic intestinal inflammation. Nat Protoc. 2017 Jul;12(7):1295-1309. doi: 10.1038/nprot.2017.044. Epub 2017 Jun 1. PMID: 28569761.).
Data exclusions	No data was excluded from the analyses.
Replication	All replication attempts were successful. For quantitative RT-PCR, western blot, exosome isolation, immunoprecipitation assay,Flow cytometry assay ,E-Cadherin adhesion assay,In situ hybridization of siRNA assay and ELISA, we performed three independent experiments and obtained similar results. For in vivo experiments, we have included sample sizes consistent with previously published works in the field.The experimental findings were reproduced in multiple independent experiments. The number of independent experiments and biological replicates in each data panel is indicated in the figure legends.
Randomization	Mice/cells were assigned randomly into experimental groups and processed in an arbitrary order. Animals were numbered and randomly divided into groups according to a random number table.
Blinding	All the investigators were blinded to group allocation during data collection and analysis.

Reporting for specific materials, systems and methods

We require information from authors about some types of materials, experimental systems and methods used in many studies. Here, indicate whether each material, system or method listed is relevant to your study. If you are not sure if a list item applies to your research, read the appropriate section before selecting a response.

Materials & experimental systems

n/a	Involved in the study	
☐	☒	Antibodies
☐	☒	Eukaryotic cell lines
☒	☐	Palaeontology and archaeology
☐	☒	Animals and other organisms
☒	☐	Human research participants
☒	☐	Clinical data
☐	☒	Dual use research of concern

Methods

n/a	Involved in the study	
☒	☐	ChIP-seq
☐	☒	Flow cytometry
☒	☐	MRI-based neuroimaging

Antibodies

Antibodies used	CD63 antibody (Proteintech, 25682-1-AP,1:1000 dilution for WB, lot number:lot number: 00059205)<br>CD9 antibody (Proteintech, 20597-1-AP,1:1000 dilution for WB)<br>TSG101 antibody (Proteintech, 28283-1-AP,1:1000 dilution for WB)<br>Anti-Rabbit IgG (H+L) (Proteintech, SA00001-2, 1:2000 dilution for WB, lot number: 20000003)<br>F4/80 Monoclonal Antibody (Invitrogen,11-4801-81)

B7-1 (Abcam,ab93507, lot number:GR33715921-1)
integrin α4 (Abcam,ab202969,lot number:GR3201534-14)
CD25 (Abcam,ab86908,1:500 dilution for IHC)
TNF-α (Abcam, ab183218,lot number:GR3341243-9)
B7-1 (Abcam, ab254579,lot number:GR3301023-6)
integrin α4 (Cell Signalling Technology, 8440S,lot number:1)
F4/80 (Abcam, ab60343,lot number:GR3248881-12)
CD4 (Proteintech, CL488-65141,lot number:210001469)
CoraLite594 – conjugated Goat Anti-Rabbit IgG(H+L) (Proteintech, SA00013-4)
anti-CD11b-PerCP (clone M1/70,Biolegend,101230,lot number:B334907)
αCD64-PE (clone X54-5/7.1,Biolegend,139304,lot number:B341637)
αCD206-AF488 (clone C068C2,Biolegend,C068C2)
anti-Ly6C-APC (clone HK1.4,128016,Biolegend,lot number:B225258)
anti-digoxigenin (Roche, 11207741910)
Anti-Mouse CD4-PerCP-Cy5.5(MultiSciences(Lianke)Biotech Co. Ltd,AM00407-25,lot number:11132)
Anti-Mouse IFN-γ-PE(MultiSciences(Lianke)Biotech Co. Ltd,AM0IF04-100, lot number:11015)
Argonaute 2 (C34C6) Rabbit mAb #2897(Cell Signalling Technology, 2897,1:1000 dilution for WB, lot number:8)

Validation

CD63 antibody https://www.ptgcn.com/products/CD63-Antibody-25682-1-AP.htm
CD9 antibody https://www.ptgcn.com/products/CD9-Antibody-20597-1-AP.htm
TSG101 antibody https://www.ptgcn.com/products/TSG101-Antibody-28283-1-AP.htm
Anti-Rabbit IgG (H+L) https://www.ptgcn.com/products/HRP-conjugated-Affinipure-Goat-Anti-Rabbit-IgG-H-L-secondary-antibody.htm
F4/80 Monoclonal Antibody https://www.thermofisher.cn/cn/zh/antibody/product/F4-80-Antibody-clone-BM8-Monoclonal/11-4801-81
B7-1 https://www.abcam.cn/pe-cd80-antibody-16-10a1-ab93507.html
integrin α4 https://www.abcam.cn/integrin-alpha-4cd49d-antibody-ab202969.html
CD25 https://www.abcam.cn/il-2-receptor-alpha-antibody-pc61-ab86908.html
TNF-α https://www.abcam.cn/tnf-alpha-antibody-epr19147-ab183218.html
B7-1 https://www.abcam.cn/cd80-antibody-ab254579.html
integrin α4 https://www.cellsignal.cn/products/primary-antibodies/integrin-a4-d2e1-xp-rabbit-mab/8440?site-search-type=Products&N=4294956287&Ntt=8440s&fromPage=plp&_requestid=2194049
F4/80 https://www.abcam.cn/fitc-f480-antibody-bm8-ab60343.html
CD4 https://www.ptgcn.com/products/CD4-Antibody-CL488-65141.htm
CoraLite594 – conjugated Goat Anti-Rabbit IgG(H+L) https://www.ptgcn.com/products/CoraLite594-conjugated-Goat-Anti-Rabbit-IgG-H-L.htm
anti-CD11b-PerCP https://www.biolegend.com/en-us/products/percp-anti-mouse-human-cd11b-antibody-4315
αCD64-PE https://www.biolegend.com/en-us/products/pe-anti-mouse-cd64-fcgammari-antibody-6691
αCD206-AF488 https://www.biolegend.com/en-us/products/alexa-fluor-488-anti-mouse-cd206-mmr-antibody-7426
anti-Ly6C-APC https://www.biolegend.com/en-us/products/apc-anti-mouse-ly-6c-antibody-6047
anti-digoxigenin https://www.biocompare.com/9776-Antibodies/192277-Sheep-AntiDigoxigenin-Fab-fragments-Antibody-Fluorescein-Conjugated/
Anti-Mouse CD4-PerCP-Cy5.5 http://www.liankebio.com/product-653142.html
Anti-Mouse IFN-γ-PE http://www.liankebio.com/product-653348.html
Argonaute 2 (C34C6) Rabbit mAb #2897 https://www.cellsignal.cn/products/primary-antibodies/argonaute-2-c34c6-rabbit-mab/2897?site-search-type=Products&N=4294956287&Ntt=ago2&fromPage=plp

Eukaryotic cell lines

Policy information about cell lines

Cell line source(s)

The mouse macrophage cell line ANA-1 (CL-0023) were purchased from Procell Life Science&Technology Co.,Ltd (Wuhan, China). Human embryonic kidney cell line HEK293T (GNHu 43) were purchased from the Shanghai Institute of Cell Biology, Chinese Academy of Sciences (Shanghai, China).

Authentication

All cell lines were identified by STR (Short Tandem Repeat) profiling by the source. Cells were expanded after being received and subsequently stored in liquid nitrogen. The storage vials were thawed for experiments and used within 2 months.

Mycoplasma contamination

Regular mycoplasma test showed both cell lines were mycoplasma negative.

Commonly misidentified lines
(See ICLAC register)

No commonly misidentified cell line was used in this study.

Animals and other organisms

Policy information about studies involving animals; ARRIVE guidelines recommended for reporting animal research

Laboratory animals

Six-week-old male BALB/c mice were purchased from the Model Animal Research Center of Nanjing University. Eleven-week-old male IL-10-/- mice and wild-type C57BL/6 mice were kindly gifted by Prof. Zhen Huang (BioMed Laboratory, Nanjing University, China). All the animals were maintained under specific pathogen-free conditions at Nanjing University. All the animal experiments were approved by Animal Ethical and Welfare Committee of Nanjing University (approval number IACUC-2011006). The animals were

	housed under specific pathogen-free conditions, maintained in a temperature-controlled room with a 12h light/dark cycle, and provided ad libitum access to water and standard laboratory chow.
Wild animals	The study did not involve wild animals
Field-collected samples	The study did not involve samples collected from the field
Ethics oversight	All experiments were approved by the Animal Ethical and Welfare Committee of NJU

Note that full information on the approval of the study protocol must also be provided in the manuscript.

Dual use research of concern

Policy information about dual use research of concern

Hazards

Could the accidental, deliberate or reckless misuse of agents or technologies generated in the work, or the application of information presented in the manuscript, pose a threat to:

No	Yes	
☒	☐	Public health
☒	☐	National security
☒	☐	Crops and/or livestock
☒	☐	Ecosystems
☒	☐	Any other significant area

Experiments of concern

Does the work involve any of these experiments of concern:

No	Yes	
☒	☐	Demonstrate how to render a vaccine ineffective
☒	☐	Confer resistance to therapeutically useful antibiotics or antiviral agents
☒	☐	Enhance the virulence of a pathogen or render a nonpathogen virulent
☒	☐	Increase transmissibility of a pathogen
☒	☐	Alter the host range of a pathogen
☒	☐	Enable evasion of diagnostic/detection modalities
☒	☐	Enable the weaponization of a biological agent or toxin
☒	☐	Any other potentially harmful combination of experiments and agents

Flow Cytometry

Plots

Confirm that:

☒ The axis labels state the marker and fluorochrome used (e.g. CD4-FITC).

☒ The axis scales are clearly visible. Include numbers along axes only for bottom left plot of group (a 'group' is an analysis of identical markers).

☒ All plots are contour plots with outliers or pseudocolor plots.

☒ A numerical value for number of cells or percentage (with statistics) is provided.

Methodology

Sample preparation	Mononuclear cells were isolated from the peripheral blood and spleen by using mouse peripheral blood mononuclear cells separation kit (TBD science, LDS1090, Tianjin, China) and mouse splenic mononuclear extraction kit (TBD science, LDS1090PK), respectively. Lamina propria mononuclear cells were isolated using a method as described previously. Briefly, the colon was removed from the sacrificed mice, cut into 0.5 cm pieces and washed thoroughly with cold PBS to remove all debris and blood. After incubating with 2 mM dithiothreitol and 1 mM EDTA in PBS at 37°C for 2×20 minutes under gentle shaking to remove intestinal epithelial cells, the tissues were digested in 10 mL 2% FBS-RPMI-Collagenase A (1 mg/mL, Roche, Mannheim, Germany) at 37°C for 30 minutes. Lamina propria cells were then collected and further purified via density gradient centrifugation with 40% and 70% Percoll–RPMI solution. Lamina propria mononuclear cells were collected from the interphase.<br>Monocytes were isolated from the peripheral blood mononuclear cells using the peripheral blood monocyte extraction kit (TBD science, TBD2011M). Splenic macrophages and colonic macrophages were isolated by using anti-F4/80 MicroBeads

UltraPure (Miltenyi Biotec, 130-110-443, Bergisch gladbach, Germany) from spleen mononuclear cells and lamina propria mononuclear cell.
Peripheral blood lymphocytes and spleen lymphocytes were enriched using mouse peripheral blood lymphocyte separation kit (TBD science, LTS1092) and mouse splenic lymphocyte extraction kit (TBD science, LTS1092PK). CD4+ T cells were obtained from peripheral blood lymphocytes, spleen lymphocytes andlamina propria mononuclear cells with CD4+ T cell Isolation kit (Miltenyi Biotec, 130-104-454, Bergisch gladbach, Germany).

Instrument

Thermo Fisher Attune NxT Flow Cytometer

Software

Flow cytometry on a FACScan flow cytometer using FlowJo 10 software

Cell population abundance

Lamina propria mononuclear cells with about 6% positive for CD11b+CD64+macrophages.
Peripheral blood mononuclear cells with about 10% positive for F4/80+ monocytes, spleen mononuclear cells with about 30% positive for F4/80+ macrophages, lamina propria mononuclear cells with about 25% positive for F4/80+ macrophages.
Peripheral blood mononuclear cells with about 5% positive for CD4+ T cells, spleen lymphocytes with about 20% positive for CD4+ T cells, lamina propria mononuclear cells with about 40% positive for CD4+ T cells.

Gating strategy

The results of flow cytometric plots of M1-like (Ly6ChighCD206low) and M2-like (Ly6ClowCD206high) macrophages had been obtained using the ‘’CD11b+CD64+’’ gating strategy.
The results of flow cytometric plots of Th1 cells had been obtained using the ‘’CD4+ / IFN-γ+’’ gating strategy.
The results of flow cytometric plots of Th17 cells had been obtained using the ‘’CD4+ / IL-17A+’’ gating strategy.
The results of flow cytometric plots of B7-1 had been obtained using the ‘’F4/80+ /B7-1+’’ gating strategy.
The results of flow cytometric plots of integrin α4+ had been obtained using the ‘’CD4+ / integrin α4+’’ gating strategy.
The boundaries between positive and negative staining cell populations had been shown in the corresponding figure.

☒ Tick this box to confirm that a figure exemplifying the gating strategy is provided in the Supplementary Information.

March 2021
