# Supplementary material for: In vivo self-assembled siRNA as a modality for combination therapy of ulcerative colitis
Source: Nat Commun. 2022 Sep 28;13:5700. doi: 10.1038/s41467-022-33436-0 (PMC9519883; doi:10.1038/s41467-022-33436-0)
Supplement: Supplementary file 2 — Peer Review File [file 41467_2022_33436_MOESM2_ESM.pdf]

nature portfolio

Peer Review File

Title: In vivo self-assembled siRNA as a new modality for combination therapy of ulcerative colitis

Open Access This file is licensed under a Creative Commons Attribution 4.0 International License, which permits use, sharing, adaptation, distribution and reproduction in any medium or format, as long as you give appropriate credit to the original author(s) and the source, provide a link to the Creative Commons license, and indicate if changes were made. In the cases where the authors are anonymous, such as is the case for the reports of anonymous peer reviewers, author attribution should be to 'Anonymous Referee' followed by a clear attribution to the source work. The images or other third party material in this file are included in the article's Creative Commons license, unless indicated otherwise in a credit line to the material. If material is not included in the article's Creative Commons license and your intended use is not permitted by statutory regulation or exceeds the permitted use, you will need to obtain permission directly from the copyright holder. To view a copy of this license, visit http://creativecommons.org/licenses/by/4.0/.

REVIEWER COMMENTS</B>

Reviewer #1 (Remarks to the Author):

The manuscript by Zhou et al. describes an siRNA-mediated approach for combination therapy for ulcerative colitis. After expressing a combination of siRNAs in the livers of mice, extracellular vesicles (exosome)-mediated intercellular transfer of siRNAs was hypothesized to lead to silencing of a combination of genes in the colon, with therapeutic benefits as a result. The technology/approach has been published before by this group (PMID 33782530, as correctly cited), which somewhat limits the novelty of the current work. However, the work is timely, well written, and therapeutic efficacy convincingly shown in multiple disease models in mice. Mechanistically however, the hypothesis suggested by the authors (i.e. siRNA is transferred from liver to colon via extracellular vesicles) is only weakly supported by data.

Major comments:

- The authors claim that the CMV promotor module directs packaging of siRNA into EVs. However, no evidence for this statement is shown. Do the authors truly mean packaging (as in sorting), or do they simply mean that CMV drives expression? In the latter case, please change the wording.
- Plasmid injection. What was the volume of injection of circuit plasmids? Is this a hydrodynamic injection?
- EV isolation. The authors isolate EVs using an Exosome Isolation kit, which is known to yield EVs of low purity, especially when purified from plasma samples. Therefore, in order to be able to claim that high siRNA levels in plasma (2000 fM!) are truly associated to exosomes/EVs (Fig 2C, almost 1500 fM!), the authors need to show 1) siRNA levels after purifying exosomes/EVs further using e.g. bead pull downs or density gradient centrifugation and 2) demonstrating that siRNA is protected from RNAses in plasma in the absence but not presence of detergents that destroy exosomes/EVs.
- Figure 2f/h. Concentrations of siRNA in colon and spleen are cumulatively similar or even higher than in liver. This is difficult to envision using the model proposed by the authors, where (1) only a small fraction of siRNA will be packaged into exosomes/EVs and secreted, and (2) those siRNAs will be further diluted and distributed throughout the body. A more logical explanation would be that circuit DNA has been delivered into colon or spleen or to immune cells directly that have homed to sites of inflammation. Can the authors demonstrate that their plasmid DNA (hydrodynamic) injection does not lead to expression of siRNA outside liver, especially in this model of high inflammation?

Minor comments:

- Terminology: Exosomes are a multivesicular body-derived subtype of extracellular vesicles. Without demonstrating the intracellular origin of vesicles studied, the general term ‘extracellular vesicle’ seems more appropriate.
- Abstract: The final sentence (after requiring) is ended abruptly.
- Suppl Table 2. The differences between the sequences of siRNA expression cassettes listed in this table, as compared to the CMV-circuit vector as described in the methods, are unclear and should be clearly explained.
- Suppl Table 2. Sense and antisense sequences are identical for CMV-siRTNFa-1.
- Suppl figure 1a. In the figure, it seems LPS is also present in the condition ‘normal’, which I guess is not

the case.
- Figure 2D. What was the final concentration of siRNA added here?
- Line 377, “a synergistic advantage”. Wouldn’t this effect simply be additive?

Reviewer #2 (Remarks to the Author):

In this study, authors have designed a synthetic biology strategy that integrates the naturally existing exosome-circulating system with artificial genetic circuits for self-assembly and delivery of multiple siRNAs for the combination therapy of experimental colitis model in mice. They found that this strategy effectively prevented intestinal inflammation and had a synergistic therapeutic effect against intestinal mucosal inflammation via suppressing the proinflammatory cascade in colonic macrophages, inhibiting the costimulatory signal to T cells and blocking T cell homing to sites of inflammation. However, several points need to be addressed.

1. Organ-targeting self-assembled siRNA therapy has been conducted in different disease models, and the therapeutic potential of the RNAi is also used in an experimental colitis model of IBD by targeting colon macrophages to reduce inflammatory symptoms (Nat Nanotechnol 2018 03;13(3)). It appears that this concept is not novel enough.
2.Authors focused on TNF-α as the therapeutic target to alleviate experimental colitis in a DSS-induced acute colitis mice. What about another proinflammatory cytokines, e.g., IL-6, IL-17A, IL-12, IL-23 ?
3.Did the CMV-siRTNF-α treatment show apparently advantage compared to anti-TNF-a mAb treatment (e.g., IFX or adalimumab)? It is necessary to clarify the differences between two kinds of therapy in body weight, colon length, DAI, and expression of proinflammatory genes or proteins in inflamed colon.
4.After CMV-siRTNF-α treatment, what about the functional alterations of macrophages besides reducing the production of TNF-α? Or, in detail, whether CMV-siRTNF-α treatment could affect the differentiation and polarization of the macrophages in the inflamed colon?
5.TNF-α could be produced from other immune cells, e.g., CD4+ T cells. Previous studies have demonstrated that IFX treatment could modulate functional changes of CD4+ T cells, e.g., enhancing the secretion of IL-22. Therefore, CMV-siRTNF-α treatment might also affect the functional changes or differentiation of CD4+ T cells in inflamed mucosa? Authors need to have a analysis on phenotypic changes of mucosal CD4+ T cells.
6.DSS-induced acute colitis model in mice is not an immune cell-mediated model. Authors need to replicate the CMV-siRTNF-α treatment in spontaneous colitis model or CD4+ T cell-induced chronic colitis model, such as in Rag-/- mice reconstituted with CD45RBhigh CD4+ T cells.

Reviewer #3 (Remarks to the Author):

In their manuscript “In vivo self-assembled siRNA: a new modality for combination therapy of ulcerative colitis”, Zhou et al. examined a possible approach for a combination therapy for UC. Injection of DNA

plasmids reprogrammed the host liver to produce exosomes that contained siRNAs against TNFa, Integrin a4 and B7-1, three molecules with implication in the continuous inflammation in UC. With this approach the authors managed to significantly decrease inflammation in two mouse models of UC.

Overall advance to the field

Current treatments for inflammatory bowel disease relieve inflammation only in a limited number of people and continuous treatment can result in therapy-resistance. Hence, new strategies for the treatment of UC and CD are urgently needed. Efforts of targeting TNFa by siRNA were hampered by inefficient delivery of the siRNA to the target cell. In a previous paper, Zhou et al. developed an approach that allows the self-assembly and delivery of siRNA containing exosomes. In the current manuscript, the authors successfully applied this approach for the treatment of experimental UC. In addition, the efficiency of the clinically more relevant AAV-circuit was successfully tested in the present manuscript. Overall, Zhou et al.'s approach for a possible combination therapy for the treatment of UC is very interesting and innovative.

Major comments

The methods used in the manuscript are appropriate and the results achieved by using the DSS colitis model were confirmed by adding the TNBS-model as a second colitis model.

The conclusions are justified and supported by the data provided.

When using the AAC genomic circuit, the authors used a prophylactic treatment approach as they treated healthy mice with the circuit and 3 weeks later induced UC. While this is an interesting first step, it would be important to evaluate the therapeutic value of the AAC genomic circuit by inducing UC first and then treating the mice with the genomic circuit. The authors successfully tested a therapeutic approach for the use of the CMV siRNA circuit. This should also be done with the AAV-circuit.

In the mouse model, the use of a CMV siRNA circuit and more importantly of the AAV circuit did not lead to any cell toxicity or other side effects. However, considering that only the highest dose of genetic circuits resulted in an improved outcome compared to infliximab, could the authors state whether there are any known concerns when translating this approach into the clinic. Especially with regard to the clinically more relevant AAV-circuit, it would be interesting if administration of the required high doses induces issues. Also, are severe side effects known when using a long-term treatment approach?

Minor comments:

Figure 5: The legend currently describes an acute UC model, however, based on Fig 5a it should possibly say "the cycle was repeated for 3 times" (line 708)

Figure 5: Suppression of B7-1 and Integrin a4 is shown as suppression of mRNA. For completion,

addition of the flow data to show suppression of protein expression as done in Fig. 4 would be recommended.

Sup Fig. 7: Toxicity should be measured at the same concentration as in the actual experiment (eg. 10mg/kg body weight for the CMV-circuit) instead of only using 5mg/kg body weight.

Sup Fig. 8f: Please use the same graph style for cytokine measurement as in the previous figures.

Figure 2 shows that apart from the colon, siRNA loaded exosomes can also be detected in the kidney, spleen, and peripheral blood monocytes and CD4+ T-cells. While experimental animals in this study were kept under pathogen-free conditions, there is a certain risk that the triple siRNA treatment renders animals/patients highly susceptible to opportunistic infections. This concern will have to be addressed when future research in this direction is considered.

Reviewer #4 (Remarks to the Author):

This is an interesting report of the therapeutic efficacy of a novel anti-inflammatory combination therapy based on multiple siRNA delivery from either a single plasmid or a single AAV vector in a mouse model of ulcerative colitis (UC). Although the delivery platform/approach is not novel (the authors describe as “circuit” and “synthetic biology approach” what in reality are either an expression cassette or a multicistronic vector, respectively), the translational potential is high given the significant therapeutic effect observed. The study is well designed and executed.
Below are some comments:
-The conclusions that the mechanism underlying the significant therapeutic effect observed is liver-mediated transduction and secretion of exosomes seems stretched. To conclude this the authors should have used a liver-specific promoter rather than CMV, and AAV8 that targets primarily mouse hepatocytes upon iv injection rather than AAV9 which diffuses quite broadly. In the absence of data using a liver-specific approach, claims that the effects observed are liver-dependent should be moderated. The authors should acknowledge that transduction of other tissues in addition to liver can contribute to the effect observed. Along this line, a short-term (i.e. 2-3 hours after injection) DNA biodistribution study should be performed to assess which are the tissues originally targeted/infected by either the plasmid or the AAV9 vector, and presumably transduced since the CMV promoter is used.

- The liver siRNA expression/exosome release observed is quite short-lived as observed in Fig. 2B and F. What is the explanation for this? Is plasmid DNA degraded? Does the CMV promoter undergo silencing? Also here, a DNA biodistribution study would help understand the basis of such a pulsatile expression.

Point-by-Point Response to the Reviewers' Comments:

Reviewer #1

1. The authors claim that the CMV promotor module directs packaging of siRNA into EVs. However, no evidence for this statement is shown. Do the authors truly mean packaging (as in sorting), or do they simply mean that CMV drives expression? In the latter case, please change the wording.

<u>Response</u>: We appreciate reviewer's emphasis on this critical issue. We totally agree with the reviewer that it is misleading to claim that the CMV promotor module directs packaging of siRNAs into sEVs. Indeed, the exact mechanism underlying the self-assembly of siRNAs into sEVs is yet to be discovered. In particular, whether the loading and packaging of specific siRNAs into sEVs occur in an active (selective) or passive (random) manner remains an unsettled disputation. CMV promotor is only responsible for driving siRNA expression but is unable to direct sEV biogenesis or mediate sorting of sEV cargoes. Therefore, we have changed the wording throughout the revised manuscript. However, we did observe a dose-dependent increase of intracellular TNF-α siRNA levels when an increased amount of CMV-siR$^{TNF\text{-}\alpha}$ circuit was transfected into HEK293T cells (Supplementary Fig. 1c), which was accompanied by a dose-dependent increase of TNF-α siRNA in secreted sEVs (Supplementary Fig. 1d). Based on this, another possibility is that the CMV promoter drives the transcription and accumulation of siRNAs in the cytoplasm of hepatocytes, which leads to the loading and packaging of saturated cytoplasmic siRNAs into sEVs as cargo.

We have added a whole paragraph to the Discussion section of the revised manuscript to emphasize this critical issue, which reads as follows: "However, the exact mechanism underlying the self-assembly of siRNAs into sEVs is not known. Whether the loading and packaging of specific siRNAs into sEVs occur in an active (selective) or passive (random) manner remains controversial. Recent studies on secretory miRNAs may provide some inspiration for this question. There is mounting evidence that cells selectively package certain miRNAs into EVs for active secretion.[1-3] However, the mechanism by which miRNAs are sorted into EVs or retained in cells remains elusive. EVs are a heterogeneous group of endogenous nanosized membrane vesicles that are generally categorised into two subtypes: exosomes (30–100 nm in diameter) that are derived from the luminal membrane of multivesicular bodies (MVBs) and released via exocytosis and microvesicles (100–1000 nm in diameter) that are shed directly from the plasma membrane.[4] Because of the heterogeneous nature of EVs, the molecular mechanisms responsible for the sorting and packaging of siRNAs into EVs must be quite diverse depending on the biogenesis routes, cargo compositions and functional specialisation of different EV subtypes. To simplify this complex diversity, we only used exosomes and exosome-enclosed siRNAs as examples. Exosome generation includes the following steps: (i) the cytoplasmic membrane invaginates to form an early endosome; (ii) the payload sprouts inward to form intraluminal vesicles contained within the endosome and creates a structure classically described as MVB; and (iii) intraluminal vesicles are released to the extracellular space as exosomes upon fusion of the MVB with the plasma membrane.[5,6] During the exosome formation process, materials from the

cell cytoplasm, including proteins and miRNAs, are specifically sorted and encapsulated into intraluminal vesicles, which are the precursors of exosomes. In our strategy, TNF-α/B7-1/integrin α4 siRNA was embedded in a pre-miR-155 backbone, which borrowed the endogenous miRNA processing machinery of the host liver to produce siRNAs and self-assemble siRNAs into secretory sEVs.[7] Therefore, the primary means of siRNA loading into exosomes may be equivalent to miRNAs. However, recent studies identified some specific short motifs in mature miRNAs that control miRNA sorting into exosomes.[8] Although these findings emphasise the importance of the miRNA sequence for material cargo sorting, the siRNA sequences designed for the silencing of TNF-α, B7-1 and integrin α4 did not harbour these motifs, which excludes the possibility of sequence-mediated selective sorting of siRNAs into exosomes. However, we observed a dose-dependent increase in intracellular TNF-α siRNA levels when an increased amount of CMV-siR$^{TNF-\alpha}$ circuit was transfected into HEK293T cells, which was accompanied by a dose-dependent increase in TNF-α siRNA in secreted sEVs. These results suggest another possibility in which the CMV promoter drives the transcription and accumulation of siRNAs in the cytoplasm of hepatocytes and leads to the loading and packaging of saturated cytoplasmic siRNAs into sEVs as cargo. Taken together, the molecular mechanisms underlying the selective or passive assembly of siRNAs into sEVs have not been elucidated. Because the promoter, siRNA sequence and pre-miRNA structure may join together to decide the sorting route of siRNAs to sEVs in the genetic circuit design, there is much room for optimising the biodistribution of siRNAs to guarantee the preferential sorting of siRNAs into sEVs rather than retaining them within cells."

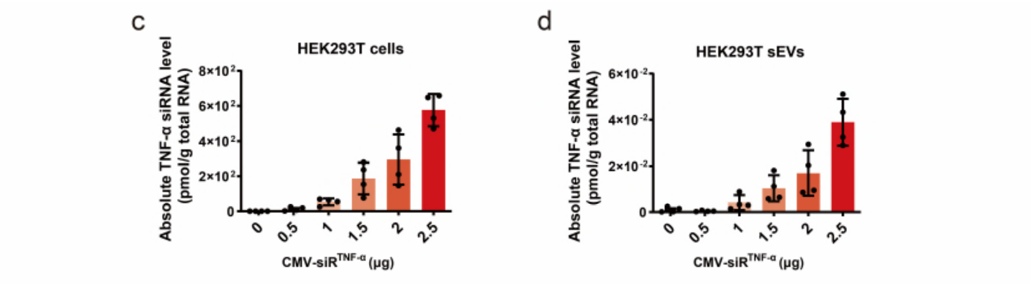

Supplementary Figure 1. (c) Quantitative RT-PCR analysis of TNF-α siRNA levels in HEK293T cells transfected with increasing dose of CMV-siR$^{TNF-\alpha}$ circuit (n = 4 in each group). **(d)** Quantitative RT-PCR analysis of TNF-α siRNA levels in the sEVs derived from the culture medium of HEK293T cells transfected with increasing dose of CMV-siR$^{TNF-\alpha}$ circuit (n = 4 in each group). Values are presented as the mean ± SEM.

2. Plasmid injection. What was the volume of injection of circuit plasmids? Is this a hydrodynamic injection?

<u>Response</u>: We apologize for not indicating this issue clearly. For hydrodynamic injection, animals are usually injected via the tail vein with a volume equivalent to 10% of the body weight in 5 seconds. In fact, we used a non-invasive normal tail vein injection procedure (100 μL solution was injected in 5 seconds for each mouse) rather than an invasive hydrodynamic injection procedure (1.6-1.8 mL solution was rapidly injected in 5 seconds for each mouse) for injection of

genetic circuits (in the form of DNA plasmids). We have added this information to the Methods section of the revised manuscript.

3. EV isolation. The authors isolate EVs using an Exosome Isolation kit, which is known to yield EVs of low purity, especially when purified from plasma samples. Therefore, in order to be able to claim that high siRNA levels in plasma (2000 fM!) are truly associated to exosomes/EVs (Fig 2C, almost 1500 fM!), the authors need to show 1) siRNA levels after purifying exosomes/EVs further using e.g. bead pull downs or density gradient centrifugation and 2) demonstrating that siRNA is protected from RNAses in plasma in the absence but not presence of detergents that destroy exosomes/EVs.

Response: We appreciate reviewer's constructive suggestions on these issues. In our last version of manuscript, we characterised the physical properties of sEVs purified from the plasma of mice injected with the CMV-scrR or CMV-siR$^{TNF\text{-}\alpha}$ circuit using a commercially available kit. Nanoparticle tracking analysis (NTA) revealed that the plasma sEVs purified from circuit-injected mice had the typical size of nanoparticles (peaked at ~120 nm) and were present at a concentration of ~3.5×10^7 particles/mL, similar to wild-type normal sEVs (Supplementary Fig. 2a). Transmission electron microscopy (TEM) showed that the purified sEVs exhibited a characteristic round vesicular morphology (Supplementary Fig. 2b). Enrichment of sEV markers (TSG101, CD63 and CD9) was detected only in purified sEVs but not in sEV-free plasma (Supplementary Fig. 2c). However, the commercially available kit is known to yield sEVs of low purity. Since density gradient centrifugation is the gold-standard method for achieving sEVs with the highest purity, we purified sEVs from the plasma of mice using density gradient centrifugation and characterised their particle size and morphology using NTA and TEM.[9] The plasma sEVs purified using density gradient centrifugation exhibited typical sEV morphology and size (peaked at ~120 nm) and a similar concentration (~4×10^6 particles/mL) in different groups. However, the sEVs from density gradient centrifugation were typically lower than the yield from the commercially available kit (Supplementary Fig. 2d and 2e). These results reveal that injection of genetic circuits does not affect the size, structure or number of sEVs generated *in vivo*. Subsequently, we measured TNF-α siRNA levels in the plasma sEVs purified from mice injected with the CMV-siR$^{TNF\text{-}\alpha}$ circuit. Notably, most TNF-α siRNA was detected in the plasma sEVs rather than the sEV-free plasma of CMV-siR$^{TNF\text{-}\alpha}$-injected mice, regardless of whether the plasma sEVs were purified using a commercially available kit or density gradient centrifugation (Fig. 2g and 2h). Since TNF-α siRNA was largely encapsulated in sEVs and nearly undetectable in the sEV-free fraction of mouse plasma, TNF-α siRNA may be released into the blood circulation in an sEV-dependent manner.

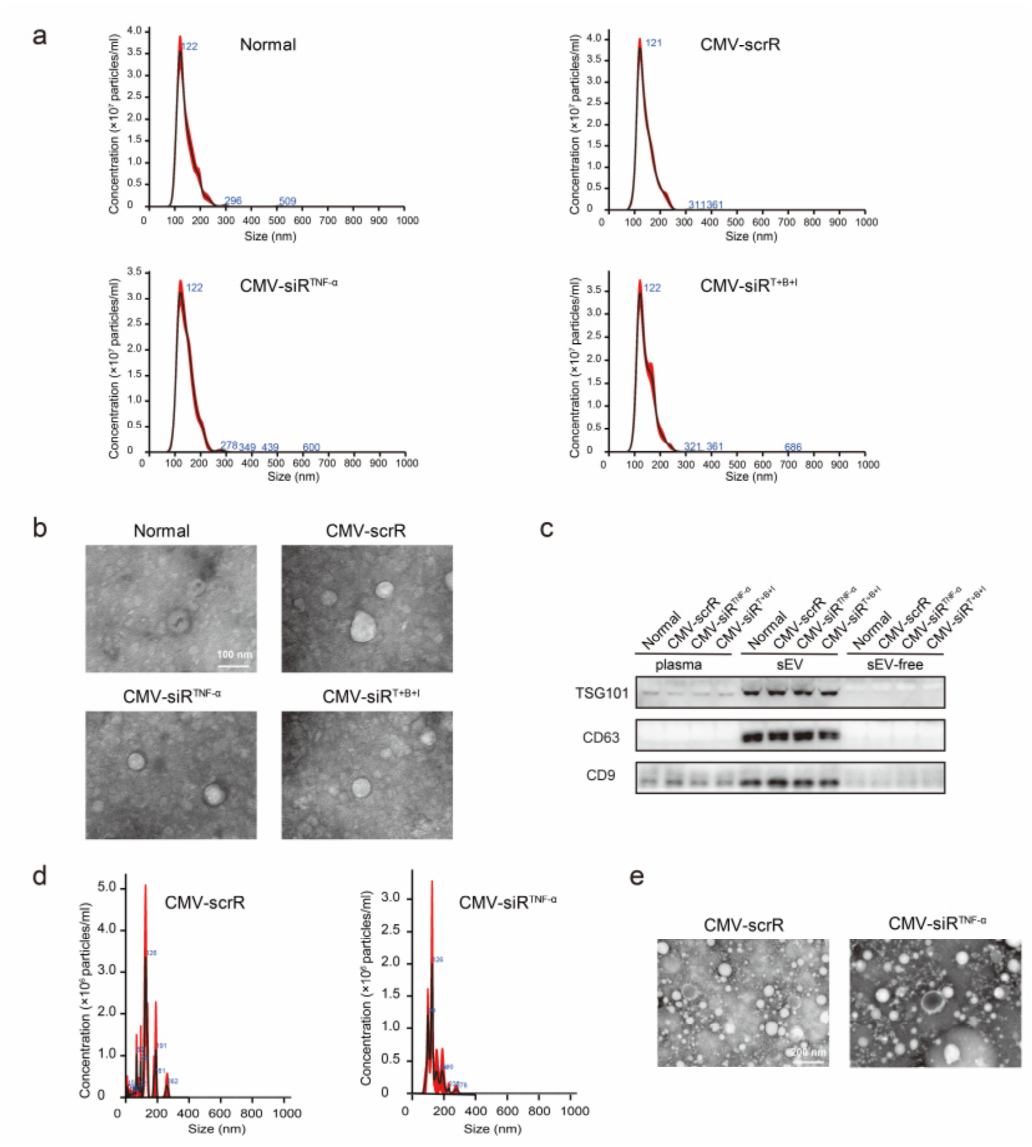

Supplementary Figure 2. Characterisation of the properties of siRNA-encapsulating sEVs. BALB/c mice were intravenously injected with 5 mg/kg CMV-scrR, CMV-siR$^{TNF-\alpha}$ or CMV-siR^{T+B+I} circuit every day for a total of 7 times, and sEVs were then purified by using commercially available kit (a-c) or density gradient centrifugation (d and e) from mouse plasma and characterised using NTA and TEM. The enrichment of sEV markers was analysed by Western blotting. sEVs derived from untreated BALB/c mice were included as normal controls. **(a)** Size distribution and concentration of purified sEVs determined by NTA. **(b)** Representative TEM images of sEVs. Scale bar: 100 nm. **(c)** Western blot analysis of specific sEV markers (TSG101, CD63 and CD9) in whole plasma, purified sEVs and sEV-free plasma. An equal amount of total protein was loaded in each lane. **(d)** Size distribution and concentration of purified sEVs determined by NTA. **(e)** Representative TEM images of sEVs. Scale bar: 100 nm.

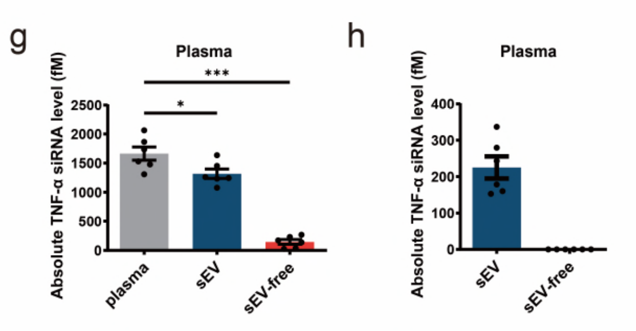

Figure 2. (g) Quantitative RT-PCR analysis of the absolute expression levels of TNF-α siRNA in plasma, sEVs and sEV-free plasma (n = 6 in each group). Mouse plasma was equally divided into two samples: one for direct isolation of total RNA and then quantification of siRNA levels, and the other for purification of sEVs using a commercially available kit and then quantification of siRNA levels in sEV fraction and sEV-free plasma fraction. Because sEV fraction and sEV-free plasma fraction are from the same plasma sample, the levels of siRNA in total plasma, sEVs and sEV-free plasma are comparable through a simple conversion process. **(h)** Quantitative RT-PCR analysis of the absolute expression levels of TNF-α siRNA in sEVs and sEV-free plasma (n = 6 in each group). sEVs were purified by using density gradient centrifugation. Values are presented as the mean ± SEM. Significance was determined using one-way ANOVA followed by Dunnett's multiple comparison. * $p < 0.05$; *** $p < 0.005$.

Meanwhile, we compared the plasma concentration of TNF-α siRNA to that of miR-16 and let-7i, which are two of the most abundant miRNAs in the blood circulation. TNF-α siRNA was present in mouse plasma in a similar concentration range as endogenous miR-16 and let-7i (Fig. 2f). Therefore, TNF-α siRNA is present in mouse plasma in a form that can be easily delivered to other cells (in sEVs) and at a concentration that is biologically relevant, similar to those endogenous secretory miRNAs.

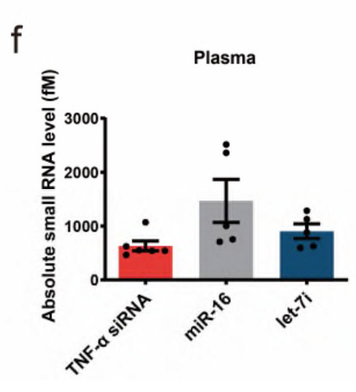

Figure 2. (f) Quantitative RT-PCR analysis of the absolute expression levels of TNF-α siRNA, miR-16 and let-7i in mouse plasma at 12 hours after injection (n = 5 in each group). Values are presented as the mean ± SEM.

According to reviewer's suggestion, we have also characterised the protective roles of the lipid bilayer structure of siRNA-encapsulating sEVs. Plasma sEVs derived from CMV-siR$^{TNF-\alpha}$-injected mice were divided into four equal portions and were untreated or treated with RNase, Triton X-

100 or RNase plus Triton X-100 for 30 min. The retained TNF-α siRNA in each sample was examined. TNF-α siRNA levels in the sEVs treated with RNase were little changed compared to untreated sEVs, and TNF-α siRNA levels in the sEVs treated with RNase and Triton X-100 were completely eliminated (Fig. 2i), which suggests that TNF-α siRNA was present within the sEVs and not simply released as a contaminant during the sEV purification process.

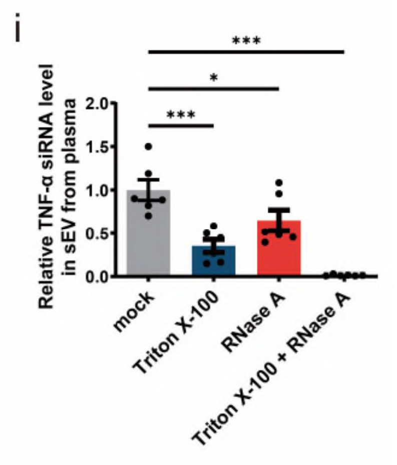

Figure 2. (i) Quantitative RT-PCR analysis of the survival percentage of TNF-α siRNA in sEVs treated with RNase A, Triton X-100 or RNase A plus Triton X-100 (n = 6 in each group). Untreated sEV sample served as a control. Values are presented as the mean ± SEM. Significance was determined using one-way ANOVA followed by Dunnett's multiple comparison. * $p < 0.05$; *** $p < 0.005$.

4. Figure 2f/h. Concentrations of siRNA in colon and spleen are cumulatively similar or even higher than in liver. This is difficult to envision using the model proposed by the authors, where (1) only a small fraction of siRNA will be packaged into exosomes/EVs and secreted, and (2) those siRNAs will be further diluted and distributed throughout the body. A more logical explanation would be that circuit DNA has been delivered into colon or spleen or to immune cells directly that have homed to sites of inflammation. Can the authors demonstrate that their plasmid DNA (hydrodynamic) injection does not lead to expression of siRNA outside liver, especially in this model of high inflammation?

Response: We understand reviewer's concern regarding this issue. Indeed, we should demonstrate that our normal tail vein injection procedure (rather than hydrodynamic injection) does not lead to uptake of genetic circuit (plasmid) and expression of siRNA outside the liver, especially in the model of high inflammation. In the revised manuscript, we have determined the amounts of the genetic circuits (plasmids) that were taken up by the liver. The CMV-siR$^{TNF-α}$ circuit (in the form of naked DNA plasmid) with known concentration was serially diluted and assessed using quantitative PCR to generate a standard curve. Then the CMV-siR$^{TNF-α}$ circuit was intravenously injected into DSS mice at the dose of 5 mg/kg. At 0, 1, 3, 6, 12 and 24 hours post-injection, mice were sacrificed, and liver, spleen and colon tissues were collected. Subsequently, the C_T values of CMV-siR$^{TNF-α}$ circuit was determined in the DNA samples extracted from 10 mg liver, spleen and colon tissues at each time point. By referring to the standard curve, the concentration of CMV-

siR$^{TNF-\alpha}$ circuit in the liver, spleen and colon was calculated. As can be seen in new Figure 2b, the CMV-siR$^{TNF-\alpha}$ circuit rapidly accumulated in the livers at 1 h then decreased to background levels at 6 h. No CMV-siR$^{TNF-\alpha}$ circuit was detected in other tissues of CMV-siR$^{TNF-\alpha}$-injected mice.

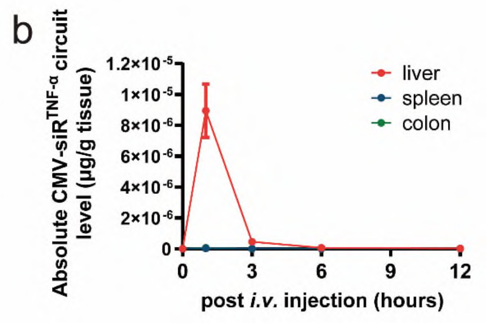

Figure 2. (b) Kinetics of CMV-siR$^{TNF-\alpha}$ circuit (DNA plasmid) in mouse liver, colon or spleen following tail vein injection of the 5 mg/kg CMV-siR$^{TNF-\alpha}$ circuit (n = 3 in each group). Values are presented as the mean ± SEM.

To further confirm that hepatocytes were the original site for the self-assembly and secretion of TNF-α siRNA-encapsulating sEVs, we isolated primary mouse hepatocytes at different time points following the injection of the CMV-siR$^{TNF-\alpha}$ circuit into mice and assessed TNF-α siRNA levels in sEVs derived from the culture medium of primary hepatocytes. TNF-α siRNA levels were higher in the sEVs of primary hepatocytes isolated from CMV-siR$^{TNF-\alpha}$ circuit-injected mice at 6 hours and were diminished in the sEVs of primary hepatocytes isolated from CMV-siR$^{TNF-\alpha}$ circuit-injected mice at 24 hours (Fig. 2j). These results indicate that the secretion of TNF-α siRNA-encapsulating sEVs increased after the injection of CMV-siR$^{TNF-\alpha}$ circuit, reached a peak at approximately 6 hours then declined to background at 24 hours in the *ex vivo* model. Overall, these results support the idea that the liver can assemble and secrete siRNA-encapsulating sEVs introduced by intravenously injected genetic circuits, although the genetic circuits (plasmids) undergo rapid DNA degradation or promoter silencing *in vivo*.

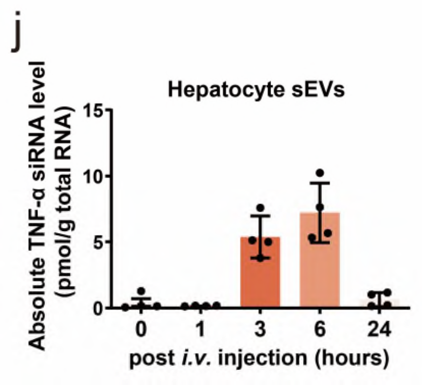

Figure 2. (j) Primary mouse hepatocytes were isolated from mice at different time points following intravenous injection of 5 mg/kg CMV-siR$^{TNF-\alpha}$ circuit. A quantitative RT-PCR assay was performed to assess TNF-α siRNA levels in sEVs derived from the culture medium of primary hepatocytes (n = 4 in each group). Values are presented as the mean ± SEM.

On the other hand, we totally agree with the reviewer that the concentrations of TNF-α siRNA in colon and spleen need to be measured more accurately. Indeed, because only a small fraction of TNF-α siRNA in the liver will be packaged into sEVs and secreted into blood, and because these secretory siRNAs will be further diluted and distributed throughout the body, it is particularly important to ensure that the TNF-α siRNA delivered to the sites of intestinal inflammation has reached a working concentration to inhibit its target. In the revised manuscript, we have shown that (1) TNF-α siRNA was largely encapsulated in sEVs rather than sEV-free plasma; (2) TNF-α siRNA was indeed present within the bilayer-enclosed sEVs and is protected from blood-borne RNases by the intact structure of sEVs; and (3) TNF-α siRNA was present in the mouse plasma in a similar concentration range to endogenous miR-16 and let-7i. Thus, TNF-α siRNA is present in mouse plasma in a form that can be easily delivered to other cells (in sEVs) and at a concentration that is biologically relevant, similar to those endogenous secretory miRNAs.

5. Terminology: Exosomes are a multivesicular body-derived subtype of extracellular vesicles. Without demonstrating the intracellular origin of vesicles studied, the general term ‘extracellular vesicle’ seems more appropriate.

<u>Response</u>: We apologize for the confusion. The terminology describing extracellular vesicles has been changed substantially. We totally agree with the reviewer that the general term “extracellular vesicle” is more appropriate without demonstrating the intracellular origin of the vesicles studied. Since we actually focused on small extracellular vesicles (< 200 nm), we have changed the term “exosomes” to “small extracellular vesicles (sEVs)” throughout the manuscript.

6. Abstract: The final sentence (after requiring) is ended abruptly.

<u>Response</u>: We apologize for this error. We have re-written this sentence as follows: “Overall, this study established a feasible combination therapeutic strategy for UC, which is superior to the conventional biological therapies requiring two or more independent compounds or antibodies.”

7. Suppl Table 2. The differences between the sequences of siRNA expression cassettes listed in this table, as compared to the CMV-circuit vector as described in the methods, are unclear and should be clearly explained.

<u>Response</u>: We apologize for not indicating this issue clearly. To visualize the information and structure of the sequences, we have changed Supplementary Table 2 to Supplementary Figure 25 in the revised manuscript. In Supplementary Figure 25, we provided the sequences of siRNA expression cassettes, which are composed of siRNA guide strand, pre-miR-155 backbone and siRNA passenger strand. As can be seen in this new figure, how guide and passenger strands and pre-miR-155 backbone are organized as a siRNA expression cassette is present more clearly.

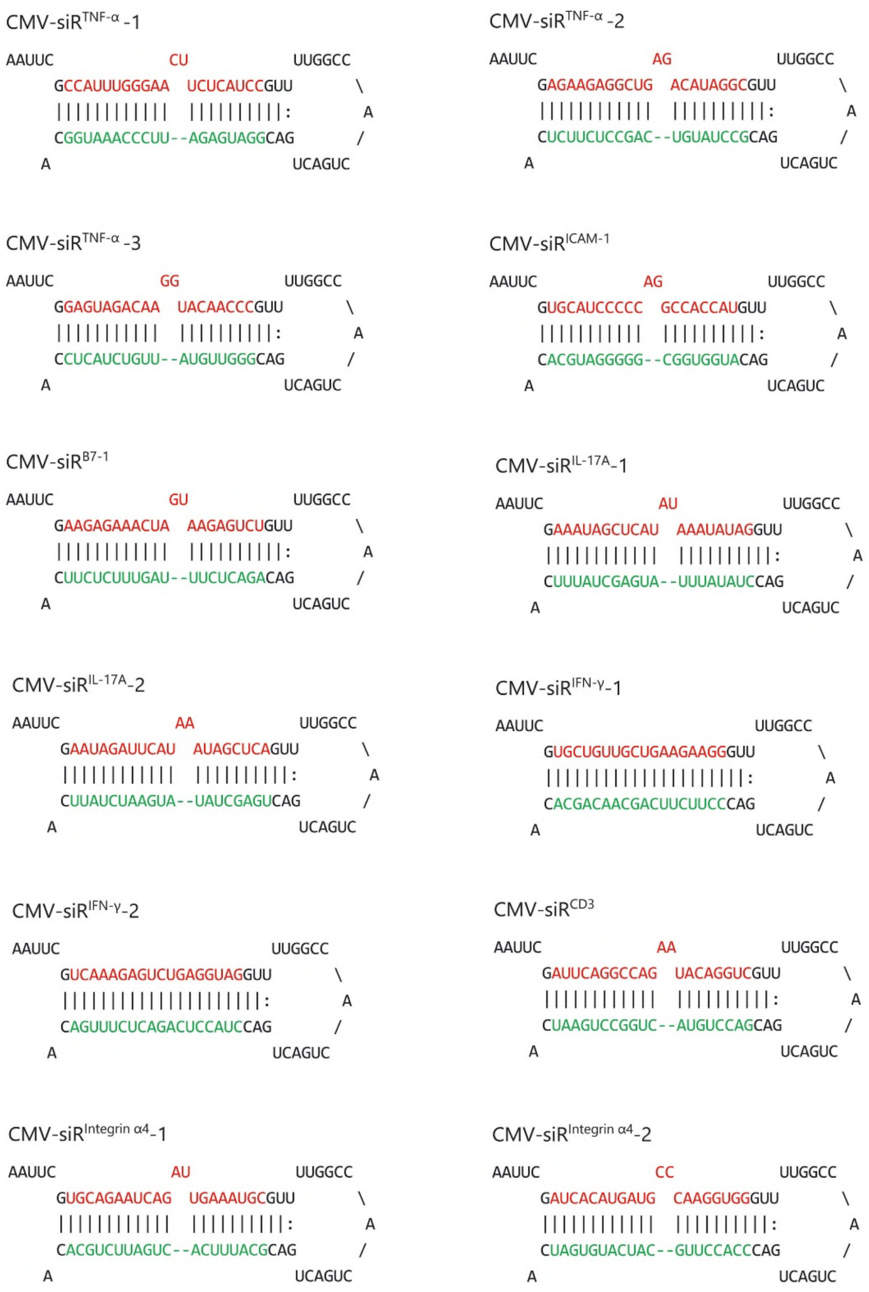

Supplementary Figure 25. Sequences of siRNA expression cassettes. Sequences of the siRNA expression cassettes designed for silencing of TNF-α, IL-17A, IFN-γ, IL-6, integrin α4, ICAM-1, CD3 and B7-1 are shown. Guide strands are marked in red, and passenger strands are marked in green.

8. Suppl Table 2. Sense and antisense sequences are identical for CMV-siR^{TNFa}-1.

Response: We apologize for this error and have corrected it in the revised manuscript. Moreover, we have double-checked the revised manuscript to exclude such errors.

9. Suppl figure 1a. In the figure, it seems LPS is also present in the condition 'normal', which I guess is not the case.

<u>Response</u>: We greatly thank the reviewer for pointing out this issue. We have modified Supplementary Figure 1a and 1b to avoid the confusion.

10. Figure 2D. What was the final concentration of siRNA added here?

<u>Response</u>: We apologize for missing this experimental detail. In new Figure 2k and 2l (original Figure 2D), acute UC was induced in male BALB/c mice by replacing their drinking water with a 2.5% DSS solution for 7 days. DSS mice were intravenously injected with 5 mg/kg CMV-scrR or CMV-siR$^{TNF\text{-}\alpha}$ circuit every day for a total of 7 times, and then the sEVs were purified from the plasma of each mouse and dissolved in 50 µL PBS. BCA method was employed to quantify total protein content in sEVs, and the isolated sEVs had a total protein concentration of ~0.8 µg/µL. Subsequently, the sEV solution (50 µL) was incubated with 1×10^5 primary macrophages. After stimulating macrophages with 50 ng/mL LPS, quantitative RT-PCR analysis was performed to determine the relative expression levels of TNF-α mRNA in primary macrophages (Fig. 2k), and ELISA was performed to determine the levels of secretory TNF-α protein in cell culture supernatant (Fig. 2l). We have added this information to the legend of Figure 2k and 2l of the revised manuscript.

11. Line 377, "a synergistic advantage". Wouldn't this effect simply be additive?

<u>Response</u>: We thank the reviewer for pointing out this issue. We have corrected this sentence according to reviewer's suggestion.

Reviewer #2

1. Organ-targeting self-assembled siRNA therapy has been conducted in different disease models, and the therapeutic potential of the RNAi is also used in an experimental colitis model of IBD by targeting colon macrophages to reduce inflammatory symptoms (Nat Nanotechnol 2018 03;13(3)). It appears that this concept is not novel enough.

<u>Response</u>: We greatly thank the reviewer for pointing out this critical issue. Indeed, recent study has developed a self-assembled modular platform based on a membrane-anchored lipoprotein that is incorporated into siRNA-loaded lipid nanoparticles that interact with the antibody crystallizable fragment (Fc) domain.[10] The therapeutic potential of the platform has been demonstrated in an IBD model by targeting colon macrophages to reduce inflammatory symptoms. This study demonstrates the superiority of the self-assembled modular platform to deliver siRNAs to targeted cell populations *in vivo* and establishes a promising tool that can be simply translated into clinic, because the self-assembly strategy is quite simple and avoids the the massive development and production requirements and the high batch-to-batch variability of current technologies. Likewise,

we employed a similar concept of self-assembly based on the nature of sEVs, since sEVs and their cargoes are self-assembled by endogenous cells.

We have added a whole paragraph to the Discussion section of the revised manuscript to emphasize this critical issue, which reads as follows: "Despite recent advancements in our knowledge of the pathogenic mechanisms of UC, a considerable unmet medical need for the treatment of UC remains. Overactive immune responses are the most promising therapeutic targets, and biological therapy using monoclonal antibodies (e.g., infliximab) has been specifically developed to block immunological targets and alleviate immune responses.[11,12] However, the high treatment costs, serious side effects and development of drug resistance associated with the development of autoimmunity to antibodies remain serious problems for biological therapy. Therefore, it is urgent to develop an alternative strategy with high therapeutic efficiency and few side effects. Because siRNA is solely dependent on the mRNA sequence and inhibits immunological targets with strong specificity, RNAi therapy has the intrinsic ability to overcome the shortcomings of biological therapy.[13,14] Unfortunately, the lack of safe and effective carriers for the delivery of siRNA therapeutics remains a major barrier to its broad clinical application, especially for extrahepatic delivery situations. An ideal siRNA carrier must overcome a series of biological hurdles throughout the course of delivery: it should have no stimulatory effects on immune systems, protect siRNA from degradation by RNases in the internal environment, have proper permeability of the cell membrane, possess a suitable binding strength with siRNA and enable escape from lysosomes before the release of siRNAs into the cytoplasm of target cells. Kedmi et al. recently developed a self-assembled modular platform based on a membrane-anchored lipoprotein that was incorporated into siRNA-loaded lipid nanoparticles that interacted with the antibody crystallisable fragment (Fc) domain.[10] The therapeutic potential of the platform was demonstrated in an IBD model by targeting colon macrophages to reduce inflammatory symptoms. This study demonstrated the superiority of the self-assembled modular platform for the delivery of siRNAs to targeted cell populations *in vivo*. Notably, it established a promising tool that may be simply translated into the clinic because the self-assembly strategy is quite simple and avoids the massive development and production requirements and the high batch-to-batch variability of current technologies. We used a similar concept of self-assembly based on the nature of sEVs because sEVs and their cargoes are self-assembled by endogenous cells. Based on the intrinsic ability of the small RNA processing machinery of the host liver to self-assemble siRNA into sEVs and the endogenous circulating system of sEVs to transport siRNAs, we designed genetic circuits to engineer mouse liver to self-assemble siRNAs into secretory sEVs and deliver siRNA-encapsulating sEVs via the blood circulation to the sites of intestinal inflammation, which resulted in significant target gene reduction and symptom alleviation in acute and chronic UC models. Notably, genetic circuits showed an overall advantage over infliximab in alleviating the symptoms and signs of UC in different UC models (Supplementary Table 1). Because sEVs harbour excellent cellular compatibility and less immunogenicity than other delivery vehicles, no cytotoxicity, immunogenicity or side effects were observed after treatment with genetic circuits."

2. Authors focused on TNF-α as the therapeutic target to alleviate experimental colitis in a DSS-induced acute colitis mice. What about another proinflammatory cytokines, e.g., IL-6, IL-17A, IL-12, IL-23 ?

Response: We apologise for not indicating this issue clearly. In fact, to develop a multitargeted genetic circuit to simultaneously block multiple causal genes relevant to the onset and persistence of UC, we have evaluated the therapeutic efficacy of the genetic circuits targeting IL-17A, IFN-γ and IL-6 in a DSS-induced acute UC model. However, genetic circuits targeting IL-17A, IFN-γ and IL-6 were shown to be less effective than the circuit targeting TNF-α in ameliorating the manifestations of DSS-induced UC, as assessed by the DAI score and colon length (Supplementary Fig. 9a-9c). Meanwhile, genetic circuits targeting IL-17A, IFN-γ and IL-6 were defective in suppressing their corresponding target genes in acute UC model (Supplementary Fig. 9d-9j). Therefore, these proinflammatory cytokines were not included as the target genes in the multitargeted genetic circuit carrying triplicate siRNA expression cassettes. The causes of low therapeutic effects may lie in the fact that the siRNA sequences targeting IL-17A, IFN-γ and IL-6 have not been optimized to achieve the highest silencing efficacy, or the amount of *in vivo* self-assembled siRNAs have not reached a sufficient level to inhibit the abundant proinflammatory cytokines in inflamed colon. On the other hand, although numerous pro-inflammatory cytokines are actively involved in the pathogenesis of UC, there is still a huge gap to target these pro-inflammatory cytokines for the treatment of UC, perhaps because the precise aetiology and pathogenesis of UC remain unclear. While the primary goal of this study is to develop a feasible combination therapeutic strategy for UC, our findings have provided proof-of-concept for combination therapy of UC using *in vivo* self-assembled siRNAs. For the screening of more ideal target combinations, we plan to design a systematic and comprehensive project and conduct the relevant experiments in the future.

3. Did the CMV-siR$^{TNF\text{-}\alpha}$ treatment show apparently advantage compared to anti-TNF-α mAb treatment (e.g., IFX or adalimumab)? It is necessary to clarify the differences between two kinds of therapy in body weight, colon length, DAI, and expression of proinflammatory genes or proteins in inflamed colon.

Response: We appreciate reviewer’s emphasis on this critical issue. We totally agree with the reviewer that we should clarify the differences between treatment with genetic circuits and anti-TNF-α mAb more clearly. We have provided a table (Supplementary Table 1) to compare the therapeutic effects between genetic circuits and infliximab in term of body weight, colon length, DAI score, histological score and expression of proinflammatory proteins in inflamed colon. As can be seen in this table, treatment with genetic circuits shows apparently advantage compared to treatment with infliximab in different UC models.

Supplementary Table 1. Comparison of the therapeutic effects between genetic circuits and infliximab (IFX).

Models	Groups	Body weight	Colon length	DAI	Expression of pro-inflammatory cytokines	Pathological evaluation
DSS-induced acute UC model	CMV-siR$^{TNF-\alpha}$ *vs.* IFX	≈	>	>	>	<
TNBS-induced acute colitis model	CMV-siR$^{TNF-\alpha}$ *vs.* IFX	>	>	>	\	\
DSS-induced chronic UC model	CMV-siR$^{TNF-\alpha}$ *vs.* IFX	>	>	>	>	>
TNBS-induced chronic colitis model	CMV-siR$^{TNF-\alpha}$ *vs.* IFX	>	≈	>	>	>
IL-10$^{-/-}$ mice with spontaneous chronic colitis	CMV-siR$^{TNF-\alpha}$ *vs.* IFX	≈	>	\	>	<
IL-10$^{-/-}$ mice with spontaneous chronic colitis	CMV-siR^{T+B+I} *vs.* IFX	≈	>	\	>	>
DSS-induced chronic UC model	AAV8-TBG-siR^{T+B+I} *vs.* IFX	≈	>	>	≈	≈

>: Plasmid-based or AAV-driven genetic circuits perform better than IFX.
<: Plasmid-based or AAV-driven genetic circuits perform worse than IFX.
≈: Plasmid-based or AAV-driven genetic circuits perform same as IFX.
\: not available.

4. After CMV-siR$^{TNF-\alpha}$ treatment, what about the functional alterations of macrophages besides reducing the production of TNF-α? Or, in detail, whether CMV-siR$^{TNF-\alpha}$ treatment could affect the differentiation and polarization of the macrophages in the inflamed colon?

Response: We greatly thank the reviewer for this constructive suggestion. Macrophages are highly plastic cells that polarise into different phenotypes (pro-inflammatory M1 macrophages *vs.* immunosuppressive M2 macrophages) in response to surrounding stimuli.[15] Macrophages in the colon of UC are recruited and polarised locally into the M1 phenotype to drive further inflammation.[16] According to reviewer's suggestion, we investigated whether CMV-siR$^{TNF-\alpha}$ treatment could affect the differentiation and polarization of the macrophages in the inflamed colon (new Fig. 4h-4k). CMV-siR$^{TNF-\alpha}$ circuit-treated mice exhibited increased expression of CD206 (a marker of M2 macrophages) and reduced expression of NOS2 (a marker of M1 macrophages) in the colon compared to the control mice treated with the CMV-scrR circuit (Fig. 4h and 4i). The increased level of CD206 and the depressed level of NOS2 in the chronic UC model indicated a shift in intestinal macrophage balance towards the anti-inflammatory M2 phenotype. Macrophages were specifically isolated and characterised using flow cytometry to determine the expression of Ly6C and CD206, which differentiate between pro-inflammatory and immunosuppressive subsets of macrophages. A significantly higher rate of macrophages derived from the CMV-siR$^{TNF-\alpha}$ circuit-treated mice were identified as Ly6C^{low}CD206^{high} macrophages compared to CMV-scrR circuit-treated control mice (Fig. 4j and 4k), which is consistent with an anti-inflammatory M2 phenotype. Infliximab also increased M2 macrophages and decreased M1 macrophages in the inflamed colon of the chronic UC model, and the therapeutic efficacy was equivalent to the CMV-siR$^{TNF-\alpha}$ circuit (Fig. 4h-4k). Overall, these results demonstrate that *in vivo*

self-assembled TNF-α siRNA actively regulates the differentiation and polarisation of macrophages in the inflamed colon and modulates the balance between M1 and M2 macrophages to prevent intestinal inflammation.

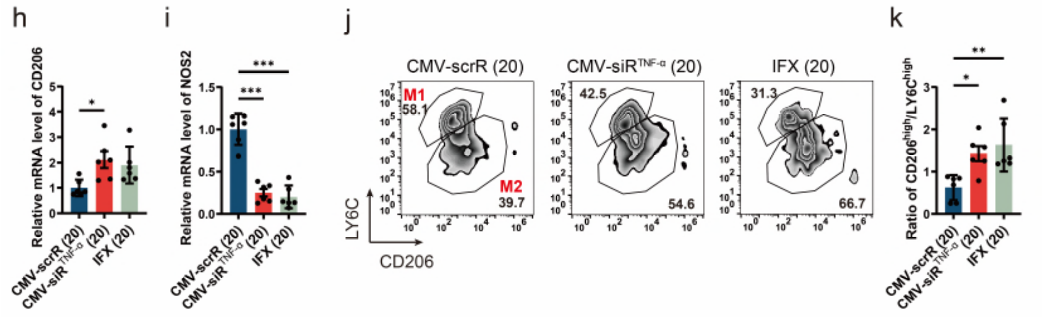

Figure 4. (h and i) Quantitative RT-PCR analysis of the relative expression levels of CD206 and NOS2 mRNA in the colon (n = 6 in each group). **(j)** Representative flow cytometric characterisation of colonic macrophages ($CD11b^+CD64^+$) subdivided into M1-like ($Ly6C^{high}CD206^{low}$) and M2-like ($Ly6C^{low}CD206^{high}$) macrophages. **(k)** Bar graph depicting the ratio between M2-like versus M1-like macrophages (n = 5 in each group). Values are presented as the mean ± SEM. Significance was determined using one-way ANOVA followed by Dunnett's multiple comparison. * $p < 0.05$; ** $p < 0.01$; *** $p < 0.005$.

5. TNF-α could be produced from other immune cells, e.g., $CD4^+$ T cells. Previous studies have demonstrated that IFX treatment could modulate functional changes of $CD4^+$ T cells, e.g., enhancing the secretion of IL-22. Therefore, CMV-siR$^{TNF\text{-}\alpha}$ treatment might also affect the functional changes or differentiation of $CD4^+$ T cells in inflamed mucosa? Authors need to have a analysis on phenotypic changes of mucosal $CD4^+$ T cells.

Response: We thank the reviewer for this excellent suggestion. According to reviewer's suggestion, we investigated whether CMV-siR$^{TNF\text{-}\alpha}$ treatment affected functional changes of $CD4^+$ T cells in inflamed mucosa (new Figure 4l-4o and Supplementary Fig. 4g-4k). IBD is classically associated with gut accumulation of pro-inflammatory T-helper 1 (Th1) and T-helper 17 (Th17) cells and an insufficient presence of regulatory T cells (Tregs) to suppress inflammation.[17,18] Analysis of the canonical Th1 cytokine IFN-γ and the Th17 cytokines IL-17A and IL-22 using ELISA revealed that treatment with the CMV-siR$^{TNF\text{-}\alpha}$ circuit significantly reduced the levels of IFN-γ, IL-17A and IL-22 in the colon of DSS mice. In contrast, the levels of IL-10 and transforming growth factor-β1 (TGF-β1), two main Treg cytokines that suppress the inflammatory response, were increased by treatment with the CMV-siR$^{TNF\text{-}\alpha}$ circuit (Supplementary Fig. 4g-4k). We also analysed the phenotypes of $CD4^+$ T cells using flow cytometry and found that CMV-siR$^{TNF\text{-}\alpha}$ circuit treatment resulted in a significantly reduced percentage of IFN-γ^+CD4^+ T cells and IL-$17A^+CD4^+$ T cells in the colonic lamina propria of DSS mice (Fig. 4l-4o). These results indicate that *in vivo* self-assembled TNF-α siRNA restores the balance of Th1/Th17/Treg subsets and protects against intestinal inflammation in the UC model.

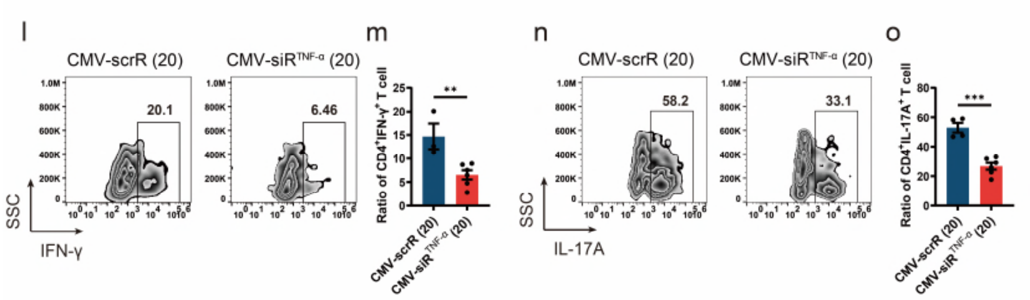

Figure 4. (l and n) Lamina propria mononuclear cells **(**LPMCs) were stimulated with PMA/Ionomycin mixture and BFA/Monensin mixture for 5 hours, and the percentage of $CD4^{+}IFN\text{-}\gamma^{+}$ cells and $CD4^{+}IL\text{-}17A^{+}$ cells in $CD4^{+}$ T cells was measured by flow cytometry. **(m and o)** Bar graph depicting the percentage of $CD4^{+}IFN\text{-}\gamma^{+}$ T cells and $CD4^{+}IL\text{-}17A^{+}$ T cells (n = 3-4 in each group). Values are presented as the mean ± SEM. Significance was determined using one-way ANOVA followed by Dunnett's multiple comparison. ** p < 0.01; *** p < 0.005.

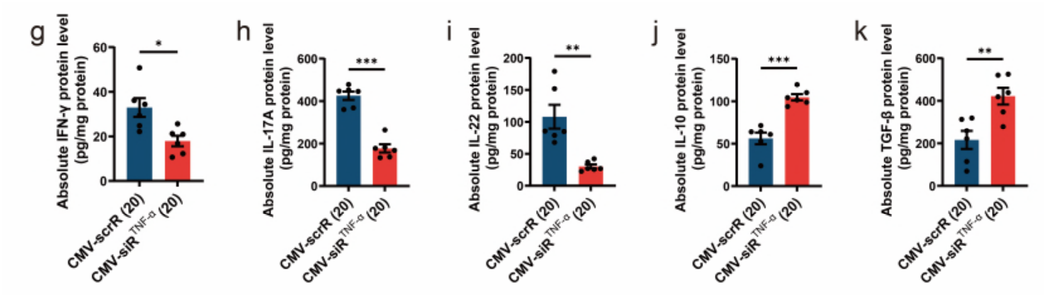

Supplementary Figure 4. (g–k) Determination of the levels of IFN-γ, IL-17A, IL-22, IL-10 and TGF-β1 in the colon by ELISA (n = 6 in each group). Values are presented as the mean ± SEM. Significance was determined using one-way ANOVA followed by Dunnett's multiple comparison. * p < 0.05; ** p < 0.01; *** p < 0.005.

6. DSS-induced acute colitis model in mice is not an immune cell-mediated model. Authors need to replicate the CMV-siR$^{TNF\text{-}\alpha}$ treatment in spontaneous colitis model or CD4+ T cell-induced chronic colitis model, such as in Rag-/- mice reconstituted with CD45RBhigh CD4+ T cells.

__**Response**__: We greatly thank the reviewer for this constructive suggestion. Interleukin-10 (IL-10) is an anti-inflammatory cytokine that plays a major role in gut homeostasis.[19] IL-10 knockout (IL-10$^{-/-}$) mice develop spontaneous chronic enterocolitis that closely resembles the phenotypes of human IBD. According to reviewer's suggestion, we have administered a single-targeted CMV-siR$^{TNF\text{-}\alpha}$ circuit and a multi-targeted CMV-siR^{T+B+I} circuit to IL-10$^{-/-}$ mice and evaluated the therapeutic effects of self-assembled siRNAs in this spontaneous colitis model (new Figure 7). Compared to wild-type normal mice, CMV-scrR circuit-treated IL-10$^{-/-}$ mice developed severe gut inflammation at approximately 8 weeks of age, which was characterised by substantial weight loss and a shorter colon length (Fig. 7a-7c). In contrast, treatment with the CMV-siR$^{TNF\text{-}\alpha}$ and CMV-siR^{T+B+I} circuits significantly attenuated the loss of body weight and shortening of the colon in IL-10$^{-/-}$ mice (Fig. 7a-7c). Notably, the pro-inflammatory cytokines of the Th1 and Th17 classes, including IFN-γ, IL-17A, IL-6, IL-12/23p40, IL-23 and IL-12p70, were abundantly

present in the colon of CMV-scrR circuit-treated IL-$10^{-/-}$ mice, but these pro-inflammatory cytokines were significantly diminished in the colon of IL-$10^{-/-}$ mice following treatment with CMV-$siR^{TNF\text{-}\alpha}$ and CMV-siR^{T+B+I} circuits (Fig. 7d and 7e). Histological examination detected obvious pathological signs of colitis in CMV-scrR circuit-treated IL-$10^{-/-}$ mice, including epithelial hyperplasia, crypt abscesses, ulceration, bowel wall thickening and massive leukocytic infiltration, but the IL-$10^{-/-}$ mice treated with CMV-$siR^{TNF\text{-}\alpha}$ and CMV-siR^{T+B+I} circuits exhibited much better consistency and morphology of colons without infiltrating inflammatory cells, and the histological scores of these mice were significantly lower than the CMV-scrR circuit-treated mice (Fig. 7f and 7g). Although infliximab protected IL-$10^{-/-}$ mice from loss of body weight, shortening of colon lengths and production of excessive pro-inflammatory cytokines and mitigated the histological signs of colitis in colon sections, its therapeutic effect was inferior to the CMV-siR^{T+B+I} circuit (Fig. 7a-7g). At the molecular level, treatment with the CMV-$siR^{TNF\text{-}\alpha}$ circuit, CMV-siR^{T+B+I} circuit and infliximab substantially reduced TNF-α levels in the colon tissues (Fig. 7h and 7i), but only the CMV-siR^{T+B+I} circuit knocked down B7-1 and integrin α4 levels in the colons of IL-$10^{-/-}$ mice (Fig. 7j and 7k). Taken together, these data demonstrate that the *in vivo* self-assembled siRNAs limit excessive inflammation and promote intestinal recovery in a spontaneous chronic colitis model.

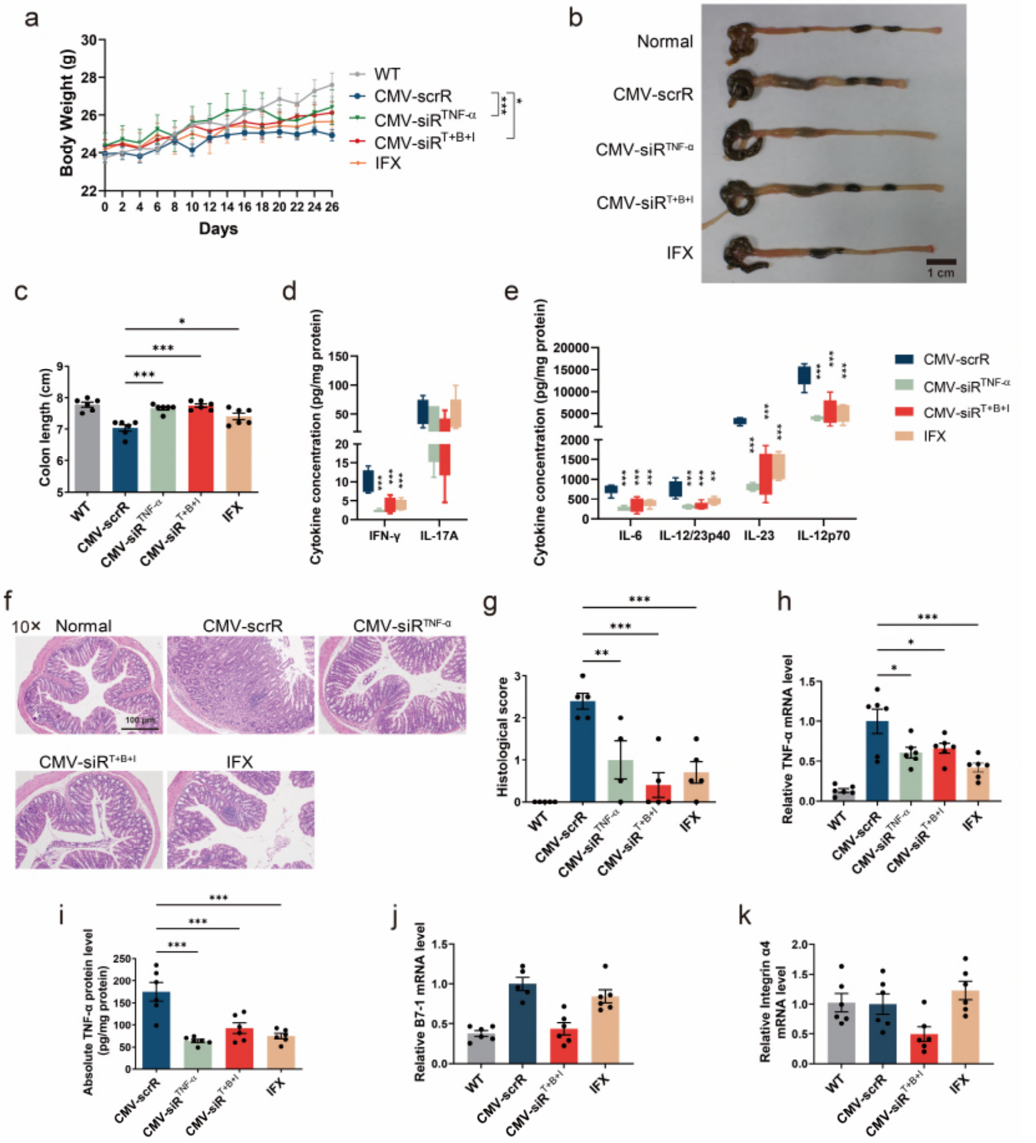

Figure 7. Intravenous injection of the CMV-siR$^{TNF\text{-}\alpha}$ or CMV-siR^{T+B+I} circuit ameliorates spontaneous UC in IL-10$^{-/-}$ mice. Eleven-week-old IL-10$^{-/-}$ mice were intravenously injected with equal dose (10 mg/kg) of CMV-scrR, CMV-siR$^{TNF\text{-}\alpha}$ or CMV-siR^{T+B+I} circuit or with 10 mg/kg infliximab (IFX) every two days (n = 6 in each group). After 14 injections, the mice were euthanised. Body weights were monitored every two days, and symptoms and histology were evaluated on day 28. Untreated C57BL/6 mice were included as normal controls. **(a)** Body weight curves (n = 6 in each group). **(b)** Representative macroscopic features of colons. Scale bar: 1 cm. **(c)** Mean colon length (n = 6 in each group). **(d and e)** Determination of the levels of IFN-γ, IL-17A, IL-6, IL-12/23p40, IL-23 and IL-12p70 in the colon by ELISA (n = 6 in each group). **(f)** Representative images of H&E staining of colon sections. Scale bar: 200 μm. **(g)** Histological scores of colon sections (n = 4-5 in each group). **(h)** Quantitative RT-PCR analysis of the relative expression levels of TNF-α mRNA in the colon (n = 6 in each group). **(i)** Determination of the absolute expression levels of TNF-α protein in the colon by ELISA (n = 6 in each group). **(j and**

k) Quantitative RT-PCR analysis of the relative expression levels of B7-1 and integrin α4 mRNA in the colon (n = 6 in each group). Values are presented as the mean ± SEM. Significance was determined using one-way ANOVA followed by Dunnett's multiple comparison in panel c, d, e, g, h, i, j and k or using two-way ANOVA followed by Dunnett's multiple comparison in panel a. * $p < 0.05$; ** $p < 0.01$; *** $p < 0.005$.

Reviewer #3

1. The methods used in the manuscript are appropriate and the results achieved by using the DSS colitis model were confirmed by adding the TNBS-model as a second colitis model. The conclusions are justified and supported by the data provided.

Response: We appreciate the reviewer for these positive comments.

2. When using the AAV genomic circuit, the authors used a prophylactic treatment approach as they treated healthy mice with the circuit and 3 weeks later induced UC. While this is an interesting first step, it would be important to evaluate the therapeutic value of the AAV genomic circuit by inducing UC first and then treating the mice with the genomic circuit. The authors successfully tested a therapeutic approach for the use of the CMV siRNA circuit. This should also be done with the AAV-circuit.

Response: We appreciate reviewer's constructive suggestion on this issue. We totally agree with the reviewer that we should evaluate the therapeutic value of the AAV-driven genetic circuit by inducing UC first and then treating the mice with the genetic circuit. Starting at week 0, we established a chronic UC model by allowing BALB/c mice to rhythmically drink DSS for 1 week and water for 2 weeks, and the cycle was repeated 3 times. At the same time at week 0, we intravenously injected chronic UC mice with a control AAV encoding a scrambled RNA or with three dosages of AAV-driven genetic circuit. As a control, we intravenously injected an additional group of chronic UC mice with infliximab every 2 days throughout the experimental period. Finally, we evaluated the therapeutic effects at week 10.

On the other hand, because AAV9 has tropism for a wide range of tissues and CMV promoter is a strong constitutive promoter without tissue specificity, the therapeutic effects observed in UC models might not be liver-dependent. Since AAV8 has strong liver tropism and thyroxine-binding globulin (TBG) promoter is a hepatocyte-specific promoter,[20-23] we inserted the expression cassette designed for tandem expression of TNF-α siRNA, B7-1 siRNA and integrin α4 siRNA downstream of the TBG promoter and further incorporated the whole circuit into an AAV8 vector (hereafter, AAV8-TBG-siR^{T+B+I}) to ensure that the corresponding siRNAs are transcribed only in hepatocytes while avoiding undesired siRNA expression in extrahepatic cells.

We evaluated the therapeutic effects of AAV8-TBG-siR^{T+B+I} in a chronic UC model (Fig. 8a). AAV8-TBG-mediated luciferase expression was dose-dependently increased from weeks 2 to 7 and remained stable over 7 weeks. The luciferase signal was primarily restricted to the liver with

negligible signals in extrahepatic tissues (Fig. 8b and Supplementary Fig. 19a and 19b). Control mice receiving AAV8-TBG-scrR showed typical characteristics of colitis in the chronic UC model, such as sustained body weight loss, increased DAI score, shortened colon length, enhanced inflammatory cytokine levels and apparent histological features in colonic sections. However, the mice receiving a high dose of AAV8-TBG-siR^{T+B+I} rapidly recovered the lost body weight and colon length, experienced a low DAI score, showed a significant decline in inflammatory cytokines and exhibited an improved colonic histological appearance (Fig. 8c-8f and Supplementary Fig. 19c-19f). At the molecular level, treatment with AAV8-TBG-siR^{T+B+I} resulted in a dose-dependent production of TNF-α siRNA, B7-1 siRNA and integrin α4 siRNA in the liver (Supplementary Fig. 20a-20c), which were accompanied by a dose-dependent accumulation of TNF-α siRNA, B7-1 siRNA and integrin α4 siRNA in the colon (Fig. 8g-8i). Consequently, dose-dependent reductions in colonic TNF-α, B7-1 and integrin α4 mRNA levels were detected after AAV8-TBG-siR^{T+B+I} treatment (Supplementary Fig. 20d-20f). ELISA confirmed a dose-dependent decline in TNF-α protein levels in the colonic lamina propria derived from AAV8-TBG-siR^{T+B+I}-treated mice (Fig. 8j), and flow cytometry confirmed a dose-dependent loss of B7-1 and integrin α4 proteins on the membrane surface of mononuclear cells derived from the colonic lamina propria, peripheral blood and spleen of AAV8-TBG-siR^{T+B+I}-treated mice (Fig. 8k and 8l and Supplementary Fig. 21 and 22). Infliximab also exhibited significant therapeutic activity in the chronic UC model, but AAV8-TBG-siR^{T+B+I} ameliorated the manifestations of chronic colitis and restored the expression of TNF-α, B7-1 and integrin α4 to a better extent than infliximab (Fig. 8c-8l and Supplementary Fig. 19c-19f, 20, 21 and 22). Taken together, these results suggest that AAV8-TBG-mediated liver expression and assembly of siRNAs may serve as a potential solution to induce long-term combination therapy for chronic UC.

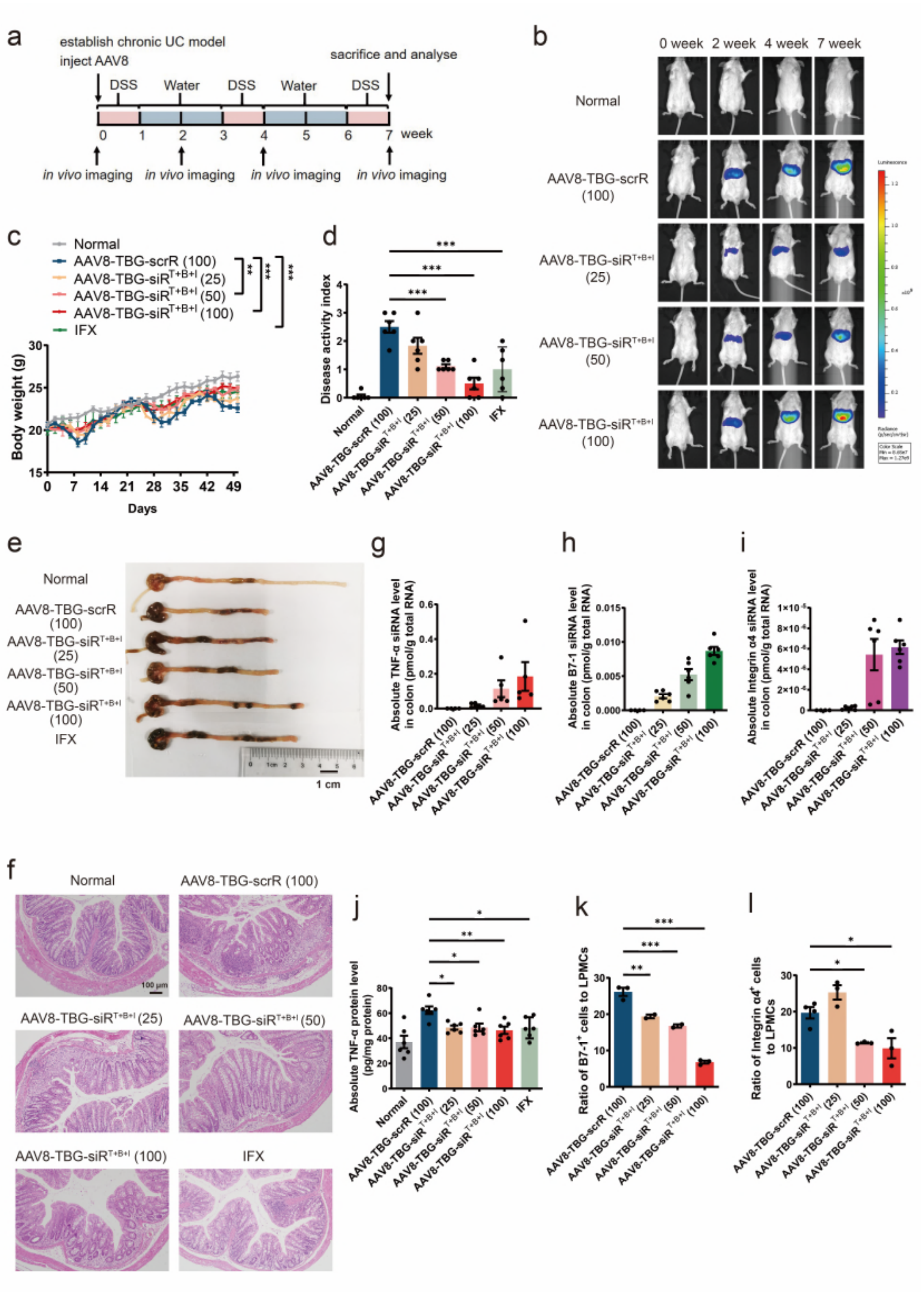

Figure 8. Intravenous injection of the AAV8-TBG-siR^{T+B+I} induces long-term combination therapy in the DSS-induced chronic UC model. (a) Flow chart of the experimental design. On week 0, chronic UC was induced by rhythmically administering to BALB/c mice 2.5% DSS for 1 week and water for 2 weeks, and the cycle was repeated for 3 times. At the same time, mice were intravenously injected with 100 μL AAV8-TBG-scrR (3 × 10^{12} V. G/mL) or 25, 50 or 100 μL AAV8-TBG-siR^{T+B+I} (3 × 10^{12} V. G/mL). Four days after each DSS drinking, mice were

intravenously injected with 10 mg/kg infliximab for a total of 3 times, once every 2 days. Untreated BALB/c mice were included as normal controls. Body weights were monitored every two days, and the symptoms and histology were evaluated at week 7. **(b)** Evaluation of AAV-mediated luciferase expression to reflect coexpressed siRNA accumulation *in vivo* (n = 6 in each group). **(c)** Body weight curves (n = 6 in each group). **(d)** DAI scores (n = 6 in each group). **(e)** Representative macroscopic features of colons. Scale bar: 1 cm. **(f)** Representative images of H&E staining of colon sections. Scale bar: 100 μm. **(g-i)** Quantitative RT-PCR analysis of the absolute expression levels of TNF-α siRNA, B7-1 siRNA and integrin α4 siRNA in the colon (n = 5-6 in each group). **(j)** Determination of the absolute expression levels of TNF-α protein in the colon by ELISA (n = 6 in each group). **(k)** The population of B7-1^+ cells in total colonic lamina propria mononuclear cells (n = 2-3 in each group). **(l)** The population of integrin α4^+ lymphocytes in total colonic lamina propria lymphocytes (n = 3-4 in each group). Values are presented as the mean ± SEM. Significance was determined using one-way ANOVA followed by Dunnett's multiple comparison in panel d, j, k and l or using two-way ANOVA followed by Dunnett's multiple comparison in panel c. * $p < 0.05$; ** $p < 0.01$; *** $p < 0.005$.

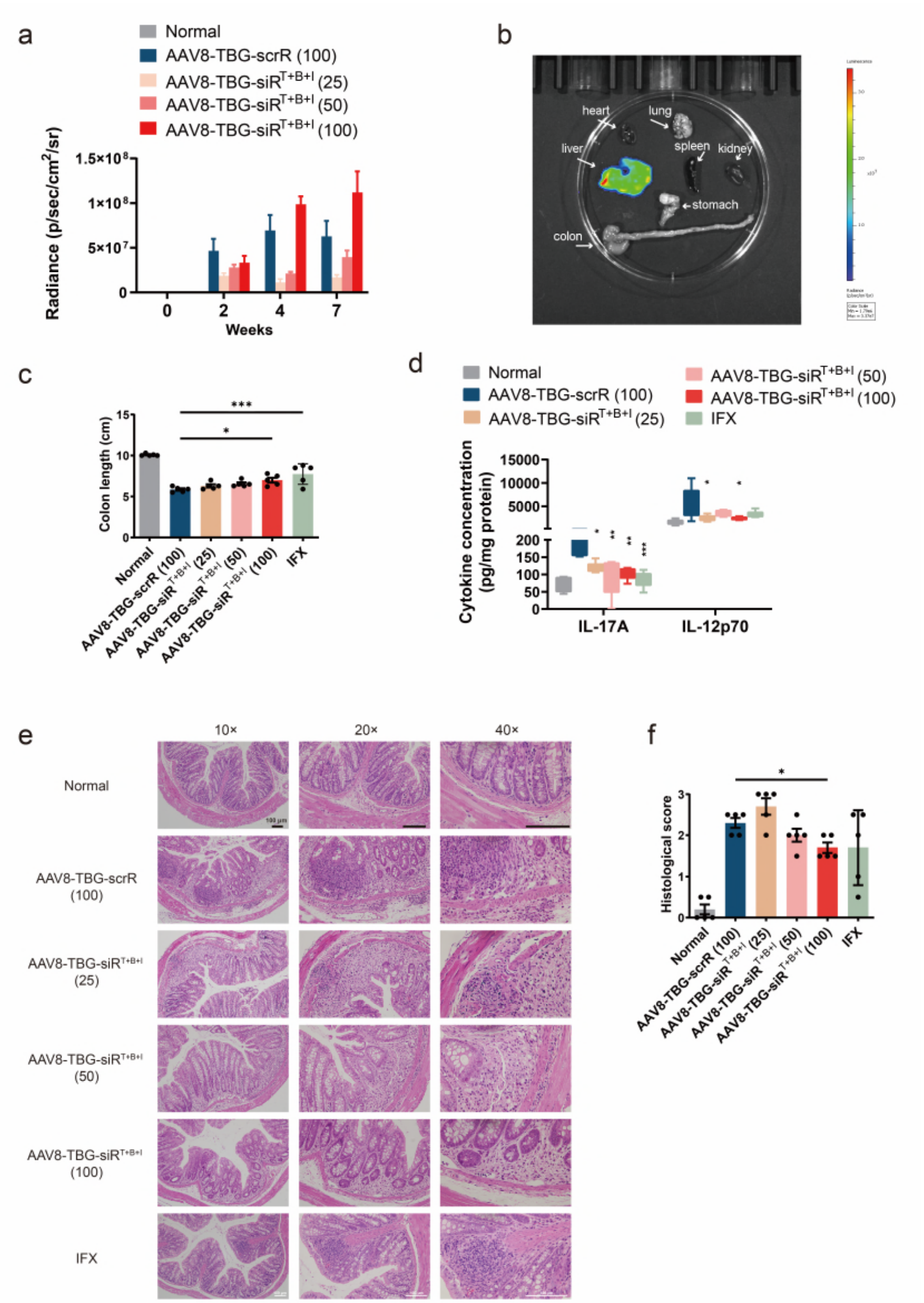

Supplementary Figure 19. Intravenous injection of the AAV8-TBG-siR^{T+B+I} induces long-term combination therapy in the DSS-induced chronic UC model. On week 0, chronic UC was induced by rhythmically administering to BALB/c mice 2.5% DSS for 1 week and water for 2 weeks and the cycle was repeated for 3 times. At the same time, mice were intravenously injected with 100 μL AAV8-TBG-scrR (3.0 × 10^{12} V. G/mL) or 25, 50 or 100 μL AAV8-TBG-siR^{T+B+I} (3.0 × 10^{12} V. G/mL). Four days after each DSS drinking, mice were intravenously injected with

10 mg/kg infliximab for a total of 3 times, once every 2 days. Untreated BALB/c mice were included as normal controls. Body weights were monitored every two days, and the symptoms and histology were evaluated at week 7. **(a)** Evaluation of AAV-mediated luciferase expression to reflect coexpressed siRNA accumulation *in vivo* (n = 6 in each group). **(b)** Evaluation of AAV-mediated luciferase expression in various tissues of mice. **(c)** Mean colon length (n = 5 in each group). **(d)** Determination of serum levels of IL-6, IL-17A and IL-12p70 by ELISA (n = 5 in each group). **(e)** Representative images of H&E staining of colon sections. Scale bar: 100 µm. **(f)** Histological scores of colon sections (n = 5 in each group). Values are presented as the mean ± SEM. Significance was determined using one-way ANOVA followed by Dunnett's multiple comparison. * $p < 0.05$; ** $p < 0.01$; *** $p < 0.005$.

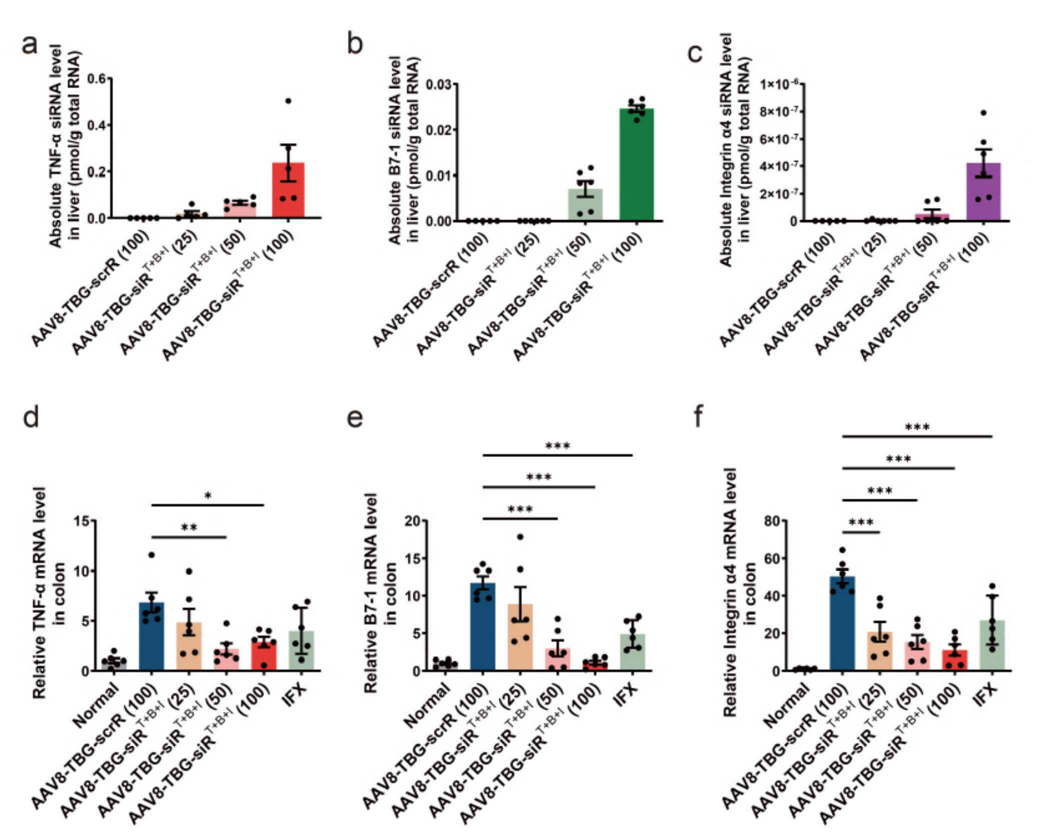

Supplementary Figure 20. Intravenous injection of the AAV8-TBG-siR^{T+B+I} results in accumulation of TNF-α siRNA, B7-1 siRNA and integrin α4 siRNA *in vivo* and silencing of target genes in the colon. (a-c) Quantitative RT-PCR analysis of the absolute expression levels of TNF-α siRNA, B7-1 siRNA and integrin α4 siRNA in the liver (n = 6 in each group). **(d-f)** Quantitative RT-PCR analysis of the absolute expression levels of TNF-α mRNA, B7-1 mRNA and integrin α4 mRNA in the colon (n = 5-6 in each group). Values are presented as the mean ± SEM. Significance was determined using one-way ANOVA followed by Dunnett's multiple comparison. * $p < 0.05$; ** $p < 0.01$; *** $p < 0.005$.

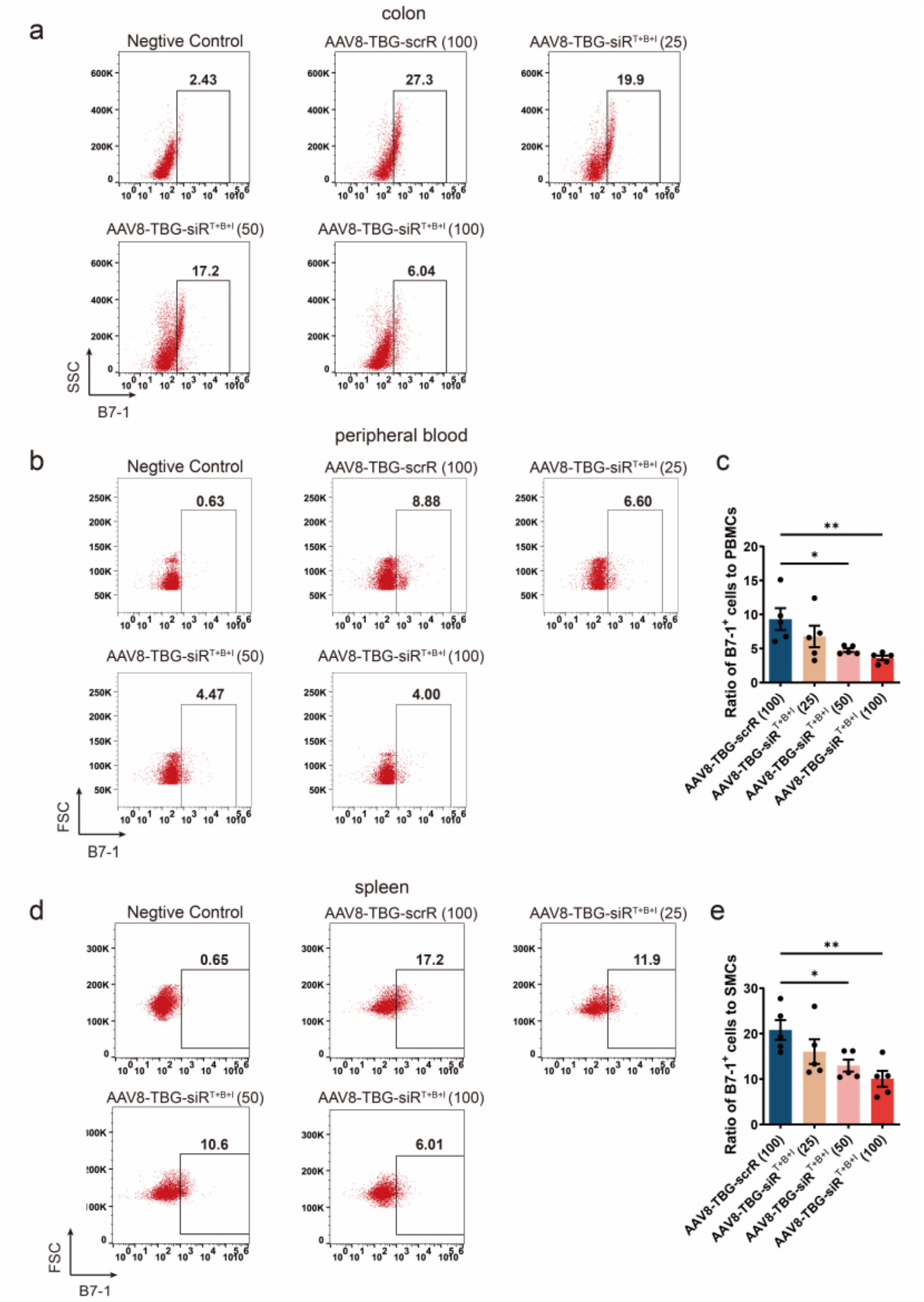

Supplementary Figure 21. Intravenous injection of the AAV8-TBG-siR^{T+B+I} results in loss of B7-1 protein on the membrane surface of mononuclear cells. (a) Representative flow cytometric plots of B7-1 on the surface of colonic lamina propria mononuclear cells. IgG isotype-labelled cells was used as a negative control. **(b)** Representative flow cytometric plots of B7-1 on the surface of peripheral blood mononuclear cells. IgG isotype-labelled cells was used as a negative control. **(c)** The population of B7-1^{+} cells in peripheral blood mononuclear cells (n = 5 in

each group). **(d)** Representative flow cytometric plots of B7-1 on the surface of splenic mononuclear cells. IgG isotype-labelled cells was used as a negative control. **(e)** The population of B7-1^{+} cells in splenic mononuclear cells (n = 5 in each group). Values are presented as the mean ± SEM. Significance was determined using one-way ANOVA followed by Dunnett's multiple comparison. * p < 0.05; ** p < 0.01.

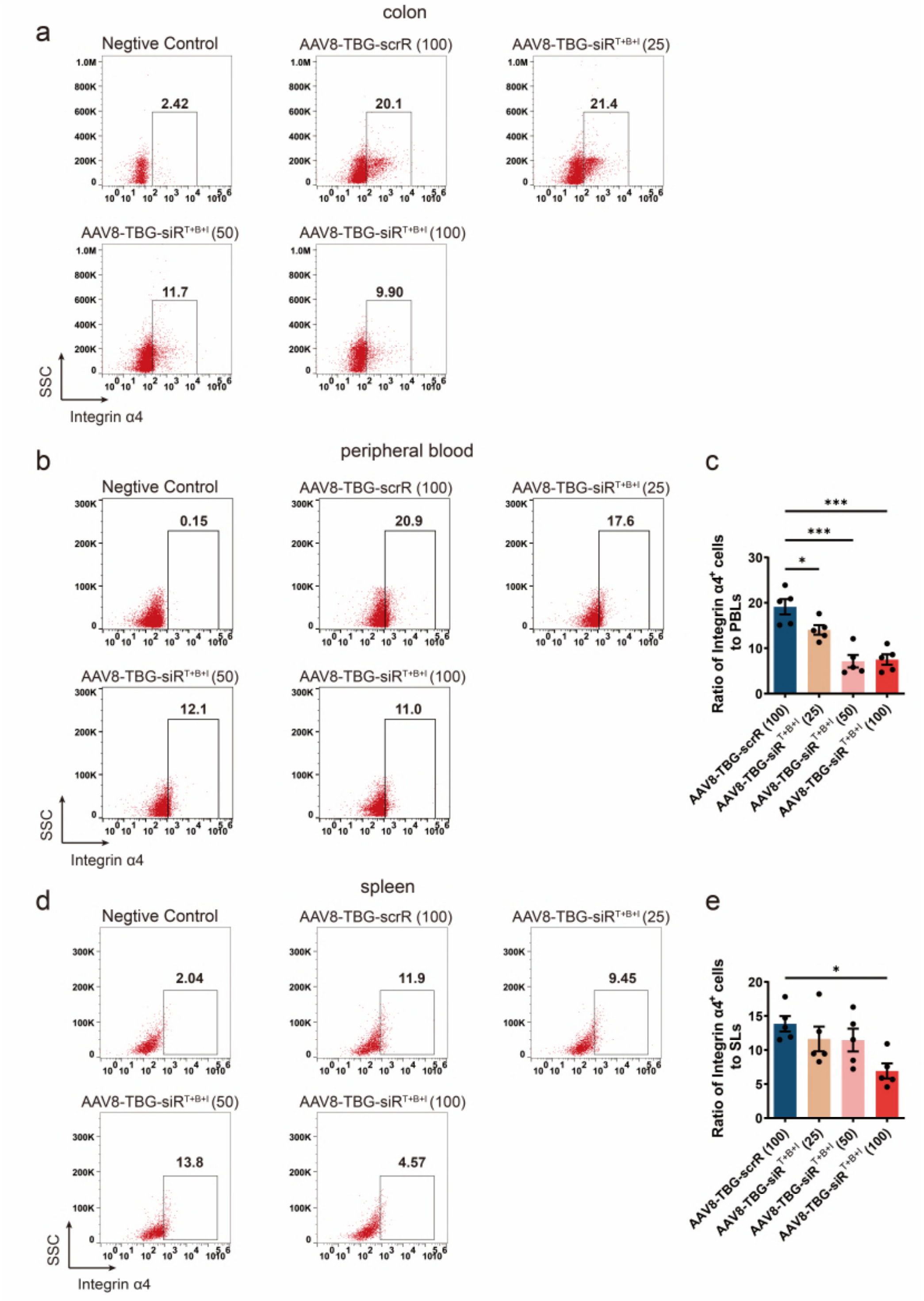

Supplementary Figure 22. Intravenous injection of the AAV8-TBG-siR^{T+B+I} results in loss of integrin α4 protein on the membrane surface of mononuclear cells. (a) Representative flow cytometric plots of integrin α4 on the surface of mononuclear cells derived from the colonic lamina propria. IgG isotype-labelled cells was used as a negative control. **(b)** Representative flow cytometric plots of integrin α4 on the surface of peripheral blood lymphocytes. IgG isotype-labelled cells was used as a negative control. **(c)** The population of integrin α4^{+} cells in peripheral blood lymphocytes (n = 5 in each group). **(d)** Representative flow cytometric plots of integrin α4 on the surface of splenic lymphocytes. IgG isotype-labelled cells was used as a negative control. **(e)** The population of integrin α4^{+} cells in splenic lymphocytes (n = 5 in each group). Values are presented as the mean ± SEM. Significance was determined using one-way ANOVA followed by Dunnett's multiple comparison. * $p < 0.05$; *** $p < 0.005$.

3. In the mouse model, the use of a CMV siRNA circuit and more importantly of the AAV circuit did not lead to any cell toxicity or other side effects. However, considering that only the highest dose of genetic circuits resulted in an improved outcome compared to infliximab, could the authors state whether there are any known concerns when translating this approach into the clinic. Especially with regard to the clinically more relevant AAV-circuit, it would be interesting if administration of the required high doses induces issues. Also, are severe side effects known when using a long-term treatment approach?

Response: We thank the reviewer for pointing out this important issue. Because AAV8 induced long-term transgene expression in the liver, we assessed whether AAV8-TBG-mediated production, assembly and delivery of siRNAs produced toxic effects in certain tissues, especially the liver (new Supplementary Fig. 23). The mice treated with different doses of AAV8-TBG-siR^{T+B+I}, like AAV8-TBG-scrR-treated mice and untreated normal mice, did not exhibit apparent toxicity in the liver and other tissues. A panel of 6 serum biochemical parameters remained within the normal range for each group, except ALT (a sensitive marker of hepatocyte necrosis or damage) exhibited a gradual increasing trend after treatment with increasing amounts of AAV8-TBG-siR^{T+B+I}, but without statistical significance (Supplementary Fig. 23a-23f). Histological analyses also detected no pathological changes or signs of inflammation in the liver and kidney sections derived from AAV8-TBG-siR^{T+B+I}-treated mice (Supplementary Fig. 23g). Overall, these results suggest AAV8-TBG-mediated liver expression and assembly of siRNAs has an acceptable safety profile.

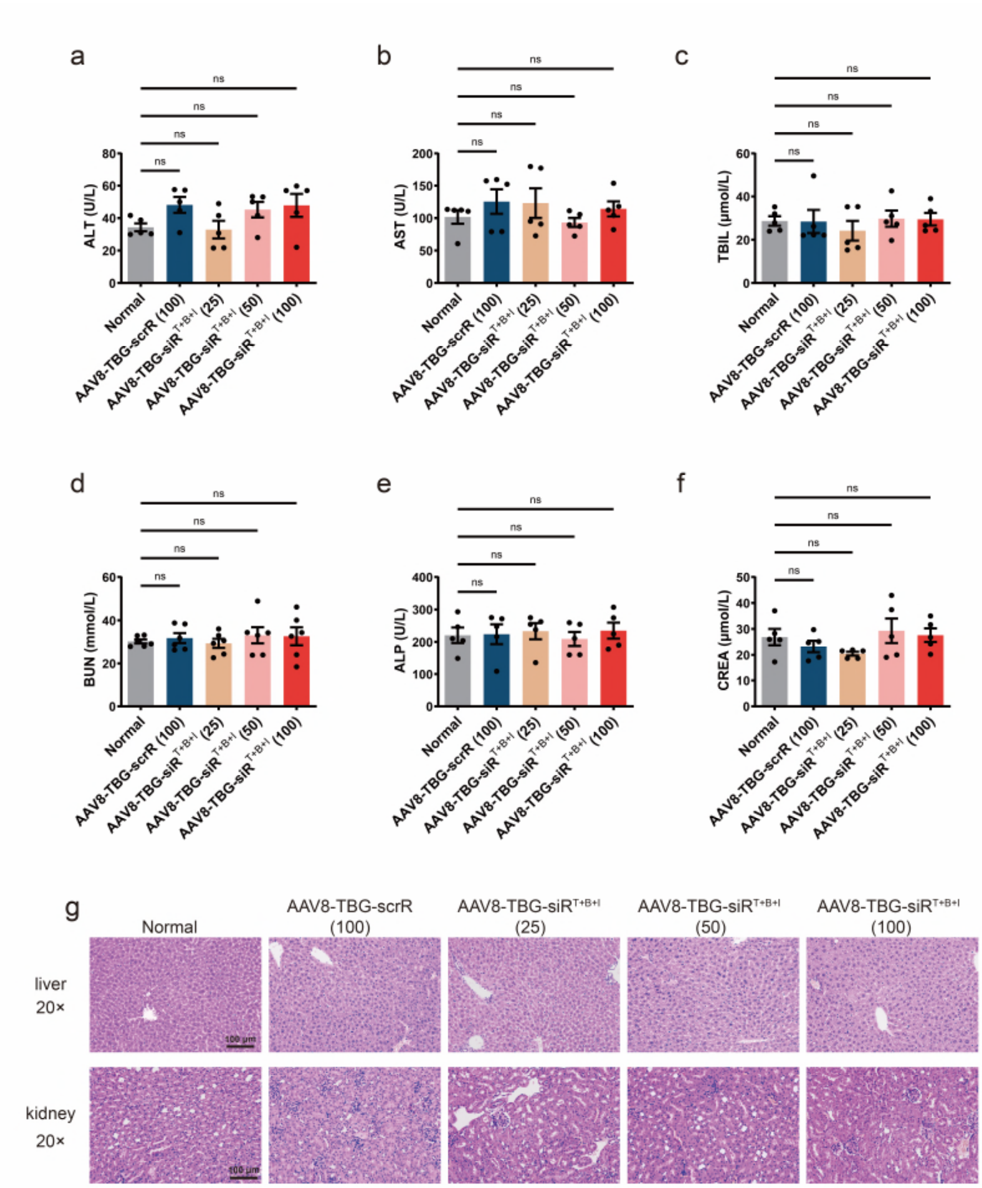

Supplementary Figure 23. Evaluation of the toxic effects and tissue damage in mice after intravenous injection of AAV8-TBG-siR^{T+B+I}. On week 0, BALB/c mice were intravenously injected with 100 μL AAV8-TBG-scrR (3.0 × 10^{12} V. G/mL) or 25, 50 or 100 μL AAV8-TBG-siR^{T+B+I} (3.0 × 10^{12} V. G/mL). At the same time, chronic UC was induced by rhythmically administering to mice 2.5% DSS for 1 week and water for 2 weeks and the cycle was repeated for 3 times. Untreated BALB/c mice were included as normal controls. After the treatment, mice were sacrificed, and blood and tissue samples were collected and analysed for serum biochemical indicators and tissue damage. **(a-f)** Measurement of representative serum biochemical indicators, including alanine aminotransferase (ALT), aspartate aminotransferase (AST), total bilirubin (TBIL), blood urea nitrogen (BUN), alkaline phosphatase (ALP) and creatinine (CREA), in the serum. **(g)** Histological examination of the liver and kidney. Scale bar: 100 μm. Values are presented as the mean ± SEM. Significance was determined using one-way ANOVA followed by Dunnett's multiple comparison. NS, not significant.

Furthermore, we have modified the Discussion section of the revised manuscript to emphasize the critical issue about long-term treatment of chronic UC with AAV-driven genetic circuits, which reads as follows: "Considering the short *in vivo* half-life of the genetic circuits formed as naked DNA plasmids, repeated injection of genetic circuits is inevitable for the long-term treatment of chronic UC. The problems associated with repeated injection should be dealt with properly.[24] To develop a strategy for permanent self-assembly of sEV-enclosed siRNAs for the treatment of chronic UC, we selected AAV as the carrier of genetic circuits because AAV is capable of establishing long-term transgene expression with minimal immunogenicity, toxicity and side effects.[25] A growing number of human clinical trials have used AAVs, achieving a good safety profile and significant clinical benefit in many diseases.[26] The advantage of AAV-driven genetic circuits is that they can carry multiple distinct siRNA expression cassettes targeting different causal genes of chronic UC to improve therapeutic outcomes via a single administration. We showed that AAV9-CMV- and AAV8-TBG-mediated production and assembly of multiple siRNAs caused substantial and lasting inhibition of the corresponding target genes in chronic UC models. The therapeutic benefit was comparable between a single injection of the AAV-driven genetic circuits and repeated injections of the plasmid-based genetic circuits. Although the AAV9 vector and CMV promoter exhibit high efficiency in a broad range of tissues, the AAV8 vector and TBG promoter have a strong liver tropism, which validates that the liver is the original site for the uptake of genetic circuits and self-assembly and secretion of siRNA-encapsulating sEVs. We confirmed that peripheral infusion of an AAV8-TBG-driven genetic circuit constantly generated self-assembled siRNAs in a safe, non-toxic and biocompatible manner. However, the expression levels of TNF-α siRNA, B7-1 siRNA and integrin α4 siRNA were decreased in the liver when the CMV promoter was replaced with a liver-specific TBG promoter in the genetic circuits, regardless of whether the genetic circuits were placed in an AAV or plasmid. This difference may be due to the lower efficiency of the TBG promoter in transcribing siRNAs than the CMV promoter, which is consistent with the results from previous studies. Therefore, although the AAV8-TBG-driven genetic circuit yielded promising therapeutic outcomes in DSS-induced chronic UC models, there is much room for improvement. Nevertheless, our results provide broad insight into how a liver-tropic, AAV-based RNAi therapeutic strategy alleviated disease progression and promoted immune balance recovery in chronic UC, which led to mucosal restoration in disease sites and reduced the likelihood of adverse side effects. Although more work is needed to verify the therapeutic effects and ensure safety, AAV-driven genetic circuits hold strong promise for becoming a new option for the treatment of complex chronic diseases, such as UC."

4. Figure 5: The legend currently describes an acute UC model, however, based on Fig 5a it should possibly say "the cycle was repeated for 3 times" (line 708).

Response: We apologize for this error. Indeed, we have evaluated the therapeutic effects of AAV-CMV-siR^{T+B+I} in a chronic UC model. We have corrected this error and double-checked the revised manuscript to exclude such errors.

5. Figure 5: Suppression of B7-1 and Integrin a4 is shown as suppression of mRNA. For completion, addition of the flow data to show suppression of protein expression as done in Fig. 4 would be recommended.

<u>Response</u>: This is an excellent suggestion. According to reviewer's suggestion, we have measured the protein levels of B7-1 and integrin α4 on the membrane surface of mononuclear cells derived from AAV8-TBG-siR^{T+B+I}-treated mice using flow cytometry (new Fig. 8k and 8l and Supplementary Fig. 21 and 22). As expected, flow cytometry confirmed a dose-dependent loss of B7-1 and integrin α4 proteins on the membrane surface of mononuclear cells derived from the colonic lamina propria, peripheral blood and spleen of AAV8-TBG-siR^{T+B+I}-treated mice (Fig. 8k and 8l and Supplementary Fig. 21 and 22).

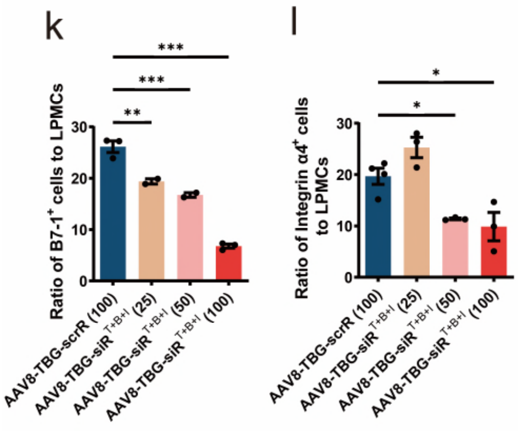

Figure 8. (k) The population of B7-1^{+} cells in total colonic lamina propria mononuclear cells (n = 2-3 in each group). **(l)** The population of integrin α4^{+} lymphocytes in total colonic lamina propria lymphocytes (n = 3-4 in each group). Values are presented as the mean ± SEM. Significance was determined using one-way ANOVA followed by Dunnett's multiple comparison. * $p < 0.05$; ** $p < 0.01$; *** $p < 0.005$.

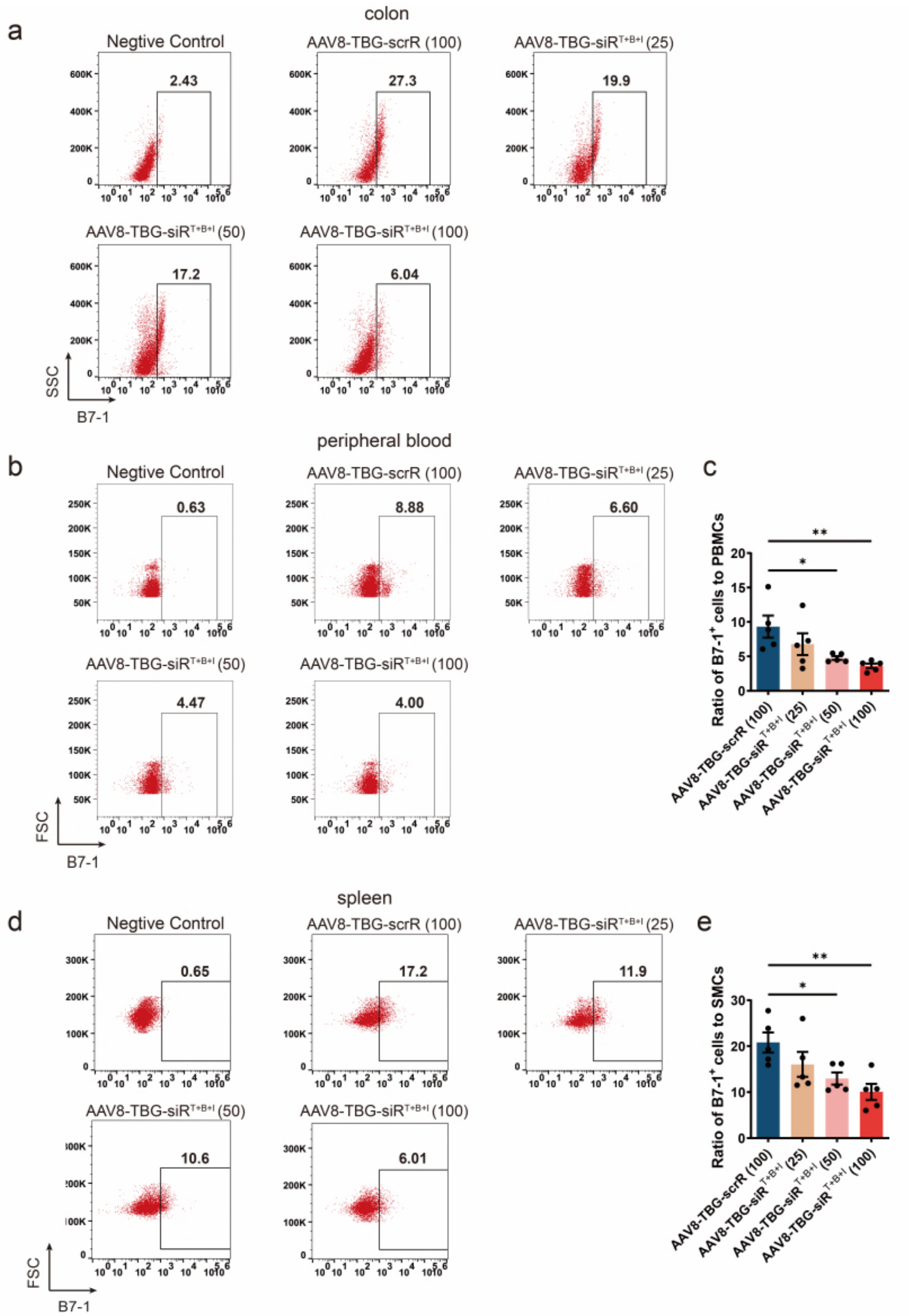

Supplementary Figure 21. Intravenous injection of the AAV8-TBG-siR^{T+B+I} results in loss of B7-1 protein on the membrane surface of mononuclear cells. (a) Representative flow cytometric plots of B7-1 on the surface of colonic lamina propria mononuclear cells. IgG isotype-labelled cells was used as a negative control. **(b)** Representative flow cytometric plots of B7-1 on the surface of peripheral blood mononuclear cells. IgG isotype-labelled cells was used as a negative control. **(c)** The population of B7-1^{+} cells in peripheral blood mononuclear cells (n = 5 in

each group). **(d)** Representative flow cytometric plots of B7-1 on the surface of splenic mononuclear cells. IgG isotype-labelled cells was used as a negative control. **(e)** The population of B7-1^{+} cells in splenic mononuclear cells (n = 5 in each group). Values are presented as the mean ± SEM. Significance was determined using one-way ANOVA followed by Dunnett's multiple comparison. * p < 0.05; ** p < 0.01.

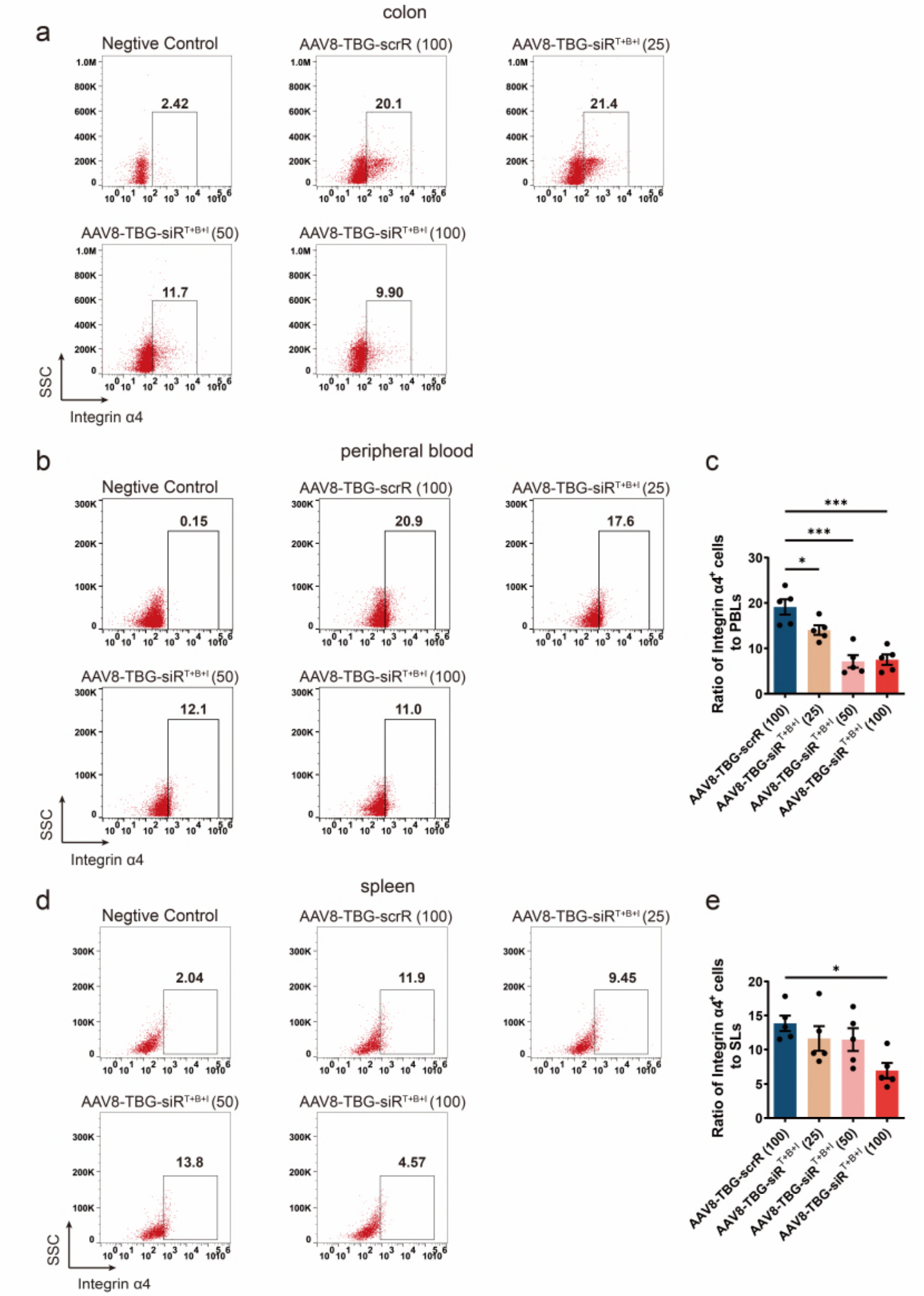

Supplementary Figure 22. Intravenous injection of the AAV8-TBG-siR^{T+B+I} results in loss of integrin α4 protein on the membrane surface of mononuclear cells. (a) Representative flow cytometric plots of integrin α4 on the surface of mononuclear cells derived from the colonic lamina propria. IgG isotype-labelled cells was used as a negative control. **(b)** Representative flow cytometric plots of integrin α4 on the surface of peripheral blood lymphocytes. IgG isotype-labelled cells was used as a negative control. **(c)** The population of integrin $\alpha4^+$ cells in peripheral blood lymphocytes (n = 5 in each group). **(d)** Representative flow cytometric plots of integrin α4 on the surface of splenic lymphocytes. IgG isotype-labelled cells was used as a negative control. **(e)** The population of integrin $\alpha4^+$ cells in splenic lymphocytes (n = 5 in each group). Values are presented as the mean ± SEM. Significance was determined using one-way ANOVA followed by Dunnett's multiple comparison. * $p < 0.05$; *** $p < 0.005$.

6. Sup Fig. 7: Toxicity should be measured at the same concentration as in the actual experiment (eg. 10mg/kg body weight for the CMV-circuit) instead of only using 5mg/kg body weight.

Response: We appreciate reviewer's emphasis on this critical issue. We have evaluated the potential side effects and tissue toxicity of the genetic circuits at the same concentration (20 mg/kg) as in the therapeutic experiment. Chronic UC was induced in male BALB/c mice by rhythmically administering to mice 2.5% DSS for 1 week and water for 2 weeks and the cycle was repeated for 3 times. Four days after each DSS drinking, mice were intravenously injected with PBS or with equal dose (20 mg/kg) of CMV-scrR, CMV-siR$^{TNF-\alpha}$ or CMV-siR^{T+B+I} circuit for a total of 3 times, once every 2 days. After the treatment, toxic effects were evaluated by histological examination of tissues and measurement of representative serum biochemical indicators. No hepatic and renal toxicity and abnormal alterations in serum biochemical indicators were observed after repeated injections with 20 mg/kg CMV-siR$^{TNF-\alpha}$ or CMV-siR^{T+B+I} circuit. These results suggest that injection of 20 mg/kg genetic circuits can generate self-assembled siRNAs in a safe, non-toxic and biocompatible manner. We have replaced original version of Supplementary Figure 7 with these new data.

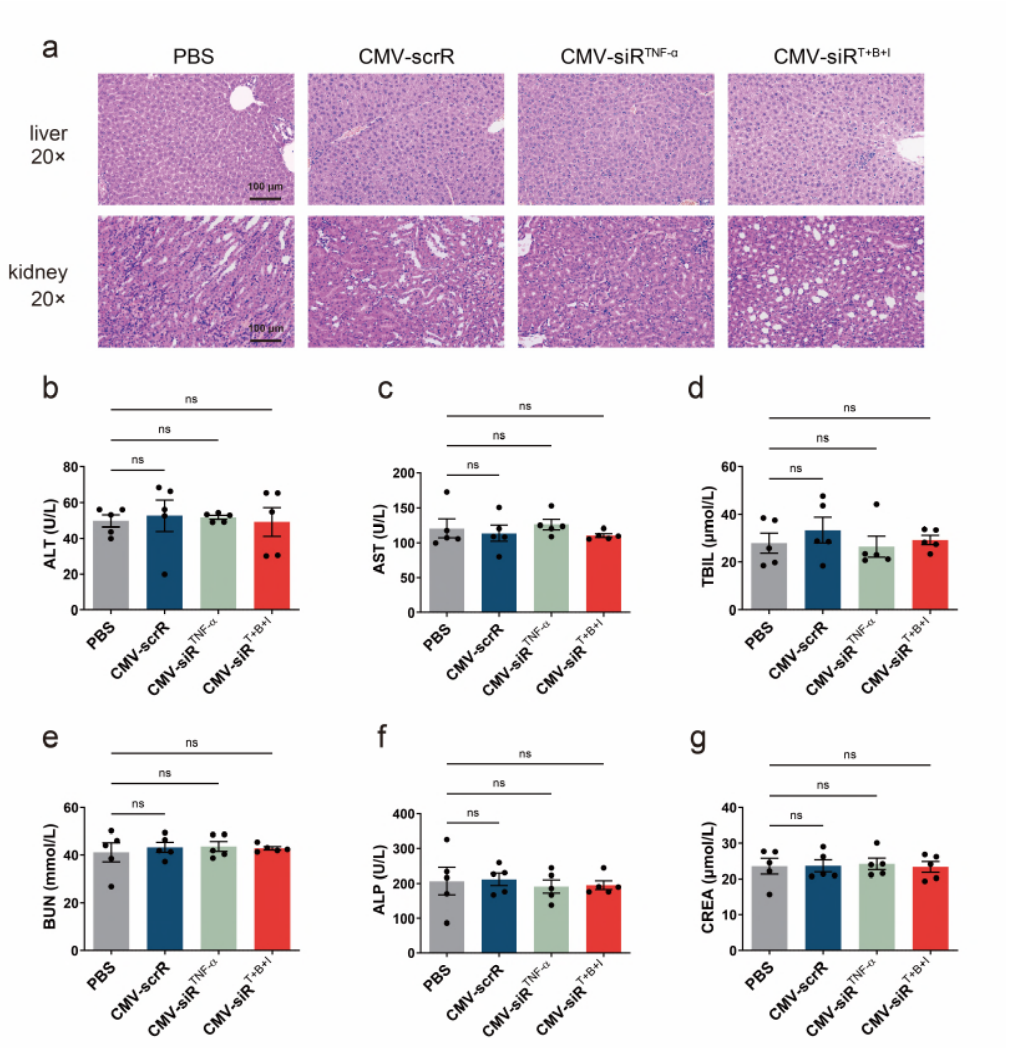

Supplementary Figure 7. Evaluation of the toxic effects and tissue damage in mice after intravenous injection of the genetic circuits. Chronic UC was induced in male BALB/c mice by rhythmically administering to mice 2.5% DSS for 1 week and water for 2 weeks and the cycle was repeated for 3 times. Four days after each DSS drinking, mice were intravenously injected with PBS or with equal dose (20 mg/kg) of CMV-scrR, CMV-siR$^{TNF-\alpha}$ or CMV-siR^{T+B+I} circuit for a total of 3 times, once every 2 days. Twelve hours after the last injection, mice were sacrificed, and blood and tissue samples were collected and analysed for serum biochemical indicators and tissue damage. **(a)** Histological examination of the liver and kidney. Scale bar: 100 μm. **(b-g)** Measurement of representative serum biochemical indicators, including alanine aminotransferase (ALT), aspartate aminotransferase (AST), total bilirubin (TBIL), blood urea nitrogen (BUN), alkaline phosphatase (ALP) and creatinine (CREA), in the serum. Values are presented as the mean ± SEM. Significance was determined using one-way ANOVA followed by Dunnett's multiple comparison. NS, not significant.

7. Sup Fig. 8f: Please use the same graph style for cytokine measurement as in the previous figures.

Response: We thank the reviewer for pointing out this critical issue. We have modified original Supplementary Figure 8f (new Supplementary Figure 8f) to make sure that the same graph style is used for cytokine measurement.

8. Figure 2 shows that apart from the colon, siRNA loaded exosomes can also be detected in the kidney, spleen, and peripheral blood monocytes and $CD4^+$ T-cells. While experimental animals in this study were kept under pathogen-free conditions, there is a certain risk that the triple siRNA treatment renders animals/patients highly susceptible to opportunistic infections. This concern will have to be addressed when future research in this direction is considered.

Response: We understand reviewer's concern regarding this issue. We completely agree with the reviewer that the increased risk of opportunistic infections associated with combined immunosuppression needs to be defined more precisely. We have added a whole paragraph to the Discussion section of the revised manuscript to emphasize this critical safety concern, which reads as follows: "Biological therapies, particularly anti-TNF-α agents (e.g., infliximab) and anti-integrin agents (e.g., natalizumab), have substantially extended the therapeutic armamentarium of IBD in the last decade. Combined immunosuppression using biological therapies and immunomodulators has become standard in the medical management of moderate-to-severe IBD because of clearly demonstrated efficacy.[27] However, the combination of immunosuppressive medications is also a risk factor for opportunistic infections. For example, infliximab-treated patients are susceptible to infections with opportunistic pathogens, including *Mycobacterium tuberculosis*, *Histoplasma capsulatum*, *Coccidioides immitis*, *Pneumocystis* and *Cytomegalovirus*.[28] Positive prevention, regular monitoring and timely control of opportunistic infections are the current focuses for improving the prognosis of IBD patients.[29] Therefore, a combination therapy towards multiple immunological targets may function as a "double-edged sword". Although combination therapy may suppress inflammatory responses more rapidly and effectively, it may increase the risk of opportunistic infections. Therefore, an alternative strategy that enhances the immunomodulatory effects and minimises the undesired side effects should be developed. The present study raised the possibility that simultaneous inhibition of TNF-α, B7-1 and integrin α4 with multiple self-assembled siRNAs, via a distinct mechanism other than biological therapies and immunomodulators, rapidly relieved intestinal inflammation and exerted a synergistic therapeutic effect against UC. We did not observe abnormal phenotypes, apparent tissue damage or severe side effects in the liver, spleen, lung or kidney after the injection of genetic circuits, although self-assembled siRNAs were also delivered to the kidney, spleen and peripheral blood monocytes and $CD4^+$ T cells apart from the colon. These results suggest that *in vivo* self-assembled siRNA is a promising combination therapeutic strategy for UC with an acceptable safety profile. However, the animal models were maintained under specific pathogen-free conditions in this study, and there is a certain risk that the multi-targeted self-assembled siRNAs may induce excessive immunosuppression and render the animals/patients highly susceptible to opportunistic infections in the real world. Therefore, the concerns associated with the combined immunosuppression mediated by self-assembled siRNAs should be addressed properly. First, the safety, dosing and durability of self-assembled siRNAs must be adequately fine-tuned. Second, the biodistribution of siRNAs in various tissues and immune cells must be measured more accurately, and whether the physiological functions and status of these tissues and

immune cells are impaired after long-term therapy should be evaluated more systematically. Third, to confer targeting capability on sEVs, the guiding peptides that modify the membrane-anchored proteins of sEVs should be specifically designed and integrated into the genetic circuits to endow the modified sEVs with the ability to target immune cells with a high affinity and facilitate the preferential delivery of siRNAs into immune cells to minimise undesired off-target effects."

Reviewer #4

1. The conclusions that the mechanism underlying the significant therapeutic effect observed is liver-mediated transduction and secretion of exosomes seems stretched. To conclude this the authors should have used a liver-specific promoter rather than CMV, and AAV8 that targets primarily mouse hepatocytes upon iv injection rather than AAV9 which diffuses quite broadly. In the absence of data using a liver-specific approach, claims that the effects observed are liver-dependent should be moderated. The authors should acknowledge that transduction of other tissues in addition to liver can contribute to the effect observed. Along this line, a short-term (i.e. 2-3 hours after injection) DNA biodistribution study should be performed to assess which are the tissues originally targeted/infected by either the plasmid or the AAV9 vector, and presumably transduced since the CMV promoter is used.

Response: We greatly thank the reviewer for this constructive suggestion. Although we optimised the delivery vehicles of the genetic circuit and developed an AAV9-based strategy for permanent self-assembly of sEV-enclosed siRNAs for the treatment of UC, the therapeutic effects observed in UC models may not be liver dependent because AAV9 has tropism for a wide range of tissues, and the CMV promoter is a strong constitutive promoter without tissue specificity. AAV8 has strong liver tropism,[22,23] and the thyroxine-binding globulin (TBG) promoter is a hepatocyte-specific promoter.[20,21] Therefore, the expression cassette designed for tandem expression of TNF-α siRNA, B7-1 siRNA and integrin α4 siRNA was inserted downstream of the TBG promoter, and the entire circuit was further incorporated into an AAV8 vector (hereafter, AAV8-TBG-siR^{T+B+I}). Such an AAV8-TBG-driven genetic circuit ensures that the corresponding siRNAs are transcribed only in hepatocytes while avoiding undesired siRNA expression in extrahepatic cells.

We evaluated the therapeutic effects of AAV8-TBG-siR^{T+B+I} in a chronic UC model (Fig. 8a). AAV8-TBG-mediated luciferase expression was dose-dependently increased from weeks 2 to 7 and remained stable over 7 weeks. The luciferase signal was primarily restricted to the liver with negligible signals in extrahepatic tissues (Fig. 8b and Supplementary Fig. 19a and 19b). Control mice receiving AAV8-TBG-scrR showed typical characteristics of colitis in the chronic UC model, such as sustained body weight loss, increased DAI score, shortened colon length, enhanced inflammatory cytokine levels and apparent histological features in colonic sections. However, the mice receiving a high dose of AAV8-TBG-siR^{T+B+I} rapidly recovered the lost body weight and colon length, experienced a low DAI score, showed a significant decline in inflammatory cytokines and exhibited an improved colonic histological appearance (Fig. 8c-8f and Supplementary Fig. 19c-19f). At the molecular level, treatment with AAV8-TBG-siR^{T+B+I} resulted in a dose-dependent production of TNF-α siRNA, B7-1 siRNA and integrin α4 siRNA in the liver

(Supplementary Fig. 20a-20c), which were accompanied by a dose-dependent accumulation of TNF-α siRNA, B7-1 siRNA and integrin α4 siRNA in the colon (Fig. 8g-8i). Consequently, dose-dependent reductions in colonic TNF-α, B7-1 and integrin α4 mRNA levels were detected after AAV8-TBG-siR^{T+B+I} treatment (Supplementary Fig. 20d-20f). ELISA confirmed a dose-dependent decline in TNF-α protein levels in the colonic lamina propria derived from AAV8-TBG-siR^{T+B+I}-treated mice (Fig. 8j), and flow cytometry confirmed a dose-dependent loss of B7-1 and integrin α4 proteins on the membrane surface of mononuclear cells derived from the colonic lamina propria, peripheral blood and spleen of AAV8-TBG-siR^{T+B+I}-treated mice (Fig. 8k and 8l and Supplementary Fig. 21 and 22). Infliximab also exhibited significant therapeutic activity in the chronic UC model, but AAV8-TBG-siR^{T+B+I} ameliorated the manifestations of chronic colitis and restored the expression of TNF-α, B7-1 and integrin α4 to a better extent than infliximab (Fig. 8c-8l and Supplementary Fig. 19c-19f, 20, 21 and 22).

Because AAV8 induced long-term transgene expression in the liver, we assessed whether AAV8-TBG-mediated production, assembly and delivery of siRNAs produced toxic effects in certain tissues, especially the liver. The mice treated with different doses of AAV8-TBG-siR^{T+B+I}, like AAV8-TBG-scrR-treated mice and untreated normal mice, did not exhibit apparent toxicity in the liver and other tissues. A panel of 6 serum biochemical parameters remained within the normal range for each group, except ALT (a sensitive marker of hepatocyte necrosis or damage) exhibited a gradual increasing trend after treatment with increasing amounts of AAV8-TBG-siR^{T+B+I}, but without statistical significance (Supplementary Fig. 23a-23f). Histological analyses also detected no pathological changes or signs of inflammation in the liver and kidney sections derived from AAV8-TBG-siR^{T+B+I}-treated mice (Supplementary Fig. 23g). Overall, these results suggest AAV8-TBG-mediated liver expression and assembly of siRNAs as a potential solution to induce long-term combination therapy for chronic UC, with an acceptable safety profile.

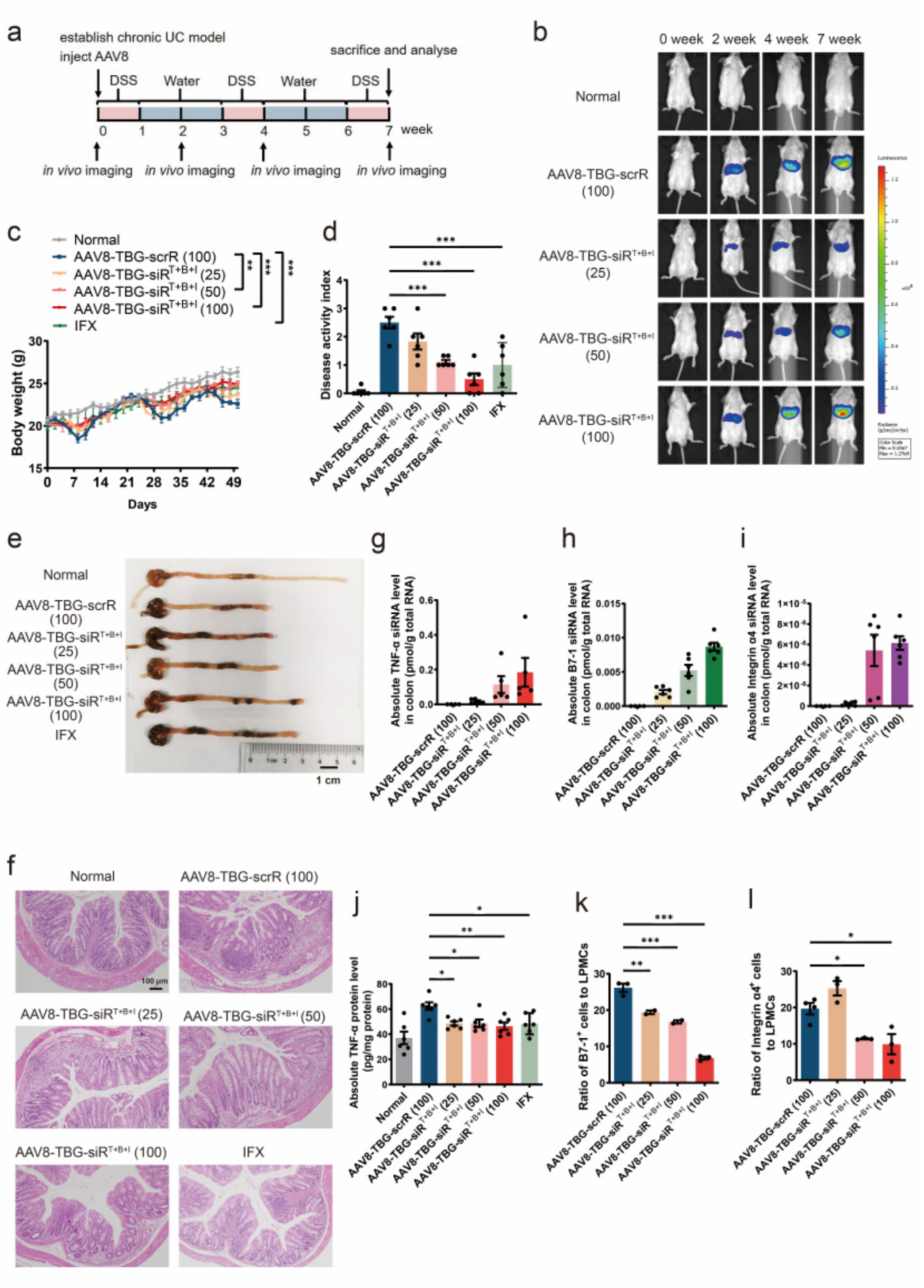

Figure 8. Intravenous injection of the AAV8-TBG-siR^{T+B+I} induces long-term combination therapy in the DSS-induced chronic UC model. (a) Flow chart of the experimental design. On week 0, chronic UC was induced by rhythmically administering to BALB/c mice 2.5% DSS for 1 week and water for 2 weeks, and the cycle was repeated for 3 times. At the same time, mice were intravenously injected with 100 μL AAV8-TBG-scrR (3×10^{12} V. G/mL) or 25, 50 or 100 μL AAV8-TBG-siR^{T+B+I} (3×10^{12} V. G/mL). Four days after each DSS drinking, mice were

intravenously injected with 10 mg/kg infliximab for a total of 3 times, once every 2 days. Untreated BALB/c mice were included as normal controls. Body weights were monitored every two days, and the symptoms and histology were evaluated at week 7. **(b)** Evaluation of AAV-mediated luciferase expression to reflect coexpressed siRNA accumulation *in vivo* (n = 6 in each group). **(c)** Body weight curves (n = 6 in each group). **(d)** DAI scores (n = 6 in each group). **(e)** Representative macroscopic features of colons. Scale bar: 1 cm. **(f)** Representative images of H&E staining of colon sections. Scale bar: 100 μm. **(g-i)** Quantitative RT-PCR analysis of the absolute expression levels of TNF-α siRNA, B7-1 siRNA and integrin α4 siRNA in the colon (n = 5-6 in each group). **(j)** Determination of the absolute expression levels of TNF-α protein in the colon by ELISA (n = 6 in each group). **(k)** The population of B7-1$^+$ cells in total colonic lamina propria mononuclear cells (n = 2-3 in each group). **(l)** The population of integrin α4$^+$ lymphocytes in total colonic lamina propria lymphocytes (n = 3-4 in each group). Values are presented as the mean ± SEM. Significance was determined using one-way ANOVA followed by Dunnett's multiple comparison in panel d, j, k and l or using two-way ANOVA followed by Dunnett's multiple comparison in panel c. * $p < 0.05$; ** $p < 0.01$; *** $p < 0.005$.

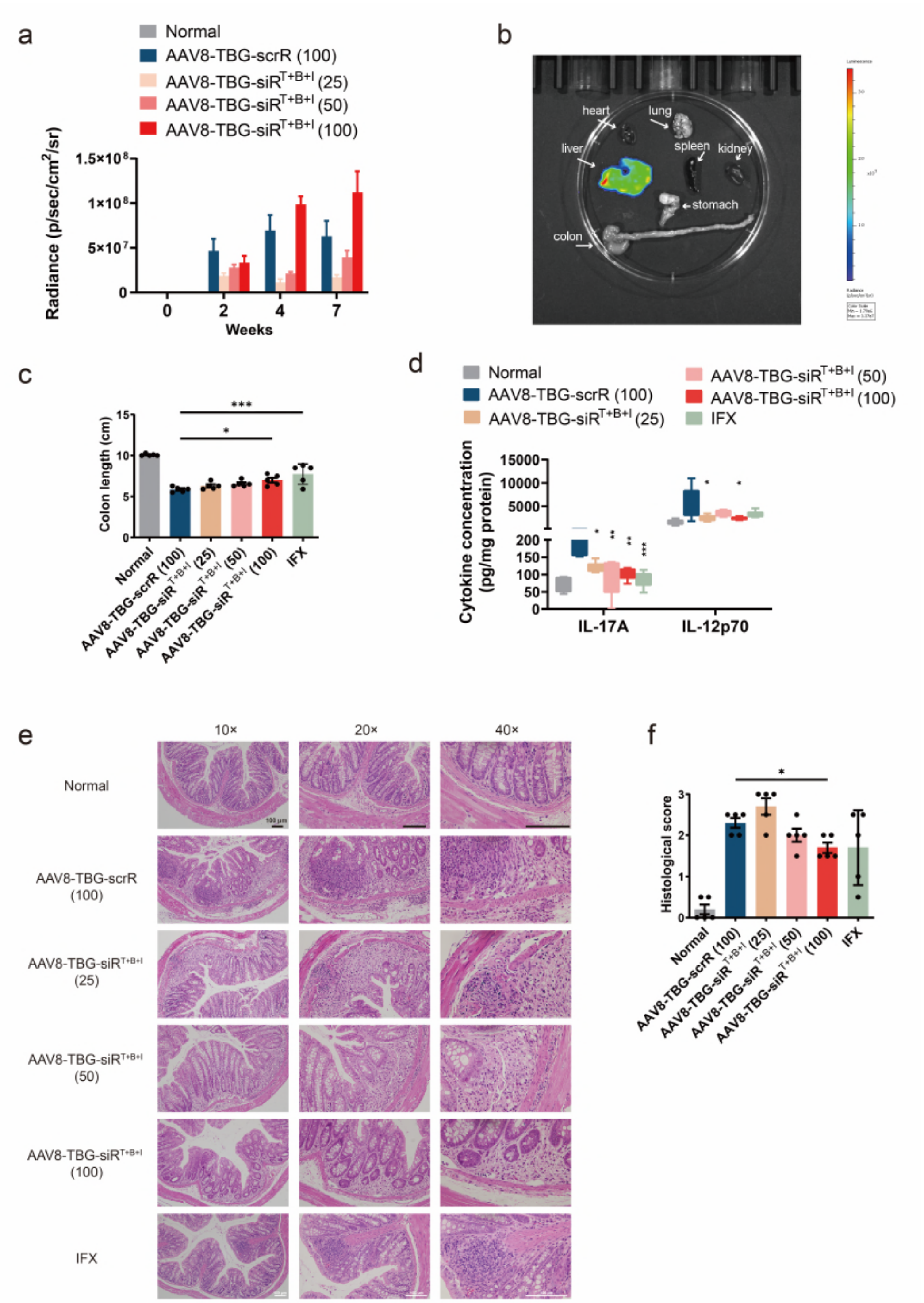

Supplementary Figure 19. Intravenous injection of the AAV8-TBG-siR^{T+B+I} induces long-term combination therapy in the DSS-induced chronic UC model. On week 0, chronic UC was induced by rhythmically administering to BALB/c mice 2.5% DSS for 1 week and water for 2 weeks and the cycle was repeated for 3 times. At the same time, mice were intravenously injected with 100 μL AAV8-TBG-scrR (3.0×10^{12} V. G/mL) or 25, 50 or 100 μL AAV8-TBG-siR^{T+B+I} (3.0×10^{12} V. G/mL). Four days after each DSS drinking, mice were intravenously injected with

10 mg/kg infliximab for a total of 3 times, once every 2 days. Untreated BALB/c mice were included as normal controls. Body weights were monitored every two days, and the symptoms and histology were evaluated at week 7. **(a)** Evaluation of AAV-mediated luciferase expression to reflect coexpressed siRNA accumulation *in vivo* (n = 6 in each group). **(b)** Evaluation of AAV-mediated luciferase expression in various tissues of mice. **(c)** Mean colon length (n = 5 in each group). **(d)** Determination of serum levels of IL-6, IL-17A and IL-12p70 by ELISA (n = 5 in each group). **(e)** Representative images of H&E staining of colon sections. Scale bar: 100 µm. **(f)** Histological scores of colon sections (n = 5 in each group). Values are presented as the mean ± SEM. Significance was determined using one-way ANOVA followed by Dunnett's multiple comparison. * p < 0.05; ** p < 0.01; *** p < 0.005.

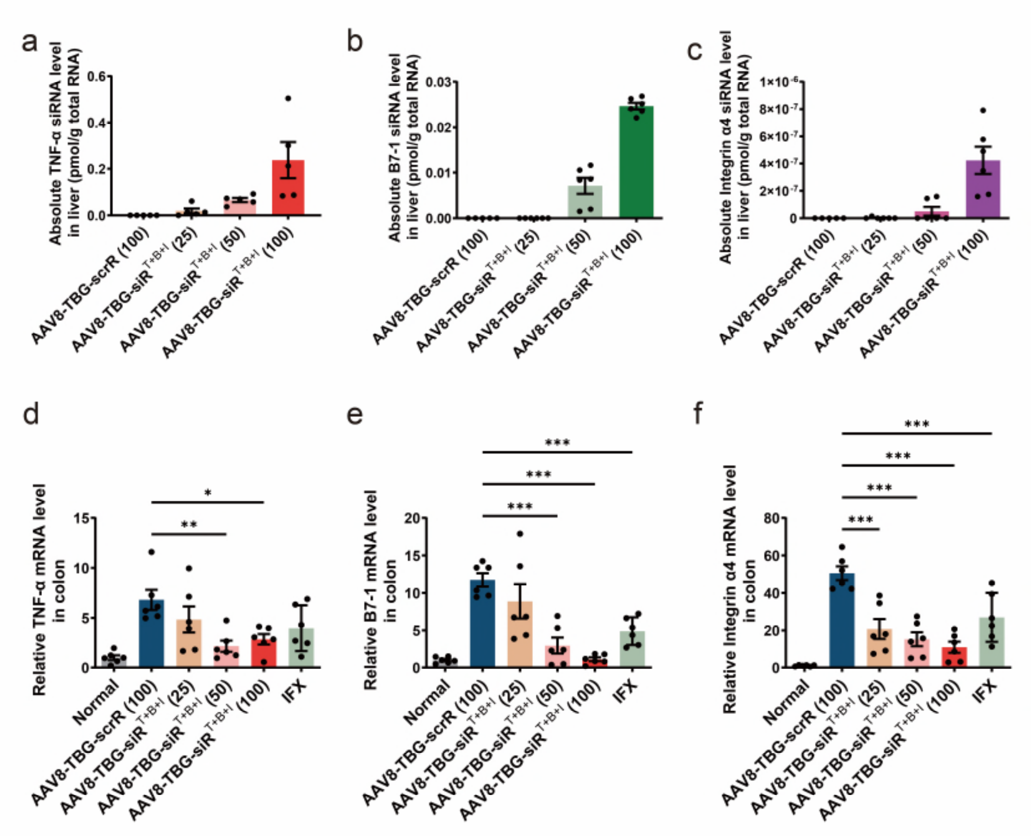

Supplementary Figure 20. Intravenous injection of the AAV8-TBG-siR^{T+B+I} results in accumulation of TNF-α siRNA, B7-1 siRNA and integrin α4 siRNA *in vivo* and silencing of target genes in the colon. (a-c) Quantitative RT-PCR analysis of the absolute expression levels of TNF-α siRNA, B7-1 siRNA and integrin α4 siRNA in the liver (n = 6 in each group). **(d-f)** Quantitative RT-PCR analysis of the absolute expression levels of TNF-α mRNA, B7-1 mRNA and integrin α4 mRNA in the colon (n = 5-6 in each group). Values are presented as the mean ± SEM. Significance was determined using one-way ANOVA followed by Dunnett's multiple comparison. * p < 0.05; ** p < 0.01; *** p < 0.005.

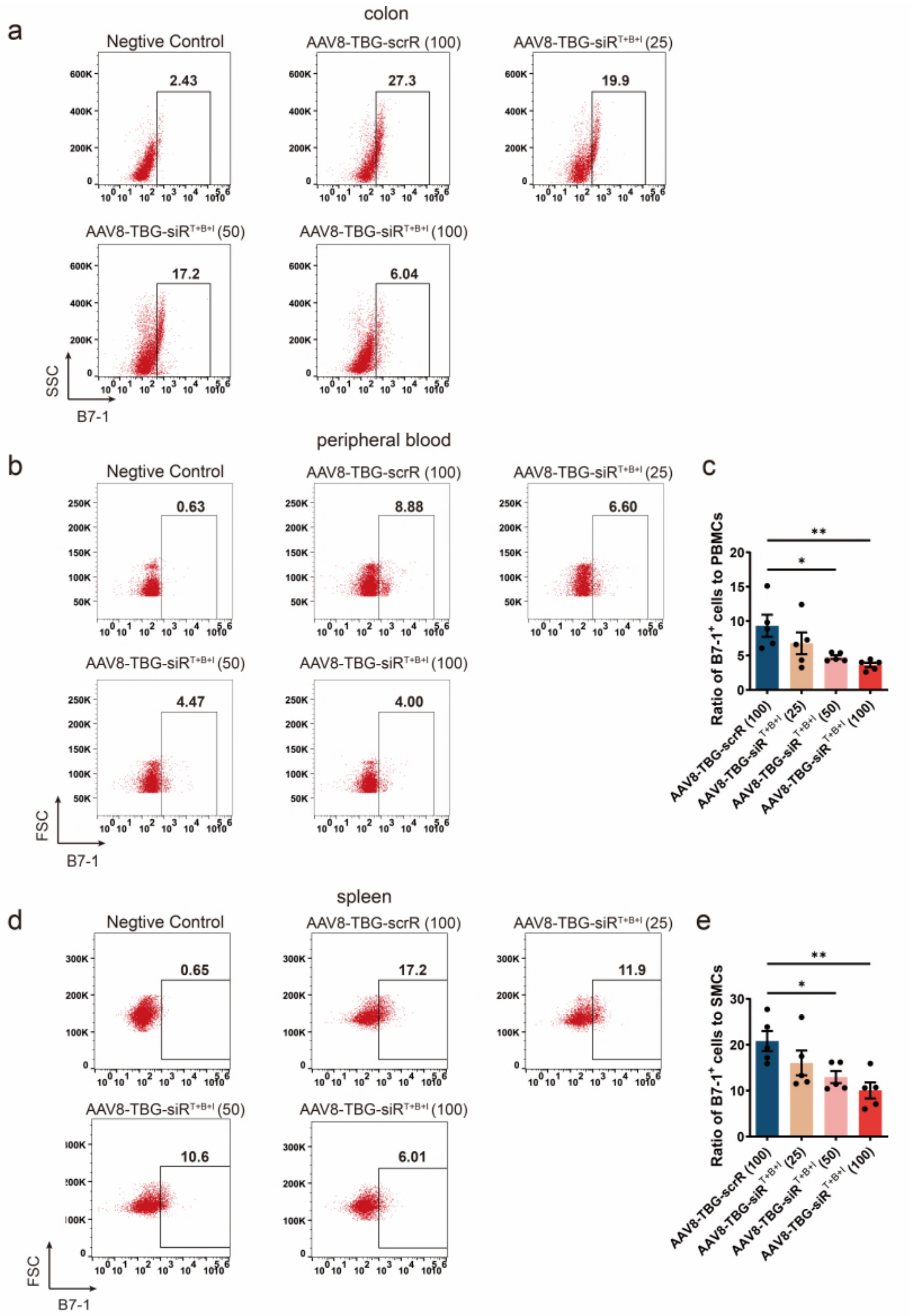

Supplementary Figure 21. Intravenous injection of the AAV8-TBG-siR^{T+B+I} results in loss of B7-1 protein on the membrane surface of mononuclear cells. (a) Representative flow cytometric plots of B7-1 on the surface of colonic lamina propria mononuclear cells. IgG isotype-labelled cells was used as a negative control. **(b)** Representative flow cytometric plots of B7-1 on the surface of peripheral blood mononuclear cells. IgG isotype-labelled cells was used as a negative control. **(c)** The population of B7-1^{+} cells in peripheral blood mononuclear cells (n = 5 in

each group). **(d)** Representative flow cytometric plots of B7-1 on the surface of splenic mononuclear cells. IgG isotype-labelled cells was used as a negative control. **(e)** The population of B7-1^{+} cells in splenic mononuclear cells (n = 5 in each group). Values are presented as the mean ± SEM. Significance was determined using one-way ANOVA followed by Dunnett's multiple comparison. * p < 0.05; ** p < 0.01.

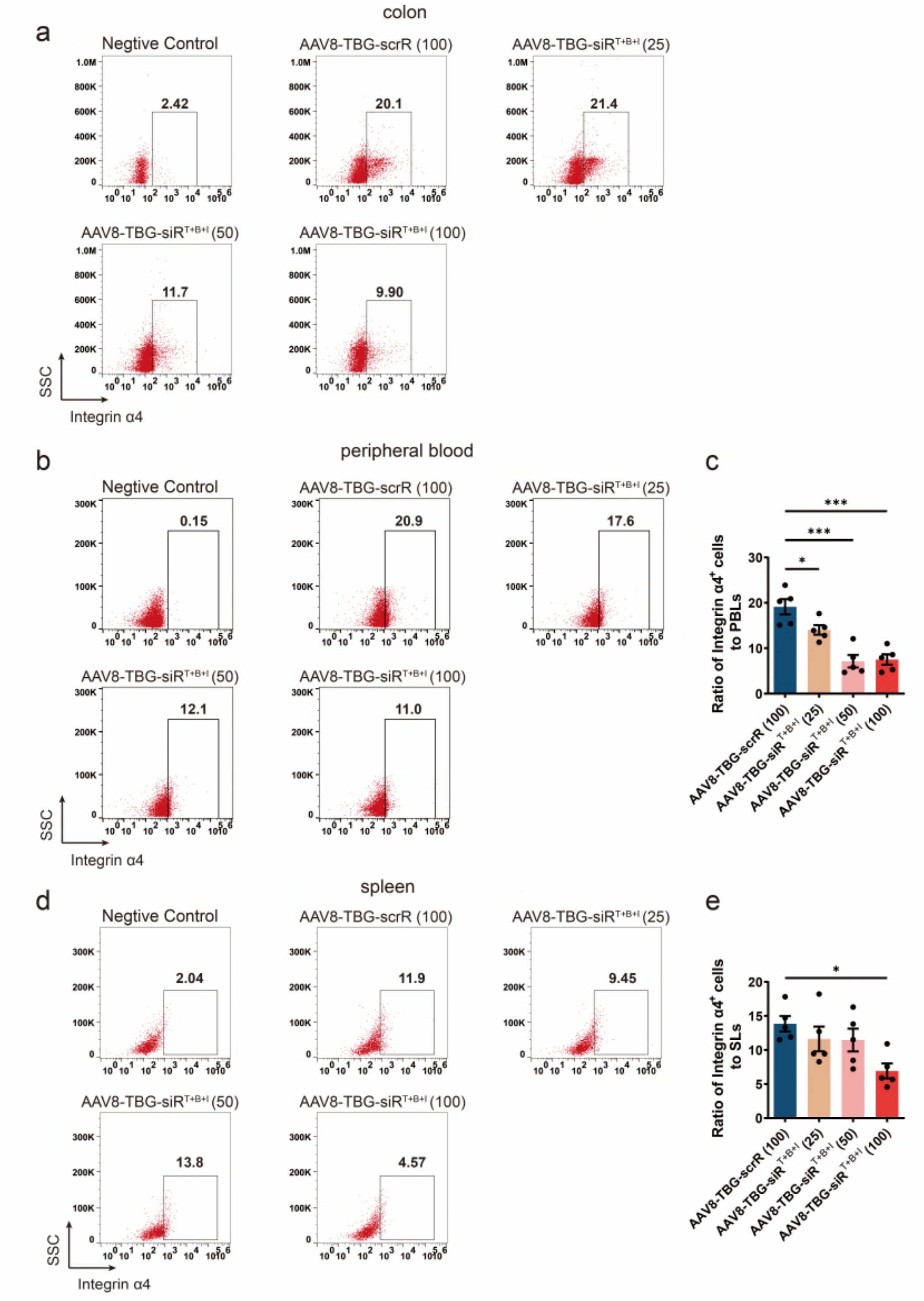

Supplementary Figure 22. Intravenous injection of the AAV8-TBG-siR^{T+B+I} results in loss of integrin α4 protein on the membrane surface of mononuclear cells. (a) Representative flow cytometric plots of integrin α4 on the surface of mononuclear cells derived from the colonic lamina propria. IgG isotype-labelled cells was used as a negative control. **(b)** Representative flow cytometric plots of integrin α4 on the surface of peripheral blood lymphocytes. IgG isotype-labelled cells was used as a negative control. **(c)** The population of integrin α4^{+} cells in peripheral blood lymphocytes (n = 5 in each group). **(d)** Representative flow cytometric plots of integrin α4 on the surface of splenic lymphocytes. IgG isotype-labelled cells was used as a negative control. **(e)** The population of integrin α4^{+} cells in splenic lymphocytes (n = 5 in each group). Values are presented as the mean ± SEM. Significance was determined using one-way ANOVA followed by Dunnett's multiple comparison. * $p < 0.05$; *** $p < 0.005$.

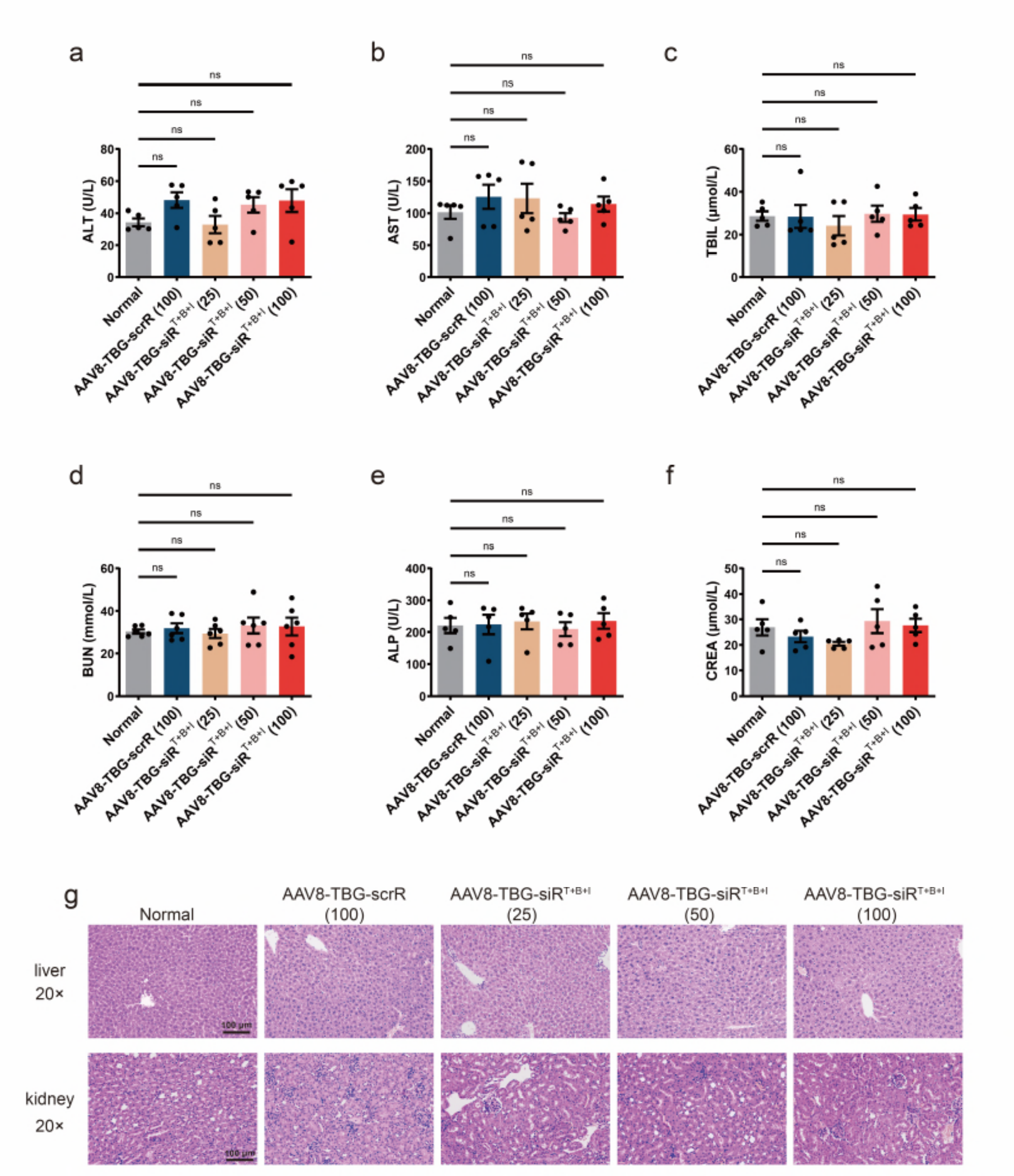

Supplementary Figure 23. Evaluation of the toxic effects and tissue damage in mice after intravenous injection of AAV8-TBG-siR^{T+B+I}. On week 0, BALB/c mice were intravenously injected with 100 μL AAV8-TBG-scrR (3.0 × 10^{12} V. G/mL) or 25, 50 or 100 μL AAV8-TBG-siR^{T+B+I} (3.0 × 10^{12} V. G/mL). At the same time, chronic UC was induced by rhythmically administering to mice 2.5% DSS for 1 week and water for 2 weeks and the cycle was repeated for 3 times. Untreated BALB/c mice were included as normal controls. After the treatment, mice were sacrificed, and blood and tissue samples were collected and analysed for serum biochemical indicators and tissue damage. **(a-f)** Measurement of representative serum biochemical indicators, including alanine aminotransferase (ALT), aspartate aminotransferase (AST), total bilirubin (TBIL), blood urea nitrogen (BUN), alkaline phosphatase (ALP) and creatinine (CREA), in the serum. **(g)** Histological examination of the liver and kidney. Scale bar: 100 μm. Values are presented as the mean ± SEM. Significance was determined using one-way ANOVA followed by Dunnett's multiple comparison. NS, not significant.

2. The liver siRNA expression/exosome release observed is quite short-lived as observed in Fig. 2B and F. What is the explanation for this? Is plasmid DNA degraded? Does the CMV promoter undergo silencing? Also here, a DNA biodistribution study would help understand the basis of such a pulsatile expression.

<u>**Response**</u>**:** We understand reviewer's concern regarding this issue. According to reviewer's suggestion, we have determined the amounts of the genetic circuits (plasmids) that were taken up by the liver. The CMV-siR$^{TNF-\alpha}$ circuit (in the form of naked DNA plasmid) with known concentration was serially diluted and assessed using quantitative PCR to generate a standard curve. Then the CMV-siR$^{TNF-\alpha}$ circuit was intravenously injected into DSS mice at the dose of 5 mg/kg. At 0, 1, 3, 6, 12 and 24 hours post-injection, mice were sacrificed, and liver, spleen and colon tissues were collected. Subsequently, the C_T values of CMV-siR$^{TNF-\alpha}$ circuit was determined in the DNA samples extracted from 10 mg liver, spleen and colon tissues at each time point. By referring to the standard curve, the concentration of CMV-siR$^{TNF-\alpha}$ circuit in the liver, spleen and colon was calculated. As can be seen in new Figure 2b, the CMV-siR$^{TNF-\alpha}$ circuit rapidly accumulated in the livers at 1 h then decreased to background levels at 6 h. No CMV-siR$^{TNF-\alpha}$ circuit was detected in other tissues of CMV-siR$^{TNF-\alpha}$-injected mice.

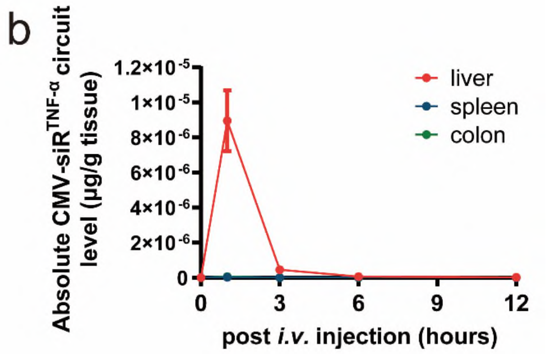

Figure 2. (b) Kinetics of CMV-siR$^{TNF-\alpha}$ circuit (DNA plasmid) in mouse liver, colon or spleen following tail vein injection of the 5 mg/kg CMV-siR$^{TNF-\alpha}$ circuit (n = 3 in each group). Values are presented as the mean ± SEM.

To further confirm that hepatocytes were the original site for the self-assembly and secretion of TNF-α siRNA-encapsulating sEVs, we isolated primary mouse hepatocytes at different time points following the injection of the CMV-siR$^{TNF-\alpha}$ circuit into mice and assessed TNF-α siRNA levels in sEVs derived from the culture medium of primary hepatocytes. TNF-α siRNA levels were higher in the sEVs of primary hepatocytes isolated from CMV-siR$^{TNF-\alpha}$ circuit-injected mice at 6 hours and were diminished in the sEVs of primary hepatocytes isolated from CMV-siR$^{TNF-\alpha}$ circuit-injected mice at 24 hours (Fig. 2j). These results indicate that the secretion of TNF-α siRNA-encapsulating sEVs increased after the injection of CMV-siR$^{TNF-\alpha}$ circuit, reached a peak at approximately 6 hours then declined to background at 24 hours in the *ex vivo* model. Overall, these results support the idea that the liver can assemble and secrete siRNA-encapsulating sEVs introduced by intravenously injected genetic circuits, although the genetic circuits (plasmids) undergo rapid DNA degradation or promoter silencing *in vivo*.

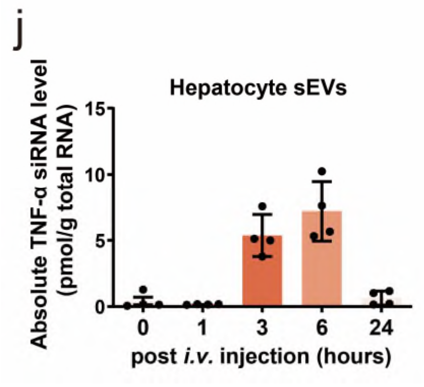

Figure 2. (j) Primary mouse hepatocytes were isolated from mice at different time points following intravenous injection of 5 mg/kg CMV-siR$^{TNF-\alpha}$ circuit. A quantitative RT-PCR assay was performed to assess TNF-α siRNA levels in sEVs derived from the culture medium of primary hepatocytes (n = 4 in each group). Values are presented as the mean ± SEM.

References

1 Groot, M. & Lee, H. Sorting Mechanisms for MicroRNAs into Extracellular Vesicles and Their Associated Diseases. *Cells* **9**, doi:10.3390/cells9041044 (2020).

2 Jiang, F. *et al.* Hepatocyte-derived extracellular vesicles promote endothelial inflammation and atherogenesis via microRNA-1. *Journal of Hepatology* **72**, 156-166, doi:10.1016/j.jhep.2019.09.014 (2020).

3 Nguyen, M.-A. *et al.* Extracellular Vesicles Secreted by Atherogenic Macrophages Transfer MicroRNA to Inhibit Cell Migration. *Arteriosclerosis Thrombosis and Vascular Biology* **38**, 49-63, doi:10.1161/atvbaha.117.309795 (2018).

4 Akers, J. C., Gonda, D., Kim, R., Carter, B. S. & Chen, C. C. Biogenesis of extracellular vesicles (EV): exosomes, microvesicles, retrovirus-like vesicles, and apoptotic bodies. *Journal of Neuro-Oncology* **113**, 1-11, doi:10.1007/s11060-013-1084-8 (2013).

5 Kalluri, R. & LeBleu, V. S. The biology, function, and biomedical applications of exosomes. *Science* **367**, 640-+, doi:10.1126/science.aau6977 (2020).

6 van Niel, G., D'Angelo, G. & Raposo, G. Shedding light on the cell biology of extracellular vesicles. *Nature Reviews Molecular Cell Biology* **19**, 213-228, doi:10.1038/nrm.2017.125 (2018).

7 Fu, Z. *et al.* In vivo self-assembled small RNAs as a new generation of RNAi therapeutics. *Cell Research*, doi:10.1038/s41422-021-00491-z (2021).

8 Garcia-Martin, R. *et al.* MicroRNA sequence codes for small extracellular vesicle release and cellular retention. *Nature* **601**, 446-+, doi:10.1038/s41586-021-04234-3 (2022).

9 Jeppesen, D. K. *et al.* Reassessment of Exosome Composition. *Cell* **177**, 428-+, doi:10.1016/j.cell.2019.02.029 (2019).

10 Kedmi, R. *et al.* A modular platform for targeted RNAi therapeutics. *Nature Nanotechnology* **13**, 214-+, doi:10.1038/s41565-017-0043-5 (2018).

11 Neurath, M. F. Current and emerging therapeutic targets for IBD. *Nature Reviews Gastroenterology & Hepatology* **14**, 269-278, doi:10.1038/nrgastro.2016.208 (2017).

12 Sandborn, W. J. & Hanauer, S. B. Antitumor necrosis factor therapy for inflammatory bowel disease: A review of agents, pharmacology, clinical results, and safety. *Inflammatory Bowel Diseases* **5**, 119-133, doi:10.1097/00054725-199905000-00008 (1999).

13 Burnett, J. C., Rossi, J. J. & Tiemann, K. Current progress of siRNA/shRNA therapeutics in clinical trials. *Biotechnology Journal* **6**, 1130-1146, doi:10.1002/biot.201100054 (2011).

14 Pecot, C. V., Calin, G. A., Coleman, R. L., Lopez-Berestein, G. & Sood, A. K. RNA interference in the clinic: challenges and future directions. *Nature Reviews Cancer* **11**, 59-67, doi:10.1038/nrc2966 (2011).

15 Vos, A. C. W. *et al.* Regulatory macrophages induced by infliximab are involved in healing in vivo and in vitro. *Inflammatory Bowel Diseases* **18**, 401-408, doi:10.1002/ibd.21818 (2012).

16 Bloemendaal, F. M. *et al.* Anti-Tumor Necrosis Factor With a Glyco-Engineered Fc-Region Has Increased Efficacy in Mice With Colitis. *Gastroenterology* **153**, 1351-+, doi:10.1053/j.gastro.2017.07.021 (2017).

17 Cader, M. Z. & Kaser, A. Recent advances in inflammatory bowel disease: mucosal immune cells in intestinal inflammation. *Gut* **62**, 1653-1664, doi:10.1136/gutjnl-2012-303955 (2013).

18 Hueber, W. *et al.* Secukinumab, a human anti-IL-17A monoclonal antibody, for moderate to severe Crohn's disease: unexpected results of a randomised, double-blind placebo-controlled trial. *Gut* **61**, 1693-1700, doi:10.1136/gutjnl-2011-301668 (2012).

19 Kuhn, R., Lohler, J., Rennick, D., Rajewsky, K. & Muller, W. Interleukin-10-deficient mice develop chronic enterocolitis *Cell* **75**, 263-274, doi:10.1016/0092-8674(93)80068-p (1993).

20 Greig, J. A. *et al.* Nonclinical Pharmacology/Toxicology Study of AAV8.TBG.mLDLR and AAV8.TBG.hLDLR in a Mouse Model of Homozygous Familial Hypercholesterolemia. *Human Gene Therapy Clinical Development* **28**, 28-38, doi:10.1089/humc.2017.007 (2017).

21 Kiourtis, C. *et al.* Specificity and off-target effects of AAV8-TBG viral vectors for the manipulation of hepatocellular gene expression in mice. *Biology Open* **10**, doi:10.1242/bio.058678 (2021).

22 Wang, L. *et al.* Systematic Evaluation of AAV Vectors for Liver-directed Gene Transfer in Murine Models. *Molecular Therapy* **18**, 118-125, doi:10.1038/mt.2009.246 (2010).

23 Zincarelli, C., Soltys, S., Rengo, G. & Rabinowitz, J. E. Analysis of AAV serotypes 1-9 mediated gene expression and tropism in mice after systemic injection. *Molecular Therapy* **16**, 1073-1080, doi:10.1038/mt.2008.76 (2008).

24 Sakurai, H., Kawabata, K., Sakurai, F., Nakagawa, S. & Mizuguchi, H. Innate immune response induced by gene delivery vectors. *International Journal of Pharmaceutics* **354**, 9-15, doi:10.1016/j.ijpharm.2007.06.012 (2008).

25 Liu, F. *et al.* Current Transport Systems and Clinical Applications for Small Interfering RNA (siRNA) Drugs. *Mol. Diagn. Ther.* **22**, 551-569, doi:10.1007/s40291-018-0338-8 (2018).

26 Wang, D., Tai, P. W. L. & Gao, G. Adeno-associated virus vector as a platform for gene therapy delivery. *Nature Reviews Drug Discovery* **18**, 358-378, doi:10.1038/s41573-019-0012-9 (2019).

27 Rahier, J. F. *et al.* Second European evidence-based consensus on the prevention, diagnosis and management of opportunistic infections in inflammatory bowel disease. *Journal of Crohns & Colitis* **8**, 443-468, doi:10.1016/j.crohns.2013.12.013 (2014).

28 Miehsler, W. *et al.* A decade of infliximab: The Austrian evidence based consensus on the safe use of infliximab in inflammatory bowel disease. *Journal of Crohns & Colitis* **4**, 221-256, doi:10.1016/j.crohns.2009.12.001 (2010).

29 Furst, D. E., Cush, J., Kaufmann, S., Siegel, J. & Kurth, R. Preliminary guidelines for diagnosing and treating tuberculosis in patients with rheumatoid arthritis in immunosuppressive trials or being treated with biological agents. *Annals of the Rheumatic Diseases* **61**, 62-63 (2002).

REVIEWER COMMENTS

Reviewer #1 (Remarks to the Author):

The authors have addressed some of my concerns. However, two major points remain:
- The authors claim in their revised manuscript that TNF-a siRNA is almost exclusively present in sEVs and not in sEV-free plasma. However, for almost all miRNAs (and siRNAs), it has been shown that the majority in plasma is found in Ago complexes and not in EVs (see e.g. PMID 32929008). Can the authors show that Ago complexes are not co-isolated with their EVs, but are present in the sEV-free plasma?
- The authors seem to agree with my statement that concentrations of siRNA in colon and spleen are cumulatively similar or even higher than in liver. This is difficult to envision using the model proposed by the authors, where (1) only a small fraction of siRNA will be packaged into exosomes/EVs and secreted, and (2) those siRNAs will be further diluted and distributed throughout the body. In the light of their model, can the authors discuss a scenario where this would be possible?

Reviewer #2 (Remarks to the Author):

The manuscript was throughly revised according to the comments of all reviewers and editor, and my questions were also addressed.

Reviewer #3 (Remarks to the Author):

The authors addressed all my comments and concerns successfully and the modifications made have added to the value of the manuscript. The additional experiments performed by the authors strengthen the statements, and limitations of the study are now clearly stated in the discussion.

Reviewer #4 (Remarks to the Author):

The authors have addressed all the reviewer concerns with new experimental data that prove liver-mediated expression of exosomes and provide information on their biodistribution/pharmacokinetics. The manuscript is considerably improved by this new data.

Point-by-Point Response to the Reviewers' Comments:

Reviewer #1

1. The authors claim in their revised manuscript that TNF-a siRNA is almost exclusively present in sEVs and not in sEV-free plasma. However, for almost all miRNAs (and siRNAs), it has been shown that the majority in plasma is found in Ago complexes and not in EVs (see e.g. PMID 32929008). Can the authors show that Ago complexes are not co-isolated with their EVs, but are present in the sEV-free plasma?

<u>**Response**</u>**:** We appreciate reviewer's emphasis on this critical issue. Previous studies have raised two possible mechanisms for the stability of circulating miRNAs despite the presence of ubiquitous RNases in the blood: (1) protection of miRNAs by the membrane structures of EVs;[1-3] and (2) stabilization of miRNAs by their association with RNA-binding proteins, such as Argonaute-2 (Ago2).[4,5] We totally agree with the reviewer that it is important to dertermine which mechanism – sEV membrane enclosure or Ago2 conjunction – may protect TNF-α siRNA from degradation by RNases in the blood. Therefore, we investigated the mechanism of Ago2 conjunction in the revised manuscript. The plasma of CMV-scrR circuit- or CMV-siR$^{TNF\text{-}\alpha}$ circuit-injected DSS mice was immunoprecipitated using control IgG or anti-Ago2 antibody, and the Ago2 and IgG immunoprecipitates were assayed for miR-16 and TNF-α siRNA to determine if the TNF-α siRNA is associated with Ago2 protein in blood circulation. Western blot analysis showed a specific Ago2 band at ~97 kDa in the Ago2 immunoprecipitates, while no Ago2 was detected in IgG immunoprecipitates and in the supernatant immunoprecipitated with anti-Ago2 antibody (Supplementary Fig. 3), demonstrating that Ago2 was specifically precipitated from plasma. Quantitative RT-PCR analysis revealed that miR-16 was largely depleted from plasma by Ago2 immunoprecipitation compared with control IgG immunoprecipitation and that the depleted miR-16 could be efficiently recovered from the immunoprecipitated beads (Fig. 2g). In contrast, no TNF-α siRNA seemed to be immunoprecipitated from the plasma by anti-Ago2 antibody, and Ago2 immunoprecipitates were almost totally free of TNF-α siRNA (Fig. 2h). Thus, unlike the miR-16 that is predominantly associated with Ago2 protein in blood plasma, TNF-α siRNA may be protected by a mechanism other than conjunction with Ago2 protein. Because we have shown that TNF-α siRNA was mainly detected in the plasma sEVs bot not in the sEV-free plasma of CMV-siR$^{TNF\text{-}\alpha}$-injected mice, and because we have characterised the protective roles of the lipid bilayer structure of TNF-α siRNA-encapsulating sEVs, sEV membrane enclosure, rather than Ago2 conjunction, may provide a protected and controlled internal microenvironment for TNF-α siRNA, allowing it to travel in the blood without degradation by RNases. Considering that miR-155 has been identified to be associated predominantly with sEV-rich fraction in the plasma and that Tables S2 in PMID 32929008 also supports this feature of miR-155,[6-9] TNF-α siRNA, generated through the pre-miR-155 backbone, might be released to the blood in a similar, sEV-dependent manner.

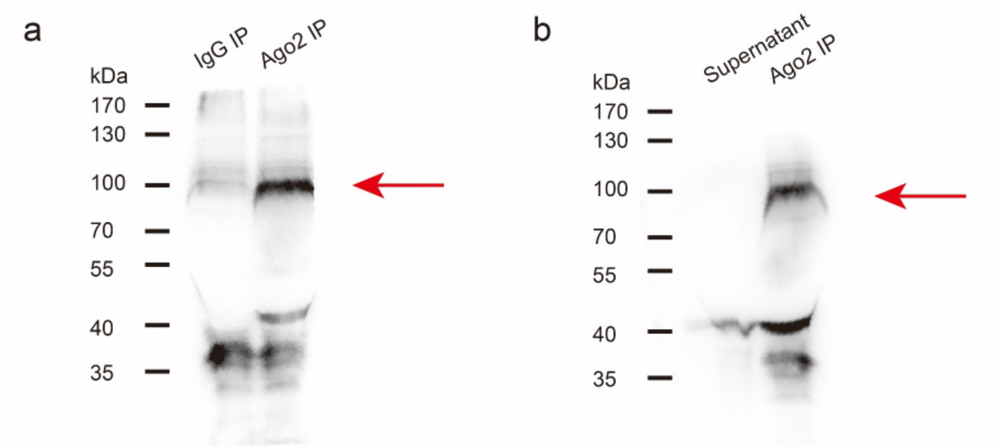

Supplementary Figure 3. Western blots probed with an anti-Ago2 antibody to assess the Ago2 associated with the captured beads following Ago2 immunoprecipitation. Bead-conjugated anti-Ago2 antibody was incubated with the plasma of CMV-scrR circuit- or CMV-siR$^{TNF-\alpha}$ circuit-injected mice under native conditions. The beads were separated by centrifuging and processed for protein extraction and subsequent Western blotting using a rabbit anti-Ago2 antibody. Total protein extracted from the supernatant was also subjected to Western blot analysis to determine the amounts of Ago2. The IgG antibody was used as a negative control. **(a)** Red arrow indicating an Ago2 band at ~97 kDa in the Ago2 immunoprecipitates but not in the IgG immunoprecipitates. **(b)** Red arrow indicating an Ago2 band at ~97 kDa in the Ago2 immunoprecipitates but not in the supernatant.

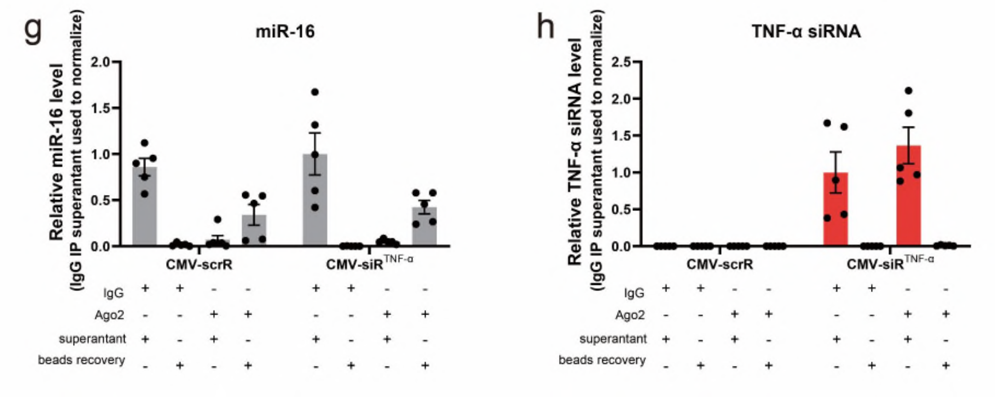

Figure 2. (g and h) Bead-conjugated anti-Ago2 antibody was incubated with the plasma of CMV-scrR circuit- or CMV-siR$^{TNF-\alpha}$ circuit-injected mice under native conditions. The beads were removed by centrifuging, and RNA extracted from the supernatant was subjected to quantitative RT-PCR analysis to determine the amounts of miR-16 and TNF-α siRNA depleted by Ago2 immunoprecipitation. The beads were processed for RNA extraction followed by quantitative RT-PCR analysis to assess the recovery rates of miR-16 and TNF-α siRNA (n = 5 in each group).

2. The authors seem to agree with my statement that concentrations of siRNA in colon and spleen are cumulatively similar or even higher than in liver. This is difficult to envision using the model proposed by the authors, where (1) only a small fraction of siRNA will be packaged into exosomes/EVs and secreted, and (2) those siRNAs will be further diluted and distributed throughout

the body. In the light of their model, can the authors discuss a scenario where this would be possible?

<u>Response</u>: We apologize for the confusion caused by the unit we used in the original manuscript. When comparing the levels of siRNA in different organs, either relative or absolute quantification can be used. For relative quantification, an internal control gene is needed as a reference to calculate the relative changes of the target siRNA. In our study, U6 was chosen as the reference gene at first; however, our results revealed that the expression levels of U6 were not stable between different tissues. Therefore, it is difficult to compare siRNA levels between different tissues using relative quantification. We then performed absolute quantification and calculated the siRNA levels in each tissue as pmol of siRNA per μg of total RNA (pmol/μg total RNA). It seems that this unit is still confusing and not suitable for comparison between different tissues. In the revised manuscript, we used a new quantification method and re-calculated the total siRNA levels in liver, spleen and colon based on the total amount of RNA isolated from one gram of each tissue and the siRNA content in one μg of total RNA. Our results showed the total siRNA level in the liver is much higher than that in the spleen or the colon, which is in accordance with the model we proposed——the sEV-encapsulated siRNA is generated by the liver and transported to other tissues through circulation.

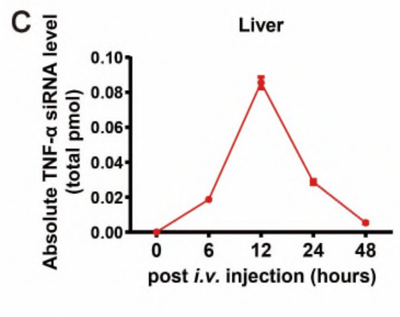

Figure 2. (c) Kinetics of TNF-α siRNA in the mouse liver following tail vein injection of the 5 mg/kg CMV-siR$^{TNF\text{-}\alpha}$ circuit (n = 3 in each group). The total TNF-α siRNA level in the liver was calculated based on the total amount of RNA isolated from one gram of liver and the TNF-α siRNA content in one μg of total RNA.

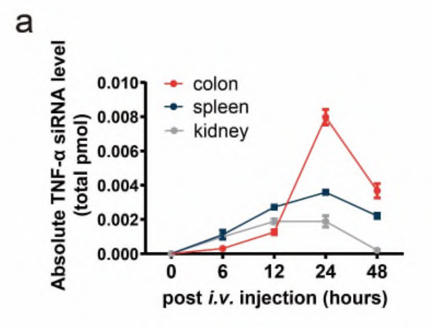

Figure 3. (a) Kinetics of TNF-α siRNA in the mouse colon, spleen and kidney following tail vein injection of the 5 mg/kg CMV-siR$^{TNF\text{-}\alpha}$ circuit (n = 3 in each group). The total TNF-α siRNA levels in the colon, spleen and kidney were calculated based on the total amount of RNA isolated from one gram of each tissue and the TNF-α siRNA content in one μg of total RNA.

Reviewer #2

1. The manuscript was thoroughly revised according to the comments of all reviewers and editor, and my questions were also addressed.

Response: We greatly thank the reviewer for these positive comments.

Reviewer #3

1. The authors addressed all my comments and concerns successfully and the modifications made have added to the value of the manuscript. The additional experiments performed by the authors strengthen the statements, and limitations of the study are now clearly stated in the discussion.

Response: We appreciate the reviewer for these positive comments.

Reviewer #4

1. The authors have addressed all the reviewer concerns with new experimental data that prove liver-mediated expression of exosomes and provide information on their biodistribution/pharmacokinetics. The manuscript is considerably improved by this new data.

Response: We greatly thank the reviewer for these positive comments.

References

1 Zhang, J. *et al.* Exosome and Exosomal MicroRNA: Trafficking, Sorting, and Function. *Genomics Proteomics & Bioinformatics* **13**, 17-24, doi:10.1016/j.gpb.2015.02.001 (2015).

2 Mori, M. A., Ludwig, R. G., Garcia-Martin, R., Brandao, B. B. & Kahn, C. R. Extracellular miRNAs: From Biomarkers to Mediators of Physiology and Disease. *Cell Metabolism* **30**, 656-673, doi:10.1016/j.cmet.2019.07.011 (2019).

3 Li, L. *et al.* Argonaute 2 Complexes Selectively Protect the Circulating MicroRNAs in Cell-Secreted Microvesicles. *Plos One* **7**, doi:10.1371/journal.pone.0046957 (2012).

4 Turchinovich, A., Weiz, L., Langheinz, A. & Burwinkel, B. Characterization of extracellular circulating microRNA. *Nucleic Acids Research* **39**, 7223-7233, doi:10.1093/nar/gkr254 (2011).

5 Geekiyanage, H., Rayatpisheh, S., Wohlschlegel, J. A., Brown, R. & Ambros, V. Extracellular microRNAs in human circulation are associated with miRISC complexes that are accessible to anti-AGO2 antibody and can bind target mimic oligonucleotides. *Proceedings of the National Academy of Sciences of the United States of America* **117**, 24213-24223, doi:10.1073/pnas.2008323117 (2020).

6 Ying, W. *et al.* Adipose Tissue Macrophage-Derived Exosomal miRNAs Can Modulate In Vivo and In Vitro Insulin Sensitivity. *Cell* **171**, 372-+, doi:10.1016/j.cell.2017.08.035 (2017).

7 Lan, J. *et al.* M2 Macrophage-Derived Exosomes Promote Cell Migration and Invasion in Colon Cancer. *Cancer Research* **79**, 146-158, doi:10.1158/0008-5472.Can-18-0014 (2019).

8 Bala, S. *et al.* Circulating microRNAs in exosomes indicate hepatocyte injury and inflammation in alcoholic, drug-induced, and inflammatory liver diseases. *Hepatology* **56**, 1946-1957, doi:10.1002/hep.25873 (2012).

9 Alexander, M. *et al.* Exosome-delivered microRNAs modulate the inflammatory response to endotoxin. *Nature Communications* **6**, doi:10.1038/ncomms8321 (2015).

REVIEWERS' COMMENTS

Reviewer #1 (Remarks to the Author):

The authors have adequately addressed my previous concerns.
